# Application of sSPhos as a Chiral Ligand for Palladium-Catalyzed Asymmetric Allylic Alkylation

**DOI:** 10.1021/acs.orglett.3c04025

**Published:** 2023-12-26

**Authors:** Philip J. Docherty, Max Kadarauch, Nisha Mistry, Robert J. Phipps

**Affiliations:** † Yusuf Hamied Department of Chemistry, University of Cambridge, Lensfield Road, Cambridge CB2 1EW, U.K.; ‡ Drug Substance Development, GSK, Stevenage SG1 2NY, U.K.

## Abstract

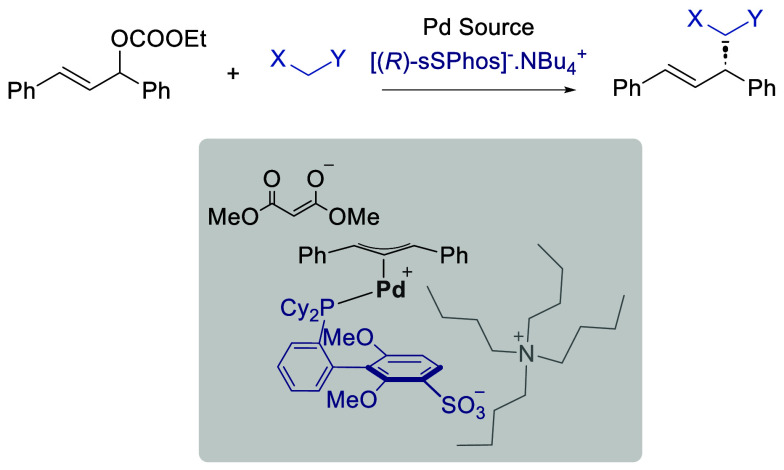

Palladium-catalyzed asymmetric allylic alkylation is a versatile method for C–C bond formation. Many established classes of chiral ligands can perform allylic alkylation reactions enantioselectively, but identification of new ligand classes remains important for future development of the field. We demonstrate that enantiopure sSPhos, a bifunctional chiral monophosphine ligand, when used as its tetrabutyl ammonium salt, is a highly effective ligand for a benchmark Pd-catalyzed allylic alkylation reaction. We explore the scope and limitations and perform experiments to probe the origin of selectivity. In contrast with reactions previously explored using enantiopure sSPhos, it appears that steric bulk around the sulfonate group is responsible for the high enantioselectivity in this case, rather than attractive noncovalent interactions.

Within the broad field of palladium catalysis, allylic alkylation, also termed the Tsuji–Trost reaction, is a process that arguably offers the most versatility in terms of mechanistic possibilities and the ability to combine with other reaction types. It often affords chiral products, offering numerous possibilities for asymmetric synthesis through various mechanistic manifolds.^1^ A number of important classes of chiral ligands have been developed over the years, but it is important that new ligand exploration continues.^1f^ While the benchmark reactions may be well served, innovative new transformations based on π-allyl palladium chemistry continue to be developed, for which well-established ligand scaffolds may not suffice. We are interested in designing ligands for transition-metal-catalyzed reactions that harness attractive noncovalent interactions between the ligand and substrate to control selectivity, a strategy that can be very powerful.^2^ Ligands that incorporate noncovalent interactions into their outer coordination sphere have been applied to π-allyl palladium chemistry in the past. Strategies have included the modification of established ligands with pendant functional groups,^3^ the pairing of a chiral anion with a cationic palladium complex,^4^ and the pairing of chiral anions with cationic ligands.^5,6^

We have recently explored the use of sulfonated phosphine ligand sSPhos, originally reported in racemic form by Anderson and Buchwald to permit water solubility,^7^ as a bifunctional ligand (Figure 1A). We first used sSPhos in racemic form for the control of site selectivity in cross-coupling reactions (Figure 1B, left).^8^ Having developed a method for obtaining sSPhos in enantiopure form via resolution using quinidine (Figure 1A, right), we subsequently used sSPhos in enantiopure form to control enantioselectivity in Suzuki–Miyaura cross-coupling reactions to form 2,2′-biphenols, as well as in arylative dearomatization reactions to afford a range of scaffolds (Figure 1B, center and right).^9^ In all cases, we believe that attractive noncovalent interactions involving the ligand sulfonate group are required for selectivity. We propose either electrostatic interactions or hydrogen bonding interactions depending on the specific reaction and conditions.^10^ Our previous work with sSPhos has highlighted several beneficial properties: its high reactivity in many Pd-catalyzed processes by virtue of its dialkylbiaryl structure,^11^ as well as its ability to engage in attractive interactions, while also being intrinsically chiral and, as we have previously demonstrated,^9a^ resolvable.

**Figure 1:**
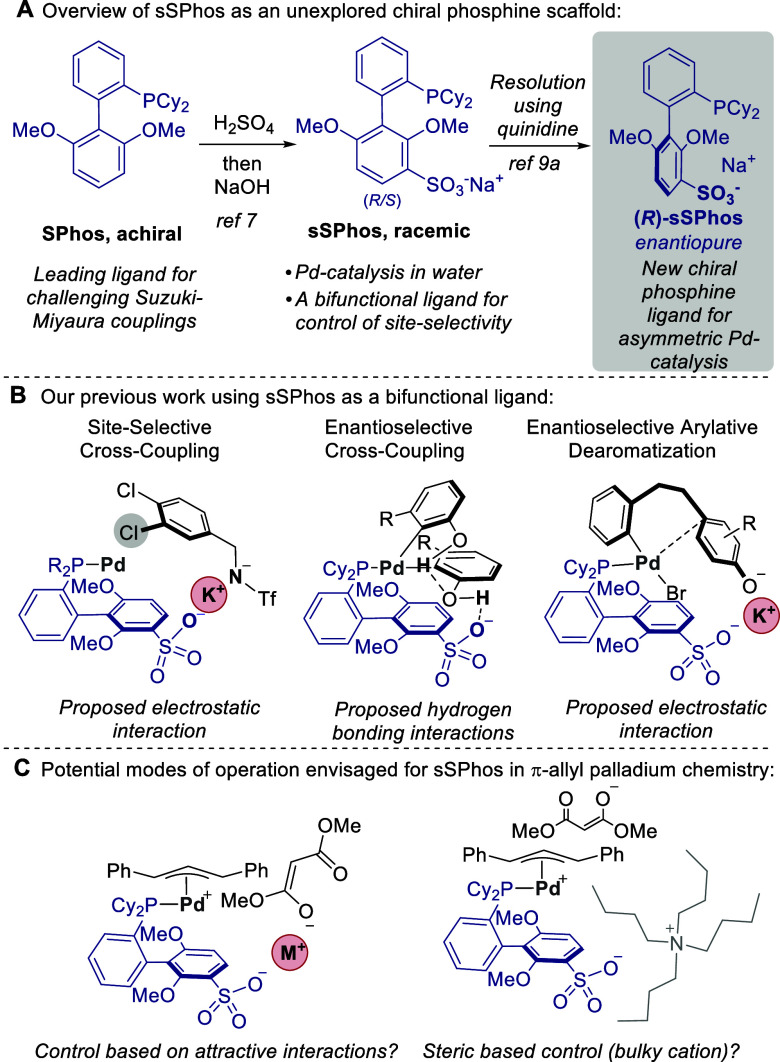
Use of sSPhos for selectivity control and potential application to π-allyl palladium chemistry.

At the outset of this study, we sought to evaluate the ability of sSPhos to act as a new class of chiral phosphine ligands for asymmetric allylic alkylation. We hypothesized that this reaction type may be particularly amenable to enantioinduction using sSPhos, as it typically features a cationic intermediate prior to outer sphere nucleophilic attack from an anionic nucleophile. We speculated that the discrete charges on several reaction components may offer possibilities for leveraging attractive ionic interactions to enable organization in the enantiodetermining transition state. One possibility that we considered, akin to our previous work on site-selective cross-coupling,^8^ was that the cation associated with an anionic nucleophile might engage in attractive electrostatic interactions with the ligand sulfonate group (Figure 1C, left). Another possibility was that steric control would predominate if the cation associated with sSPhos were bulky, such as tetra-*n*-butylammonium (Figure 1C, right). This steric effect on selectivity of an associated cation is one that we^12^ and others^13^ have previously exploited in the context of Ir-catalyzed borylation.

We commenced our study with the benchmark reaction of allylic carbonate **1a**, which reacts via a symmetrical intermediate. We opted to use an allylic carbonate electrophile, as opposed to an acetate, which would allow the reaction to proceed via a decarboxylative mechanism.^14^ This would avoid the need for an exogenous base, which could complicate any potential ionic interactions with the presence of additional anions and cations in the reaction mixture. With the tetra-*n*-butylammonium salt of (*R*)-sSPhos as the ligand, we were pleased to find that using several dimeric Pd sources, good yields and enantioselectivities could be obtained (Table 1, entries 1–3). A solvent evaluation showed a range of solvents to be compatible, and we opted to continue with tetrahydrofuran (see the Supporting Information for more details). A 1 mmol scale reaction was also carried out in which high ee was retained (see the Supporting Information). At this point, we systematically varied the cation associated with (*R*)-sSPhos, moving away from Bu_4_N^+^. Interestingly, the three alkali metal cations evaluated, including Na^+^, the default cation for sSPhos, all gave markedly reduced ee’s compared with that with Bu_4_N^+^ (entries 4–6). We then evaluated the effect of modulating the length of the alkyl chains of the tetraalkylammonium cation. Shorter alkyl chains decreased the ee significantly (methyl and ethyl, entries 7 and 8, respectively), while a larger one gave a result similar to that of Bu_4_N^+^, suggesting a possible size effect that may have plateaued with Bu_4_N^+^ (*n*-hexyl, entry 9). This apparent effect of cation size led us to consider a cation that may appear larger than an ammonium bearing linear alkyl chains. Accordingly, we paired (*R*)-sSPhos with a quinine-derived cation with a large quaternizing group, of the type used extensively in phase-transfer catalysis.^15^ We have used these cations in recent work paired with sulfonated ligands for Ir^16^ and Rh,^17^ and others have used these cations to combine asymmetric phase-transfer catalysis with palladium-catalyzed π-allyl chemistry.^18^ Gratifyingly, this increased the ee from 84% to 90% (entry 10). We were intrigued about whether the chirality of the cation might be contributing to this increased enantioselectivity and next probed whether there might be a matched–mismatched effect.^19^ (*S*)-sSPhos was therefore paired with the same chiral cation **A**^**+**^, and this catalyst resulted in a product ee that was −90%, exactly equal and opposite of the result using (*R*)-sSPhos with the same chiral cation (entry 11). These results suggest that the chirality of the cation is not contributing to the increase in enantioselectivity. We believe, therefore, that the cation effect is most likely a steric one.

**Table 1:**
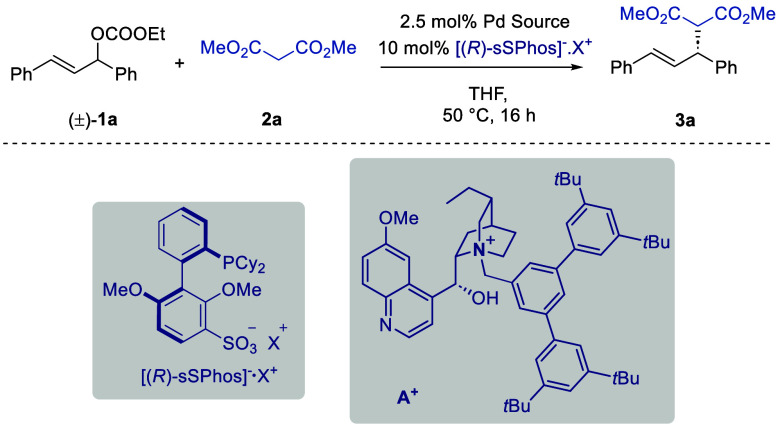
Optimization of the Allylic Alkylation Reaction Using sSPhos and Allylic Carbonate **1a**

entry	Pd source	X^+^	yield^a^ (%)	ee^a^ (%)
1	[Pd(allyl)Cl]_2_	Bu_4_N^+^	81	80
2	Pd_2_dba_3_	Bu_4_N^+^	90 (91)	84
3	[Pd(cinnamyl)Cl]_2_	Bu_4_N^+^	42	84
4	Pd_2_dba_3_	Na^+^	82	44
5	Pd_2_dba_3_	K^+^	96	32
6	Pd_2_dba_3_	Cs^+^	79	36
7	Pd_2_dba_3_	NMe_4_^+^	66	42
8	Pd_2_dba_3_	NEt_4_^+^	72	40
9	Pd_2_dba_3_	NHex_4_^+^	71	82
10	Pd_2_dba_3_	**A**^+^	79	90
11^b^	Pd_2_dba_3_	**A**^+^	68	–90

a Yields determined by ^1^H NMR with an internal standard. The value in parentheses refers to the isolated yield. ee determined by SFC analysis of the crude reaction mixture, except for entry 2, which was isolated.

b Using (*S*)-sSPhos paired with **A**^**+**^_._

We next evaluated the scope of the transformation with respect to a diverse range of acidic carbon-based nucleophiles (Scheme 1). During the optimization, only a small increase in ee was observed upon switching NBu_4_^+^ for chiral cation **A**^**+**^, at the expense of a great increase in complexity. We therefore decided to use (*R*)-NBu_4_·sSPhos when evaluating the scope of the transformation, being optimistic that excellent enantioselectivities might still be achievable with Bu_4_N^+^. We were pleased to find that the reaction was very tolerant of a broad range of variously substituted carbon nucleophiles. The absolute stereochemistry of **3a** could be readily determined by comparison with the literature, and the others are assigned by analogy (see the Supporting Information for details). An alkyl group on the malonate ester gave 90% ee (**3b**). A diketone gave slightly reduced but still useful levels of selectivity (**3c**, 78% ee). Nitroalkanes worked well (**3d** and **3e**). In the case of **3e**, which affords diastereomers, no diastereocontrol was observed but each could be isolated separately with an identical ee. A glycine-derived Schiff base also worked very well (**3f**). Similarly, no diastereocontrol was observed, but each diastereomer could be separately isolated in high ee. Excellent enantioselectivity outcomes were obtained for β-ketoester substrates (**3g** and **3h**), as well as β-nitroester substrates (**3i** and **3j**), emphasizing the breadth of nucleophiles with which sSPhos is compatible. We did identify limitations. For example, diphenylpropanedione had very low reactivity and required heating to 120 °C in toluene to obtain a reasonable yield (**3k**). As expected, at this high temperature, the enantioselectivity of the product was reduced. A bis-sulfone was reactive but gave poor enantioselectivity at 27% ee (**3l**), an outcome similar to that observed with indole (**3m**). A fluorinated ester gave no conversion (**3n**). We also evaluated a phenol as a heteroatom nucleophile (**3o**), for which some ee has been obtainable with other catalyst systems.^20^ In our case, it gave a racemate, the reason for which is presently unclear.

**Scheme 1:**
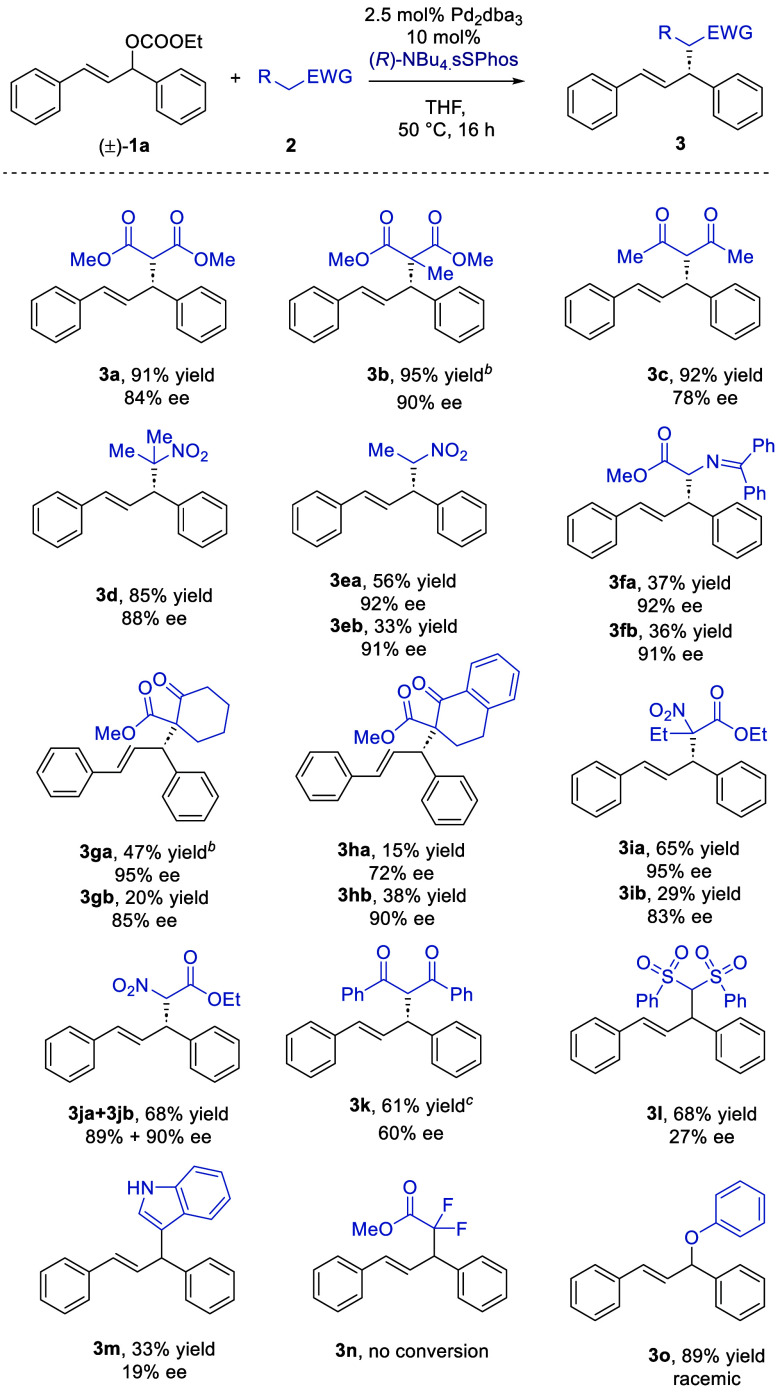
Scope of the Reaction Yields are isolated; ee values determined by SFC. Isolated with an inseparable dibenzylideneacetone impurity. See the Supporting Information for details. Reaction caried out in toluene at 120 °C.

As the scope survey indicated, excellent ee values were often obtainable using tetra-*n*-butylammonium as the cation associated with sSPhos, without the need to incorporate the more complex quinine-derived chiral cation that slightly improved the ee during the optimization. However, we were keen to explore whether the chiral cation might be able to influence diastereoselectivity for substrate combinations that feature a prochiral nucleophile. Cyclic β-ketoester **2g** was evaluated using chiral cation **A**^**+**^ paired with both (*R*)-sSPhos and (*S*)-sSPhos, to probe any matched–mismatched effect. However, the enantioselectivity outcomes were similar, and the bulky chiral cation did not significantly influence the dr (Scheme 2A). On the basis of the excellent ee values obtained with non-prochiral nucleophiles, we presumed that the poor diastereoselectivity arising with prochiral nucleophiles was a consequence of a lack of catalyst control over the stereochemistry originating from the nucleophile. Support for this was provided by an experiment with allylic carbonate **1b** and prochiral β-ketoester **2g** (Scheme 2B). In this case, a stereocenter is formed only on the nucleophilic component, and this occurred with almost no control (8% ee). We sought to test allylic carbonate **1c**, which would give rise to a nonsymmetric π-allyl intermediate and for which some ligands have shown promise in the past (Scheme 2C).^21^ However, **3q** was obtained with only 6% ee. Finally, we evaluated an (*R*)-sSPhos derivative in which the sulfonate group is esterified as a neopentyl sulfonate ester (Scheme 2D). This would preclude it from engaging in electrostatic interactions and weaken its ability to act as a hydrogen bond acceptor, meaning that any enantioselectivity would likely arise due to repulsive steric effects at the transition state. Use of (*R*)-sSPhos-Np gave **3a** in a very high 90% ee, supporting our sterics-based hypothesis for the control of enantioselectivity (Figure 1C, right). This stands in contrast to our previous reports of cross-coupling and arylative dearomatization, where sulfonate ester variants of (*R*)-sSPhos gave negligible ee, providing support in those cases for attractive noncovalent interactions.^9^

**Scheme 2:**
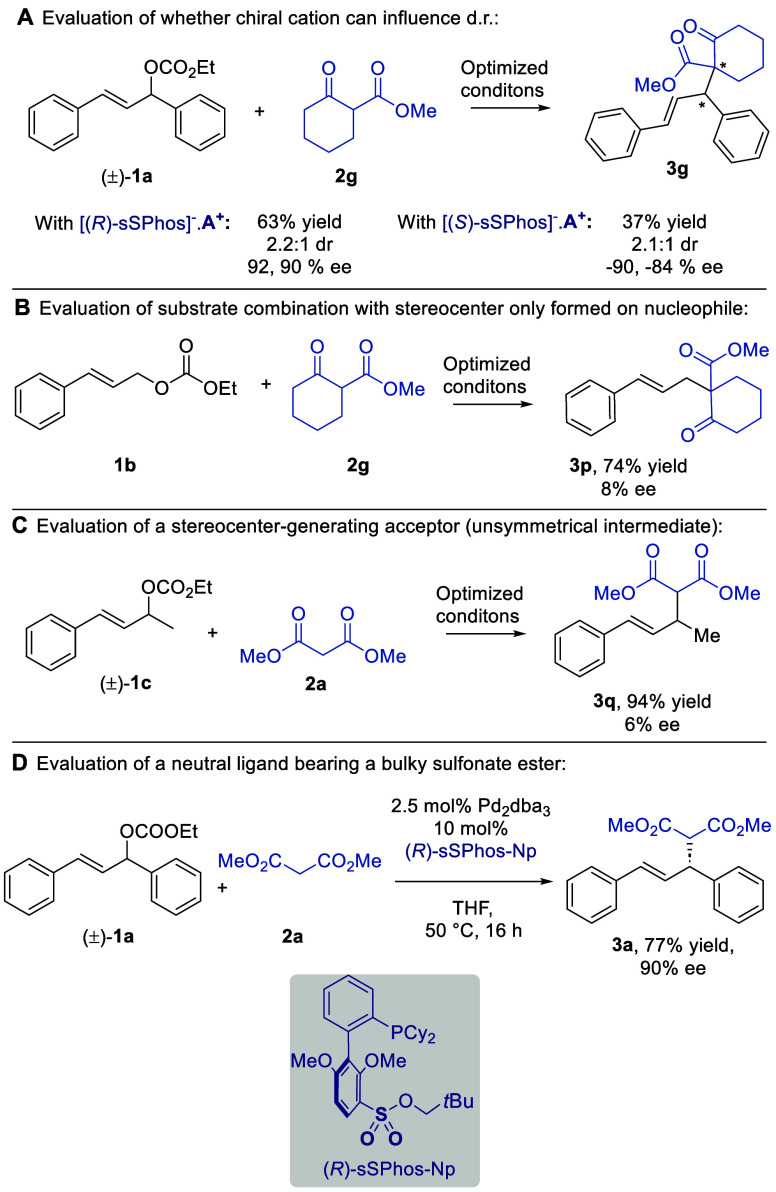
Further Experiments and Substrate Classes Examined

In summary, we report that enantiopure sSPhos, with tetra-*n*-butylammonium as the cation, is an excellent ligand in the palladium-catalyzed asymmetric allylic alkylation of bis-phenyl allylic carbonate **1a** with a variety of carbon-based nucleophiles. Prochiral nucleophiles resulted in diastereomers, but these could, in most cases, be independently isolated on silica. Our experiments suggest that sSPhos is proficient at controlling the stereochemistry on the electrophilic component but not on a prochiral nucleophile. We had initially considered the possibility that the incoming anionic nucleophile might engage in electrostatic interactions with the cation paired with the ligand sulfonate group. However, the lack of control over forming stereocenters on the nucleophile led us to believe this is probably less likely than sterically based control. The high enantioselectivity obtained with (*R*)-sSPhos-Np provided further support for the sterically based model. We envisage that the competence of sSPhos as a chiral ligand for π-allyl palladium chemistry will prompt others to explore it when developing new reactions based on this important mechanistic pathway.

## Data Availability

The data underlying this study are available in the published article and its Supporting Information.

## Supporting Information Available

Additional optimization, full experimental details, and characterization data for compounds (PDF)
