# Supplementary material for: Application of sSPhos as a Chiral Ligand for Palladium-Catalyzed Asymmetric Allylic Alkylation
Source: Org Lett. 2023 Dec 26;26(14):2862–6. doi: 10.1021/acs.orglett.3c04025 (PMC11020163; doi:10.1021/acs.orglett.3c04025)

Supporting Information

Application of sSPhos as a Chiral Ligand for Palladium-Catalyzed Asymmetric Allylic Alkylation

Philip J. Docherty,[a†] Max Kadarauch,[a†] Nisha Mistry[b] and Robert J. Phipps[a*]

[a] Yusuf Hamied Department of Chemistry, University of Cambridge, Lensfield Road, Cambridge, CB2 1EW, United Kingdom.

[b] Drug Substance Development, GSK, Stevenage, United Kingdom, SG1 2NY

† These authors contributed equally.

Table of Contents

General Experimental Information 3
Extended Reaction Optimization Data 5
Synthesis of Allylic Electrophile Starting Materials 6
Synthesis of Nucleophile Starting Materials 9
Synthesis of Ligands 9
Synthesis of Products 17
References 31
NMR Spectra 33
Chiral SFC Traces 109

General Experimental Information

Reaction Setup, Solvents and Reagents: All reactions were carried out under an inert argon or nitrogen atmosphere using standard Schlenk techniques. All reagents were used as supplied from commercial sources without further purification unless otherwise stated. Tetrahydrofuran (THF), Et_2O, MeOH, MeCN, dichloromethane (CH_2Cl_2), *n*-hexane and toluene were purified by distillation on site under inert atmosphere via the following processes: THF and Et_2O were pre-dried over sodium wire then distilled from calcium hydride and lithium aluminum hydride. MeOH, MeCN, CH_2Cl_2, *n*-hexane and toluene were distilled from calcium hydride. (*R*)–sSPhos and (*S*)–sSPhos were prepared by resolution of (*rac*)–sSPhos by preparative SFC, according to our previous publication.[1]

Chiral SFC Analysis: Performed on a Waters ACQUITY UPC2 System with YMC CHIRAL ART SA, SB, SC, or SJ columns (4.6 x 250 mm, 3.0 µm), or DAICEL CHIRAL PAK IG, IE, IJ, IK, IA or IH columns (4.6 x 250 mm, 3.0 µm) in a mixed solvent system of supercritical CO_2 and MeOH or *i*-PrOH. A system backpressure of 138 bar was used in all cases.

Chromatography: Analytical thin-layer chromatography was performed using precoated Merck glass backed silica gel plates (Silica gel 60 F254). Visualization was by ultraviolet fluorescence (λ = 254 and 365 nm) and/or staining with potassium permanganate ($KMnO_4$). Flash column chromatography was performed using silica gel 60 (pore size: 60 Å, mesh: 40-63 µm) from Material Harvest® unless otherwise stated. All ratios of eluents are quoted as v/v.

High Resolution Mass Spectrometry (HRMS): Recorded on a Waters Vion IMS Qtof at the Department of Chemistry at the University of Cambridge. The ionization method is noted—positive/negative electrospray ionization (+/–ESI). Measured values are reported to 4 decimal places and are within ± 5 ppm of the calculated value. The calculated values are based on the most abundant isotope unless otherwise stated in the chemical formula. For ions bearing more than a single unit of charge, the masses reported as 'found' and 'required' are the mass/charge ratios.

NMR Spectroscopy: ^{1}H NMR spectra were recorded on 700 MHz TXO Cryoprobe, 600 MHz Bruker Avance DRX-600, 500 MHz Bruker DCH Cryoprobe, 400 MHz Bruker DPX-400 Dual, 400 MHz Avance III HD, or 400 MHz Avance III HD Smart Probe spectrometers. Chemical shifts are reported in parts per million (ppm) and the spectra are calibrated to the resonance resulting from incomplete deuteration of the solvent ($CDCl_3$: 7.26 ppm; MeOD: 3.31 ppm; ^{13}C NMR spectra were recorded on the same spectrometers with complete proton decoupling. Chemical shifts are reported in ppm with the solvent resonance as the internal standard ($^{13}CDCl_3$: 77.16 ppm, t; $^{13}CD_3OD$: 49.00 ppm. Data are reported as follows: chemical shift δ, multiplicity (s = singlet, d = doublet, t = triplet, q = quartet, p = pentet, sext = sextet, hept = heptet, br = broad, m = multiplet or combinations thereof [^{13}C and all other nuclides except ^{1}H are singlets unless otherwise stated], coupling constants *J*, number of nuclides (signals for all other nuclides except ^{1}H refer to one nuclide unless otherwise stated). ^{1}H NMR spectra are reported in ppm to 2 decimal places and ^{31}C, ^{19}F, and ^{31}P NMR signals are reported to 1 decimal place. Coupling constants are reported in Hz to a maximum of 3 significant figures. For cinchona alkaloid- derived compounds the appearance and chemical shifts of the peaks

in the NMR spectra can vary significantly depending on sample concentration and other factors.

Optical Rotations: Measured in spectrophotometric grade $CHCl_3$, MeOH, or DMSO on a Perkin Elmer 343 Polarimeter using a sodium lamp (λ = 589 nm, D-line). $[\alpha]_D$ values are reported at the stated temperature, with concentration in g /100 mL.

Naming and Numbering of compounds: Systematic names were generated by the computer program ChemDraw according to the guidelines specified by the IUPAC.

Extended Reaction Optimization Data

Investigation of Solvents

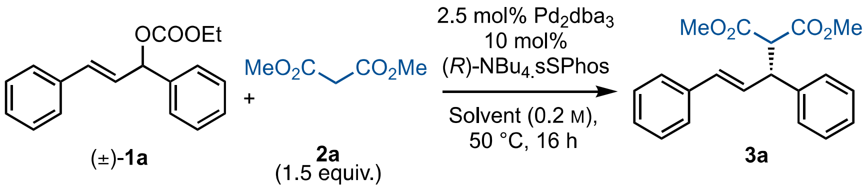

Entry	Solvent	Yield/ %[a]	ee/ %[b]
1	THF	90 (91)	84
2	PhMe	90	84
3	CH_2Cl_2	86	84
4	1,4-Dioxane	92	80
5	MeCN	81	84
6	EtOH	-	N.A.

[a] Yields determined by ^{1}H NMR with reference to a dimethoxyethane internal standard. Value in parentheses refers to isolated material [b]*ee* determined by chiral SFC analysis of the crude reaction mixture, except entry 1.

Investigation of Solvents in Diastereomer-Forming Reaction

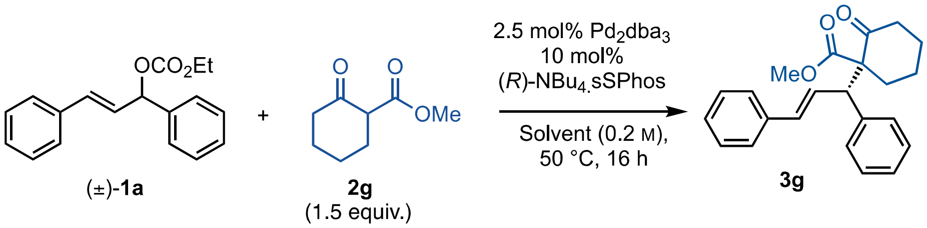

Entry	Solvent	Diast. A/ %[a]	Diast. B/ %[a]	dr	ee (Diast. A)/ %[b]	ee (Diast. B)/ %[b]
1	THF	70 (47)	29 (20)	2.4:1	95	85
2	PhMe	30	14	2.1:1	92	82
3	CH_2Cl_2	57	18	3.2:1	96	82
4	1,4-Dioxane	52	22	2.4:1	92	82
5	MeCN	23	10	2.3:1	92	76
6[c]	THF	16	6	2.7:1	95	70

[a] Yields determined by ^{1}H NMR with reference to a dimethoxyethane internal standard. Values in parentheses refer to isolated material [b]*ee* determined by chiral SFC analysis of the crude reaction mixture, except entry 1.[c] Reaction run at room temperature for 64 h

Synthesis of Allylic Electrophile Starting Materials

General Procedure 1: Esterification of allylic alcohols

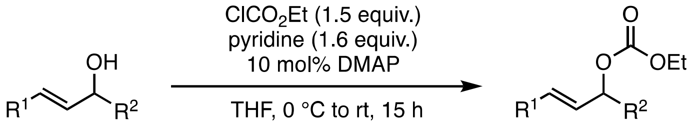

To a solution of ethyl chloroformate (1.5 eq.) in THF (5 mL per mmol of substrate) was added a solution of pyridine (1.6 eq.), DMAP (10 mol%) and allylic alcohol (1.0 eq.) at 0 °C dropwise. The solution was warmed to room temperature slowly and stirred for 15 h. Upon completion, the reaction was quenched with water. The layers were separated, and the aqueous layer extracted further with Et_2O (2×). The combined organic layers were washed sequentially with 3 M HCl (aq.), sat. $NaHCO_3$ (aq.) and H_2O, before being dried over $MgSO_4$, and the solvent removed under reduced pressure. The product was subjected to the next step without further purification.

(±)-(*E*)-1,3-Diphenylprop-2-en-1-ol (S1)

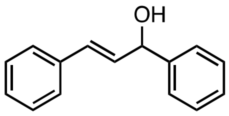

To a solution of phenylmagnesium chloride (25% w/w in diethyl ether, 18.6 mL, 34.0 mmol) in Et_2O (50 mL) was added cinnamyl aldehyde (4.20 g, 4.00 mL, 31.8 mmol) dropwise at 0 °C. The reaction was warmed slowly to room temperature and left to stir for 15 h. The reaction was quenched by the slow addition of sat. NH_4Cl (aq.) (50 mL), followed by filtration through a frit to remove the magnesium salts. The layers were separated, and the aqueous layer was extracted with Et_2O (2 × 100 mL). The combined organic layers were washed with brine (2 × 100 mL), dried over $MgSO_4$, filtered, and the solvent removed under reduced pressure to afford a yellow oil. The crude material was purified by flash column chromatography (5-10% EtOAc in petroleum ether) to afford the title compound as a colorless solid (6.35 g, 30.2 mmol, 95%).

^{1}H NMR (400 MHz, $CDCl_3$) δ 7.48 – 7.19 (m, 10H), 6.70 (d, J = 15.8 Hz, 1H), 6.40 (dd, J = 15.8, 6.5 Hz, 1H), 5.40 (dd, J = 6.7, 3.5 Hz, 1H), 2.02 (d, J = 3.5 Hz, 1H).

^{13}C NMR (101 MHz, $CDCl_3$) δ 142.9, 136.6, 131.6, 130.7, 128.8, 128.7, 128.0, 127.9, 126.8, 126.5, 75.3.

Data in accordance with literature values.[2]

(*E*)-1,3-Diphenylallyl ethyl carbonate (1a)

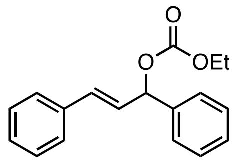

Synthesized according to **GP1** on a 5.71 mmol scale with regards to (*E*)-1,3-diphenylprop-2-en-1-ol (**S1**) to afford the title compound as a colorless oil (1.36 g, 4.82 mmol, 84%).

^{1}H NMR (400 MHz, $CDCl_3$) δ 7.57 – 7.18 (m, 10H), 6.71 (d, J = 15.7 Hz, 1H), 6.40 (dd, J = 15.7, 6.9 Hz, 1H), 6.28 (d, J = 6.9 Hz, 1H), 4.30 – 4.17 (m, 2H), 1.34 (t, J = 7.1 Hz, 3H).

^{13}C NMR (101 MHz, $CDCl_3$) δ 154.4, 138.8, 136.2, 133.1, 128.8, 128.7, 128.5, 128.3, 127.1, 127.1, 126.9, 80.1, 64.3, 14.4.

Data in accordance with literature values.[3]

Cinnamyl ethyl carbonate (1b)

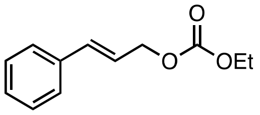

Synthesized according to a modification of **GP1** on a 7.64 mmol scale regarding (*E*)-cinnamyl alcohol. Modification: the crude product was purified by flash chromatography (0-5% EtOAc in petroleum ether) to afford the title compound as a colorless oil (394 mg, 1.91 mmol, 25%).

^{1}H NMR (400 MHz, $CDCl_3$) δ 7.40 (d, J = 7.5 Hz, 2H), 7.33 (t, J = 7.5 Hz, 2H), 7.28 (d, J = 7.5 Hz, 1H), 6.69 (d, J = 15.9 Hz, 1H), 6.39 – 6.21 (m, 1H), 4.78 (d, J = 6.1 Hz, 2H), 4.23 (q, J = 7.1 Hz, 2H), 1.33 (t, J = 7.1 Hz, 3H).

^{13}C NMR (101 MHz, $CDCl_3$) δ 155.2, 136.2, 134.8, 128.7, 128.3, 126.8, 122.7, 68.3, 64.2, 14.4.

Data in accordance with literature values.[4]

(*E*)-4-Phenylbut-3-en-2-ol (S2)

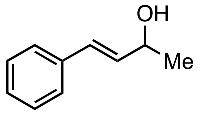

To a solution of cinnamaldehyde (5.00 mL, 36.0 mmol) in Et_2O (100 mL) was added MeLi (1.6 M solution in Et_2O, 45.0 mL, 72.0 mmol) dropwise at -78 °C. The reaction mixture was slowly warmed to room temperature and left to stir for 1 h, when complete consumption of the starting material was observed by TLC. The reaction mixture was cooled to 0 °C, and quenched with sat. NH_4Cl (aq.) (50 mL). The layers were separated, and the aqueous layer extracted with further Et_2O (2 × 50 mL). The combined organic extracts were washed with brine (2 x 50 mL), dried over $MgSO_4$, filtered, and the solvent removed under reduced pressure to afford the crude material as an orange oil. The crude material was purified by flash column chromatography (5:1 hexane/EtOAc) to afford the title compound as a colorless oil (5.19 g, 35.0 mmol, 97%).

^{1}H NMR (400 MHz, $CDCl_3$) δ 7.39 (d, J = 7.4, 2H), 7.32 (t, J = 7.4 Hz, 2H), 7.26 (d, J = 7.4 Hz, 1H), 6.57 (d, J = 15.9 Hz, 1H), 6.29 (dd, J = 15.9, 6.3 Hz, 1H), 4.56 – 4.40 (m, 1H), 2.05 (d, J = 3.9 Hz, 1H), 1.39 (d, J = 6.4 Hz, 3H).

^{13}C NMR (101 MHz, $CDCl_3$) δ 136.8, 128.5, 127.4, 126.4, 68.5, 60.4, 23.3, 14.1.

Data in accordance with literature values.[5]

(±)-(*E*)-Ethyl (4-phenylbut-3-en-2-yl) carbonate (1c)

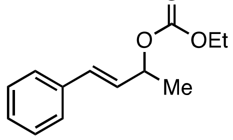

Synthesized according to a modification of **GP1** on a 20.2 mmol scale regarding **S2**. Modification: the crude product was purified by flash chromatography (0-5% v/v EtOAc in petroleum ether) to afford the title compound as a colorless oil (3.22 g, 14.6 mmol, 72%).

^{1}H NMR (400 MHz, $CDCl_3$) δ 7.41 (d, J = 7.3 Hz, 2H), 7.34 (t, J = 7.3 Hz, 2H), 7.28 (d, J = 7.3 Hz, 1H), 6.68 (d, J = 16.0 Hz, 1H), 6.24 (dd, J = 16.0, 7.0 Hz, 1H), 5.41 (m, 1H), 4.23 (q, J = 7.1 Hz, 2H), 1.50 (d, J = 6.5 Hz, 3H), 1.34 (t, J = 7.1 Hz, 3H).

^{13}C NMR (101 MHz, $CDCl_3$) δ 154.5, 136.2, 132.2, 128.6, 128.2, 128.0, 126.7, 75.0, 63.8, 20.5, 14.3.

Data in accordance with literature values.[5]

Synthesis of Nucleophile Starting Materials

Methyl 2-((diphenylmethylene)amino)acetate,[6] methyl 2-oxocyclohexane-1-carboxylate,[7] and methyl 1-oxo-1,2,3,4-tetrahydronaphthalene-2-carboxylate[8] were prepared according to the literature.

Synthesis of Ligands

Sodium (*R*)-2'-(dicyclohexylphosphaneyl)-2,6-dimethoxy-[1,1'-biphenyl]-3-sulfonate ((*R*)–sSPhos)

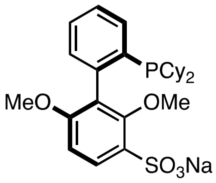

(*rac*)–sSPhos is commercially available. (*R*)–sSPhos and (*S*)–sSPhos were prepared by resolution of (*rac*)–sSPhos by preparative SFC, according to our previous publication.[1]

General Procedure 2: Salt Exchange of (*R*)–sSPhos with Tetraalkylammonium Salts

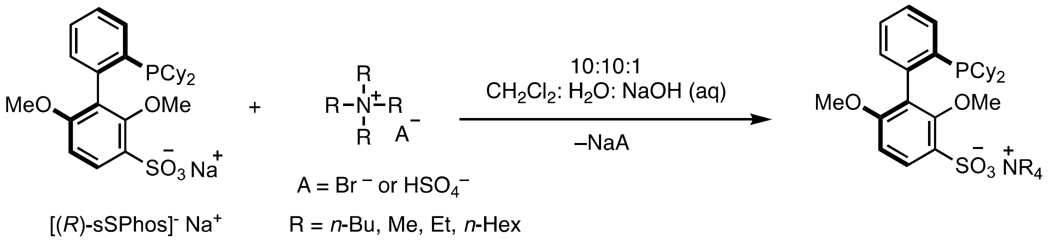

Synthesized according to the salt-exchange procedure reported by Phipps and co-workers.[9] To a biphasic solution of anionic phosphine ligand (1.00 eq.) in CH_2Cl_2 (1 mL per 0.1 mmol of ligand) and water (1 mL per 0.1 mmol of ligand), was added 2.5 M NaOH (aq.) (0.1 mL per 0.1 mmol of ligand) and ammonium salt (1.05 equiv). The biphasic solution was stirred vigorously

for 3 - 16 h and the organic phase was reserved. The aqueous phase was extracted with CH_2Cl_2. The combined organic extracts were washed with portions of water to remove excess ammonium salt (monitored by ^{1}H NMR). The organic layers were dried over $MgSO_4$, filtered, and the solvent removed under reduced pressure to afford the desired product, which was used without further purification.

Tetrabutylammonium (*R*)-2'-(dicyclohexylphosphaneyl)-2,6-dimethoxy-[1,1'-biphenyl]-3-sulfonate [(*R*)-NBu4.sSPhos] (L1)

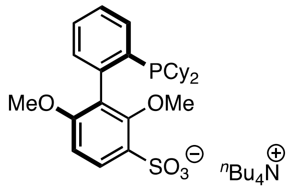

Synthesized according to **GP2** using (*R*)-sSPhos (220 mg, 0.429 mmol) and tetrabutylammonium hydrogen sulfate (150 mg, 0.441 mmol) to afford the title product as an off-white foam (242 mg, 0.331 mmol, 77%).

^{1}H NMR (400 MHz, MeOD) δ 7.90 (d, J = 8.8 Hz, 1H), 7.62 (dd, J = 6.6, 2.0 Hz, 1H), 7.43 – 7.34 (m, 2H), 7.28 – 7.21 (m, 1H), 6.77 (d, J = 8.8 Hz, 1H), 3.71 (s, 3H), 3.41 (s, 3H), 3.30 – 3.22 (m, 8H), 1.87 – 1.09 (m, 38H), 1.04 (t, J = 7.4 Hz, 12H).

^{13}C NMR (101 MHz, MeOD) δ 161.1, 157.3, 143.3 (d, J = 32.6 Hz), 138.0 (d, J = 17.8 Hz), 133.6 (d, J = 3.3 Hz), 133.1 (d, J = 6.2 Hz), 132.1, 129,9, 129.0, 127.8 (d, J = 6.9 Hz), 127.7, 105.6, 79.5, 61.4, 55.9, 36.8 (d, J = 14.8 Hz), 34.8 (d, J = 13.5 Hz), 31.6 (d, J = 10.8 Hz), 31.4 (d, J = 4.4 Hz), 31.0 (d, J = 12.1 Hz), 30.8 (d, J = 13.0 Hz), 28.7 (d, J = 8.2 Hz), 28.6 (d, J = 9.0 Hz), 28.3 (d, J = 9.2 Hz), 28.2 (d, J = 11.5 Hz), 27.7, 27.6, 24.8, 21.3 – 19.7 (m), 13.9.

^{31}P NMR (162 MHz, MeOD) δ -5.4.

HRMS for cation calculated for $[C_{16}H_{36}N]^+$ 242.2842, found 242.2836.

HRMS for anion calculated for $[C_{26}H_{34}O_5PS]^-$ 489.1870, found 489.1872.

$[\alpha]_D^{25.0}$= -17.5° (c. 0.5, MeOH).

Potassium (*R*)-2'-(dicyclohexylphosphaneyl)-2,6-dimethoxy-[1,1'-biphenyl]-3-sulfonate (L2)

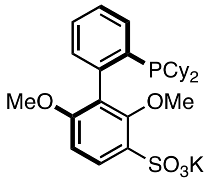

(*R*)-**sSPhos** (51.2 mg, 0.100 mmol) was dissolved in MeOH (20 mL) and passed through a column of Amberlite IRC120 H, hydrogen form (washed first with MeOH until run clear) five times. The solvent removed under reduced pressure to afford the zwitterionic (*R*)-**H⁺.sSPhos**. The zwitterionic (*R*)-**H⁺.sSPhos** was then dissolved in KOH (aq.) (10%, 30 mL), extracted with CH_2Cl_2 (3 × 30 mL), washed with deionized water (2 × 30 mL), and the solvent removed under reduced pressure to afford the title compound as a yellow solid (35.0 mg, 0.0662 mmol, 66%).

^{1}H NMR (400 MHz, MeOD) δ 7.89 (d, J = 8.8 Hz, 1H), 7.60 (m, 1H), 7.40 – 7.35 (m, 2H), 7.23 (m, 1H), 6.77 (d, J = 8.8 Hz, 1H), 3.70 (s, 3H), 3.39 (s, 3H), 1.77 – 1.22 (m, 22H).

^{13}C NMR (101 MHz, MeOD) δ 161.3, 157.1, 143.1 (d, J = 32.3 Hz), 137.8 (d, J = 17.4 Hz), 133.6 (d, J = 3.4 Hz), 133.1 (d, J = 6.1 Hz), 131.7, 129.9, 129.1, 127.9, 127.8 (d, J = 5.8 Hz), 105.8, 61.5, 56.0, 36.8 (d, J = 14.5 Hz), 34.7 (d, J = 13.0 Hz), 31.5 (d, J = 18.3 Hz), 31.3 (d, J = 11.0 Hz), 31.0, 30.8 (d, J = 12.8 Hz), 28.7 (d, J = 6.1 Hz), 28.6 (d, J = 7.2 Hz), 28.3 (d, J = 9.5 Hz), 28.1 (d, J = 11.7 Hz), 27.6 (2 × C).

^{31}P NMR (162 MHz, MeOD) δ -9.2.

HRMS for anion calculated for $[C_{26}H_{34}O_5PS]^-$ 489.1870, found 489.1863.

$[\alpha]_D^{25.0}$= -12.0° (c. 0.25, MeOH).

Cesium (*R*)-2'-(dicyclohexylphosphaneyl)-2,6-dimethoxy-[1,1'-biphenyl]-3-sulfonate (L3)

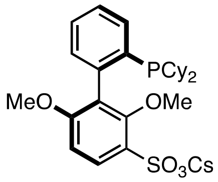

(*R*)-**sSPhos** (51.2 mg, 0.100 mmol) was dissolved in MeOH (20 mL) and passed through a column of Amberlite IRC120 H, hydrogen form (washed first with MeOH until run clear) five times. The solvent was removed under reduced pressure to afford the zwitterionic (*R*)-

H⁺.sSPhos. The zwitterionic (*R*)-**H⁺.sSPhos** was then dissolved in CsOH (aq.) (10%, 30 mL), extracted with CH_2Cl_2 (3 × 30 mL), washed with deionised water (2 × 30 mL), and the solvent removed under reduced pressure to afford the title compound as an off-white solid (33.0 mg, 0.0530 mmol, 53%).

^{1}H NMR (400 MHz, MeOD) δ 7.90 (d, J = 8.8 Hz, 1H), 7.62 (dd, J = 6.1, 2.7 Hz, 1H), 7.39 – 7.36 (m, 2H), 7.28 – 7.13 (m, 1H), 6.78 (d, J = 8.8 Hz, 1H), 3.70 (s, 3H), 3.40 (s, 3H), 1.84 – 1.00 (m, 22H).

^{13}C NMR (101 MHz, MeOD) δ 161.3, 157.1, 143.1 (d, J = 30.8 Hz), 137.8 (d, J = 17.8 Hz), 133.6 (d, J = 3.3 Hz), 133.1 (d, J = 6.0 Hz), 131.6, 129.9, 129.1, 127.9, 127.8, 105.8, 61.5, 56.0, 36.8 (d, J = 14.5 Hz), 34.6 (d, J = 13.0 Hz), 31.5 (d, J = 18.8 Hz), 31.3 (d, J = 11.7 Hz), 31.0 (d, J = 12.2 Hz), 30.8 (d, J = 12.9 Hz), 28.7 (d, J = 5.3 Hz), 28.6 (d, J = 6.4 Hz), 28.3 (d, J = 9.4 Hz), 28.1 (d, J = 11.7 Hz), 27.6 (2 × C).

^{31}P NMR (162 MHz, MeOD) δ -9.2.

HRMS for anion calculated for $[C_{26}H_{34}O_5PS]^-$ 489.1870, found 489.1869.

$[\alpha]_D^{25.0}$= -25.0° (c. 0.24, MeOH).

Tetramethylammonium (*R*)-2'-(dicyclohexylphosphaneyl)-2,6-dimethoxy-[1,1'-biphenyl]-3-sulfonate (L4)

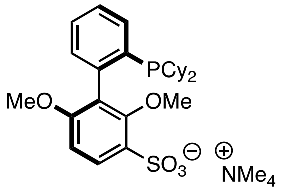

Synthesized according to **GP2** using (*R*)-**sSPhos** (25.0 mg, 0.0488 mmol) and tetramethylammonium bromide (7.7 mg, 0.050 mmol) to afford the title product as a colorless solid (27.5 mg, 0.0488 mmol, quant.)

^{1}H NMR (400 MHz, MeOD) δ 7.91 (d, J = 8.8 Hz, 1H), 7.63 (dq, J = 5.7, 2.2 Hz, 1H), 7.43 – 7.35 (m, 2H), 7.29 – 7.21 (m, 1H), 6.79 (d, J = 8.8 Hz, 1H), 3.72 (s, 3H), 3.41 (s, 3H), 3.22 (s, 12H), 1.85 – 1.09 (m, 22H).

^{13}**C NMR** (101 MHz, MeOD) δ 161.2, 157.1, 143.1 (d, J = 32.3 Hz), 137.8 (d, J = 17.3 Hz), 133.6 (d, J = 3.3 Hz), 133.1 (d, J = 6.3 Hz), 131.9, 129.9, 129.1, 127.8 (d, J = 4.5 Hz), 127.8, 105.7, 61.5, 56.0 (t, J = 1.9 Hz), 55.9, 36.8 (d, J = 14.4 Hz), 34.7 (d, J = 12.9 Hz), 31.5 (d, J = 12.2 Hz), 31.4 (d, J = 5.5 Hz), 31.0 (d, J = 12.1 Hz), 30.8 (d, J = 12.8 Hz), 28.7 (d, J = 7.2 Hz), 28.6 (d, J = 7.9 Hz), 28.3 (d, J = 9.3 Hz), 28.2 (d, J = 11.5 Hz), 27.6, 27.5.

^{31}**P NMR** (162 MHz, MeOD) δ -8.0.

HRMS for cation calculated for $[C_4H_{12}N]^+$ 74.0964, found 74.0960.

HRMS for anion calculated for $[C_{26}H_{34}O_5PS]^-$ 489.1870, found 489.1861.

$[\alpha]_D^{25.0}$= -5.4° (c. 0.24, MeOH).

Tetraethylammonium (*R*)-2'-(dicyclohexylphosphaneyl)-2,6-dimethoxy-[1,1'-biphenyl]-3 sulfonate (L5)

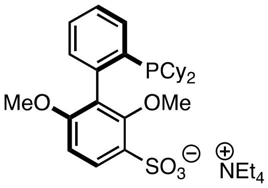

Synthesized according to **GP2** using (*R*)-**sSPhos** (25.0 mg, 0.0488 mmol) and tetraethylammonium hydrogen sulfate (11.4 mg, 0.0502 mmol) to afford the title product as a colorless solid (30.0 mg, 0.0484 mmol, 99%).

^{1}**H NMR** (400 MHz, MeOD) δ 7.89 (d, J = 8.7 Hz, 1H), 7.62 (dt, J = 6.6, 2.5 Hz, 1H), 7.42 – 7.34 (m, 2H), 7.28 – 7.19 (m, 1H), 6.77 (d, J = 8.8 Hz, 1H), 3.70 (s, 3H), 3.40 (s, 3H), 3.33 – 3.26 (m, 8H), 2.06 – 0.98 (m, 34H).

^{13}**C NMR** (101 MHz, MeOD) δ 161.2, 157.1, 143.2 (d, J = 32.4 Hz), 137.8 (d, J = 17.4 Hz), 133.6 (d, J = 3.3 Hz), 133.1 (d, J = 6.2 Hz), 131.9, 129.9, 129.1, 127.8 (d, J = 7.1 Hz), 127.8, 105.7, 61.4, 56.0, 53.4 – 53.0 (m), 36.8 (d, J = 14.4 Hz), 34.7 (d, J = 13.1 Hz), 31.4 (d, J = 29.5 Hz), 31.4, 31.0 (d, J = 12.1 Hz), 30.8 (d, J = 13.1 Hz), 28.7 (d, J = 6.2 Hz), 28.6 (d, J = 7.2 Hz), 28.3 (d, J = 9.3 Hz), 28.2 (d, J = 11.4 Hz), 27.6 (2 x C), 7.6.

^{31}**P NMR** (162 MHz, MeOD) δ -9.3.

HRMS for cation calculated for $[C_8H_{20}N]^+$ 130.1590, found 130.1585.

HRMS for anion calculated for $[C_{26}H_{34}O_5PS]^-$ 489.1870, found 489.1866.

$[\alpha]_D^{25.0}$= -6.2° (c. 0.26, MeOH).

Tetrahexylammonium (*R*)-2'-(dicyclohexylphosphaneyl)-2,6-dimethoxy-[1,1'-biphenyl]-3-sulfonate (L6)

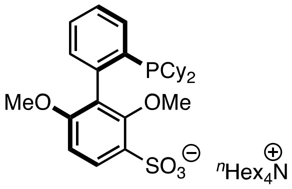

Synthesized according to **GP2** using (*R*)-**sSPhos** (25.0 mg, 0.0488 mmol) and tetrahexylammonium hydrogen sulfate (22.6 mg, 0.0500 mmol) to afford the title product as a colorless solid (38.0 mg, 0.0450 mmol, 92%).

^{1}H NMR (400 MHz, MeOD) δ 7.89 (d, J = 8.8 Hz, 1H), 7.65 – 7.60 (m, 1H), 7.42 – 7.33 (m, 2H), 7.26 – 7.17 (m, 1H), 6.76 (d, J = 8.8 Hz, 1H), 3.69 (s, 3H), 3.40 (s, 3H), 3.24 (dd, J = 8.0, 4.4 Hz, 6H), 1.88 – 1.07 (m, 68H).

^{13}C NMR (101 MHz, MeOD) δ 161.1 (m), 157.2 (m), 143.2 (d, J = 32.5 Hz), 137.9 (d, J = 17.5 Hz), 133.5 (m), 133.1 (m), 132.1, 129.9, 129.0 (m), 127.8 (d, J = 6.8 Hz), 127.7, 105.6, 61.4, 59.6 (m), 55.9, 36.8 (d, J = 14.7 Hz), 34.8 (d, J = 13.2 Hz), 32.3, 31.5 (d, J = 12.1 Hz), 31.4 (d, J = 5.7 Hz), 30.9 (d, J = 12.0 Hz), 30.8 (d, J = 13.0 Hz), 28.7 (d, J = 7.5 Hz), 28.6 (d, J = 8.3 Hz), 28.3 (d, J = 9.3 Hz), 28.2 (d, J = 11.3 Hz), 27.7, 27.6, 27.0 (m), 23.5, 22.7, 14.3.

^{31}P NMR (162 MHz, MeOD) δ -9.4.

HRMS for cation calculated for $[C_{24}H_{52}N]^+$ 354.4100, found 354.4094.

HRMS for anion calculated for $[C_{26}H_{34}O_5PS]^-$ 489.1870, found 489.1863.

$[\alpha]_D^{25.0}$= -18.0° (c. 0.50, MeOH).

(*1S,4S,5R*)-5-Ethyl-2-((*R*)-hydroxy(6-methoxyquinolin-4-yl)methyl)-1-((3,3'',5,5''-tetra-tert-butyl-[1,1':3',1''-terphenyl]-5'-yl)methyl)quinuclidin-1-ium (S3)

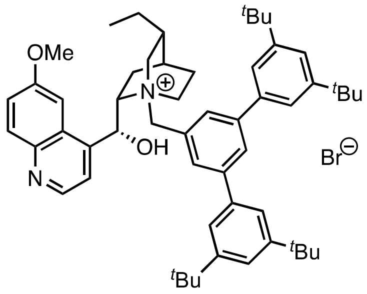

Prepared according to our previous publication.[9]

General Procedure 3: Salt-exchange of anionic phosphine ligands with a chiral cation:

A suspension of sulfonated ligand (1 eq.) and chiral cation (1.05 eq.) in CH_2Cl_2 (2 mL per mmol of ligand) was stirred rapidly for 5 minutes, at which point H_2O (0.2 mL per mmol of ligand) was added dropwise until the solution went cloudy. The suspension was allowed to stir for 3 h at room temperature. The reaction was diluted further with H_2O and CH_2Cl_2. The layers were separated, and the aqueous layer was extracted further with CH_2Cl_2 (2 ×). The combined organic layers were washed thoroughly with H_2O, dried over $MgSO_4$, filtered, and the solvent removed under reduced pressure to afford the desired ion-paired compound, which could be used without further purification.

(*1S,4S,5R*)-5-Ethyl-2-((*R*)-hydroxy(6-methoxyquinolin-4-yl)methyl)-1-((3,3'',5,5''-tetra-tert-butyl-[1,1':3',1''-terphenyl]-5'-yl)methyl)quinuclidin-1-ium (*R*)-2'-(dicyclohexylphosphaneyl)-2,6-dimethoxy-[1,1'-biphenyl]-3-sulfonate (L7)

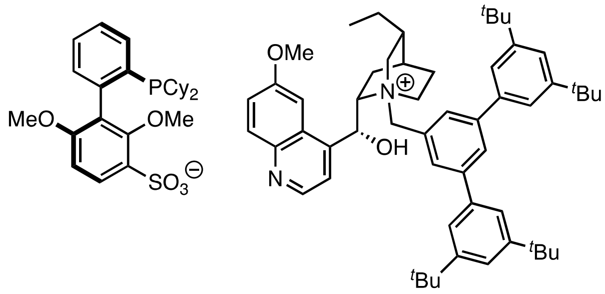

Synthesized according to **GP3** on a 0.113 mmol scale with respect to (*R*)-**sSPhos** and **S3** to afford the title compound as a pale brown solid (144 mg, 0.112 mmol, 99%).

^{1}H NMR ($CDCl_3$, 400 MHz) δ 8.75 (1H, d, *J*=4.5 Hz), 8.04 (1H, d, *J*=9.2 Hz), 7.91 – 7.87 (3H, m), 7.83 (1H, d, *J*=8.8 Hz), 7.76 (1H, d, *J*=4.5 Hz), 7.56 – 7.46 (7H, m), 7.43 (1H, s), 7.38 (1H, dd, *J*=9.2, 2.5 Hz), 7.33 – 7.24 (4H, m), 7.22 – 7.16 (1H, m), 7.10 (1H, d, *J*=6.2 Hz), 6.61 (1H, d,

J=12.4 Hz), 6.27 (1H, dd, *J*=8.9, 2.0 Hz), 5.04 (1H, t, *J*=11.9 Hz), 4.39 (1H, d, *J*=12.3 Hz), 3.97 (3H, s), 3.73 (1H, t, *J*=11.5 Hz), 3.57 (3H, s), 3.53 (1H, d, *J*=8.9 Hz), 3.37 – 3.24 (4H, m), 2.68 – 2.42 (3H, m), 2.04 (4H, d, *J*=16.2 Hz), 1.90 – 0.92 (59H, m), 0.84 (3H, t, *J*=7.3 Hz).

^{13}C NMR ($CDCl_3$, 176 MHz) δ 159.0, 158.4, 155.8, 151.7, 147.8, 144.7 – 144.3 (m), 142.0 (d, *J*=32.5 Hz), 139.7, 137.2 (d, *J*=18.7 Hz), 132.2, 132.1 (d, *J*=6.3 Hz), 131.8, 131.4, 129.4 (d, *J*=7.0 Hz), 127.9, 126.5, 126.1, 125.9 (d, *J*=6.7 Hz), 122.4, 122.2, 121.5, 120.5, 104.0, 101.6, 71.8, 66.1, 63.7, 63.3, 60.8, 56.3, 55.3, 51.4, 36.5, 35.4 (d, *J*=15.7 Hz), 35.2, 33.6 (d, *J*=13.9 Hz), 31.6, 30.9 (d, *J*=14.2 Hz), 30.1 (d, *J*=18.3 Hz), 29.8, 29.7 (d, *J*=12.6 Hz), 29.1 (d, *J*=10.9 Hz), 28.0 (d, *J*=10.4 Hz), 27.8 (d, *J*=9.0 Hz), 27.4 (d, *J*=9.3 Hz), 27.2 (d, *J*=11.2 Hz), 26.7, 26.5, 26.4, 25.5, 24.6, 20.8, 11.4.

^{31}P NMR (162 MHz, $CDCl_3$) δ -9.1.

HRMS [M^+] for cation calculated for $[C_{55}H_{73}N_2O_2]^+$ 793.5667, found 793.5676.

HRMS [M^-] for anion calculated for $[C_{26}H_{34}O_5PS]^-$ 489.1870, found 489.1867.

$[\alpha]_D^{25.0}$= -85.5° (c. 0.26, MeOH).

(*1S,4S,5R*)-5-ethyl-2-((*R*)-hydroxy(6-methoxyquinolin-4-yl)methyl)-1-((3,3'',5,5''-tetra-tert-butyl-[1,1':3',1''-terphenyl]-5'-yl)methyl)quinuclidin-1-ium (*S*)-2'-(dicyclohexylphosphaneyl)-2,6-dimethoxy-[1,1'-biphenyl]-3-sulfonate (L8)

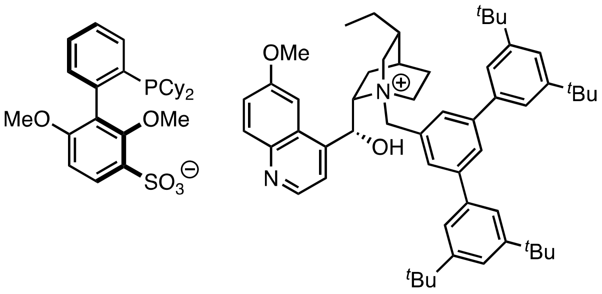

Synthesized according to **GP3** on a 0.113 mmol scale with respect to (*S*)-**sSPhos** and **S3** to afford the title compound as a pale brown solid (140 mg, 0.109 mmol, 96%).

^{1}H NMR ($CDCl_3$, 700 MHz) δ 8.75 (1H, d, *J*=4.5 Hz), 8.04 (1H, d, *J*=9.2 Hz), 7.98 – 7.90 (3H, m), 7.88 (1H, s), 7.79 (1H, d, *J*=4.5 Hz), 7.55 – 7.49 (6H, m), 7.43 – 7.40 (1H, m), 7.38 (1H, dd, *J*=9.2, 2.6 Hz), 7.30 – 7.19 (6H, m), 7.11 (1H, d, *J*=7.8 Hz), 6.81 (1H, d, *J*=12.4 Hz), 6.42 (1H, d, *J*=8.8 Hz), 5.06 (1H, t, *J*=12.1 Hz), 4.28 (1H, d, *J*=12.3 Hz), 3.94 (3H, s), 3.76 (1H, dd, *J*=12.6, 10.6 Hz),

3.60 (3H, s), 3.46 (1H, dd, *J*=10.4, 5.8 Hz), 3.29 (1H, q, *J*=10.8, 10.0 Hz), 3.15 (3H, s), 2.55 (3H, dd, *J*=19.6, 12.6 Hz), 2.06 (1H, d, *J*=4.8 Hz), 1.89 (1H, s), 1.83 (1H, p, *J*=7.8 Hz), 1.40 (60H, s), 0.84 (3H, t, *J*=7.4 Hz).

^{13}C NMR ($CDCl_3$, 176 MHz) δ 159.0, 158.4, 155.8, 151.7, 147.8, 145.0, 144.5, 144.4, 142.1 (d, *J*=32.8 Hz), 139.8, 132.3, 132.3 (d, *J*=6.4 Hz), 132.1, 131.9, 131.5, 129.6, 129.5, 127.9, 127.9 – 127.8 (m), 126.5, 126.0, 125.9 – 125.7 (m), 122.4, 122.2, 121.5, 120.6, 104.2, 101.5, 72.3, 66.0, 63.5, 63.2, 60.9, 56.3, 55.3, 51.1, 36.4, 35.2, 31.7, 30.2 (d, *J*=16.8 Hz), 29.8, 29.7 (d, *J*=12.0 Hz), 29.2 (d, *J*=11.1 Hz), 27.9 (d, *J*=9.9 Hz), 27.8 (d, *J*=9.3 Hz), 27.5 (d, *J*=9.6 Hz), 27.3 (d, *J*=11.4 Hz), 26.6 (d, *J*=10.4 Hz), 26.5, 25.4, 24.7, 20.6, 11.4.

^{31}P NMR ($CDCl_3$, 203 MHz) δ -9.2.

HRMS [M^+] for cation calculated for $[C_{55}H_{73}N_2O_2]^+$ 793.5667, found 793.5672.

HRMS [M^-] for anion calculated for $[C_{26}H_{34}O_5PS]^-$ 489.1870, found 489.1859.

$[\alpha]_D^{25.0}$= -85.3° (c. 0.26, MeOH).

(*R*)-Neopentyl 2'-(dicyclohexylphosphaneyl)-2,6-dimethoxy-[1,1'-biphenyl]-3-sulfonate [(*R*)-sSPhos-Np]

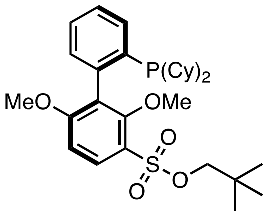

Prepared by Dr. David Whalley according to our previous publication, for which we are very grateful.[10]

Synthesis of Products

General Procedure 4: Asymmetric Allylic Alkylation Reactions

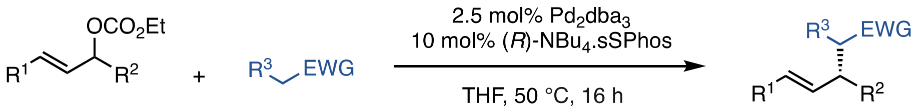

A 4 mL vial containing a magnetic stirrer bar was charged with allylic substrate (0.20 mmol), nucleophile (0.30 mmol), $Pd_2(dba)_3$ (4.6 mg, 2.5 mol%) and (*R*)-NBu_4.sSPhos (14.6 mg, 10

mol%). The vial was sealed and placed under an atmosphere of nitrogen via three evacuation/refill cycles. Anhydrous THF (1.0 mL) was added via syringe, and the reaction mixture was heated in a preheated aluminum heating block to 50 °C for 16 h. The reaction mixture was concentrated under a stream of nitrogen, and purified via flash column chromatography.

N.B. Reactions that afforded products 3e, 3f, 3g, 3i, 3j were run on a 0.30 mmol scale to aid in isolation and characterization. Loadings were scaled accordingly.

Dimethyl (*R,E*)-2-(1,3-diphenylallyl)malonate (3a)

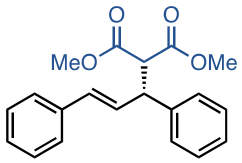

Prepared according to **GP4**. The crude product was purified by flash column chromatography (0-6% EtOAc in petroleum ether) to afford the title compound as a colorless oil (58.8 mg, 0.181 mmol, 91%, 84% ee).

^{1}H NMR (700 MHz, $CDCl_3$) δ 7.34 – 7.18 (m, 10H), 6.48 (d, J = 15.7 Hz, 1H), 6.34 (dd, J = 15.7, 8.6 Hz, 1H), 4.27 (dd, J = 10.8, 8.6 Hz, 1H), 3.96 (d, J = 10.8 Hz, 1H), 3.71 (s, 3H), 3.52 (s, 3H).

^{13}C NMR (176 MHz, $CDCl_3$) δ 168.3, 168.0, 140.4, 137.0, 132.0, 129.3, 128.9, 128.6, 128.0, 127.7, 127.3, 126.5, 57.8, 52.7, 52.6, 49.3.

$[\boldsymbol{\alpha}]_{\boldsymbol{D}}^{\mathbf{25.0}}$= +15.0° (c. 1.0, $CHCl_3$). Literature value: $[\boldsymbol{\alpha}]_{\boldsymbol{D}}^{\mathbf{25.0}}$ = +12.4° (c 1.0, $CHCl_3$) for > 99% ee, *R* enantiomer.[11]

Chiral SFC Analysis CHIRAL ART SJ (CO_2:*i*-PrOH, 99:01, 2.5 mL min^{-1}, 40 °C) t_R = 6.48 (minor), 6.81 (major) minutes.

Data in accordance with literature values.[2]

<u>**Control Experiment Scheme 2D**</u>

Prepared according to **GP4**, with (*R*)-sSPhos-Np (11.2 mg, 2.5 mol%) in place of (*R*)-sSPhos as the ligand. The crude product was purified by flash column chromatography (0-6% EtOAc in

petroleum ether) to afford the title compound as a colorless oil (49.9 mg, 0.154 mmol, 77%, 90% ee).

Chiral SFC Analysis CHIRAL PAK IA (CO_2:MeOH, 99:01, 2.5 mL min^{-1}, 40 °C) t_R = 9.23 (major), 10.11 (minor) minutes.

$[\alpha]_D^{25.0}$ = +15.2° (c. 0.60, $CHCl_3$).

1 mmol Scale Reaction

A 20 mL vial containing a magnetic stirrer bar was charged with (*E*)-1,3-diphenylallyl ethyl carbonate (**1a**) (282 mg, 1.00 mmol, 1.00 equiv.), dimethyl malonate (198 mg, 1.50 mmol, 1.50 equiv.), $Pd_2(dba)_3$ (22.9 mg, 0.0250 mmol, 2.50 mol%) and (*R*)-NBu_4.sSPhos (73.2 mg, 0.100 mmol, 10.0 mol%). The vial was sealed and placed under an atmosphere of nitrogen via three evacuation/refill cycles. Anhydrous THF (5.0 mL) was added via syringe, and the reaction mixture was heated in a preheated aluminum heating block to 50 °C for 16 h. The reaction mixture was concentrated under a stream of nitrogen, and purified via flash column chromatography (0-6% EtOAc in petroleum ether), to afford the title compound as a colorless oil (162 mg, 0.50 mmol, 50% yield, 87% ee).

Chiral SFC Analysis CHIRAL PAK IA (CO_2:MeOH, 99:01, 2.5 mL min^{-1}, 40 °C) t_R = 8.19 (major), 9.08 (minor) minutes.

Dimethyl (*S,E*)-2-(1,3-diphenylallyl)-2-methylmalonate (3b)

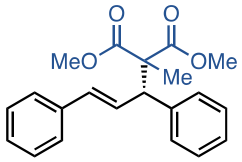

Prepared according to **GP4**. The crude product was purified by flash column chromatography (0-4% EtOAc in petroleum ether) to afford the title compound as a yellow oil. N.B. The product was isolated with a 7% inseparable dibenzylideneacetone impurity. (67.4 mg, 0.190 mmol adjusting for impurity, 95%, 90% ee).

^{1}H NMR (400 MHz, $CDCl_3$) δ 7.42 – 7.22 (m, 10H), 6.72 (dd, J = 15.6, 8.9 Hz, 1H), 6.51 (d, J = 15.6 Hz, 1H), 4.34 (d, J = 8.9 Hz, 1H), 3.74 (s, 3H), 3.66 (s, 3H), 1.52 (s, 3H).

^{13}**C NMR** (101 MHz, $CDCl_3$) δ 171.6, 171.4, 139.4, 137.4, 132.9, 129.6, 128.6, 128.6, 128.4, 127.5, 127.3, 126.5, 59.3, 54.0, 52.6, 52.6, 18.8.

Chiral SFC Analysis CHIRAL ART SJ (CO_2:*i*-PrOH, 99:01, 2.5 mL min^{-1}, 40 °C) t_R = 6.54 (major), 8.16 (minor) minutes.

$[\alpha]_D^{25.0}$= -29.1° (c. 1.0, $CHCl_3$).

Data in accordance with literature values.[2]

(*R,E*)-3-(1,3-diphenylallyl)Pentane-2,4-dione (3c)

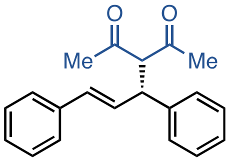

Prepared according to **GP4**. The crude product was purified by flash column chromatography (0-6% EtOAc in petroleum ether) to afford the title compound as a colorless solid (53.9 mg, 0.184 mmol, 92%, 78% ee).

^{1}**H NMR** (700 MHz, $CDCl_3$) δ 7.36 – 7.21 (m, 10H), 6.45 (d, J = 15.8 Hz, 1H), 6.22 (dd, J = 15.8, 4.9 Hz, 1H), 4.47 – 4.29 (m, 2H), 2.28 (s, 3H), 1.95 (s, 3H).

^{13}**C NMR** (176 MHz, $CDCl_3$) δ 202.9, 202.8, 140.3, 136.7, 131.8, 129.4, 129.2, 128.7, 128.1, 127.8, 127.4, 126.5, 74.7, 49.3, 30.1, 29.8.

Chiral SFC Analysis CHIRAL ART SJ (CO_2:*i*-PrOH, 99:01, 2.5 mL min^{-1}, 40 °C) t_R = 7.30 (minor), 7.81 (major) minutes.

$[\alpha]_D^{25.0}$= -4.4° (c. 1.0, $CHCl_3$).

Data in accordance with literature values.[12]

(*S,E*)-(4-methyl-4-nitropent-1-ene-1,3-diyl)Dibenzene (3d)

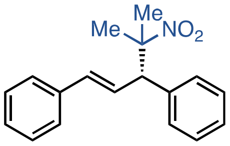

Prepared according to **GP4**. The crude product was purified by flash column chromatography (0-2% EtOAc in petroleum ether) to afford the title compound as a yellow oil (47.8 mg, 0.170 mmol, 85%, 88% ee).

^{1}H NMR (500 MHz, $CDCl_3$) δ 7.39 – 7.23 (m, 10H), 6.62 – 6.44 (m, 2H), 4.14 (d, J = 7.9 Hz, 1H), 1.66 (s, 3H), 1.56 (s, 3H).

^{13}C NMR (126 MHz, $CDCl_3$) δ 138.2, 136.7, 134.7, 129.2, 128.7, 128.7, 128.0, 127.9, 126.6, 125.6, 92.0, 58.0, 24.5, 23.5.

Chiral SFC Analysis CHIRAL ART SJ (CO_2:*i*-PrOH, 98:02, 2.5 mL min^{-1}, 40 °C) t_R = 9.14 (major), 11.44 (minor) minutes.

$[\alpha]_D^{25.0}$ = -52.4° (c. 1.0, $CHCl_3$).

Data in accordance with literature values.[13]

((*S,E*)-4-Nitropent-1-ene-1,3-diyl)dibenzene (3e)

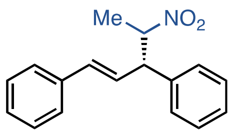

Prepared according to **GP4** on a 0.3 mmol scale. The two diastereomers were separated via flash column chromatography (0-2% EtOAc in petroleum ether).

Diastereomer A **(3ea)**

Afforded as a colorless oil (44.9 mg, 0.168 mmol, 56%, 92% ee).

^{1}H NMR (400 MHz, $CDCl_3$) δ 7.46 – 7.19 (m, 10H), 6.50 (d, J = 15.6 Hz, 1H), 6.37 (dd, J = 15.6, 8.8 Hz, 1H), 5.03 – 4.90 (m, 1H), 3.91 (dd, J = 9.5, 8.8 Hz, 1H), 1.43 (d, J = 6.2 Hz, 3H).

^{13}C NMR (101 MHz, $CDCl_3$) δ 138.5, 136.5, 133.3, 129.4, 128.6, 128.2, 128.0, 127.9, 127.4, 126.7, 87.9, 54.5, 18.1.

HRMS $[M+H]^+$ calculated for $[C_{17}H_{18}NO_2]^+$ 268.1332, found 268.1326.

Chiral SFC Analysis CHIRAL ART SJ (CO_2:*i*-PrOH, 99:01, 2.5 mL min^{-1}, 40 °C) t_R = 11.23 (minor), 11.57 (major) minutes.

$[\alpha]_D^{25.0}$=+26.1° (c. 1.0, $CHCl_3$).

Diastereomer B **(3eb)**

Afforded as a colorless oil (26.4 mg, 98.8 µmol, 33%, 91% ee).

^{1}H NMR (400 MHz, $CDCl_3$) δ 7.41 – 7.25 (m, 10H), 6.61 (d, J = 15.7 Hz, 1H), 6.28 (dd, J = 15.7, 9.4 Hz, 1H), 5.06 – 4.88 (m, 1H), 4.05 (dd, J = 9.4, 9.0 Hz, 1H), 1.69 (d, J = 6.6 Hz, 3H).

^{13}C NMR (101 MHz, $CDCl_3$) δ 139.1, 136.5, 134.2, 129.1, 128.8, 128.2, 127.8, 127.7, 126.6, 126.3, 87.2, 54.5, 18.0.

HRMS $[M+H]^+$ calculated for $[C_{17}H_{18}NO_2]^+$ 268.1332, found 268.1326.

Chiral SFC Analysis CHIRAL ART SJ (CO_2:MeOH, 98:02, 2.5 mL min^{-1}, 40 °C) t_R = 10.31 (major), 11.86 (minor) minutes.

$[\alpha]_D^{25.0}$= -25.8° (c. 0.5, $CHCl_3$).

Methyl (*S,E*)-2-((diphenylmethylene)amino)-3,5-diphenylpent-4-enoate (3f)

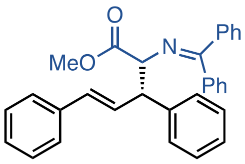

Prepared according to **GP4** on a 0.3 mmol scale. The two diastereomers were separated by flash column chromatography (0-5% EtOAc in petroleum ether).

Diastereomer A **(3fa)**

Afforded as a colorless oil (49.4 mg, 111 µmol, 37%, 92% ee).

^{1}H NMR (400 MHz, $CDCl_3$) δ 7.86 – 7.76 (1H, m), 7.66 – 7.55 (2H, m), 7.49 (1H, t, *J*=7.6 Hz), 7.43 – 7.12 (16H, m), 6.69 – 6.50 (2H, m), 4.48 (1H, d, *J*=6.0 Hz), 4.30 (1H, dd, *J*=8.3, 6.0 Hz), 3.61 (3H, s).

^{13}C NMR (101 MHz, $CDCl_3$) δ 196.9, 171.5, 141.2, 139.6, 137.7, 137.5, 136.4, 132.6, 132.5, 130.5, 130.2, 129.2, 129.0, 128.7, 128.6, 128.6, 128.5, 128.4, 128.4, 128.2, 127.9, 127.4, 126.8, 126.5, 71.1, 53.2, 52.2.

HRMS $[M+H]^+$ calculated for $[C_{31}H_{28}NO_2]^+$ 446.2115, found 446.2116.

Chiral SFC Analysis CHIRAL PAK IG (CO_2:MeOH, 95:05, 2.5 mL min^{-1}, 40 °C) t_R = 8.95 (major), 9.88 (minor) minutes.

$[\alpha]_D^{25.0}$= +46.2° (c. 1.0, $CHCl_3$).

Diastereomer B **(3fb)**

Afforded as a colorless oil (47.6 mg, 107 µmol, 36%, 91% ee).

^{1}H NMR (400 MHz, $CDCl_3$) δ 7.85 – 7.79 (1H, m), 7.53 – 7.12 (17H, m), 6.80 – 6.70 (2H, m), 6.51 (1H, d, *J*=15.7 Hz), 6.34 (1H, dd, *J*=15.7, 8.9 Hz), 4.49 (1H, d, *J*=8.9 Hz), 4.37 – 4.21 (1H, m), 3.69 (3H, s).

^{13}C NMR (101 MHz, $CDCl_3$) δ 196.9, 171.7, 140.5, 139.8, 137.7, 137.3, 136.1, 132.5, 132.1, 130.4, 130.2, 129.1, 129.0, 129.0, 128.8, 128.5, 128.4, 128.4, 128.3, 128.2, 128.0, 127.5, 126.8, 126.4, 71.6, 53.7, 52.2.

HRMS $[M+H]^+$ calculated for $[C_{31}H_{28}NO_2]^+$ 446.2115, found 446.2110.

Chiral SFC Analysis CHIRAL PAK IG (CO_2:MeOH, 95:05, 2.5 mL min^{-1}, 40 °C) t_R = 11.23 (minor), 13.82 (major) minutes.

$[\alpha]_D^{25.0}$= -101° (c. 1.0, $CHCl_3$).]

Methyl (*S*,*E*)-1-(1,3-diphenylallyl)-2-oxocyclohexane-1-carboxylate (3g)

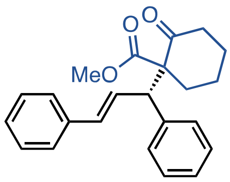

Prepared according to **GP4** on a 0.3 mmol scale. The two diastereomers were separated by flash column chromatography (0-3% EtOAc in petroleum ether).

Diastereomer A **(3ga)**

Afforded as a colorless solid. N.B. The product was isolated with a 15% inseparable dibenzylideneacetone impurity (54.4 mg, 0.142 mmol adjusting for impurity, 47%, 95% ee).

^{1}H NMR (400 MHz, $CDCl_3$) δ 7.46 – 7.15 (10H, m), 6.71 (1H, dd, *J*=15.7, 9.5 Hz), 6.41 (1H, d, *J*=15.7 Hz), 4.12 (1H, d, *J*=9.5 Hz), 3.54 (3H, s), 2.52 – 2.35 (3H, m), 2.01 – 1.88 (1H, m), 1.81 – 1.52 (4H, m).

^{13}C NMR (101 MHz, $CDCl_3$) δ 206.7, 171.4, 140.0, 137.4, 132.6, 130.2, 129.1, 129.0, 128.6, 128.2, 127.5, 127.0, 126.5, 66.2, 53.7, 42.2, 35.0, 27.1, 22.8.

HRMS $[M+H]^+$ calculated for $[C_{23}H_{25}O_3]^+$ 349.1798, found 349.1788.

Chiral SFC Analysis CHIRAL PAK IG (CO_2:MeOH, 95:05, 2.5 mL min^{-1}, 40 °C) t_R = 8.26 (minor), 8.59 (major) minutes.

$[\alpha]_D^{25.0}$= +33.3° (c. 1.0, $CHCl_3$).

Diastereomer B **(3gb)**

Afforded as a colorless solid (20.9 mg, 60.0 µmol, 20%, 85% ee).

^{1}H NMR (400 MHz, $CDCl_3$) δ 7.34 – 7.17 (m, 10H), 6.66 (dd, J = 15.8, 8.7 Hz, 1H), 6.41 (d, J = 15.8 Hz, 1H), 4.26 (d, J = 8.7 Hz, 1H), 3.51 (s, 3H), 2.64 – 2.58 (m, 1H), 2.51 – 2.33 (m, 2H), 1.95 (ddd, J = 12.3, 5.8, 3.0 Hz, 1H), 1.80 – 1.56 (m, 4H).

^{13}C NMR (101 MHz, $CDCl_3$) δ 206.4, 171.3, 139.9, 139.4, 137.5, 132.6, 129.9, 129.3, 128.6, 128.3, 127.4, 127.1, 126.5, 66.3, 53.1, 52.3, 42.1, 33.6, 26.8.

HRMS $[M+H]^+$ calculated for $[C_{23}H_{25}O_3]^+$ 349.1798, found 349.1797.

Chiral SFC Analysis CHIRAL PAK IG (CO_2:MeOH, 95:05, 2.5 mL min^{-1}, 40 °C) t_R = 9.92 (major), 12.16 (minor) minutes.

$[\alpha]_D^{25.0}$= -38.6° (c. 0.5, $CHCl_3$).

Methyl (*S*,*E*)-2-(1,3-diphenylallyl)-1-oxo-1,2,3,4-tetrahydronaphthalene-2-carboxylate (3h)

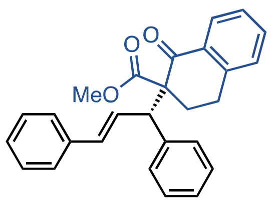

Prepared according to **GP4**. The two diastereomers were purified by flash column chromatography (0-2% EtOAc in petroleum ether) and preparatory TLC (10% EtOAc in PhMe) to afford the two pure diastereomers.

Diastereomer A **(3ha)**

Colorless solid (11.9 mg, 30.0 µmol, 15%, 72% ee).

^{1}H NMR ($CDCl_3$, 500 MHz) δ 8.05 (1H, dd, *J*=7.9, 1.4 Hz), 7.49 – 7.45 (2H, m), 7.42 (1H, td, *J*=7.4, 1.4 Hz), 7.38 – 7.34 (2H, m), 7.31 – 7.24 (5H, m), 7.23 – 7.13 (3H, m), 6.71 (1H, dd, *J*=15.5, 10.1 Hz), 6.53 (1H, d, *J*=15.5 Hz), 4.63 (1H, d, *J*=10.1 Hz), 3.51 (3H, s), 3.25 (1H, ddd, *J*=17.4, 12.2, 4.7 Hz), 2.90 (1H, ddd, *J*=17.4, 4.9, 2.8 Hz), 2.68 (1H, ddd, *J*=13.6, 4.7, 2.8 Hz), 2.14 (1H, ddd, *J*=13.6, 12.2, 4.9 Hz).

^{13}C NMR (126 MHz, $CDCl_3$) δ 193.3, 170.1, 143.1, 140.0, 137.3, 133.7, 133.0, 132.6, 130.2, 128.8, 128.6, 128.5, 128.2, 128.1, 127.6, 127.0, 126.7, 126.6, 62.9, 53.1, 52.7, 29.1, 26.4.

HRMS $[M+Na]^+$ calculated for $[C_{27}H_{24}NaO_3]^+$ 419.1618, found 419.1618.

Chiral SFC Analysis CHIRAL PAK IE (CO_2:MeOH, 90:10, 2.5 mL min^{-1}, 40 °C) t_R = 10.29 (minor), 11.72 (major) minutes.

$[\alpha]^{25.0}$= -0.8° (c. 0.5, $CHCl_3$).

Diastereomer B **(3hb)**

Colorless solid (29.8 mg, 75.2 µmol, 38%, 90% ee).

^{1}H NMR ($CDCl_3$, 500 MHz) δ 8.11 (1H, d, *J*=8.1 Hz), 7.46 – 7.34 (5H, m), 7.33 – 7.13 (8H, m), 6.99 (1H, dd, *J*=15.7, 9.1 Hz), 6.49 (1H, d, *J*=15.7 Hz), 4.38 (1H, d, *J*=9.1 Hz), 3.53 (3H, s), 3.10 (1H, ddd, *J*=17.3, 12.6, 4.4 Hz), 2.93 – 2.81 (1H, m), 2.72 – 2.58 (1H, m), 2.23 (1H, td, *J*=13.2, 4.8 Hz).

^{13}C NMR (126 MHz, $CDCl_3$) δ 193.8, 171.0, 143.0, 139.5, 137.5, 133.6, 132.9, 132.5, 130.0, 129.6, 128.7, 128.5, 128.5, 128.3, 127.4, 127.3, 126.8, 126.5, 63.6, 55.5, 52.6, 30.2, 26.7.

HRMS $[M+Na]^+$ calculated for $[C_{27}H_{24}NaO_3]^+$ 419.1618, found 419.1621.

Chiral SFC Analysis CHIRAL PAK IE (CO_2:MeOH, 90:10, 2.5 mL min^{-1}, 40 °C) t_R = 10.23 (minor), 11.67 (major) minutes.

$[\alpha]_D^{25.0}$= +0.8° (c. 0.5, $CHCl_3$).

Ethyl (*S*,*E*)-2-ethyl-2-nitro-3,5-diphenylpent-4-enoate (3i)

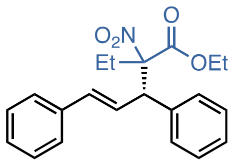

Prepared according to **GP4** on a 0.3 mmol scale. The two diastereomers were separated via flash column chromatography (0-4% Et_2O in petroleum ether).

Diastereomer A **(3ia)**

Afforded as a colorless solid (68.6 mg, 194 µmol, 65%, 95% ee).

^{1}H NMR (400 MHz, $CDCl_3$) δ 7.43 – 7.18 (m, 10H), 6.70 (dd, J = 15.7, 8.5 Hz, 1H), 6.46 (d, J = 15.7 Hz, 1H), 4.40 (d, J = 8.5 Hz, 1H), 4.35 – 4.17 (m, 2H), 2.25 – 1.98 (m, 2H), 1.26 (t, J = 7.1 Hz, 3H), 0.97 (t, J = 7.4 Hz, 3H).

^{13}C NMR (101 MHz, $CDCl_3$) δ 166.4, 137.0, 136.9, 133.8, 129.1, 128.9, 128.6, 128.2, 127.8, 127.0, 126.6, 99.8, 62.8, 54.3, 29.0, 14.1, 8.9.

HRMS $[M+Na]^+$ calculated for $[C_{21}H_{23}NNaO_4]^+$ 376.1519, found 376.1512.

Chiral SFC Analysis CHIRAL PAK IG (CO_2:MeOH, 95:05, 2.5 mL min^{-1}, 40 °C) t_R = 4.26 (minor), 4.76 (major) minutes.

$[\alpha]_D^{25.0}$= -57.4° (c. 1.0, $CHCl_3$).

Diastereomer B **(3ib)**

Afforded as a colorless oil (30.8 mg, 87.2 µmol, 29%, 83% ee).

^{1}H NMR (400 MHz, $CDCl_3$) δ 7.40 – 7.17 (m, 10H), 6.66 (dd, J = 15.7, 8.7 Hz, 1H), 6.44 (d, J = 15.7 Hz, 1H), 4.49 (d, J = 8.7 Hz, 1H), 4.40 – 4.19 (m, 2H), 2.32 – 1.98 (m, 2H), 1.30 (t, J = 7.1 Hz, 3H), 0.94 (t, J = 7.4 Hz, 3H).

^{13}C NMR (101 MHz, $CDCl_3$) δ 165.8, 137.3, 136.9, 133.8, 129.3, 128.9, 128.6, 128.1, 127.8, 126.6 (2xC), 99.9, 62.6, 54.4, 29.6, 14.0, 8.7.

HRMS $[M+Na]^+$ calculated for $[C_{21}H_{23}NNaO_4]^+$ 376.1519, found 376.1507.

Chiral SFC Analysis CHIRAL PAK IJ (CO_2:MeOH, 99:01, 2.5 mL min^{-1}, 40 °C) t_R = 8.89 (major), 9.68 (minor) minutes.

$[\alpha]_D^{25.0}$= -52.0° (c. 1.0, $CHCl_3$).

Ethyl (*S*,*E*)-2-nitro-3,5-diphenylpent-4-enoate (3ja + 3jb)

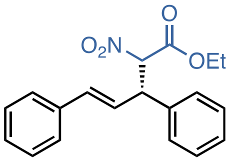

Prepared according to **GP4** on a 0.3 mmol scale. The crude product was purified by flash column chromatography (0-3% EtOAc in petroleum ether) to afford the title compound as a 52:48 ratio of diastereomers by ^{1}H NMR (66.7 mg, 205 µmol, 68%, 89% ee and 90% ee).

^{1}H NMR (400 MHz, $CDCl_3$) δ 7.40 – 7.21 (m, $10H_{maj}$ and $10H_{min}$), 6.57 (app d, J = 15.7 Hz, $1H_{maj}$ and $1H_{min}$), 6.40 (dd, J = 15.7, 8.6 Hz, $1H_{min}$), 6.27 (dd, J = 15.7, 9.0 Hz, $1H_{maj}$), 5.56 (d, J = 7.7 Hz, $1H_{min}$), 5.54 (d, J = 7.3 Hz, $1H_{maj}$), 4.61 – 4.46 (m, $1H_{maj}$ and $1H_{min}$), 4.25 (q, J = 7.1 Hz, $2H_{maj}$), 4.12 – 4.00 (m, $2H_{min}$), 1.23 (t, J = 7.1 Hz, $3H_{maj}$), 1.04 (t, J = 7.1 Hz, $3H_{min}$).

^{13}C NMR (101 MHz, $CDCl_3$) δ 163.4, 163.0, 137.6, 136.8, 136.3, 136.2, 134.2, 134.1, 129.3, 129.2, 128.7, 128.7, 128.4, 128.3, 128.2, 128.2, 128.1, 127.8, 126.7, 126.6, 125.6, 125.1, 91.9, 91.4, 63.2, 63.0, 50.7, 50.5, 14.1, 13.7.

HRMS $[M+Na]^+$ calculated for $[C_{19}H_{19}NNaO_4]^+$ 348.1206, found 348.1198.

Chiral SFC Analysis CHIRAL PAK IG (CO_2:MeOH, 95:05, 2.5 mL min^{-1}, 40 °C) *Diastereomer A* t_R = 6.06 (minor), 6.39 (major) minutes. *Diastereomer B* t_R = 5.48 (minor), 6.87 (major) minutes.

$[\alpha]_D^{25.0}$= +13.2° (c. 2.0, $CHCl_3$).

(*E*)-2-(1,3-diphenylallyl)-1,3-Diphenylpropane-1,3-dione (3k)

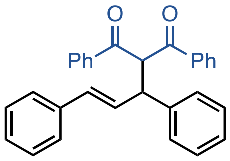

Prepared according to a modification of **GP4**:

A 4 mL vial containing a magnetic stirrer bar was loaded with allylic substrate (**1a**) (0.200 mmol), nucleophile (0.300 mmol), $Pd_2(dba)_3$ (2.50 mol%) and (*R*)-NBu_4.**sSPhos** (10.0 mol%). The vial was sealed and placed under an atmosphere of nitrogen via three evacuation/refill cycles. Anhydrous toluene (1.0 mL) was added via syringe, and the reaction mixture was heated to 120 °C in a preheated aluminum heating block for 16 h. Upon completion, the reaction mixture was concentrated under a stream of nitrogen. The crude material was purified by flash column chromatography (0-4% EtOAc in petroleum ether) to afford the title compound as a colorless solid (50. 8 mg, 122 µmol, 61%, 60% ee).

^{1}H NMR (700 MHz, $CDCl_3$) δ 8.04 (2H, d, *J*=7.4 Hz), 7.82 (2H, d, *J*=7.4 Hz), 7.55 (1H, t, *J*=7.4 Hz), 7.50 – 7.41 (3H, m), 7.36 – 7.31 (4H, m), 7.22 (2H, t, *J*=7.7 Hz), 7.17 (2H, t, *J*=7.4 Hz), 7.15 – 7.10 (2H, m), 7.08 (2H, d, *J*=7.2 Hz), 6.36 – 6.26 (2H, m), 5.96 (1H, d, *J*=10.5 Hz), 4.80 (1H, dd, *J*=10.5, 7.6 Hz).

^{13}C NMR (176 MHz, $CDCl_3$) δ 194.5, 193.9, 141.0, 137.4, 137.0, 137.0, 133.6, 133.4, 132.0, 130.0, 128.9, 128.9, 128.8, 128.7, 128.7, 128.4 (2 × C), 127.5, 127.0, 126.4, 62.8, 50.1.

Chiral SFC Analysis CHIRAL ART SC (CO_2:MeOH, 90:10, 2.5 mL min^{-1}, 40 °C) t_R = 8.71 (major), 11.31 (minor) minutes.

$[\alpha]_D^{25.0}$= +26.0° (c. 0.25, $CHCl_3$).

Data in accordance with literature values.[14]

(*E*)-(4,4-bis(phenylsulfonyl)but-1-ene-1,3-diyl)Dibenzene (3l)

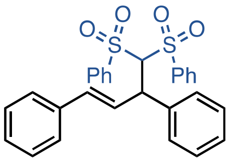

Prepared according to **GP4**. The crude product was purified by flash column chromatography (0-2% EtOAc in petroleum ether) to afford the title compound as a colorless solid (66.6 mg, 136 µmol, 68%, 27% ee).

^{1}H NMR (400 MHz, $CDCl_3$) δ 8.06 (2H, d, *J*=7.8 Hz), 7.68 (2H, d, *J*=7.9 Hz), 7.60 (2H, t, *J*=8.1 Hz), 7.51 (2H, t, *J*=7.8 Hz), 7.43 (2H, t, *J*=7.7 Hz), 7.39 – 7.22 (10H, m), 6.92 (1H, dd, *J*=15.7, 9.5 Hz), 6.24 (1H, d, *J*=15.7 Hz), 5.13 (1H, s), 4.74 (1H, d, *J*=9.5 Hz).

^{13}C NMR (101 MHz, $CDCl_3$) δ 140.8, 140.8, 138.0, 136.7, 135.1, 135.0, 134.7, 134.1, 130.4, 129.5, 129.1, 129.0, 128.8, 128.8, 128.6, 128.3, 127.9, 127.4, 126.8, 124.4, 89.3, 47.8.

Chiral SFC Analysis CHIRAL PAK IG (CO_2:*i*-PrOH, 75:25, 2.5 mL min^{-1}, 40 °C) t_R = 10.06 (major), 12.70 (minor) minutes.

Data in accordance with literature values.[15]

(*E*)-3-(1,3-diphenylallyl)-1*H*-Indole (3m)

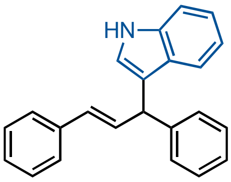

Prepared according to **GP4**. The crude product was purified by flash column chromatography (0-5% EtOAc in petroleum ether) to afford the title compound as an off-white solid (20.7 mg, 66.9 µmol, 33%, 19% ee).

^{1}H NMR ($CDCl_3$, 500 MHz) δ 7.97 (1H, s), 7.46 (1H, d, *J*=8.0 Hz), 7.42 – 7.16 (12H, m), 7.05 (1H, t, *J*=7.5 Hz), 6.92 (1H, s), 6.75 (1H, dd, *J*=15.8, 7.3 Hz), 6.47 (1H, d, *J*=15.8 Hz), 5.15 (1H, d, *J*=7.3 Hz).

^{13}C NMR (126 MHz, $CDCl_3$) δ 143.5, 137.6, 136.8, 132.7, 130.7, 128.6, 128.6, 128.6, 127.3, 126.9, 126.5, 126.5, 122.7, 122.2, 120.0, 119.6, 118.8, 111.2, 46.3.

Chiral SFC Analysis CHIRAL PAK IK (CO_2:MeOH, 85:15, 2.5 mL min^{-1}, 40 °C) t_R = 8.65 (minor), 8.95 (major) minutes.

Data in accordance with literature values.[16]

(*E*)-(3-phenoxyprop-1-ene-1,3-diyl)Dibenzene (3o)

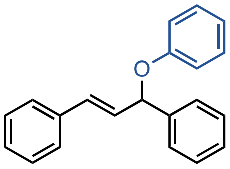

Prepared according to **GP4**. The crude material was purified by flash column chromatography (20-40% CH_2Cl_2 in petroleum ether) to afford the title compound as a colourless oil (50.8 mg, 177 µmol, 89%, rac.).

^{1}H NMR ($CDCl_3$, 400 MHz) δ 7.51 – 7.44 (2H, m), 7.38 (4H, td, *J*=6.7, 6.1, 1.7 Hz), 7.34 – 7.28 (4H, m), 7.24 – 7.20 (2H, m), 6.99 (2H, d, *J*=8.1 Hz), 6.93 (1H, t, *J*=7.3 Hz), 6.68 (1H, d, *J*=15.9 Hz), 6.45 (1H, dd, *J*=15.9, 6.3 Hz), 5.81 (1H, d, *J*=6.3 Hz).

^{13}C NMR ($CDCl_3$, 101 MHz) δ 158.1, 140.5, 136.5, 131.7, 129.5, 129.5, 128.8, 128.7, 128.0, 128.0, 126.8, 126.8, 121.2, 116.4, 80.9.

Data in accordance with literature values.[17]

Methyl 1-cinnamyl-2-oxocyclohexane-1-carboxylate (3p)

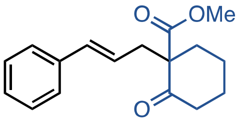

Prepared according to **GP4**. The crude product was purified by flash column chromatography (0-5% EtOAc in petroleum ether) to afford the title compound as a colorless oil (40.2 mg, 148 µmol, 74%, 8% ee).

^{1}H NMR (400 MHz, $CDCl_3$) δ 7.34 – 7.22 (m, 4H), 7.21 – 7.14 (m, 1H), 6.38 (d, J = 15.8 Hz, 1H), 6.23 – 6.09 (m, 1H), 3.70 (s, 3H), 2.75 (dd, J = 14.5, 7.6 Hz, 1H), 2.59 – 2.43 (m, 4H), 2.07 – 1.98 (m, 1H), 1.81 – 1.56 (m, 4H).

^{13}C NMR (101 MHz, $CDCl_3$) δ 207.6, 172.2, 137.4, 133.4, 128.6, 127.4, 126.3, 125.2, 61.7, 52.5, 41.3, 38.7, 36.1, 27.6, 22.7.

Chiral SFC Analysis CHIRAL ART SJ (CO_2:*i*-PrOH, 99:01, 2.5 mL min^{-1}, 40 °C) t_R = 6.13 (minor), 7.37 (major) minutes.

Data in accordance with literature values.[18]

Dimethyl (*E*)-2-(4-phenylbut-3-en-2-yl)malonate (3q)

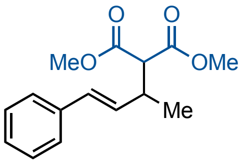

Prepared according to **GP4**. The crude material was purified by flash column chromatography (0-5% EtOAc in petroleum ether) to afford the title compound as a yellow oil (49.1 mg, 187 µmol, 94%, 6% ee).

^{1}H NMR (400 MHz, $CDCl_3$) δ 7.36 – 7.25 (4H, m), 7.24 – 7.17 (1H, m), 6.46 (1H, d, *J*=15.8 Hz), 6.13 (1H, dd, *J*=15.8, 8.5 Hz), 3.75 (3H, s), 3.67 (3H, s), 3.40 (1H, d, *J*=8.9 Hz), 3.20 – 3.06 (1H, m), 1.19 (3H, d, *J*=6.8 Hz).

^{13}C NMR (101 MHz, $CDCl_3$) δ 168.8, 168.7, 137.2, 131.3, 130.9, 128.6, 127.5, 126.4, 57.9, 52.56, 52.5, 37.9, 18.6.

Chiral SFC Analysis CHIRAL PAK IG (CO_2:MeOH, 95:05, 2.5 mL min^{-1}, 40 °C) t_R = 4.38 (major), 5.35 (minor) minutes.

Data in accordance with literature values.[19]

References

(1) Pearce-Higgins, R.; Hogenhout, L. N.; Docherty, P. J.; Whalley, D. M.; Chuentragool, P.; Lee, N.; Lam, N. Y. S.; McGuire, T. M.; Valette, D.; Phipps, R. J. *J. Am. Chem. Soc.* **2022**, *144*, 15026–15032.

(2) Carroll, A.-M.; McCarthy, M.; Lacey, P. M.; Saunders, C. P.; Connolly, D. J.; Farrell, A.; Rokade, B. V.; Goddard, R.; Fristrup, P.; Norrby, P.-O.; Guiry, P. J. *Tetrahedron*, **2020**, *76*, 130780.

(3) Nemoto, T.; Jin, L.; Nakamura, H.; Hamada, Y.; *Tetrahedron Lett.*, **2006**, *47*, 6577–6581.

(4) Horn, A.; Kazmaier, U. *Org. Lett.*, **2019**, *21*, 4595–4599.

(5) Faller, J. W.; Wilt, J. C. *Organometallics*, **2005**, *24*, 5076–5083.

(6) Pintér Á.; Haberhauer, G. *Eur. J. Org. Chem.*, **2008**, *2008*, 2375–2387.

(7) Yang, D;. Li, J.-H.; Gao, Q.; Yan, Y.-L. *Org. Lett.*, **2003**, *5*, 2869–2871.

(8) Ko, T. Y.; Youn, S. W. *Adv. Synth. Catal.*, **2016**, *358*, 1934–1941.

(9) Genov, G. R.; Douthwaite, J. L.; Lahdenperä, A. S. K.; Gibson, D. C.; Phipps, R. J. *Science,* **2020**, *367*, 1246–1251.

(10) Kadarauch, M.; Whalley, D, M.; Phipps, R. J. *J. Am. Chem. Soc.* **2023**, *145*, 25553–25558.

(11) Pálvölgyi, Á. M.; Schnürch, M.; Bica-Schröder, K.; *Tetrahedron*, **2020**, *76*, 131246.

(12) Qiu, Z.; Sun, R.; Teng, D. *Org. Biomol. Chem.*, **2018**, *16*, 7717–7724.

(13) Seebach, D.; Devaquet, E.; Ernst, A.; Hayakawa, M.; Kühnle, F. N. M.; Bernd Schweizer, W.; Weber, B.; *Helv. Chim. Acta*, **1995**, *78*, 1636–1650.

(14) Cheng, D.; Bao, W. *Adv. Synth. Catal.*, 2008, **350**, 1263–1266.

(15) Allen, J. V.; Coote, S. J.; Dawson, G. J.; Frost, C. G.; Martins, C. J.; Williams, J. M. J. *J. Chem. Soc., Trans.* **1994**, 2065–2072.

(16) Paul, D;. Chatterjee, P. N. *European J. Org. Chem.*, **2020**, *2020*, 4705–4712.

(17) Dai, L.; Li, X.; Yuan, H.; Li, X.; Li, Z.; Xu, D.; Fei, F.; Liu, Y.; Zhang J.; Zhou, Z. *Tetrahedron Asymmetry*, **2011**, *22*, 1379–1389.

(18) Nemoto, T.; Matsumoto, T.; Masuda, T.; Hitomi, T.; Hatano, K.; Hamada, Y. *J. Am. Chem. Soc.*, **2004**, *126*, 3690–3691.

(19) Tsuji, H.; Takahashi Y.; Kawatsura, M. *Tetrahedron Lett.*, **2021**, *68*, 152916.

^{1}H NMR (400 MHz, $CDCl_3$): (±)-(*E*)-1,3-Diphenylprop-2-en-1-ol (S1)

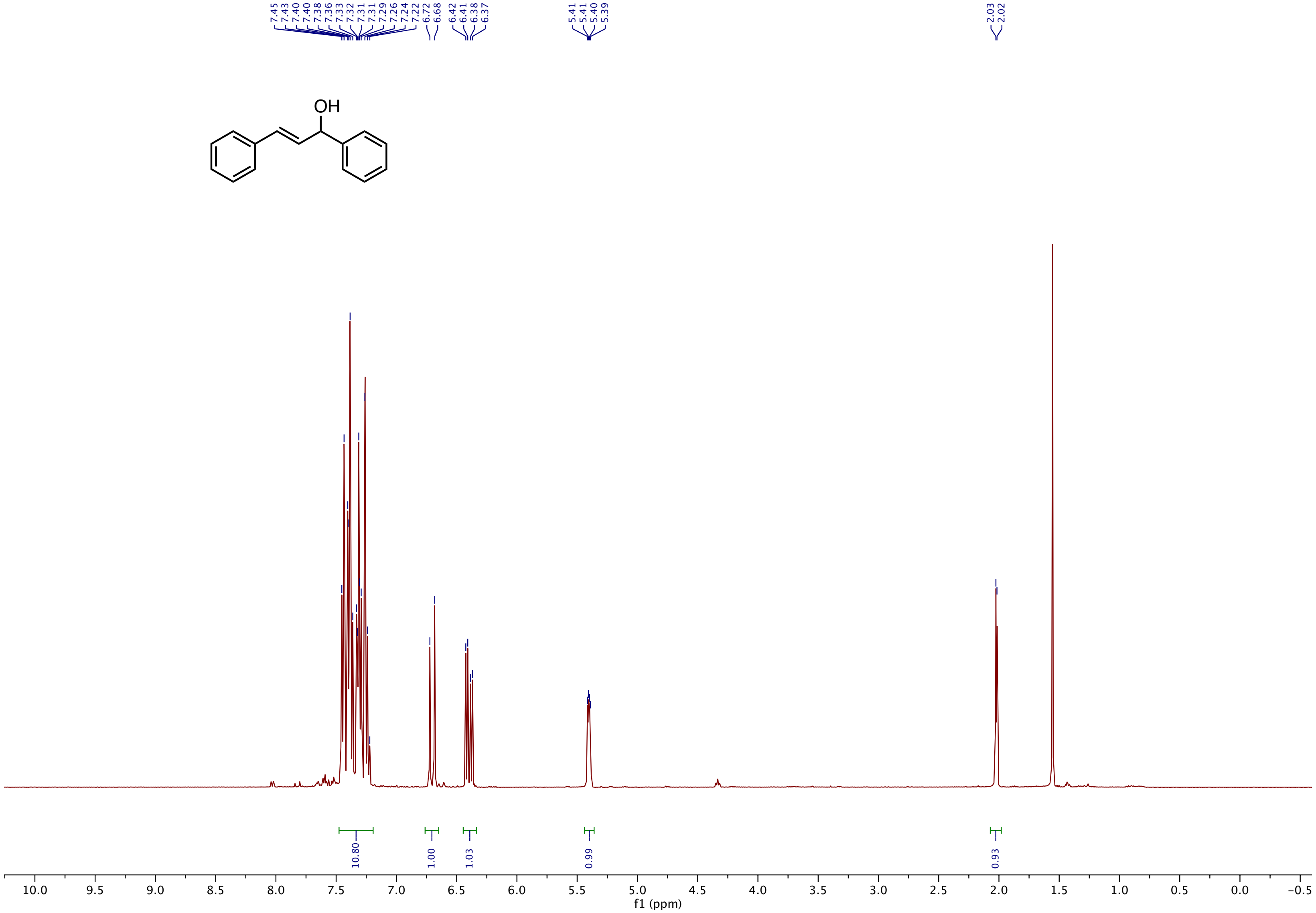

^{13}C NMR (101 MHz, $CDCl_3$): (±)-(*E*)-1,3-Diphenylprop-2-en-1-ol (S1)

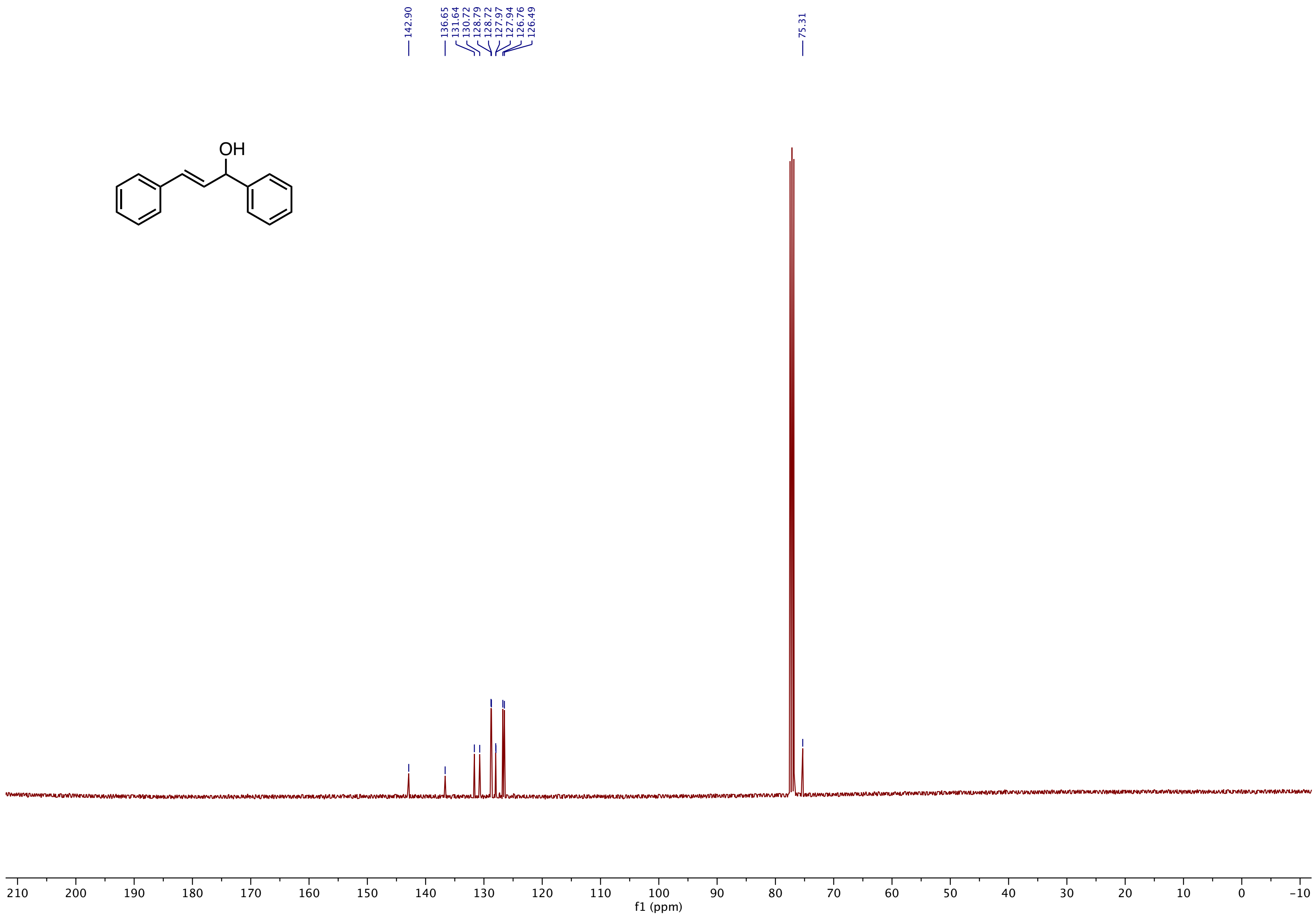

^{1}H NMR (400 MHz, $CDCl_3$): (±)-(*E*)-1,3-Diphenylallyl ethyl carbonate (1a)

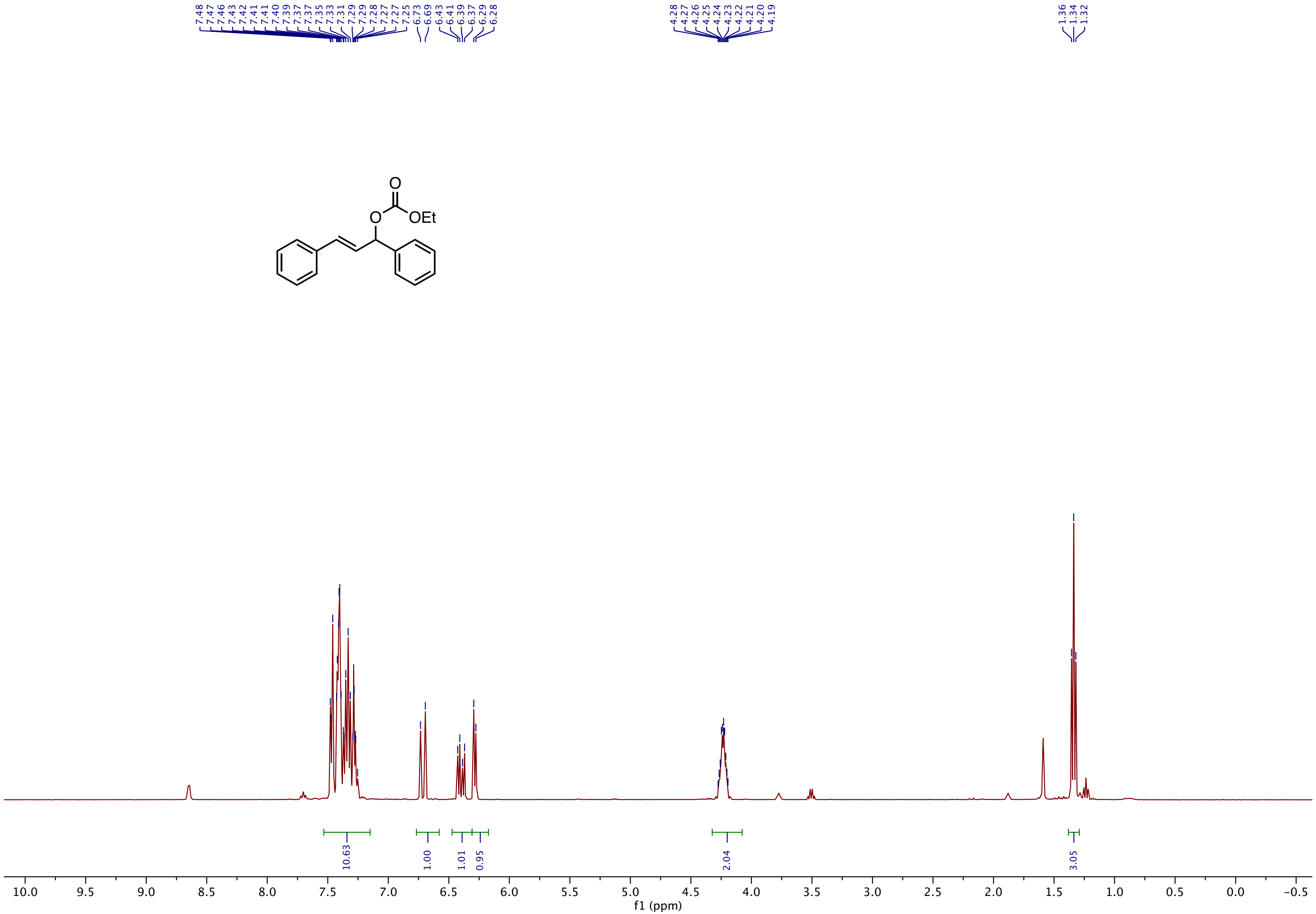

^{13}C NMR (101 MHz, $CDCl_3$): (±)-(*E*)-1,3-Diphenylallyl ethyl carbonate (1a)

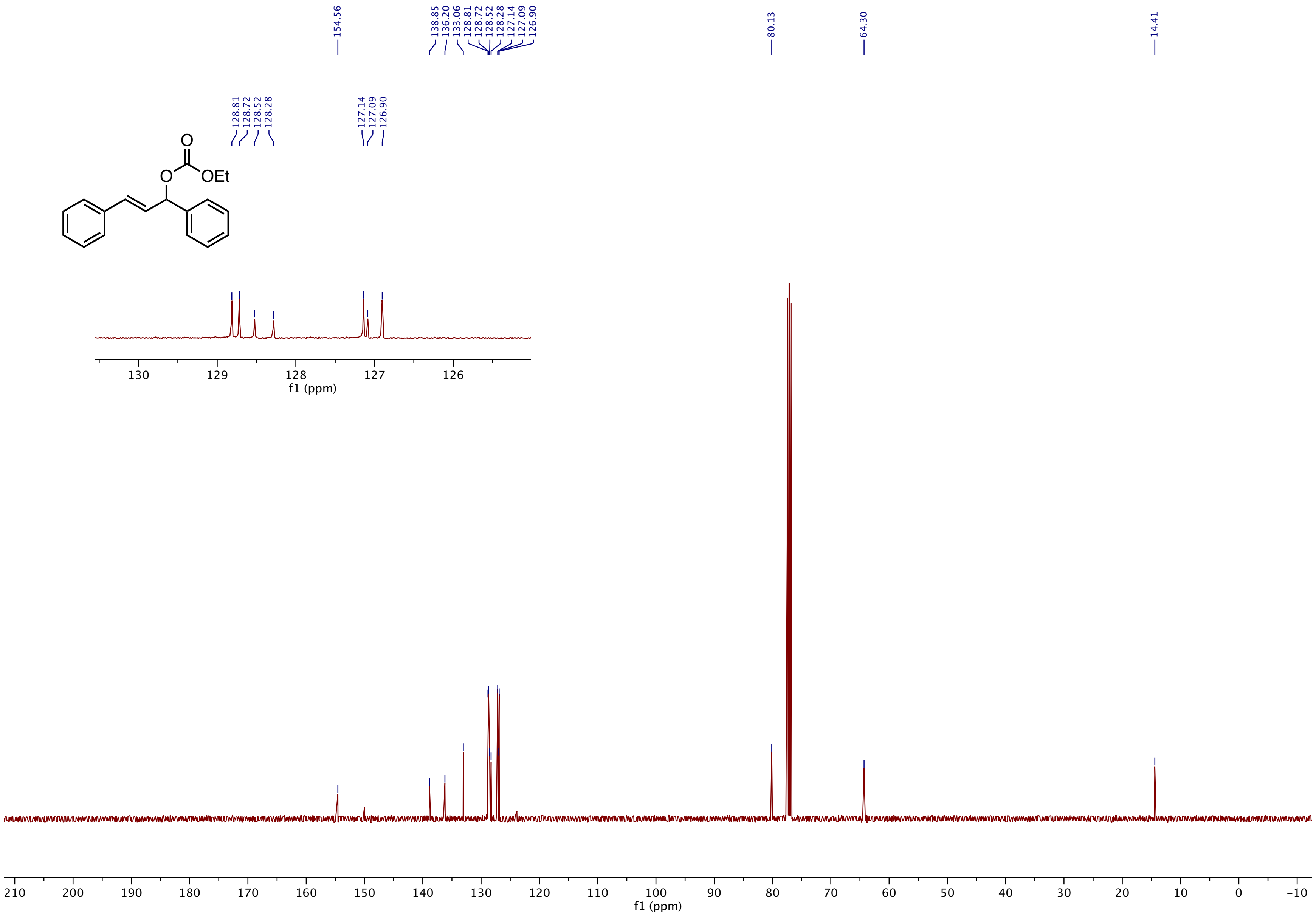

^{1}H NMR (400 MHz, $CDCl_3$): Cinnamyl ethyl carbonate (1b)

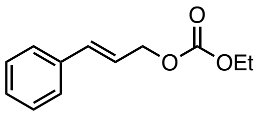

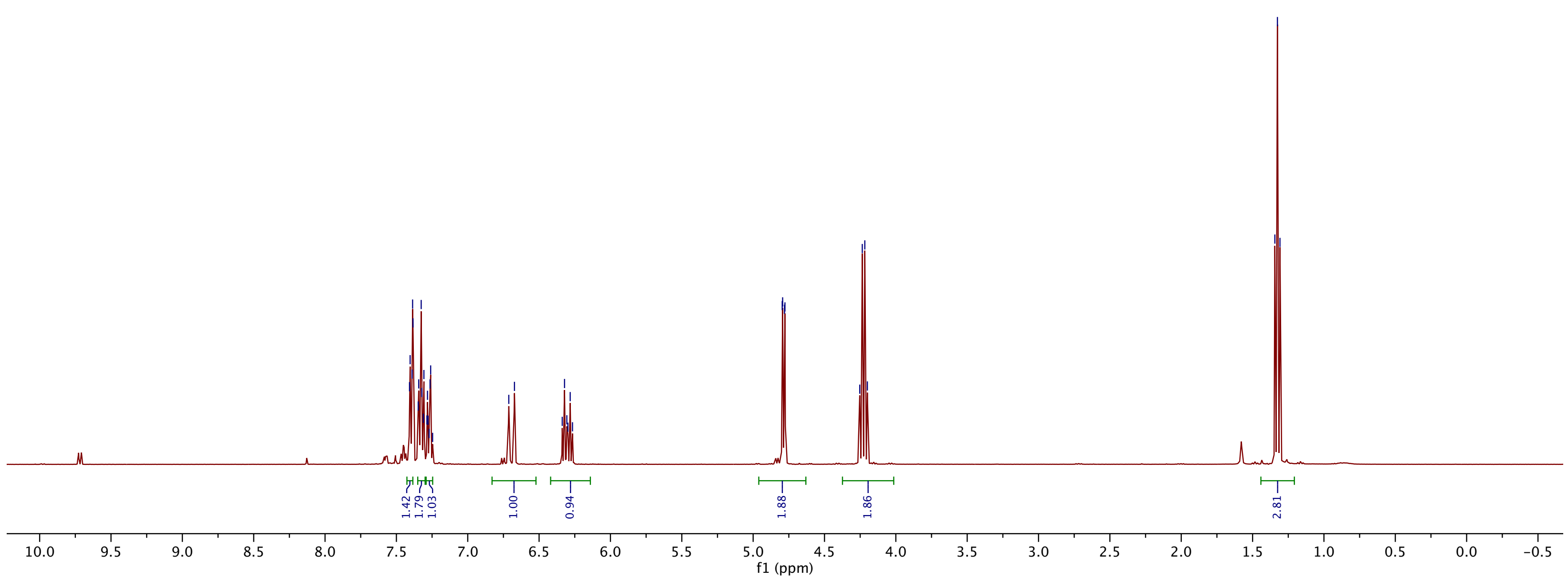

^{13}C NMR (101 MHz, $CDCl_3$): Cinnamyl ethyl carbonate (1b)

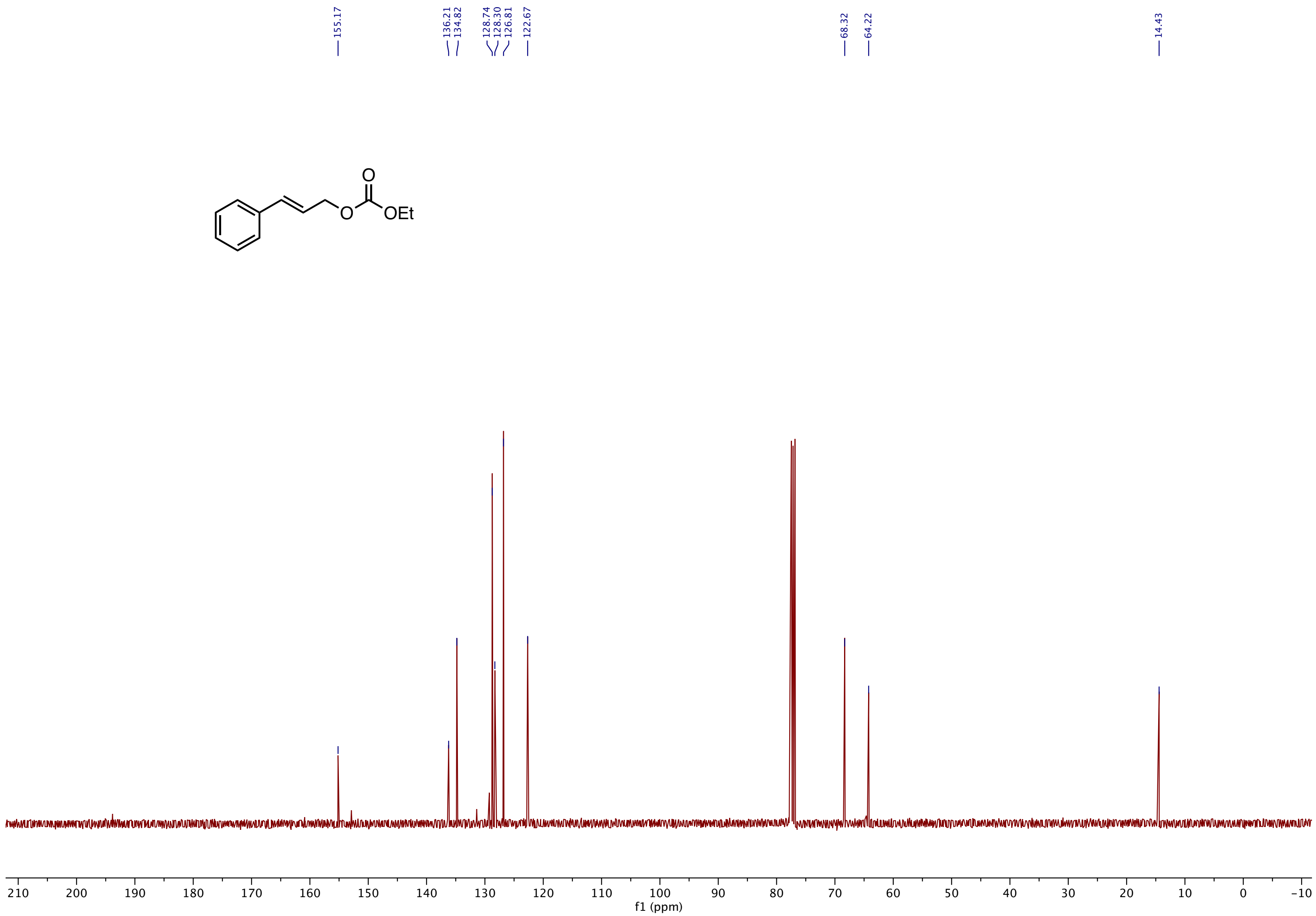

^{1}H NMR (400 MHz, $CDCl_3$): (±)-(*E*)-4-Phenylbut-3-en-2-ol (*S*2)

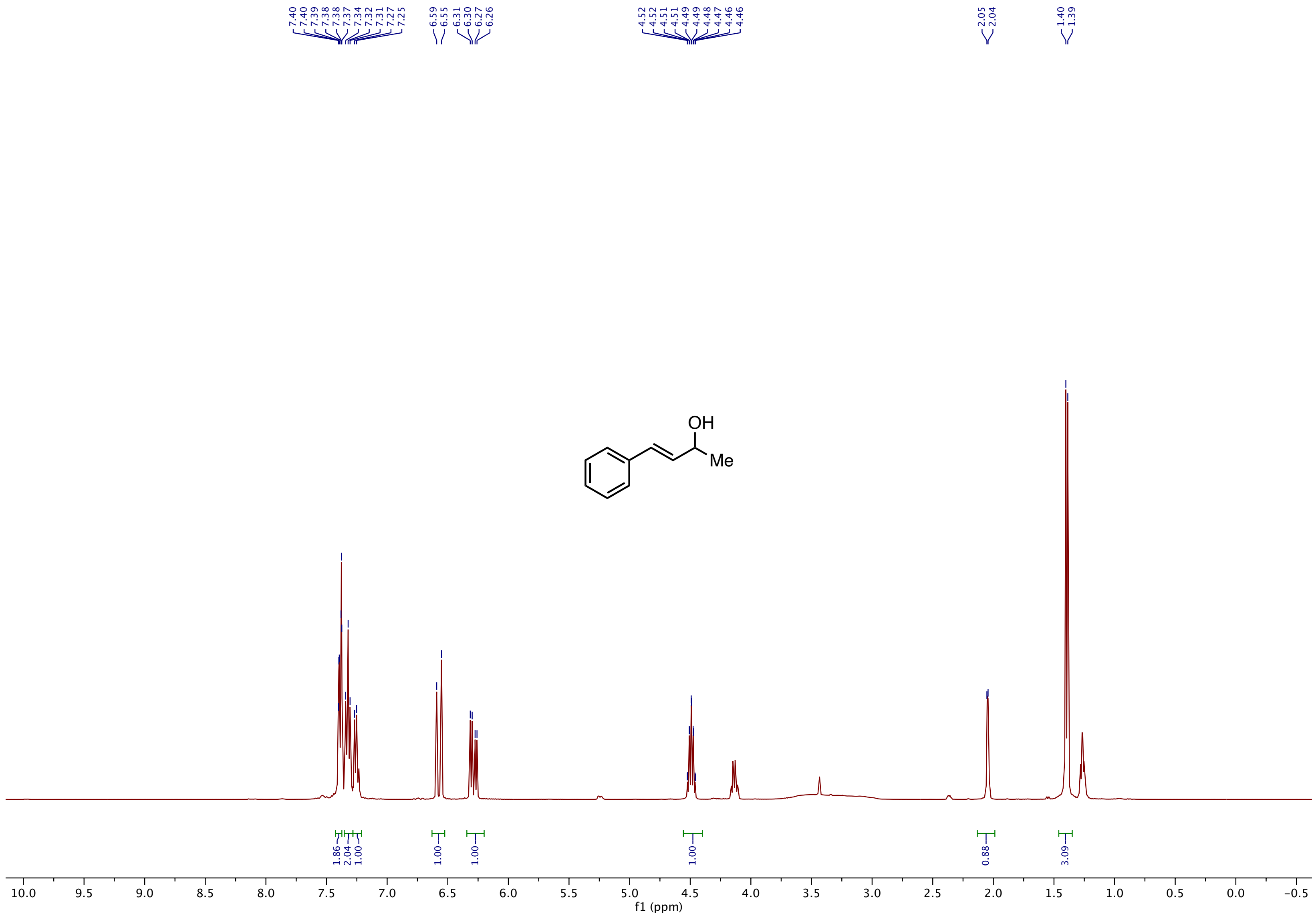

^{13}C NMR (101 MHz, $CDCl_3$): (±)-(*E*)-4-Phenylbut-3-en-2-ol (S2)

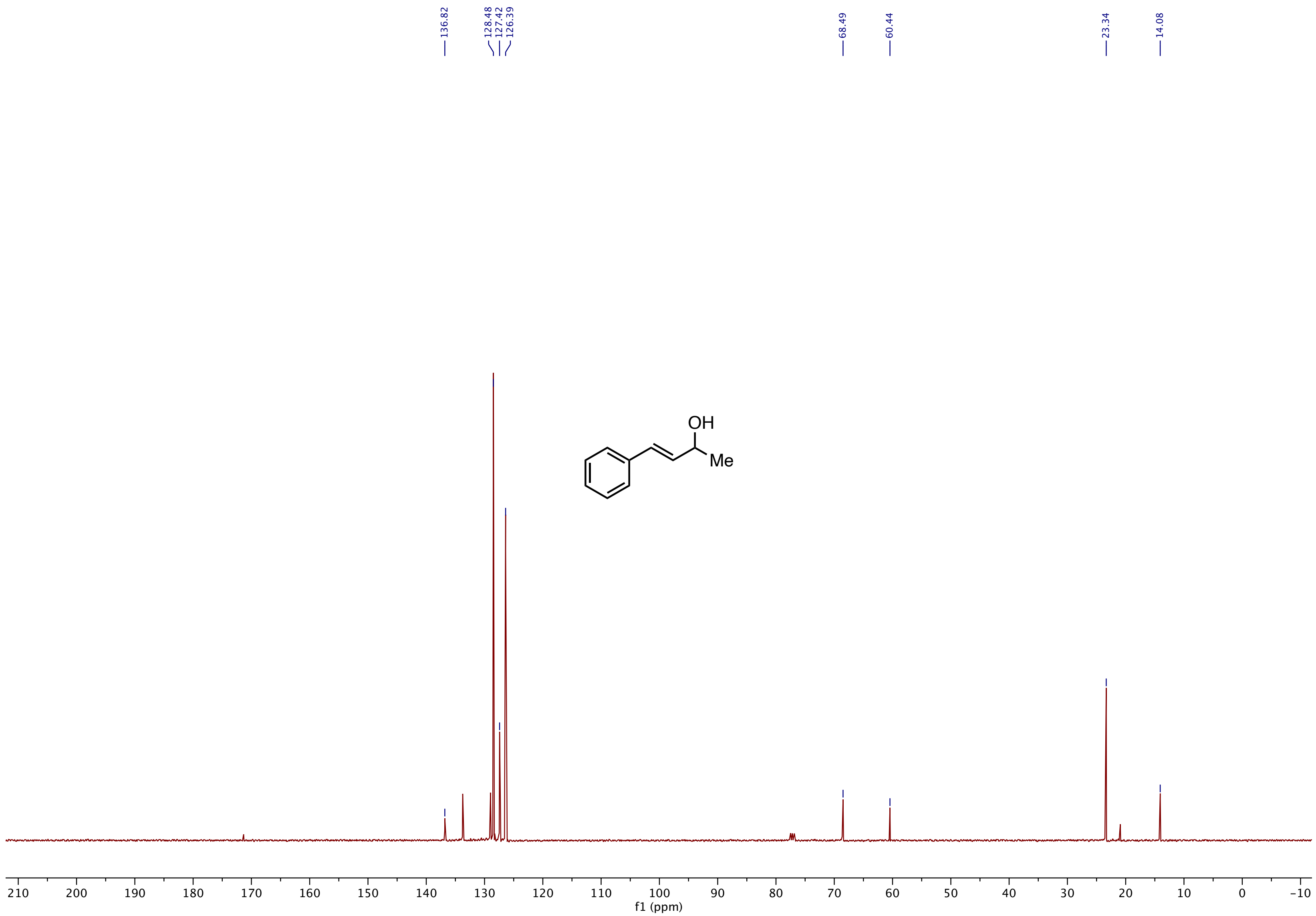

^{1}H NMR (400 MHz, $CDCl_3$): (±)-(*E*)-Ethyl (4-phenylbut-3-en-2-yl) carbonate (1c)

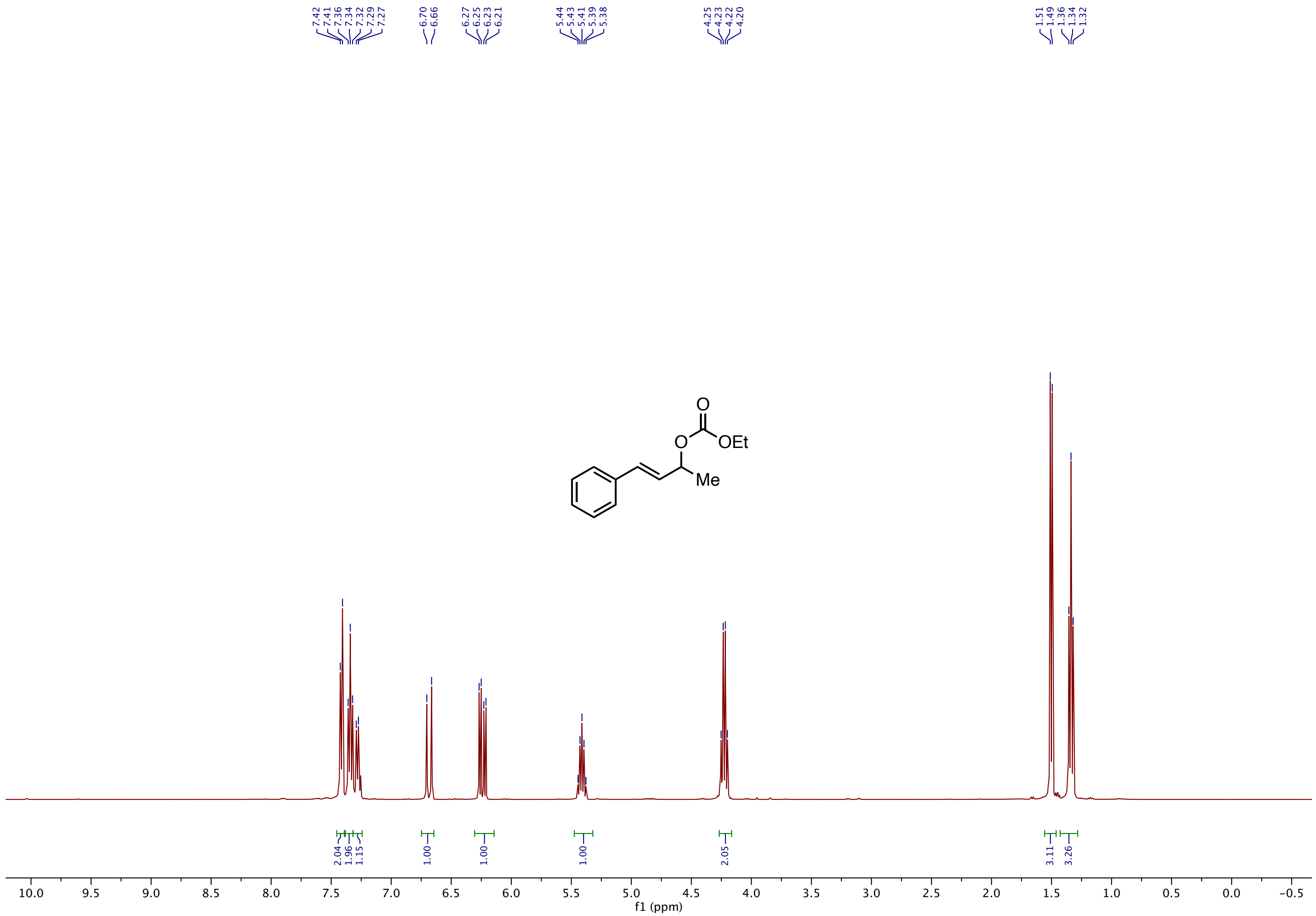

^{13}C NMR (101 MHz, $CDCl_3$): (±)-(*E*)-Ethyl (4-phenylbut-3-en-2-yl) carbonate (1c)

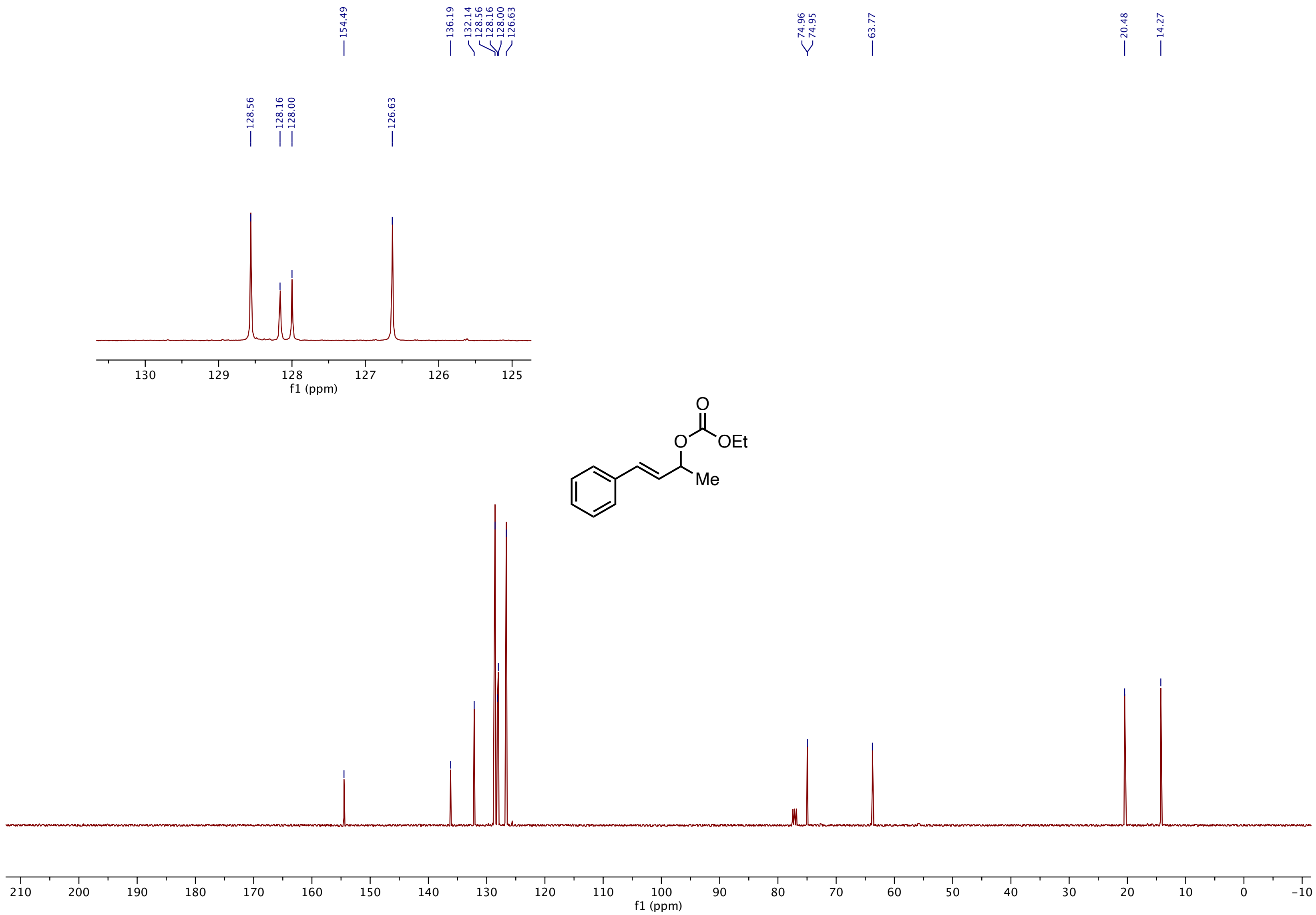

^{1}H NMR (400 MHz, MeOD): Tetrabutylammonium (*R*)-2'-(dicyclohexylphosphaneyl)-2,6-dimethoxy-[1,1'-biphenyl]-3-sulfonate [(*R*)-NBu4.sSPhos] (L1)

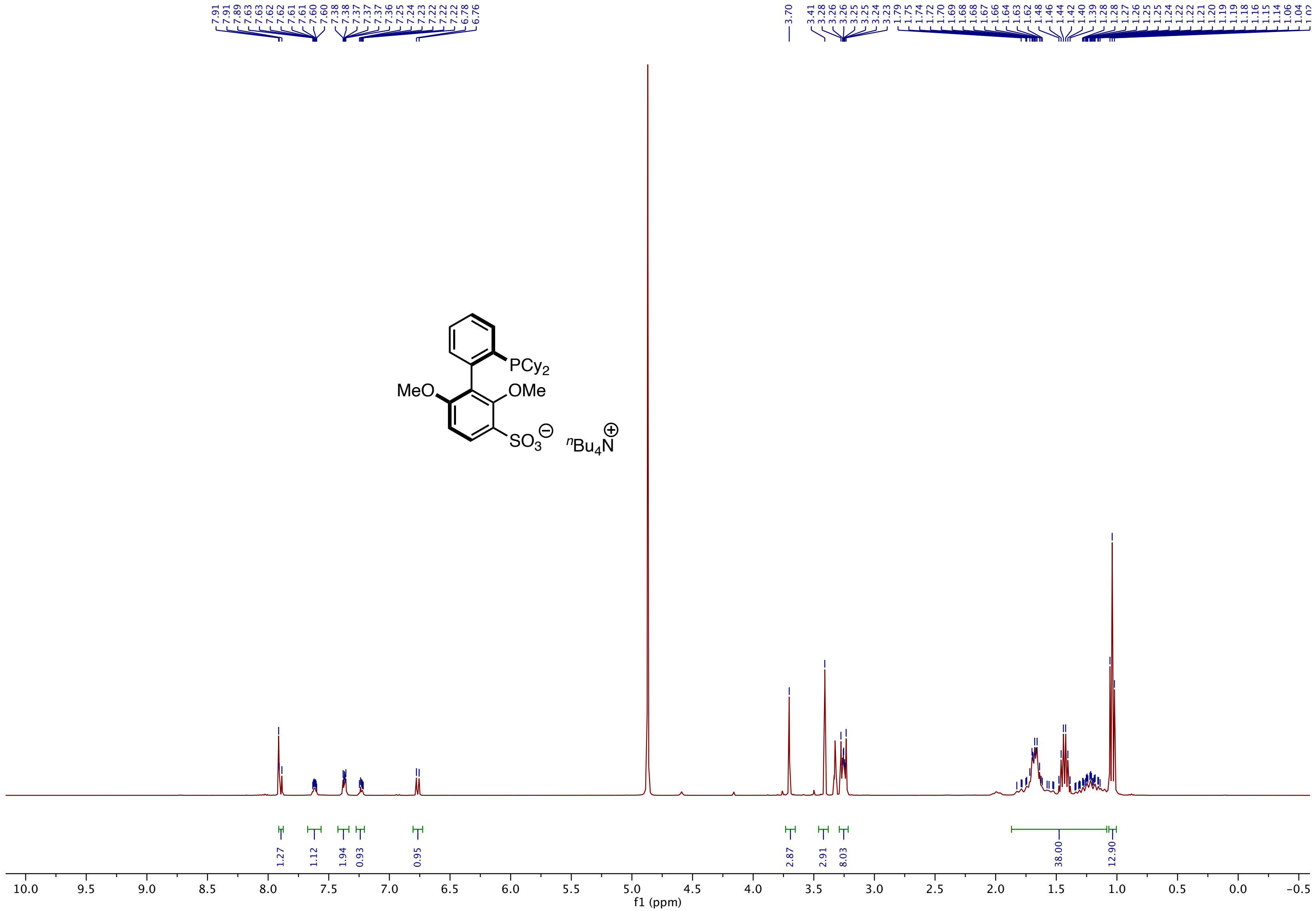

^{13}C NMR (101 MHz, MeOD): Tetrabutylammonium (*R*)-2'-(dicyclohexylphosphaneyl)-2,6-dimethoxy-[1,1'-biphenyl]-3-sulfonate [(*R*)-NBu4.sSPhos] (L1)

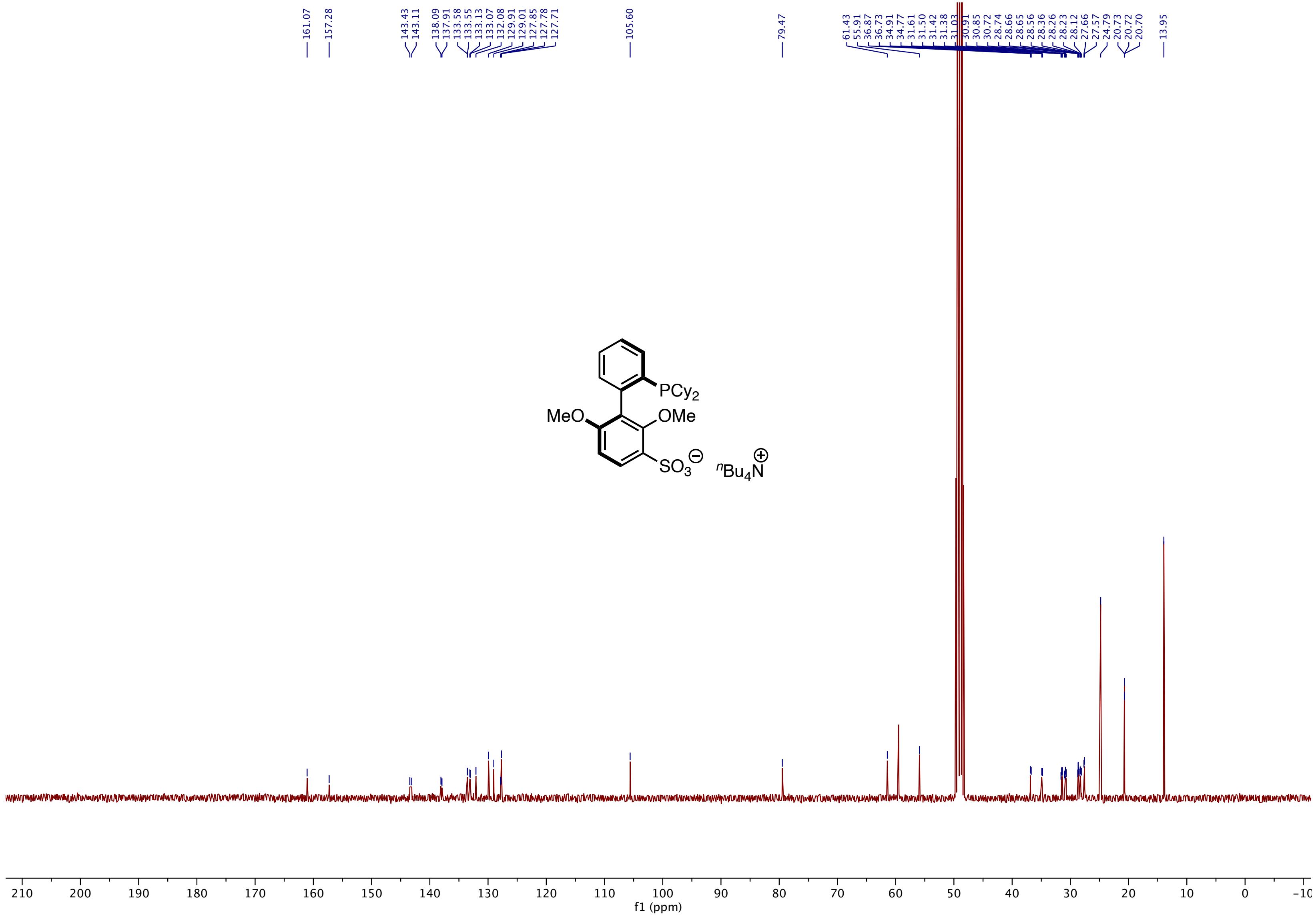

^{31}P NMR (162 MHz, MeOD): Tetrabutylammonium (*R*)-2'-(dicyclohexylphosphaneyl)-2,6-dimethoxy-[1,1'-biphenyl]-3-sulfonate [(*R*)-NBu4.sSPhos] (L1)

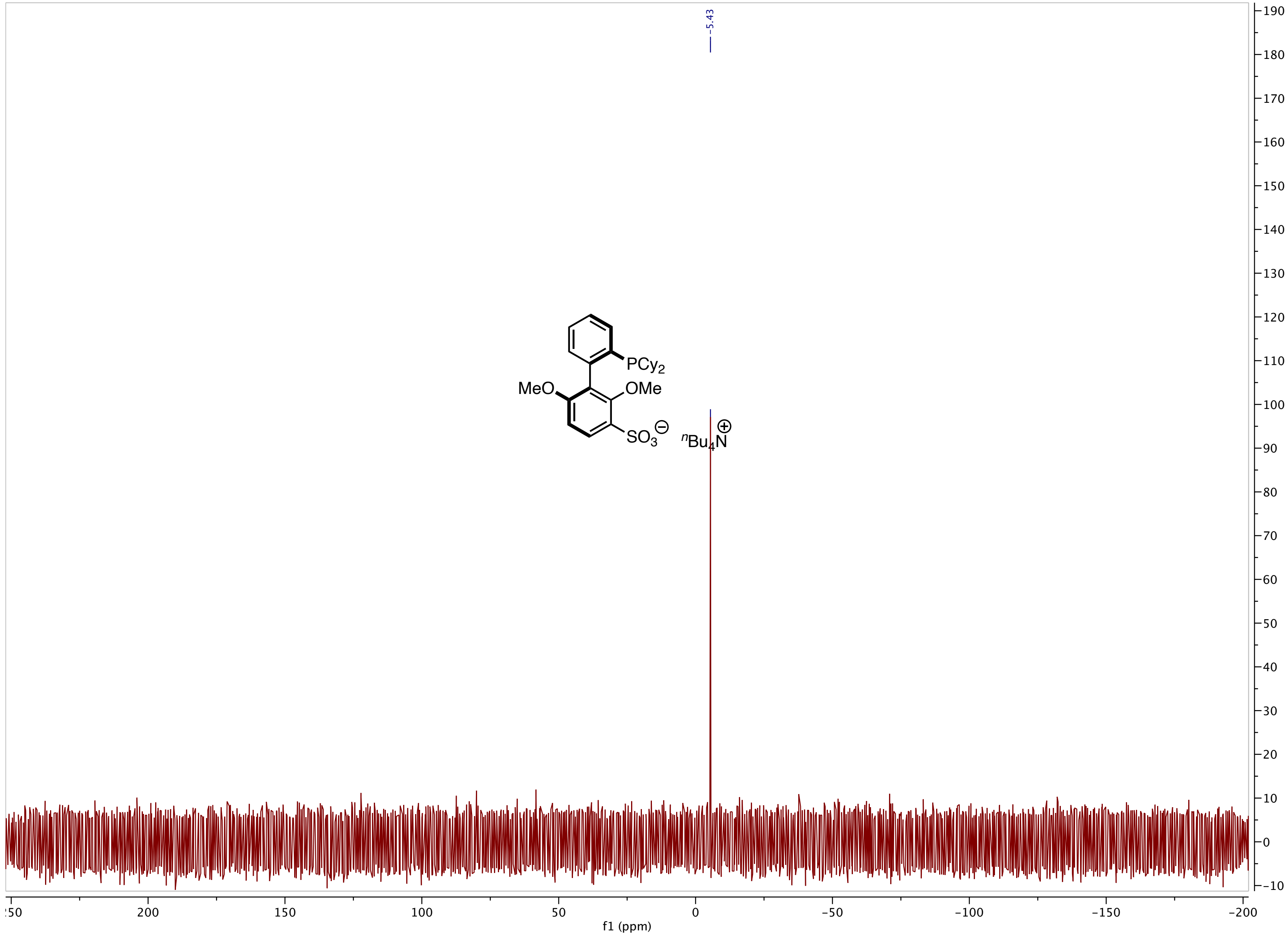

^{1}H NMR (400 MHz, MeOD): Potassium (*R*)-2'-(dicyclohexylphosphaneyl)-2,6-dimethoxy-[1,1'-biphenyl]-3-sulfonate (L2)

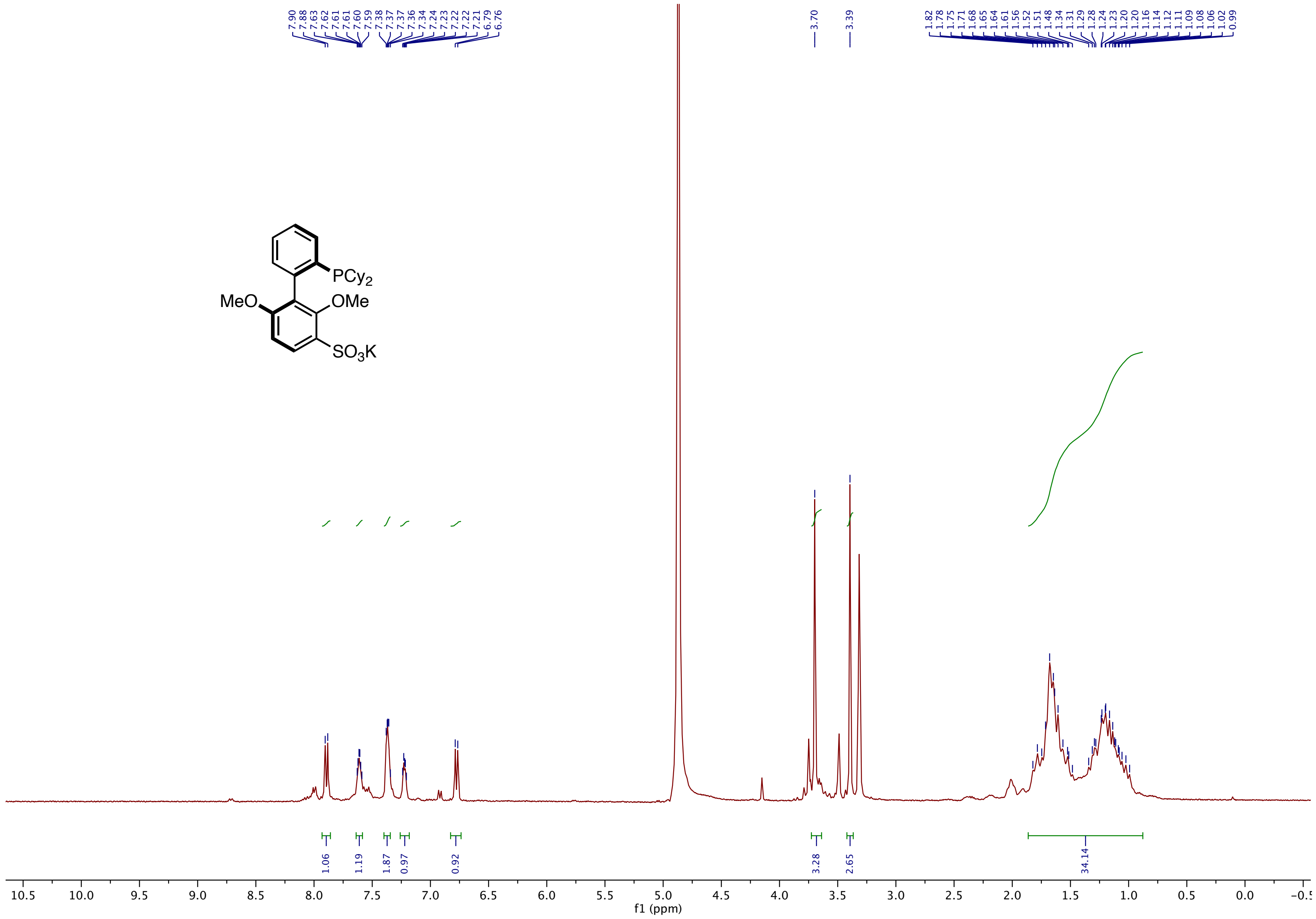

^{13}C NMR (101 MHz, MeOD): Potassium (*R*)-2'-(dicyclohexylphosphaneyl)-2,6-dimethoxy-[1,1'-biphenyl]-3-sulfonate (L2)

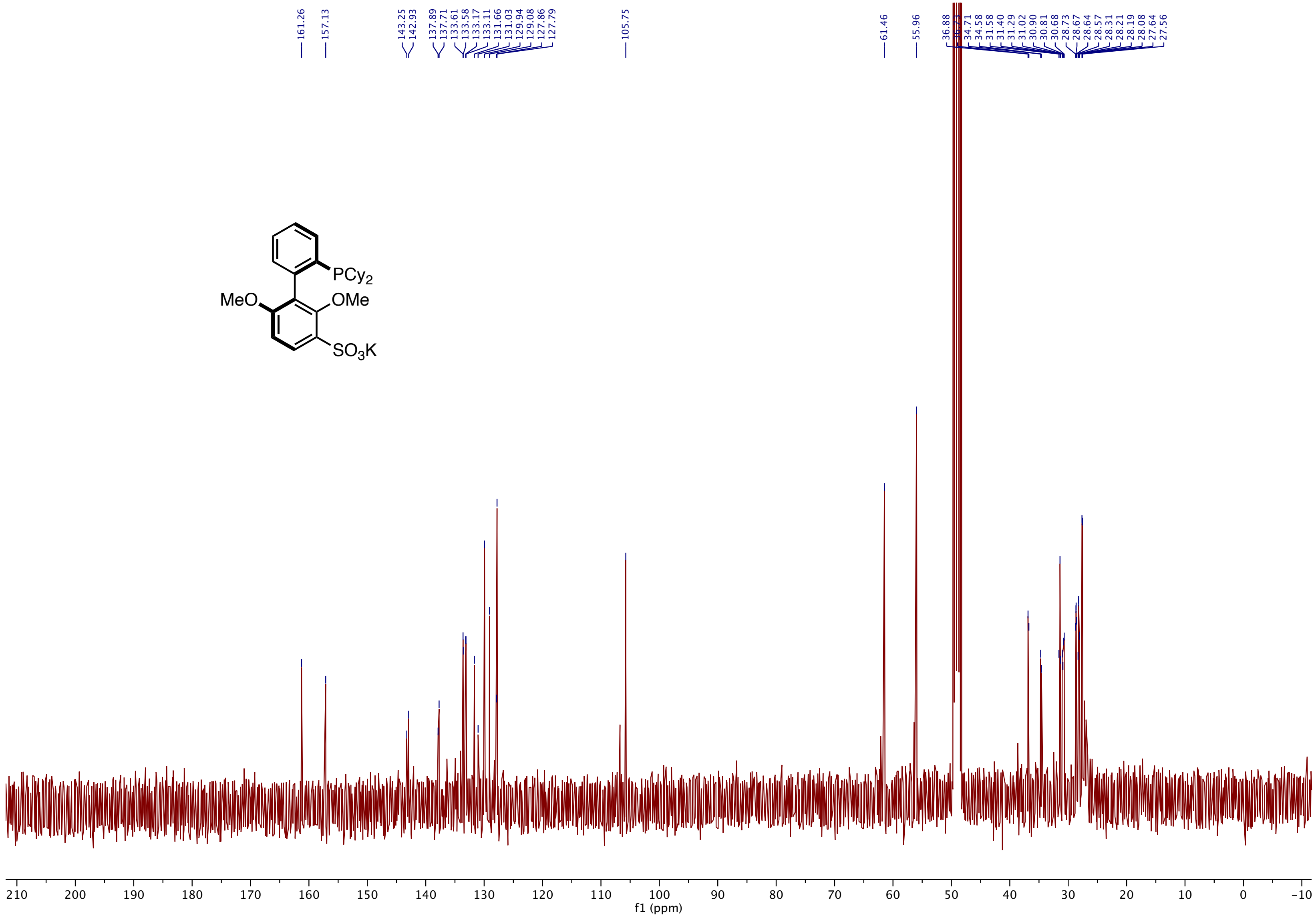

^{31}P NMR (162 MHz, MeOD): Potassium (*R*)-2'-(dicyclohexylphosphaneyl)-2,6-dimethoxy-[1,1'-biphenyl]-3-sulfonate (L2)

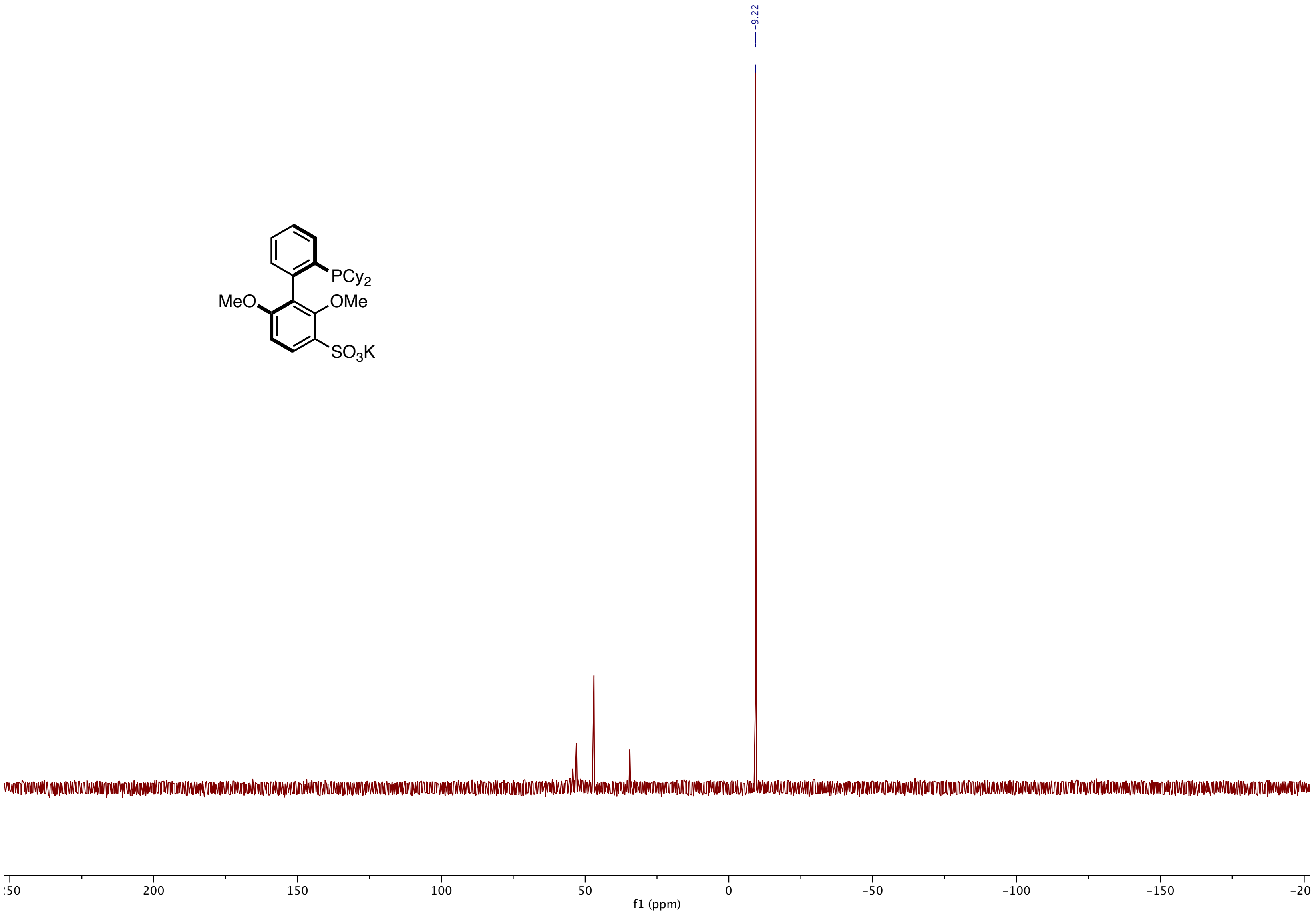

^{1}H NMR (400 MHz, MeOD): Cesium (*R*)-2'-(dicyclohexylphosphaneyl)-2,6-dimethoxy-[1,1'-biphenyl]-3-sulfonate (L3)

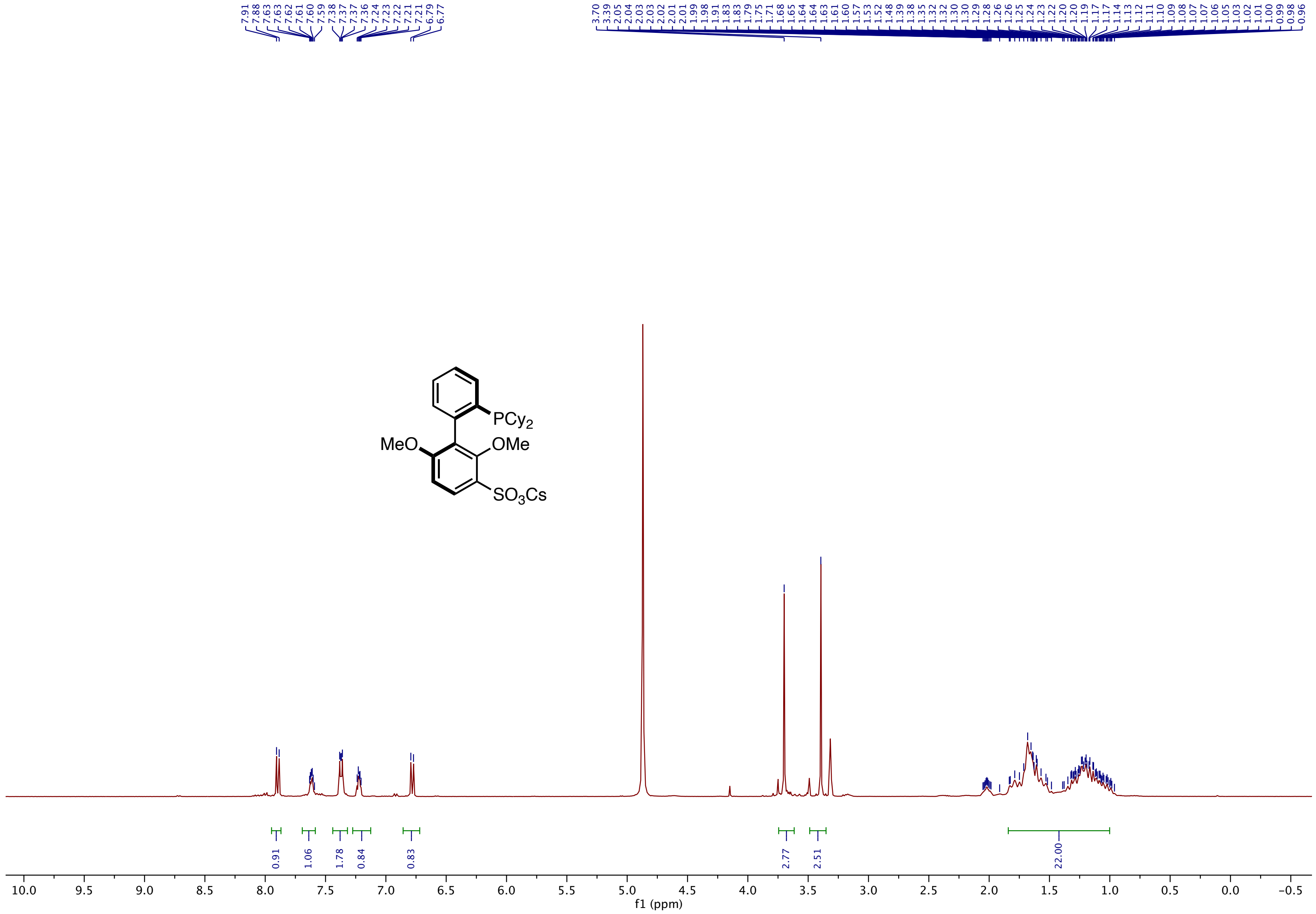

^{13}C NMR (101 MHz, MeOD): Cesium (*R*)-2'-(dicyclohexylphosphaneyl)-2,6-dimethoxy-[1,1'-biphenyl]-3-sulfonate (L3)

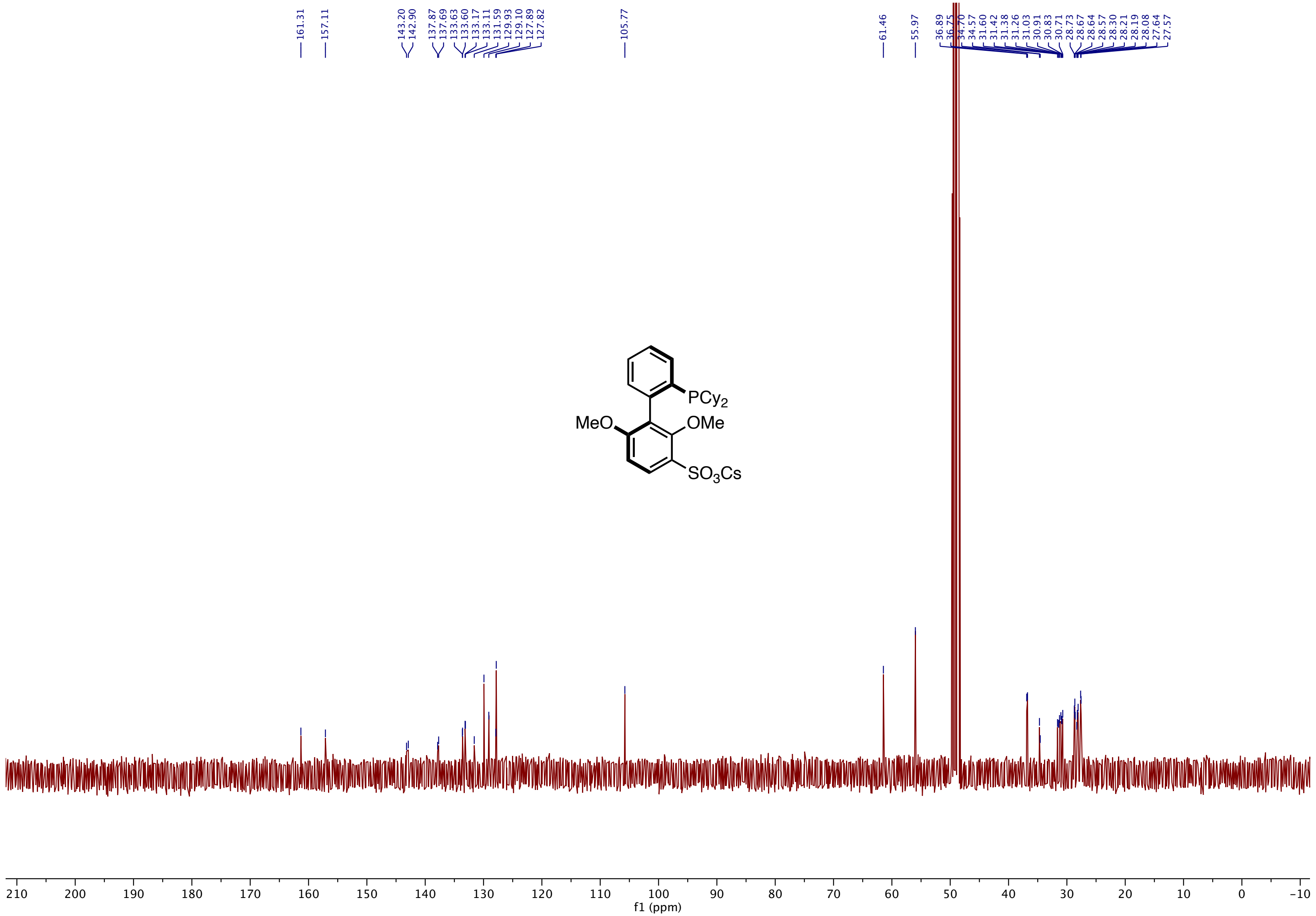

^{31}P NMR (162 MHz, MeOD): Cesium (*R*)-2'-(dicyclohexylphosphaneyl)-2,6-dimethoxy-[1,1'-biphenyl]-3-sulfonate (L3)

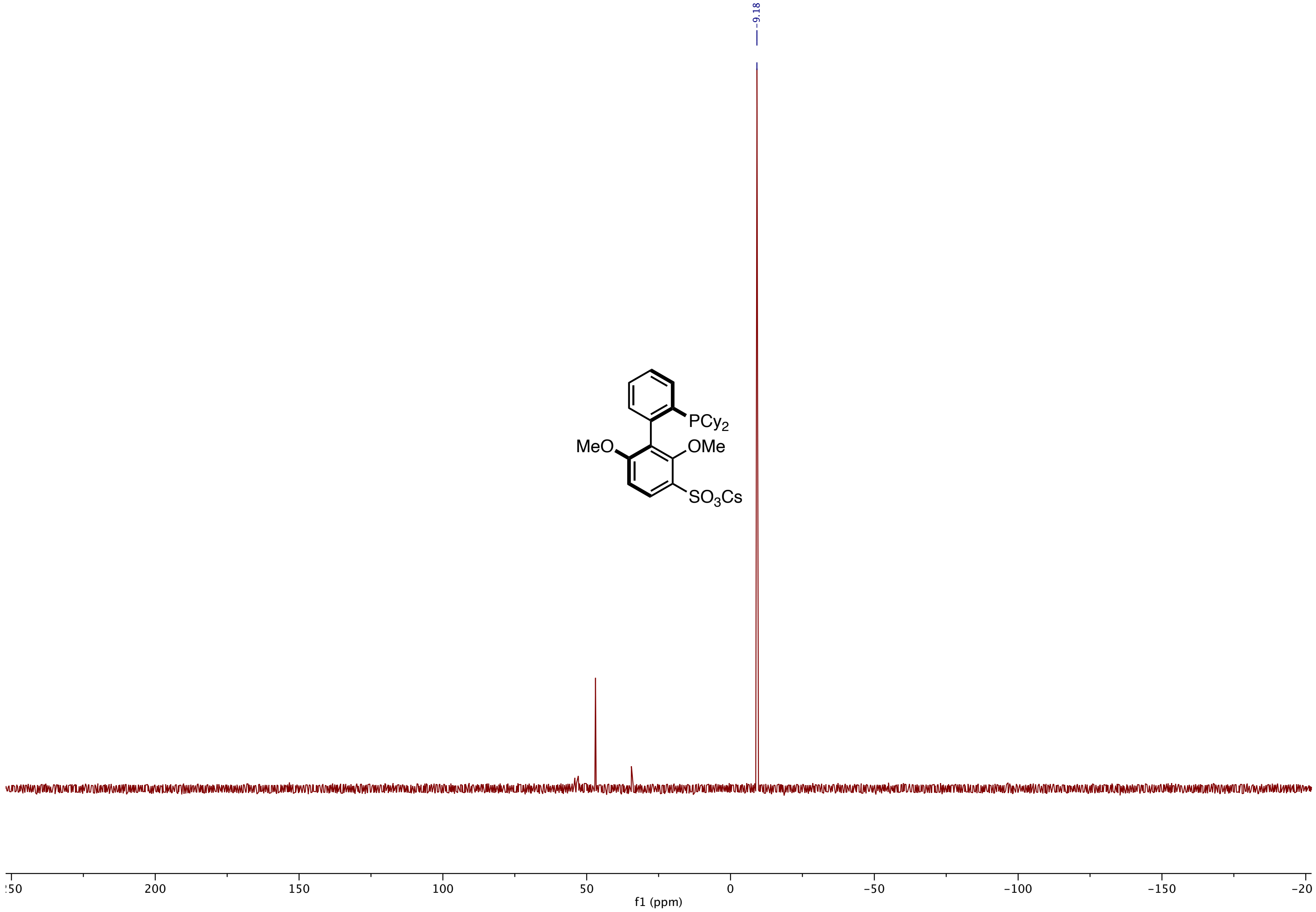

^{1}H NMR (400 MHz, MeOD): Tetramethylammonium (*R*)-2'-(dicyclohexylphosphaneyl)-2,6-dimethoxy-[1,1'-biphenyl]-3-sulfonate (L4)

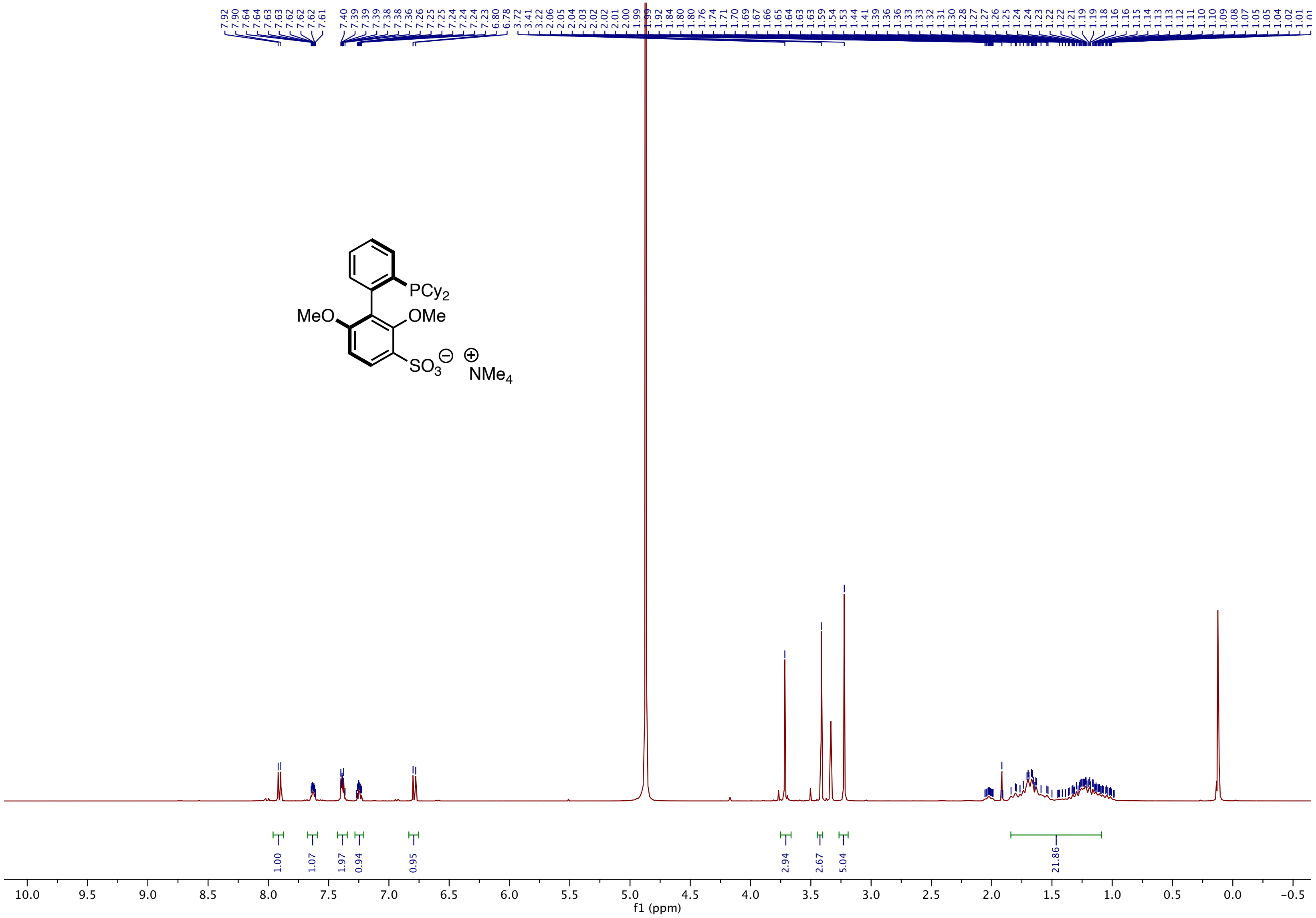

^{13}C NMR (101 MHz, MeOD): Tetramethylammonium (*R*)-2'-(dicyclohexylphosphaneyl)-2,6-dimethoxy-[1,1'-biphenyl]-3-sulfonate (L4)

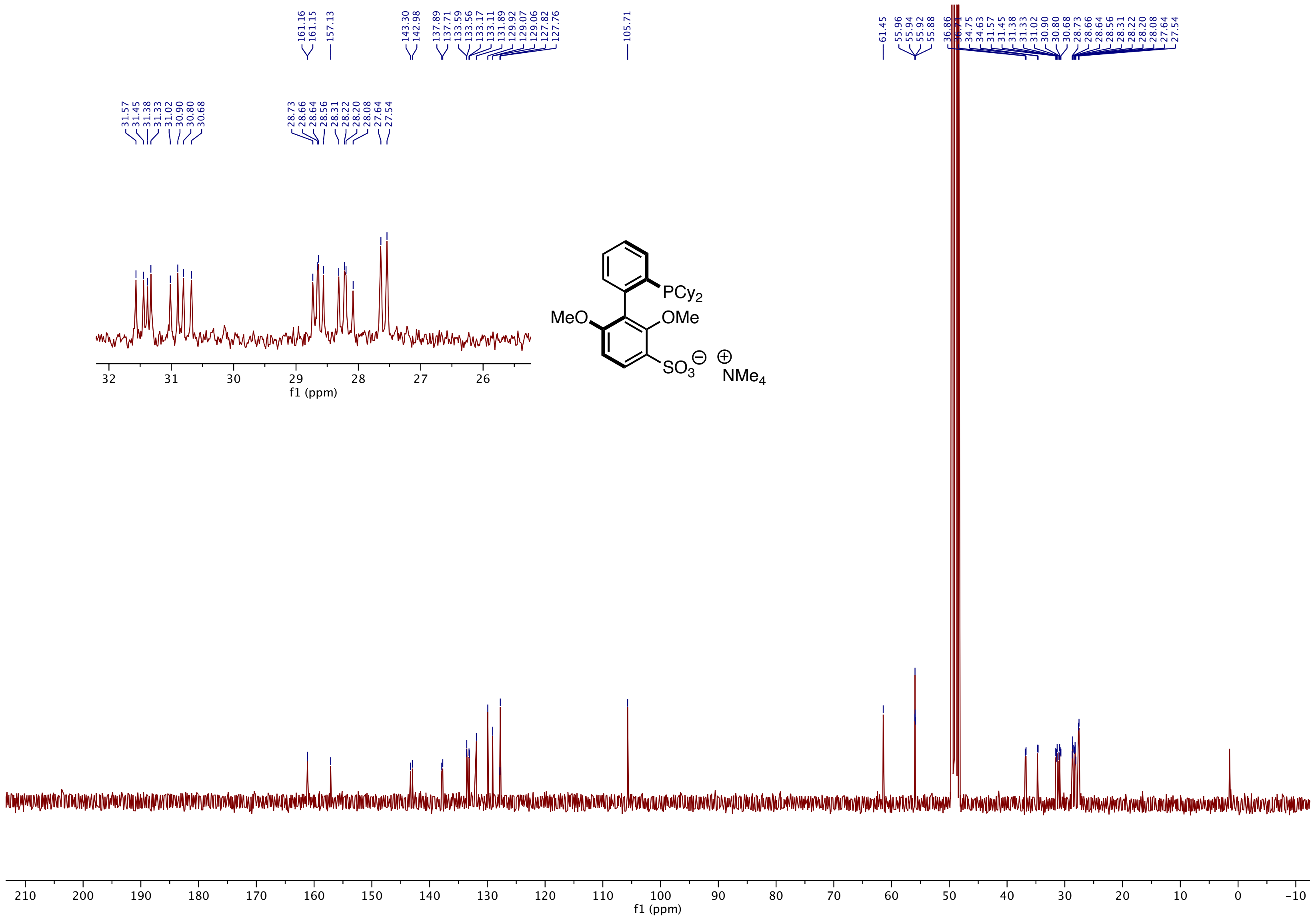

^{31}P NMR (162 MHz, MeOD): Tetramethylammonium (*R*)-2'-(dicyclohexylphosphaneyl)-2,6-dimethoxy-[1,1'-biphenyl]-3-sulfonate (L4)

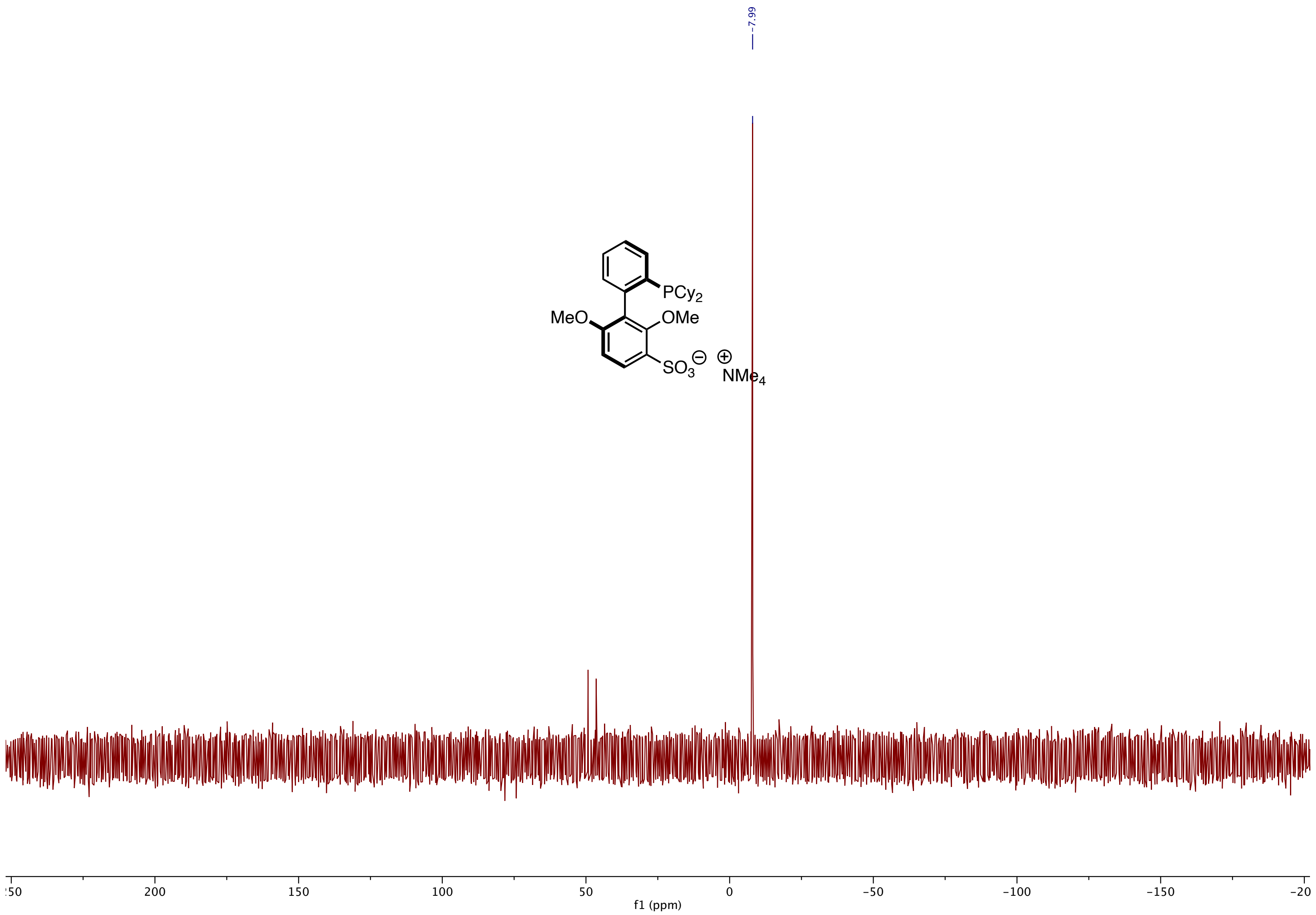

^{1}H NMR (400 MHz, MeOD): Tetraethylammonium (*R*)-2'-(dicyclohexylphosphaneyl)-2,6-dimethoxy-[1,1'-biphenyl]-3 sulfonate (L5)

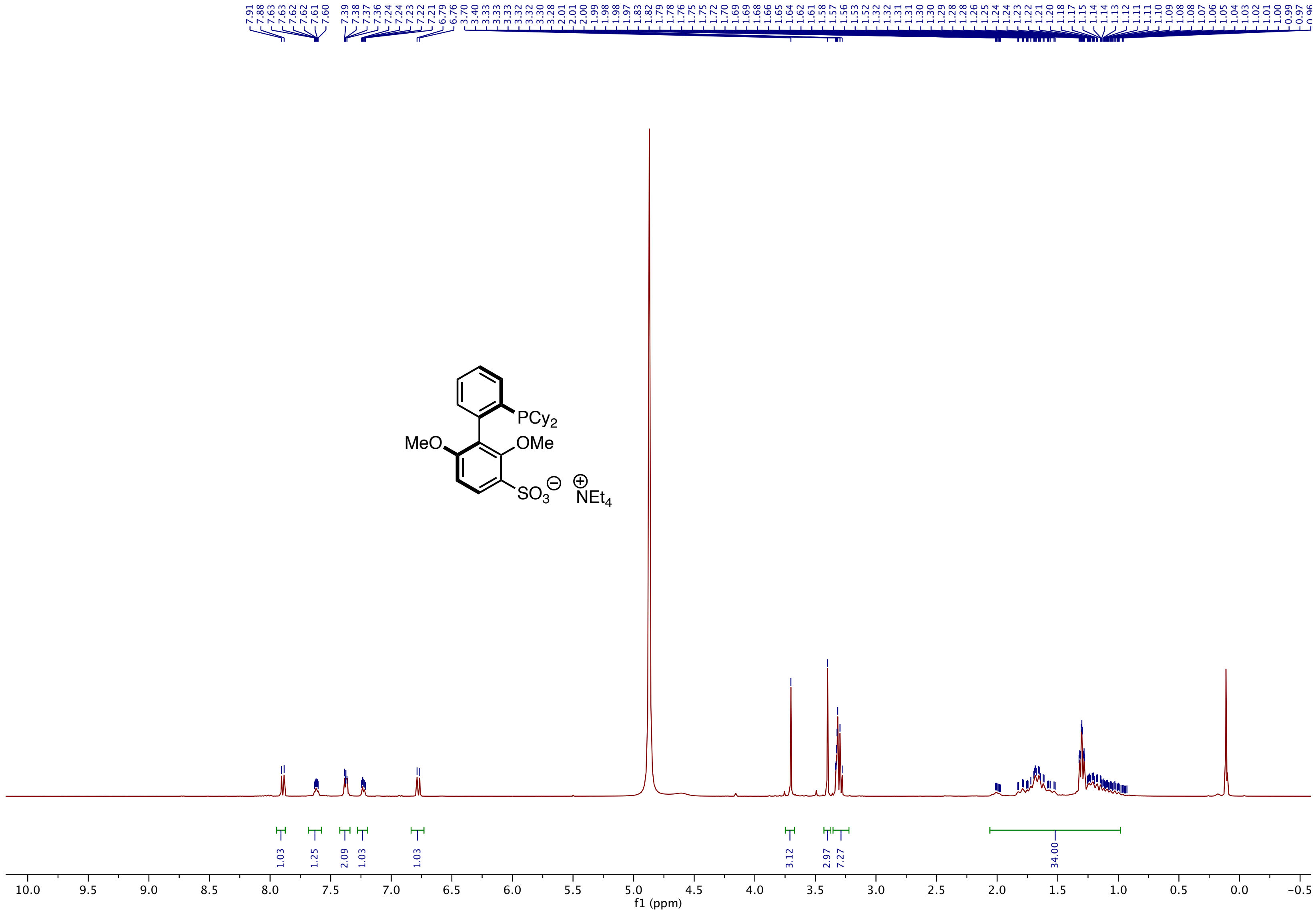

^{13}C NMR (101 MHz, MeOD): Tetraethylammonium (*R*)-2'-(dicyclohexylphosphaneyl)-2,6-dimethoxy-[1,1'-biphenyl]-3 sulfonate (L5)

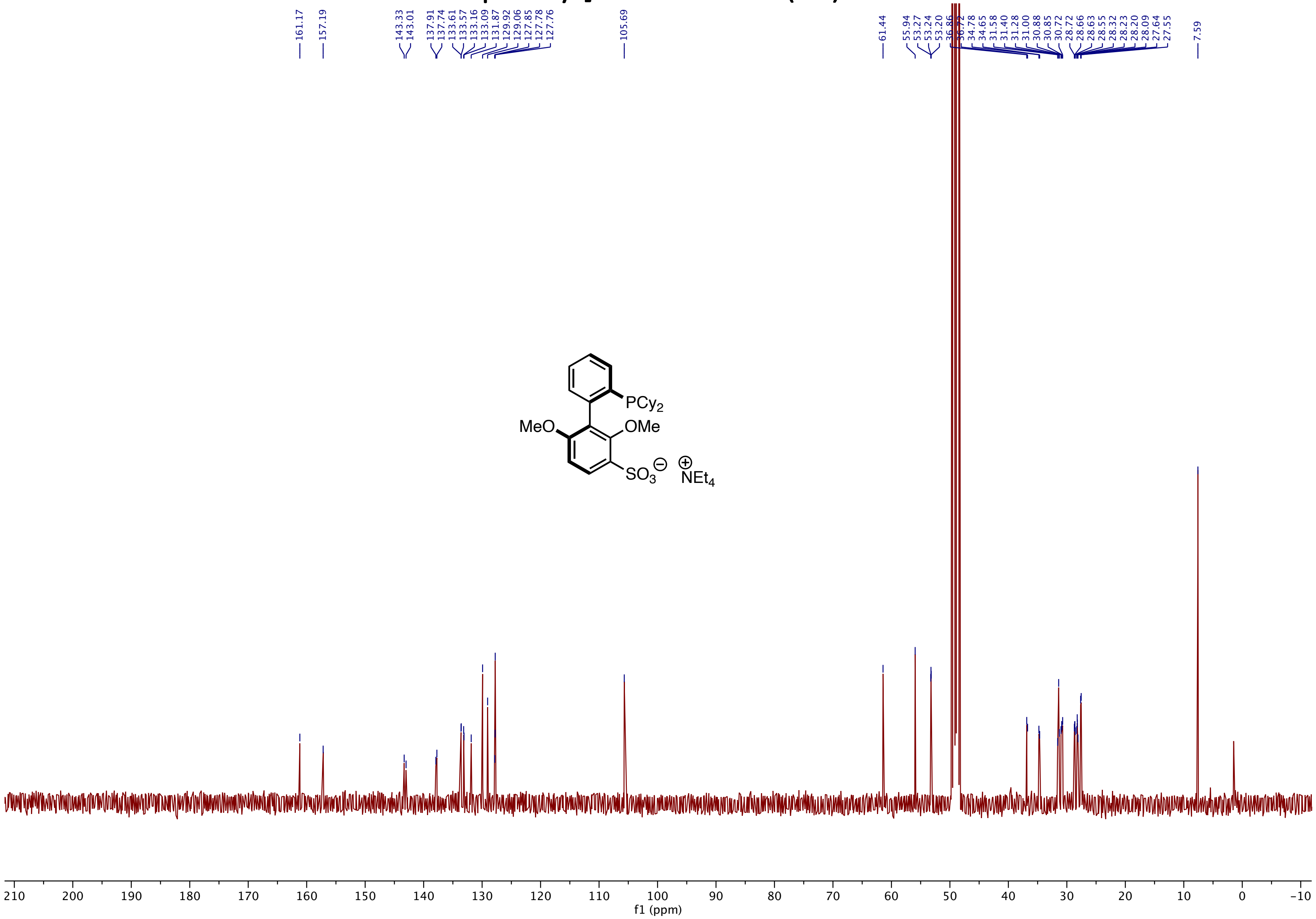

^{31}P NMR (162 MHz, MeOD): Tetraethylammonium (*R*)-2'-(dicyclohexylphosphaneyl)-2,6-dimethoxy-[1,1'-biphenyl]-3 sulfonate (L5)

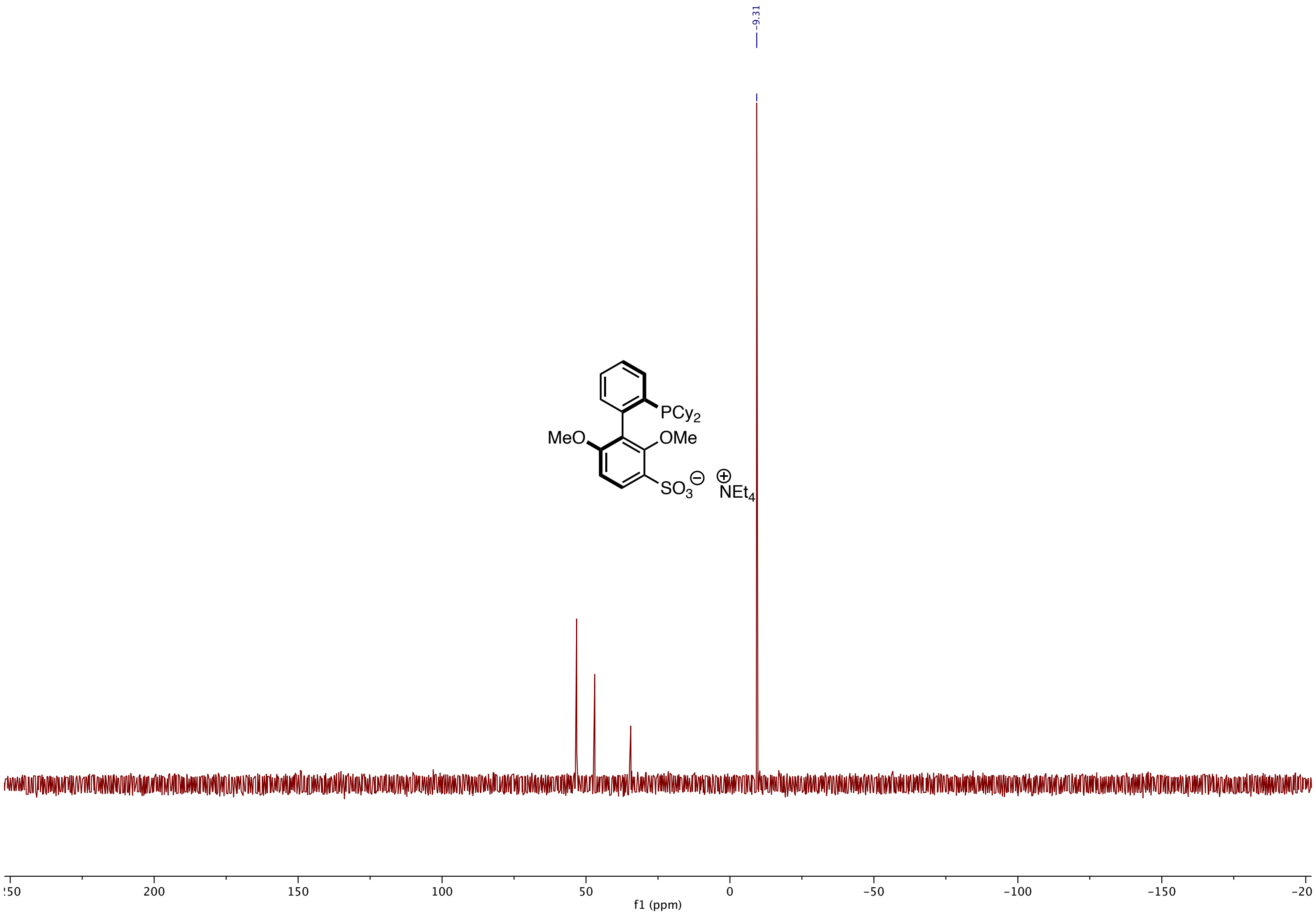

^{1}H NMR (400 MHz, MeOD): Tetrahexylammonium (*R*)-2'-(dicyclohexylphosphaneyl)-2,6-dimethoxy-[1,1'-biphenyl]-3-sulfonate (L6)

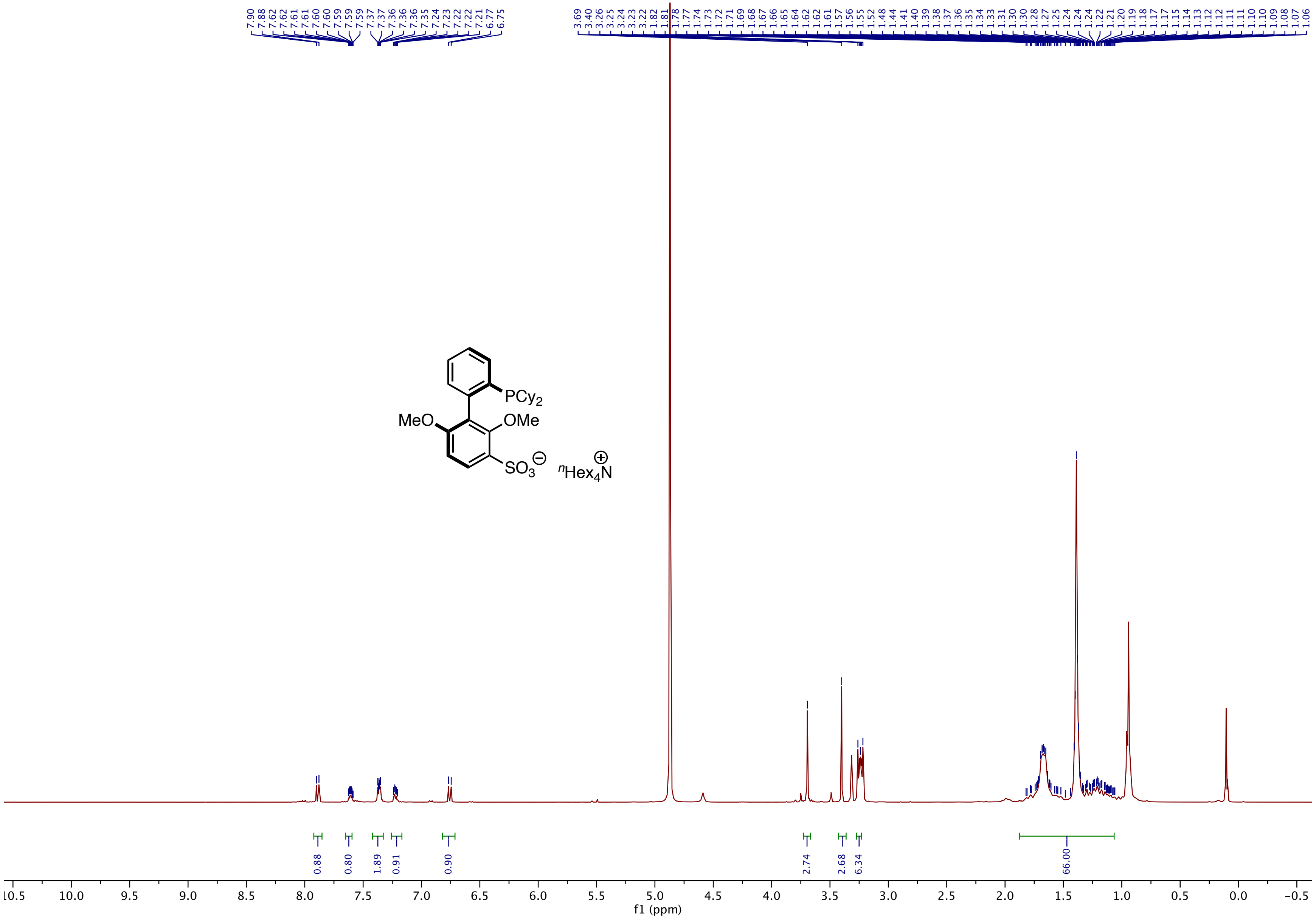

^{13}C NMR (101 MHz, MeOD): Tetrahexylammonium (*R*)-2'-(dicyclohexylphosphaneyl)-2,6-dimethoxy-[1,1'-biphenyl]-3-sulfonate (L6)

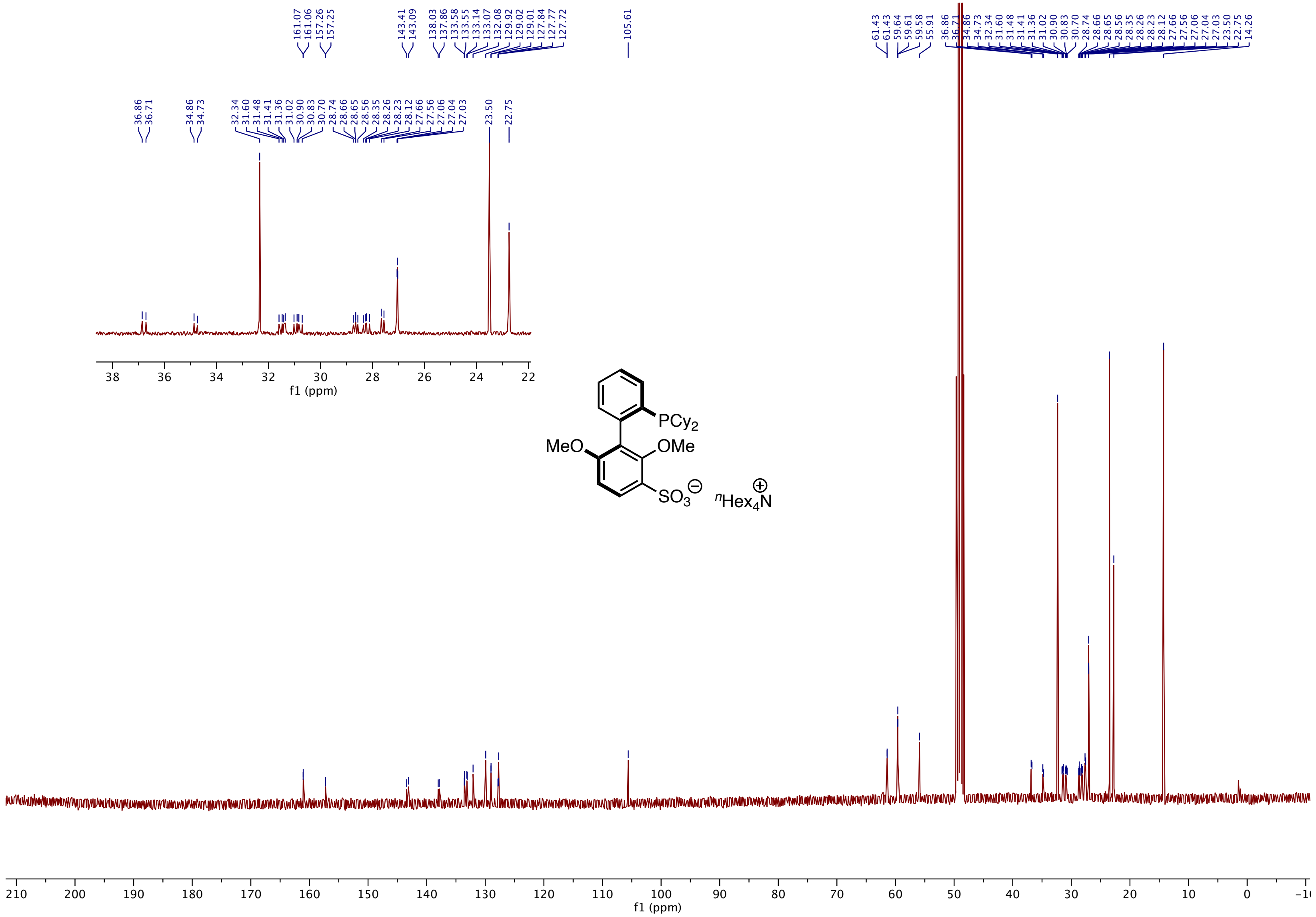

^{31}P NMR (162 MHz, MeOD): Tetrahexylammonium (*R*)-2'-(dicyclohexylphosphaneyl)-2,6-dimethoxy-[1,1'-biphenyl]-3-sulfonate (L6)

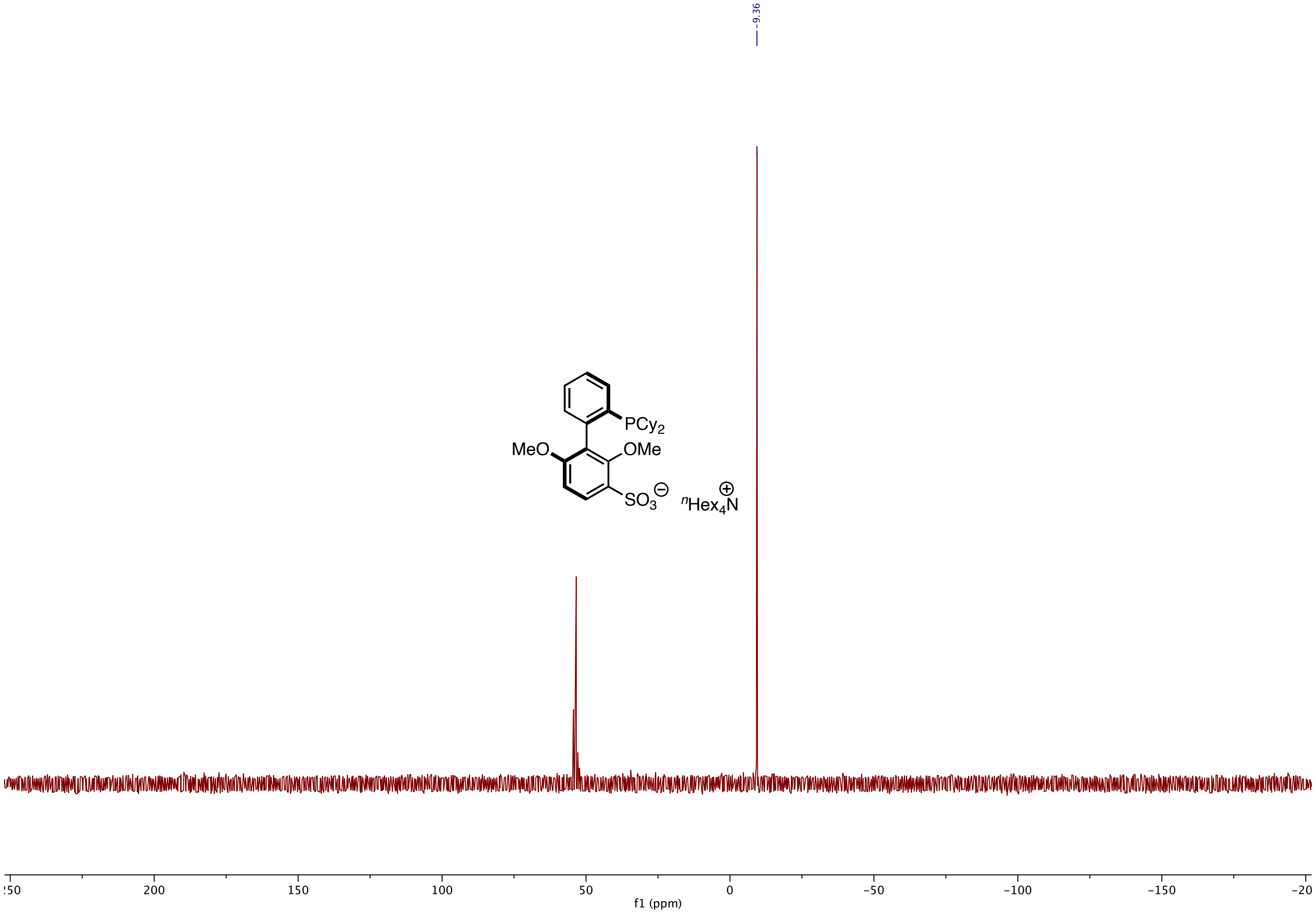

^1H NMR (400 MHz, $CDCl_3$): (*1S,4S,5R*)-5-Ethyl-2-((*R*)-hydroxy(6-methoxyquinolin-4-yl)methyl)-1-((3,3'',5,5''-tetra-tert-butyl-[1,1':3',1''-terphenyl]-5'-yl)methyl)quinuclidin-1-ium (*R*)-2'-(dicyclohexylphosphaneyl)-2,6-dimethoxy-[1,1'-biphenyl]-3-sulfonate (L7)

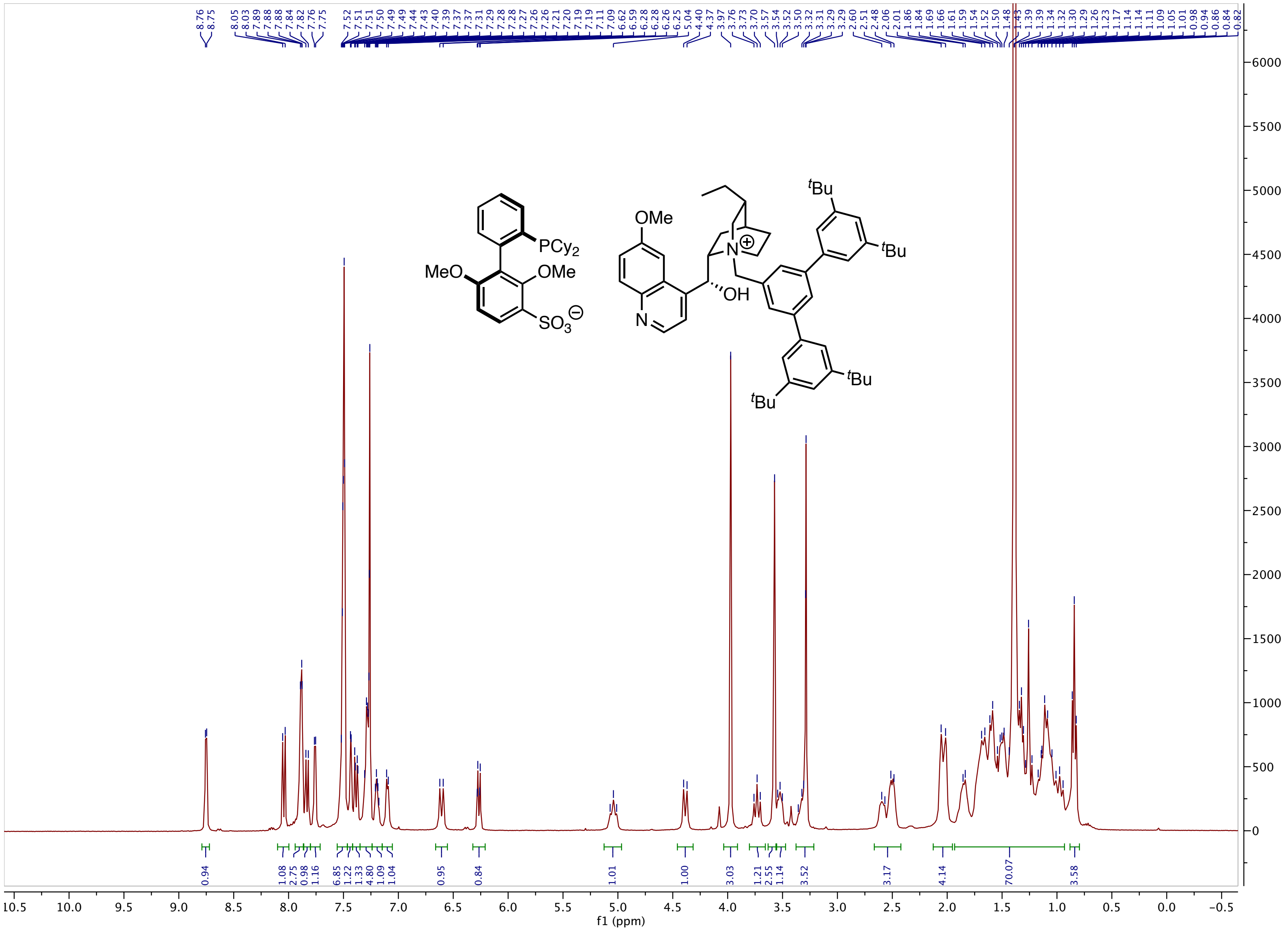

^{13}C NMR (176 MHz, $CDCl_3$): (*1S,4S,5R*)-5-Ethyl-2-((*R*)-hydroxy(6-methoxyquinolin-4-yl)methyl)-1-((3,3'',5,5''-tetra-tert-butyl-[1,1':3',1''-terphenyl]-5'-yl)methyl)quinuclidin-1-ium (*R*)-2'-(dicyclohexylphosphaneyl)-2,6-dimethoxy-[1,1'-biphenyl]-3-sulfonate (L7)

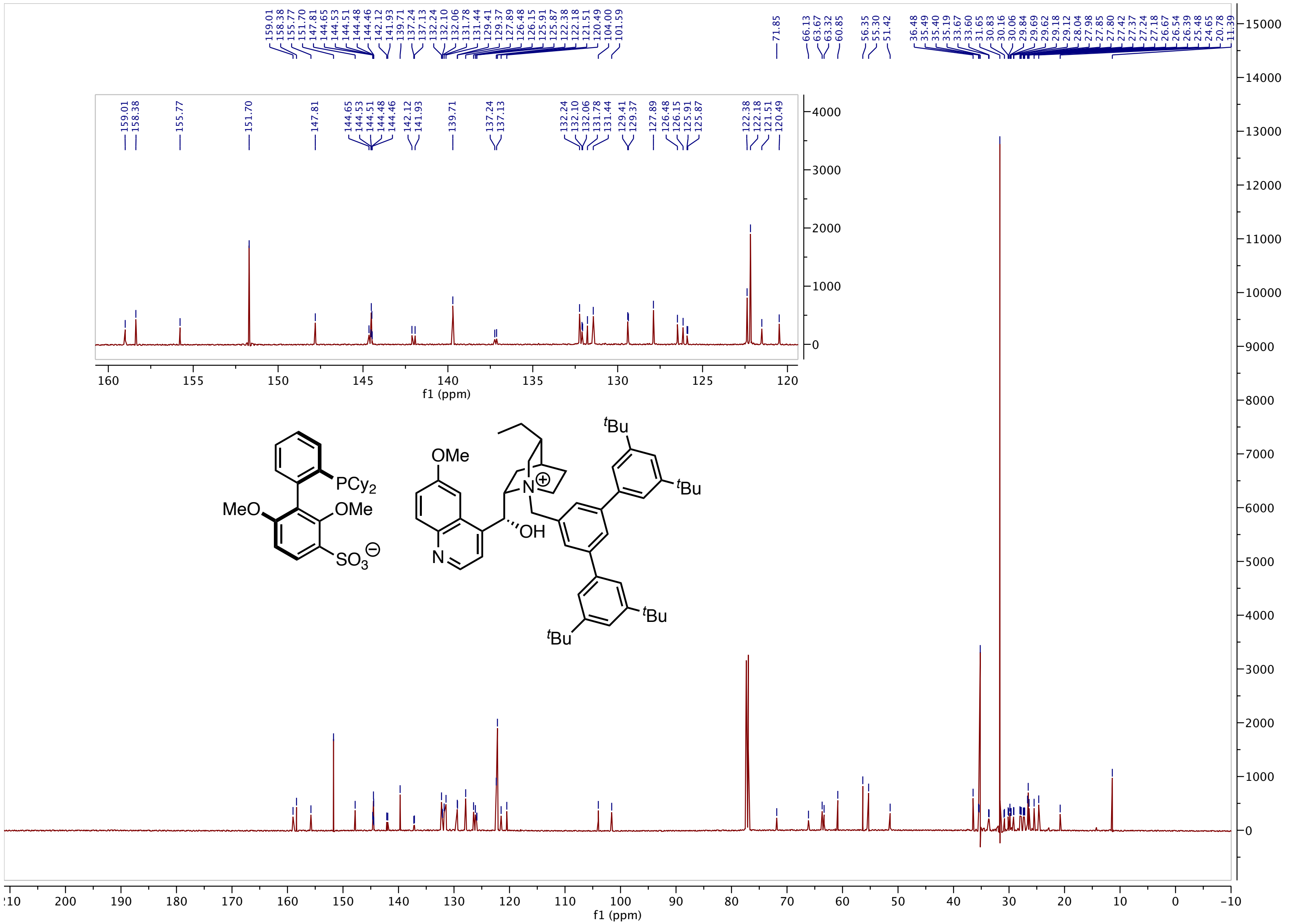

^{31}P NMR (162 MHz, CDCl$_3$): (*1S,4S,5R*)-5-Ethyl-2-((*R*)-hydroxy(6-methoxyquinolin-4-yl)methyl)-1-((3,3'',5,5''-tetra-tert-butyl-[1,1':3',1''-terphenyl]-5'-yl)methyl)quinuclidin-1-ium (*R*)-2'-(dicyclohexylphosphaneyl)-2,6-dimethoxy-[1,1'-biphenyl]-3-sulfonate (L7)

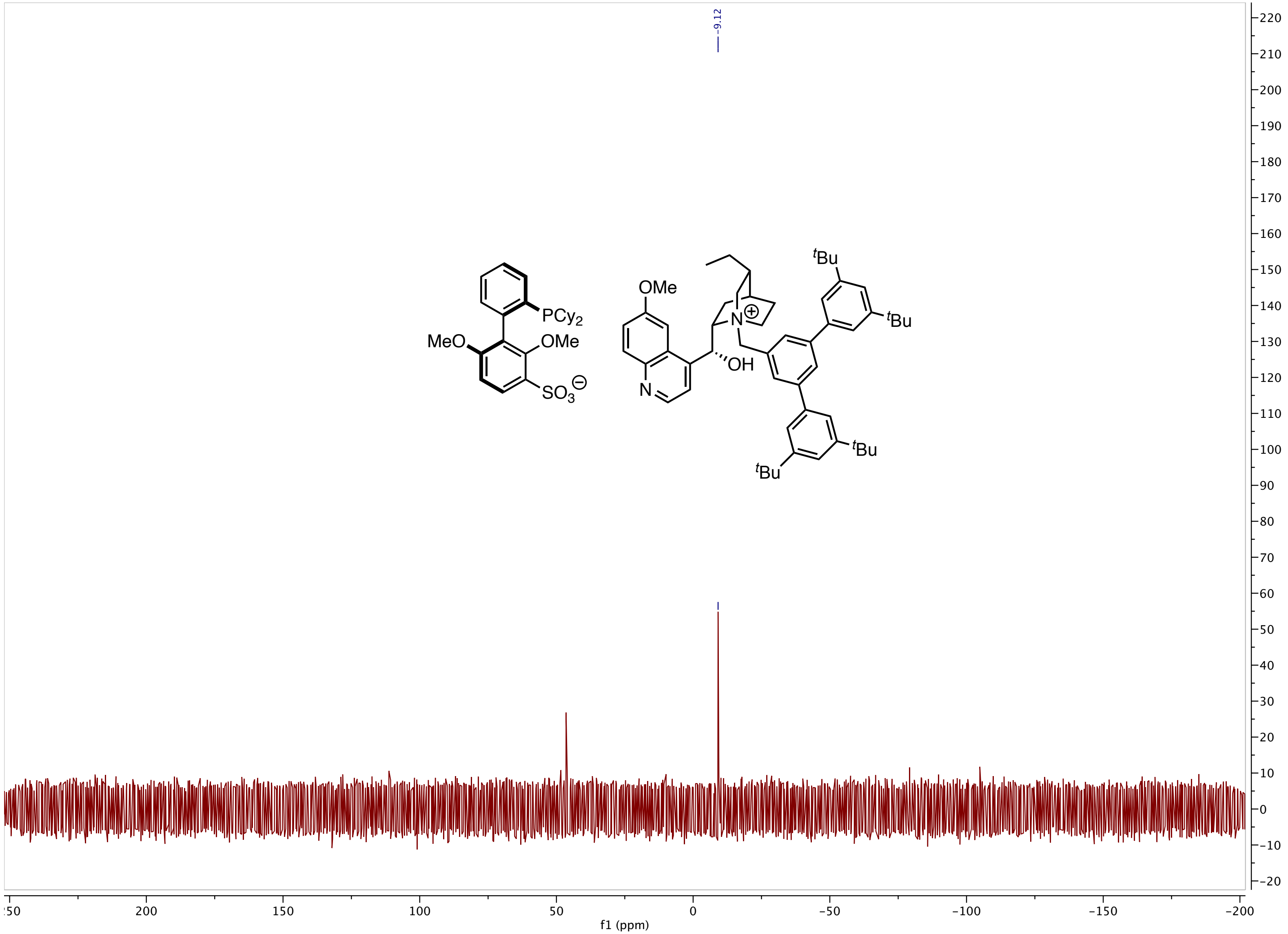

^{1}H NMR (700 MHz, $CDCl_3$): (*1S,4S,5R*)-5-Ethyl-2-((*R*)-hydroxy(6-methoxyquinolin-4-yl)methyl)-1-((3,3'',5,5''-tetra-tert-butyl-[1,1':3',1''-terphenyl]-5'-yl)methyl)quinuclidin-1-ium (*S*)-2'-(dicyclohexylphosphaneyl)-2,6-dimethoxy-[1,1'-biphenyl]-3-sulfonate (L8)

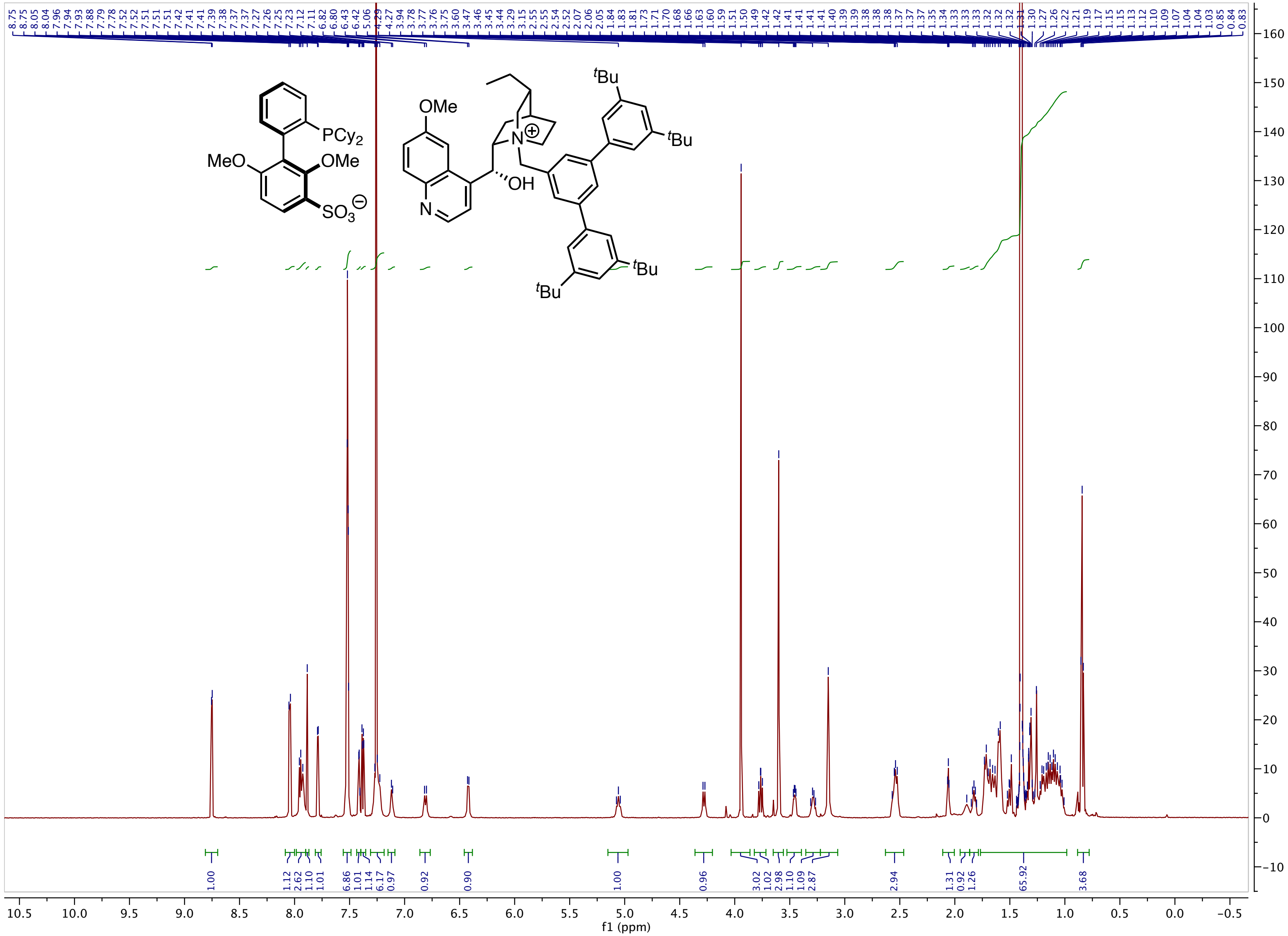

^{13}C NMR (176 MHz, $CDCl_3$): (*1S,4S,5R*)-5-Ethyl-2-((*R*)-hydroxy(6-methoxyquinolin-4-yl)methyl)-1-((3,3'',5,5''-tetra-tert-butyl-[1,1':3',1''-terphenyl]-5'-yl)methyl)quinuclidin-1-ium (*S*)-2'-(dicyclohexylphosphaneyl)-2,6-dimethoxy-[1,1'-biphenyl]-3-sulfonate (L8)

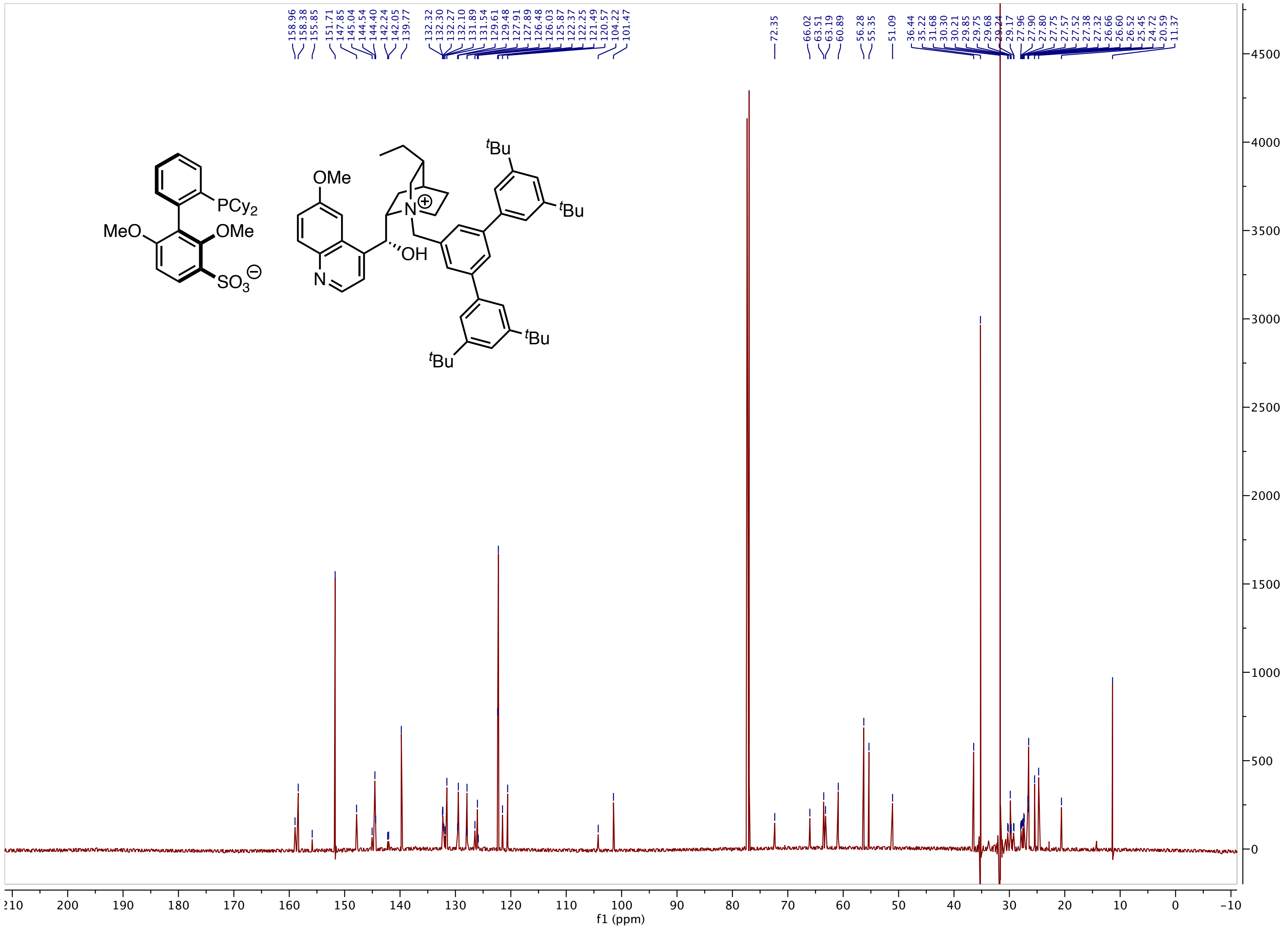

^{31}P NMR (203 MHz, $CDCl_3$): (*1S,4S,5R*)-5-Ethyl-2-((*R*)-hydroxy(6-methoxyquinolin-4-yl)methyl)-1-((3,3'',5,5''-tetra-tert-butyl-[1,1':3',1''-terphenyl]-5'-yl)methyl)quinuclidin-1-ium (*S*)-2'-(dicyclohexylphosphaneyl)-2,6-dimethoxy-[1,1'-biphenyl]-3-sulfonate (L8)

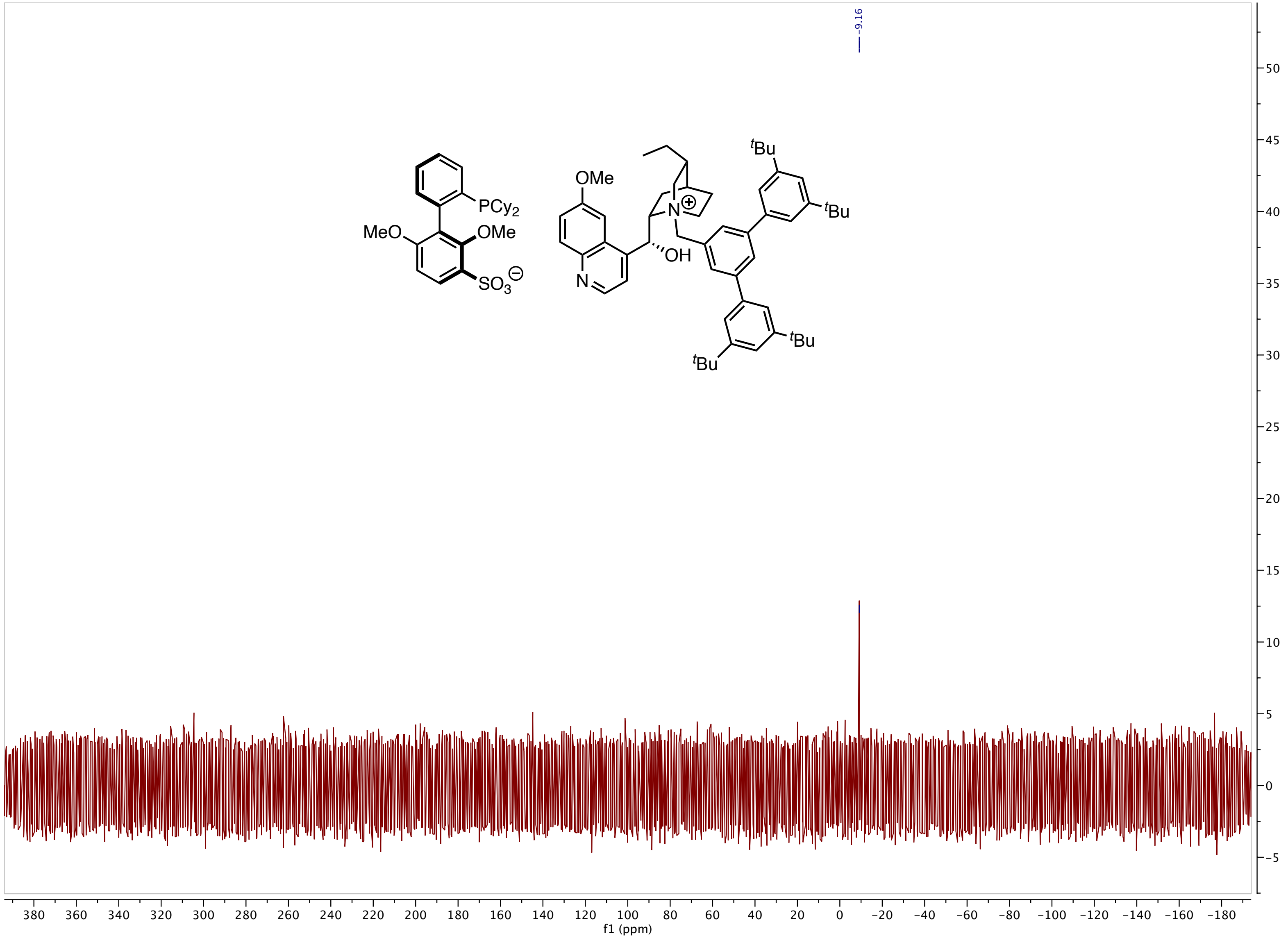

^{1}H NMR (700 MHz, $CDCl_3$): Dimethyl (*R,E*)-2-(1,3-diphenylallyl)malonate (3a)

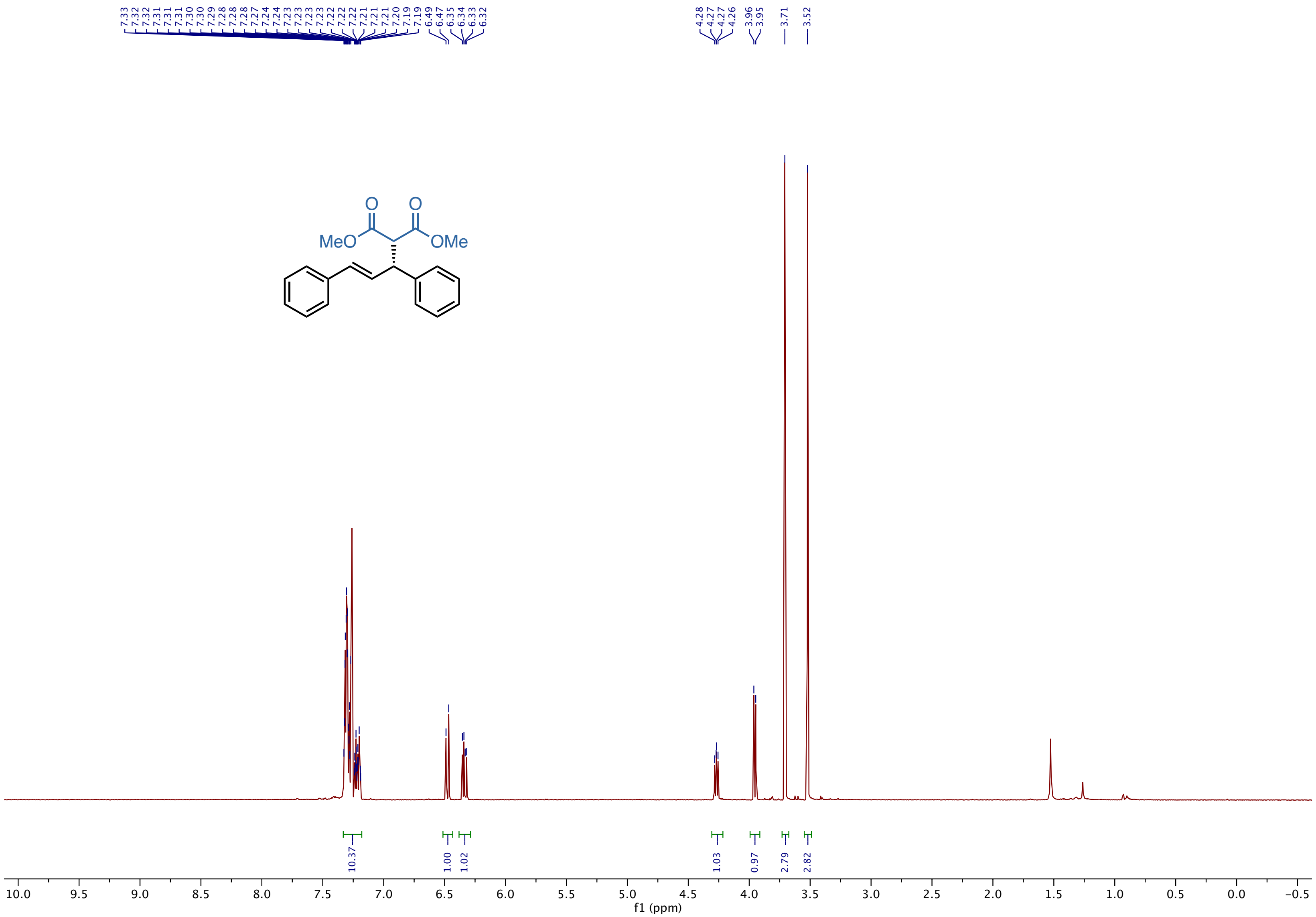

^{13}C NMR (176 MHz, $CDCl_3$): Dimethyl (*R,E*)-2-(1,3-diphenylallyl)malonate (3a)

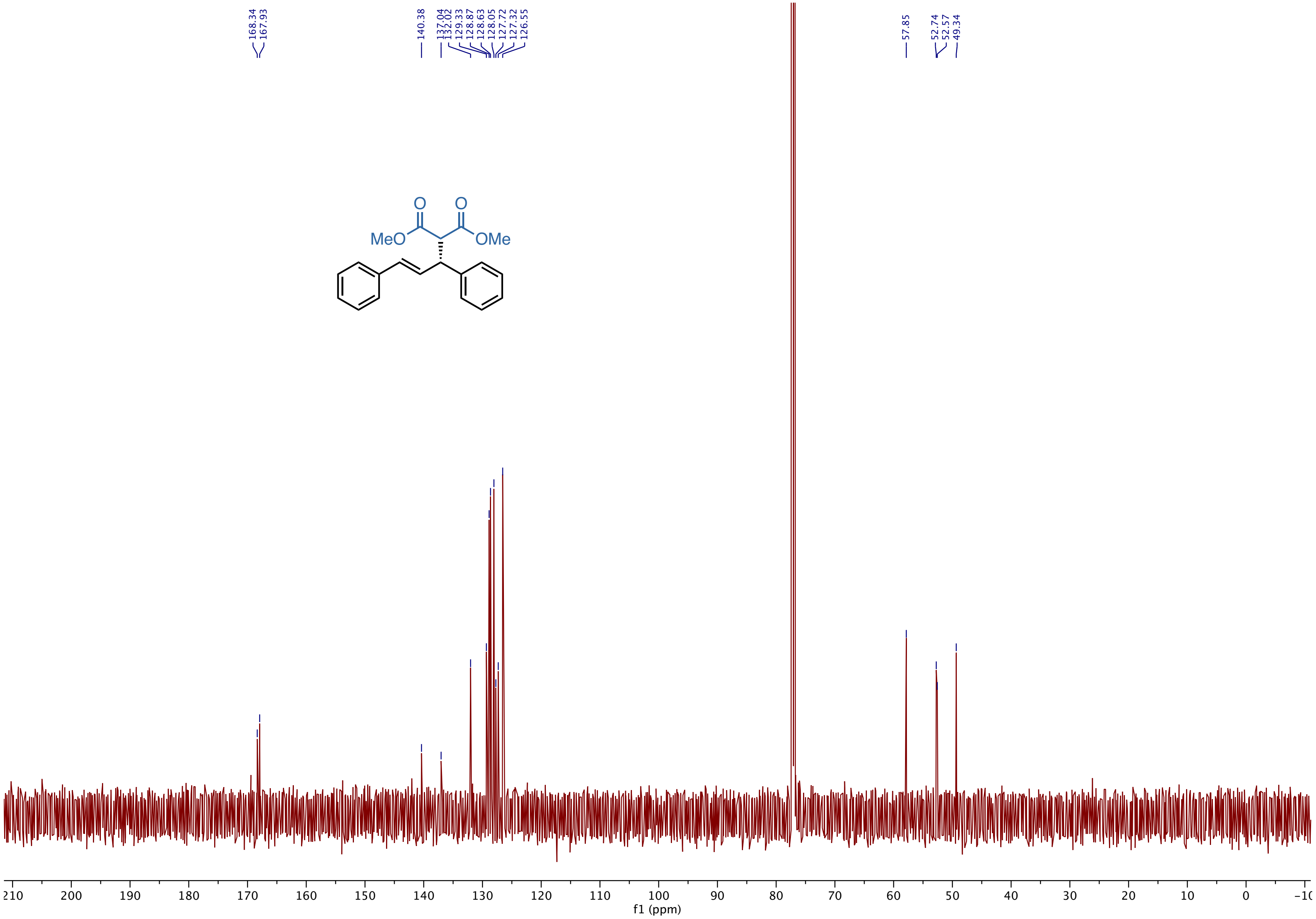

^{1}H NMR (400 MHz, $CDCl_3$): Dimethyl (*S,E*)-2-(1,3-diphenylallyl)-2-methylmalonate (3b)

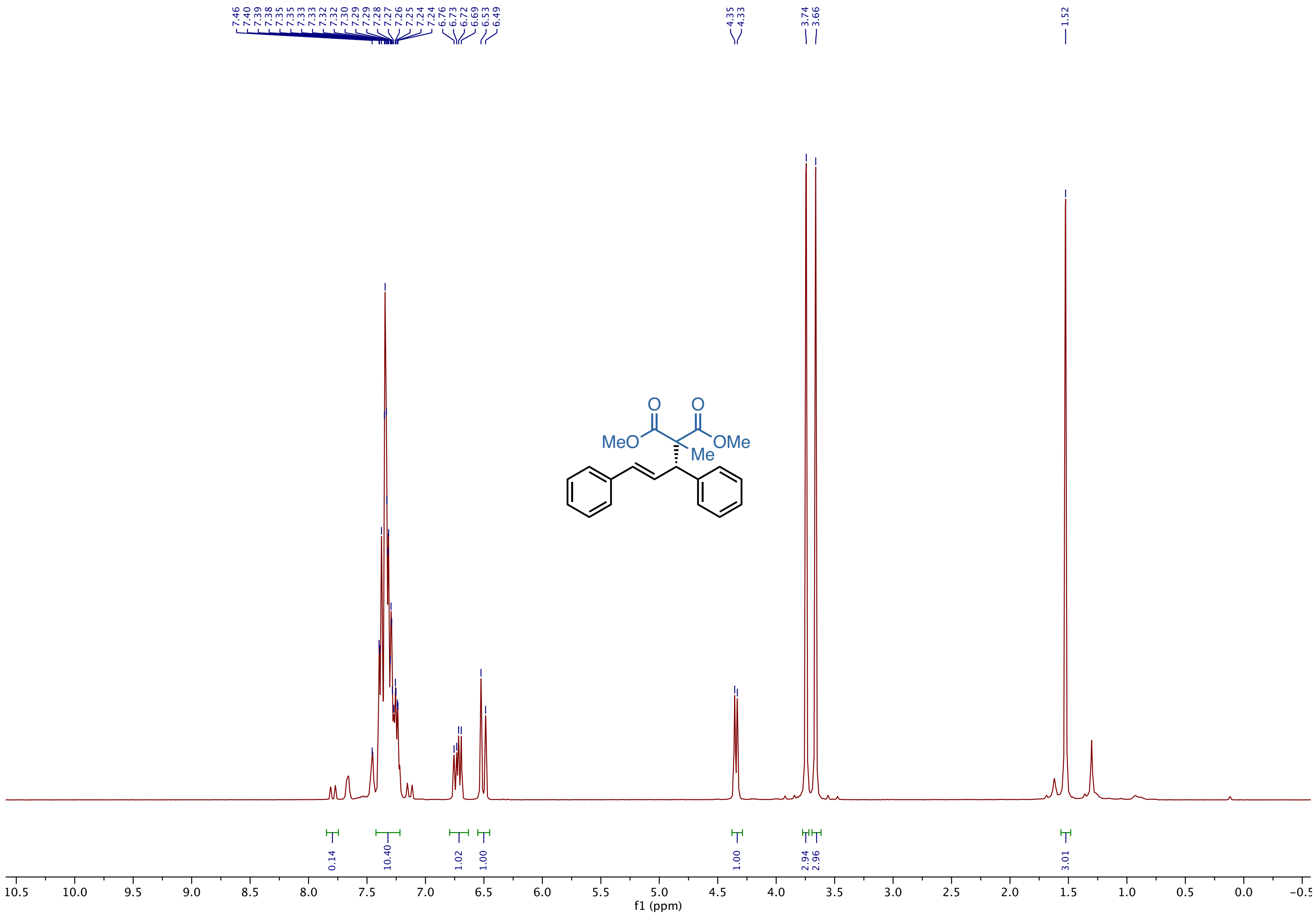

^{13}C NMR (101 MHz, $CDCl_3$): Dimethyl (*S,E*)-2-(1,3-diphenylallyl)-2-methylmalonate (3b)

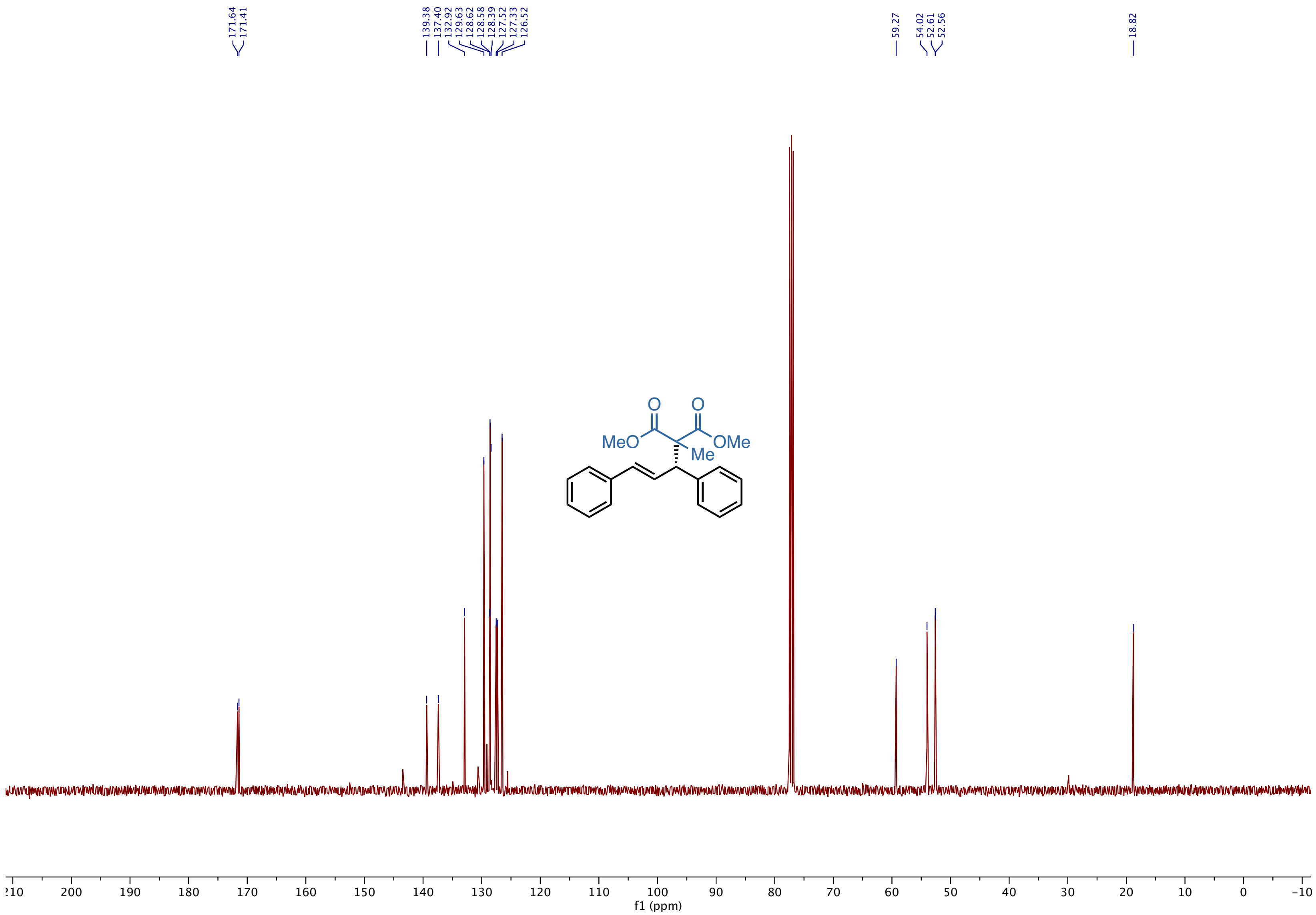

^1H NMR (700 MHz, $CDCl_3$): (*R,E*)-3-(1,3-diphenylallyl)Pentane-2,4-dione (3c)

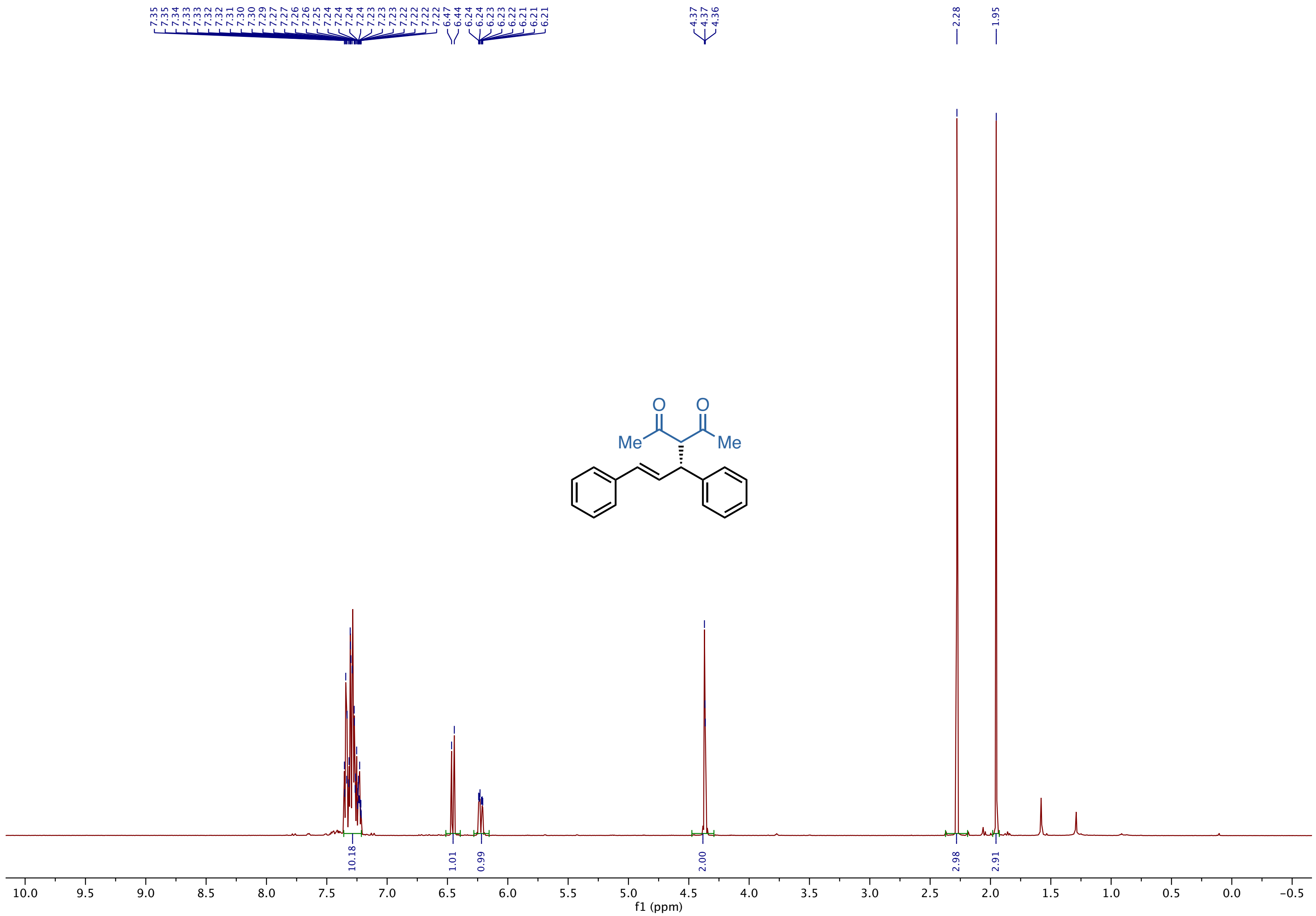

^{13}C NMR (176 MHz, $CDCl_3$): (*R,E*)-3-(1,3-diphenylallyl)Pentane-2,4-dione (3c)

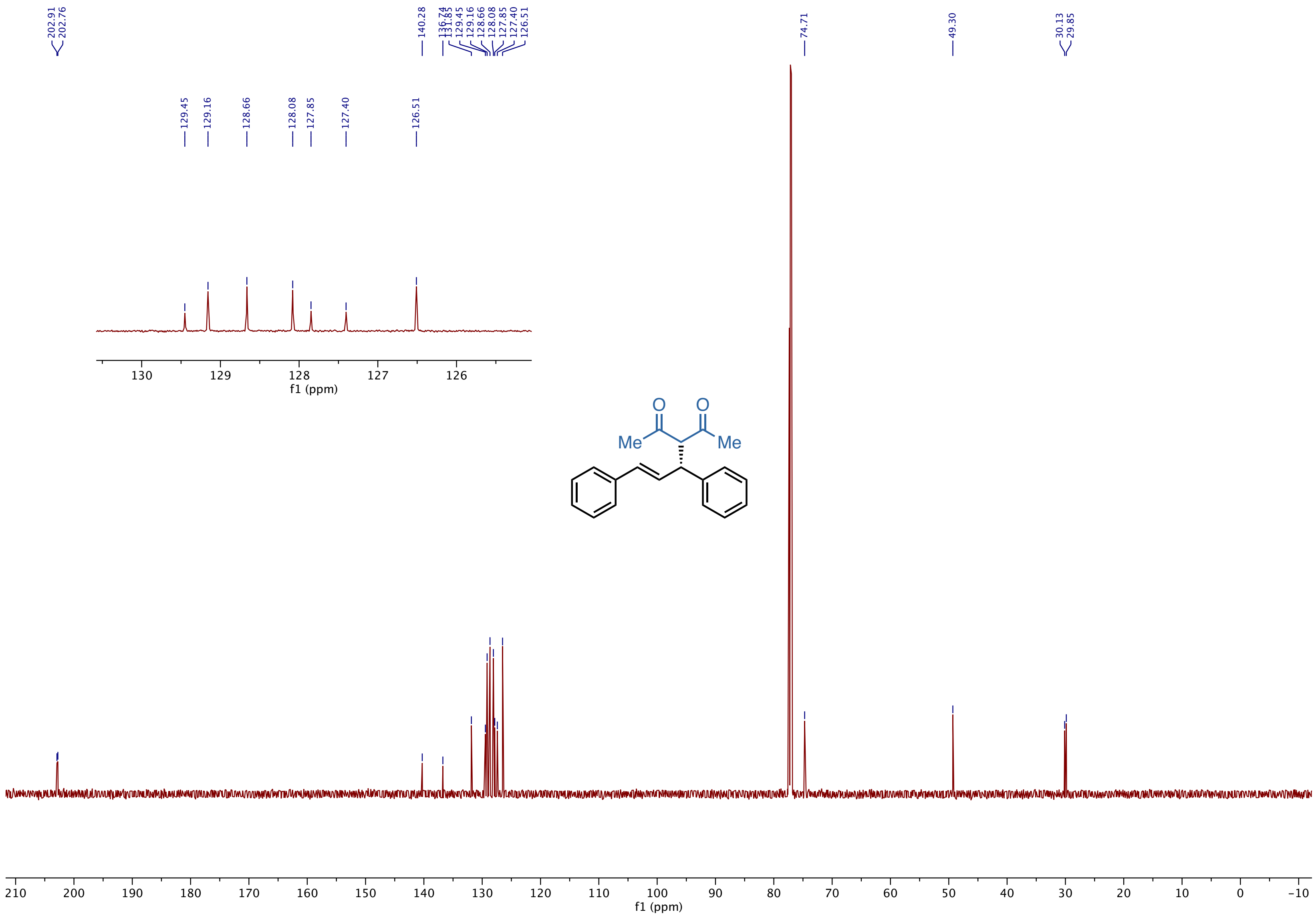

^{1}H NMR (500 MHz, $CDCl_3$): (*S,E*)-(4-methyl-4-nitropent-1-ene-1,3-diyl)Dibenzene (3d)

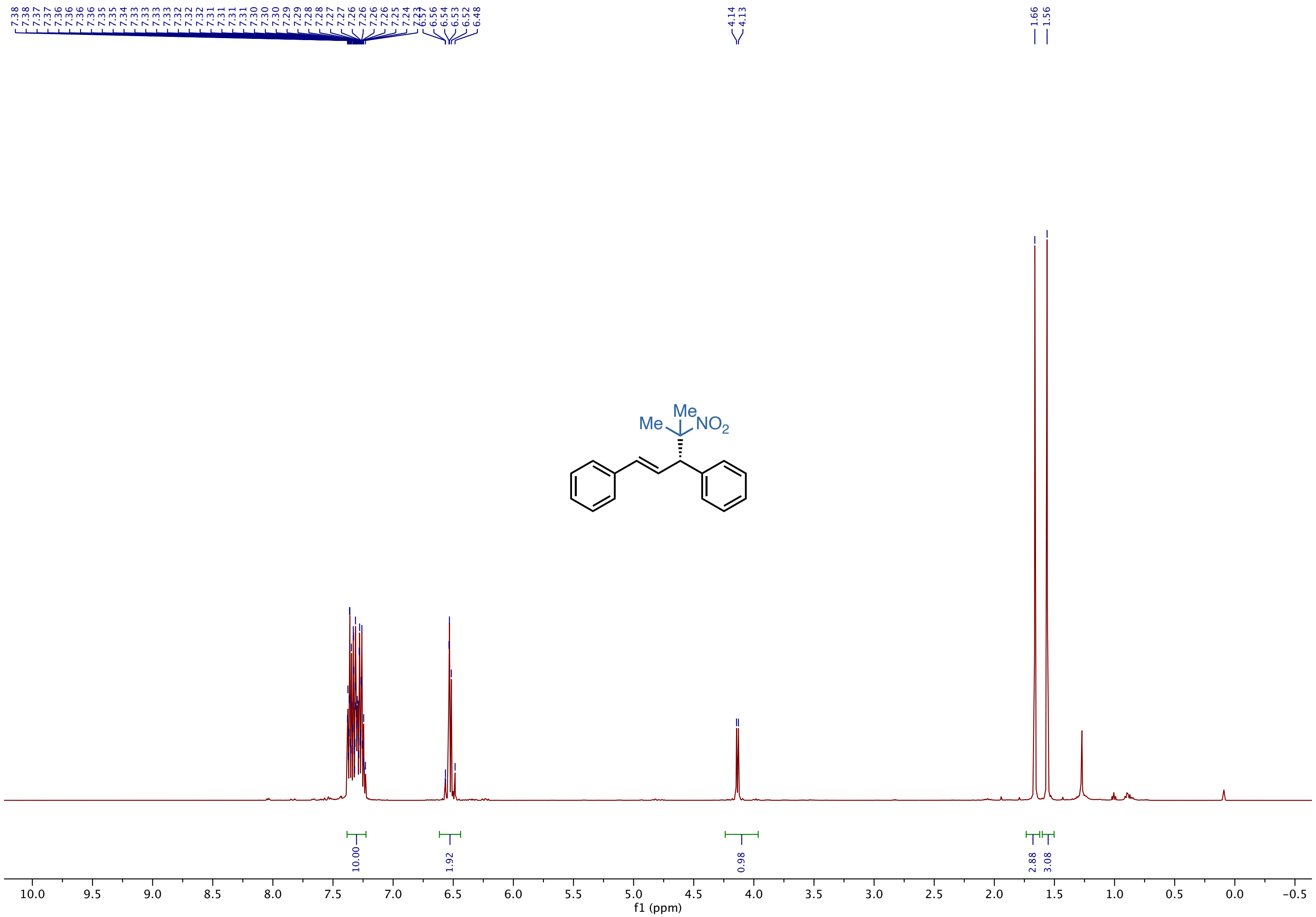

^{13}C NMR (126 MHz, $CDCl_3$): (*S,E*)-(4-methyl-4-nitropent-1-ene-1,3-diyl)Dibenzene (3d)

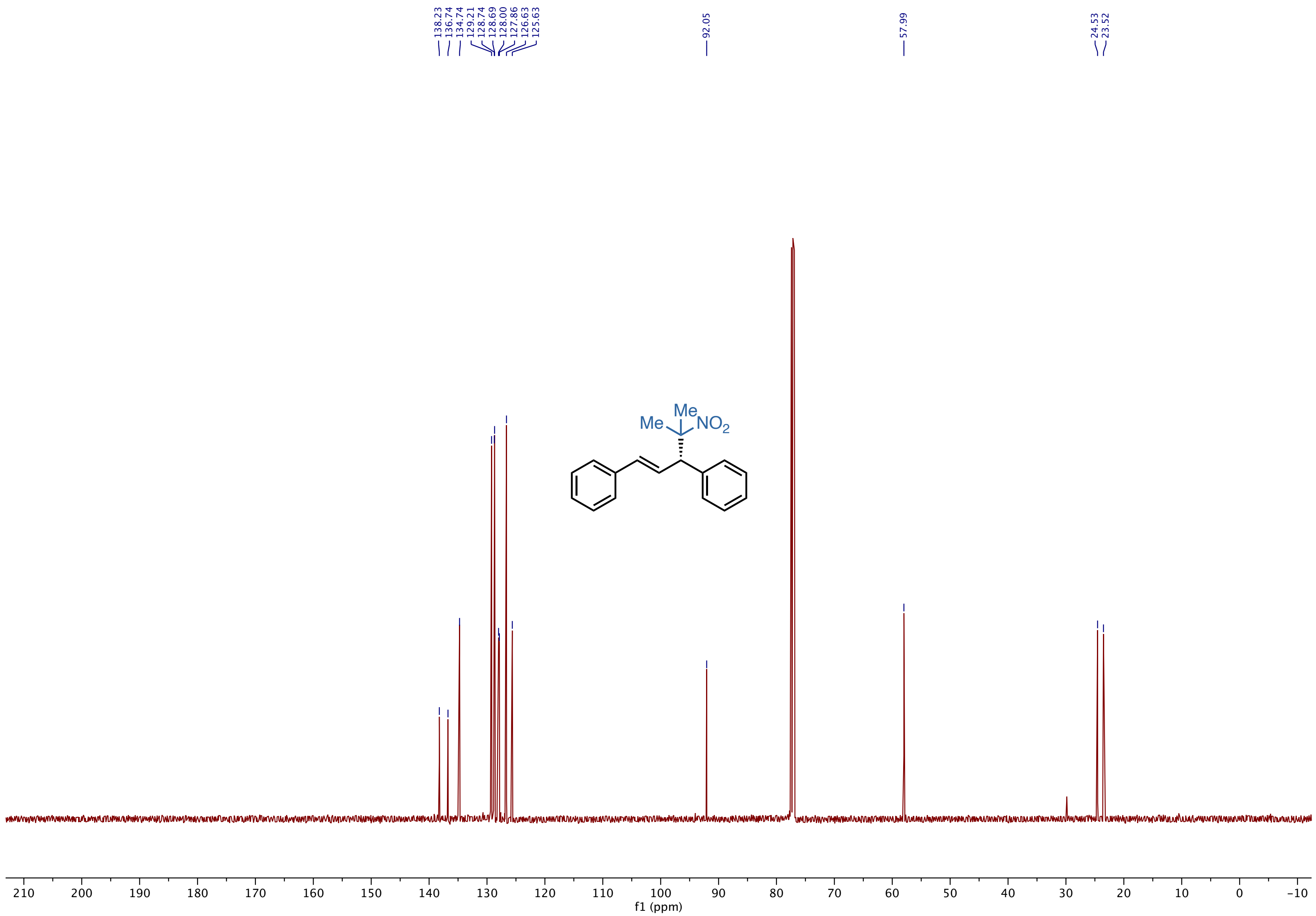

^{1}H NMR (400 MHz, $CDCl_3$): ((*S*,*E*)-4-Nitropent-1-ene-1,3-diyl)dibenzene (3ea)

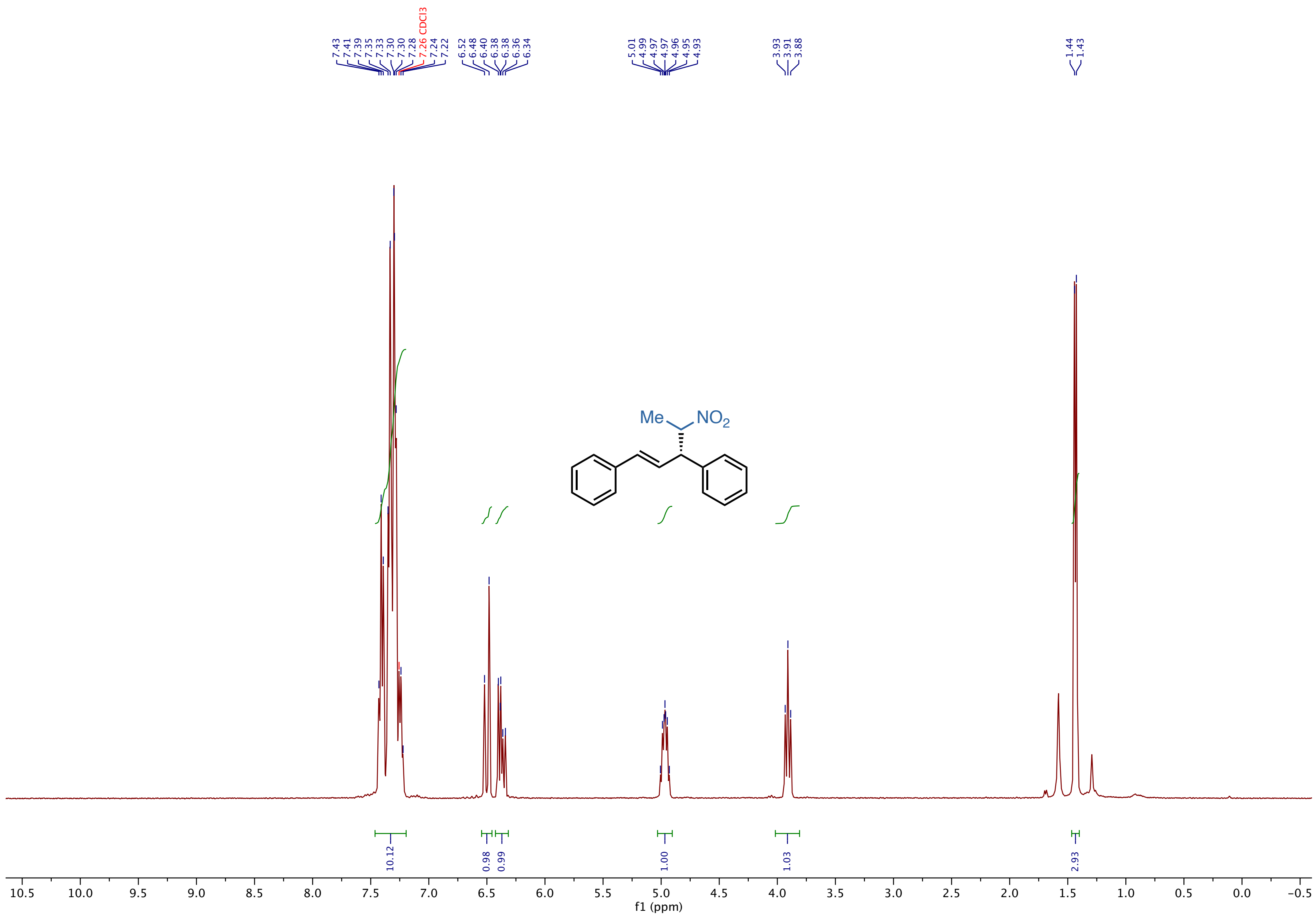

^{13}C NMR (101 MHz, $CDCl_3$): ((*S*,*E*)-4-Nitropent-1-ene-1,3-diyl)dibenzene (3ea)

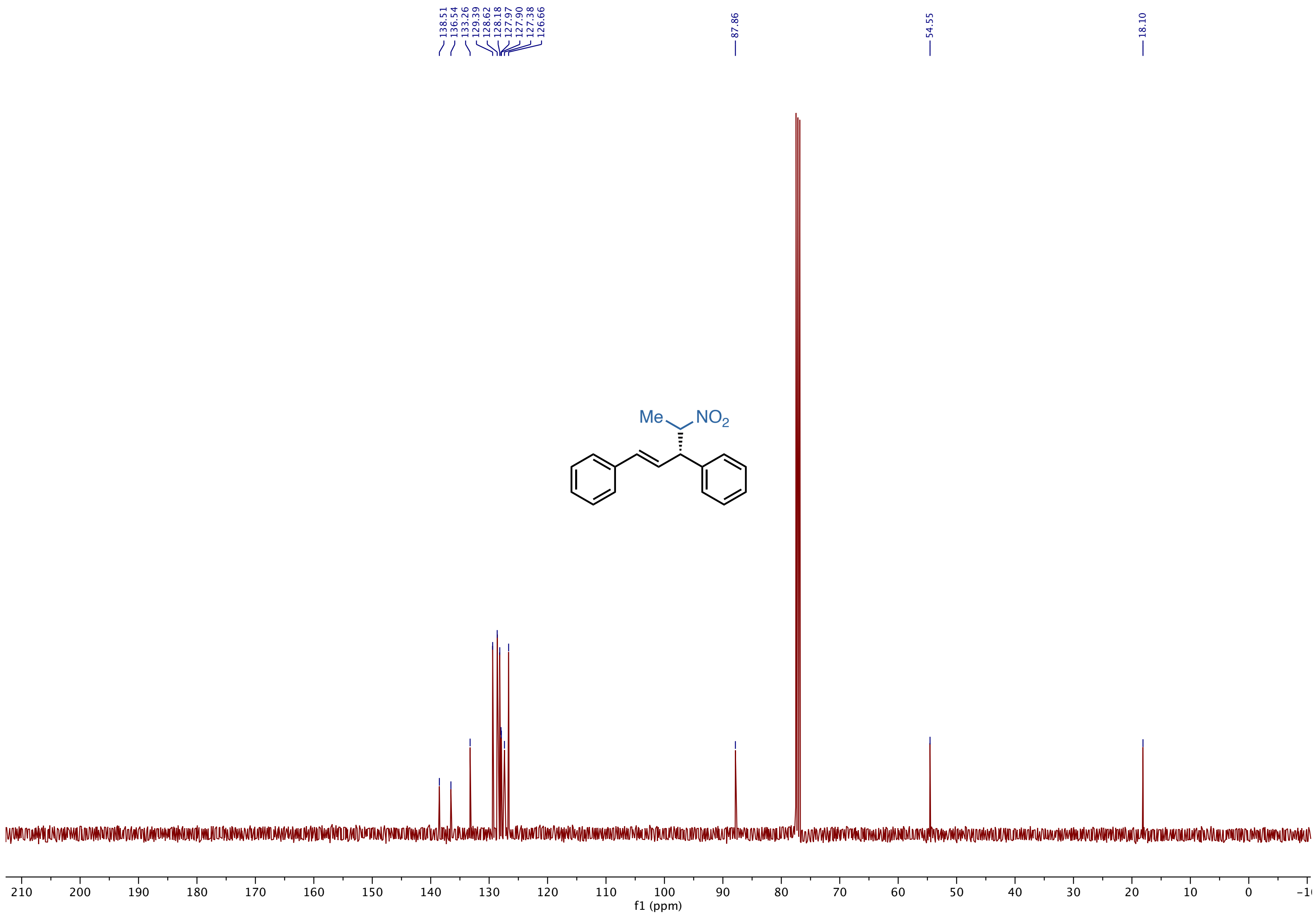

^{1}H NMR (400 MHz, $CDCl_3$): ((*S*,*E*)-4-Nitropent-1-ene-1,3-diyl)dibenzene (3eb)

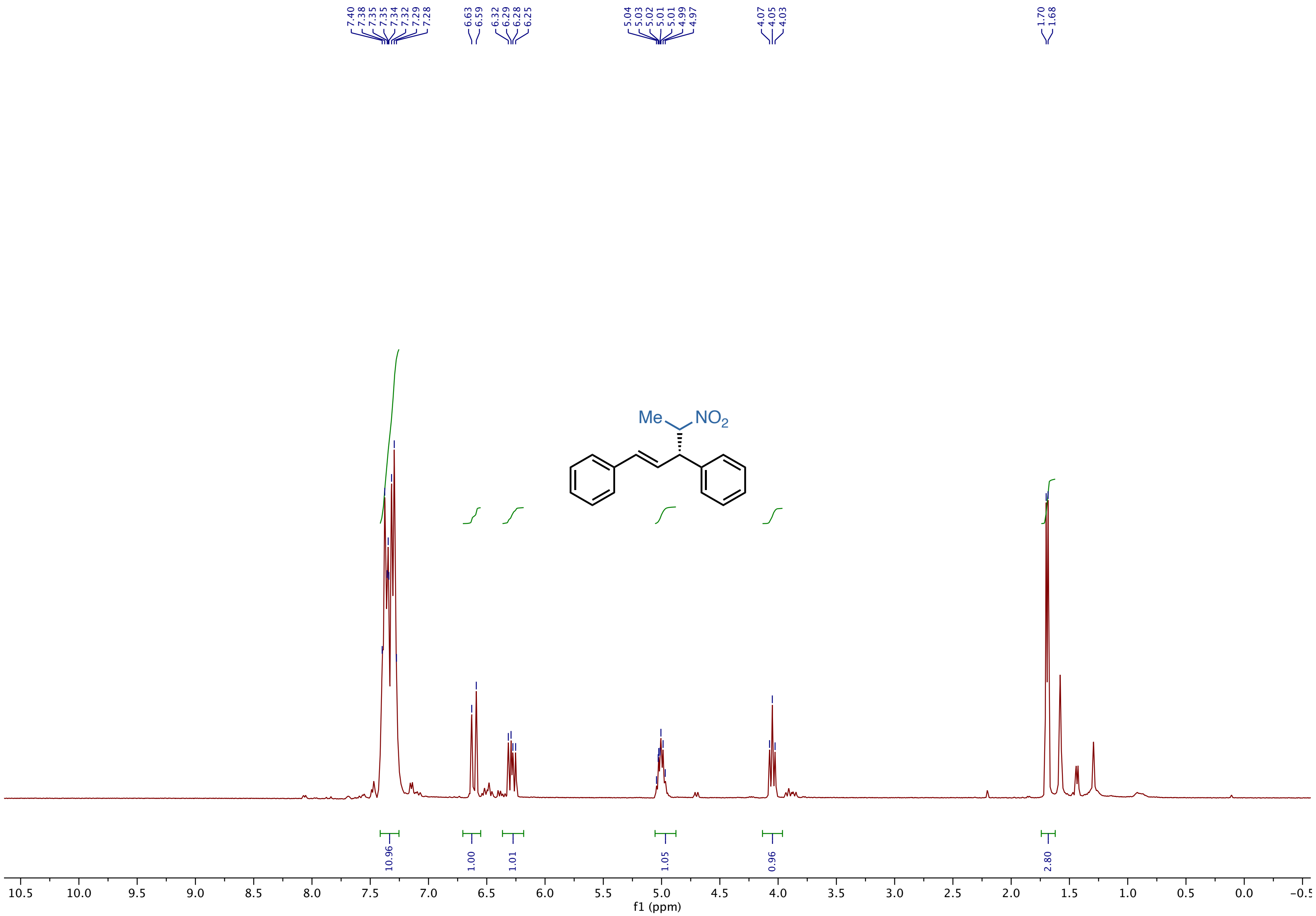

^{13}C NMR (101 MHz, $CDCl_3$): ((*S*,*E*)-4-Nitropent-1-ene-1,3-diyl)dibenzene (3eb)

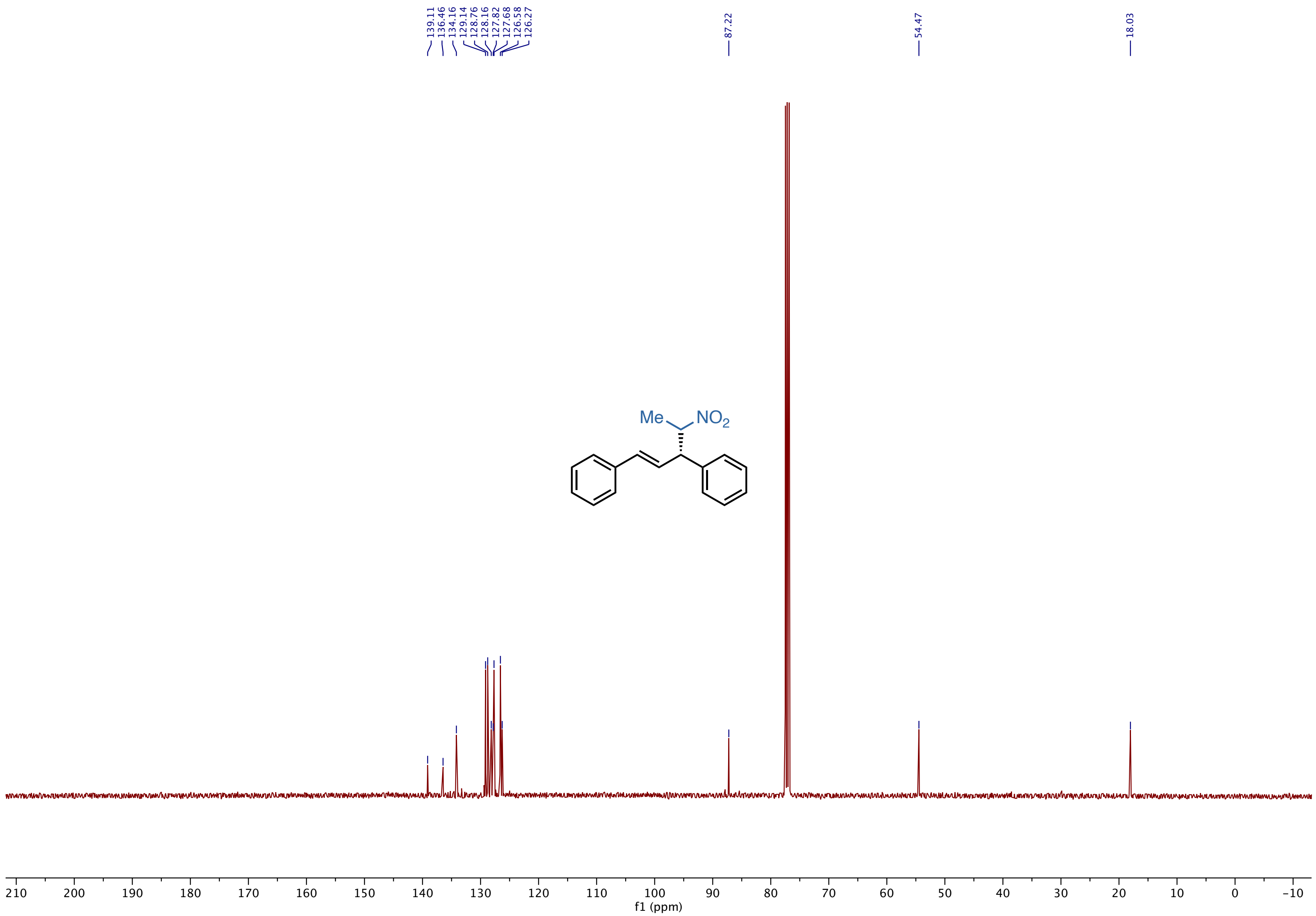

^{1}H NMR (400 MHz, $CDCl_3$): Methyl (*S,E*)-2-((diphenylmethylene)amino)-3,5-diphenylpent-4-enoate (3fa)

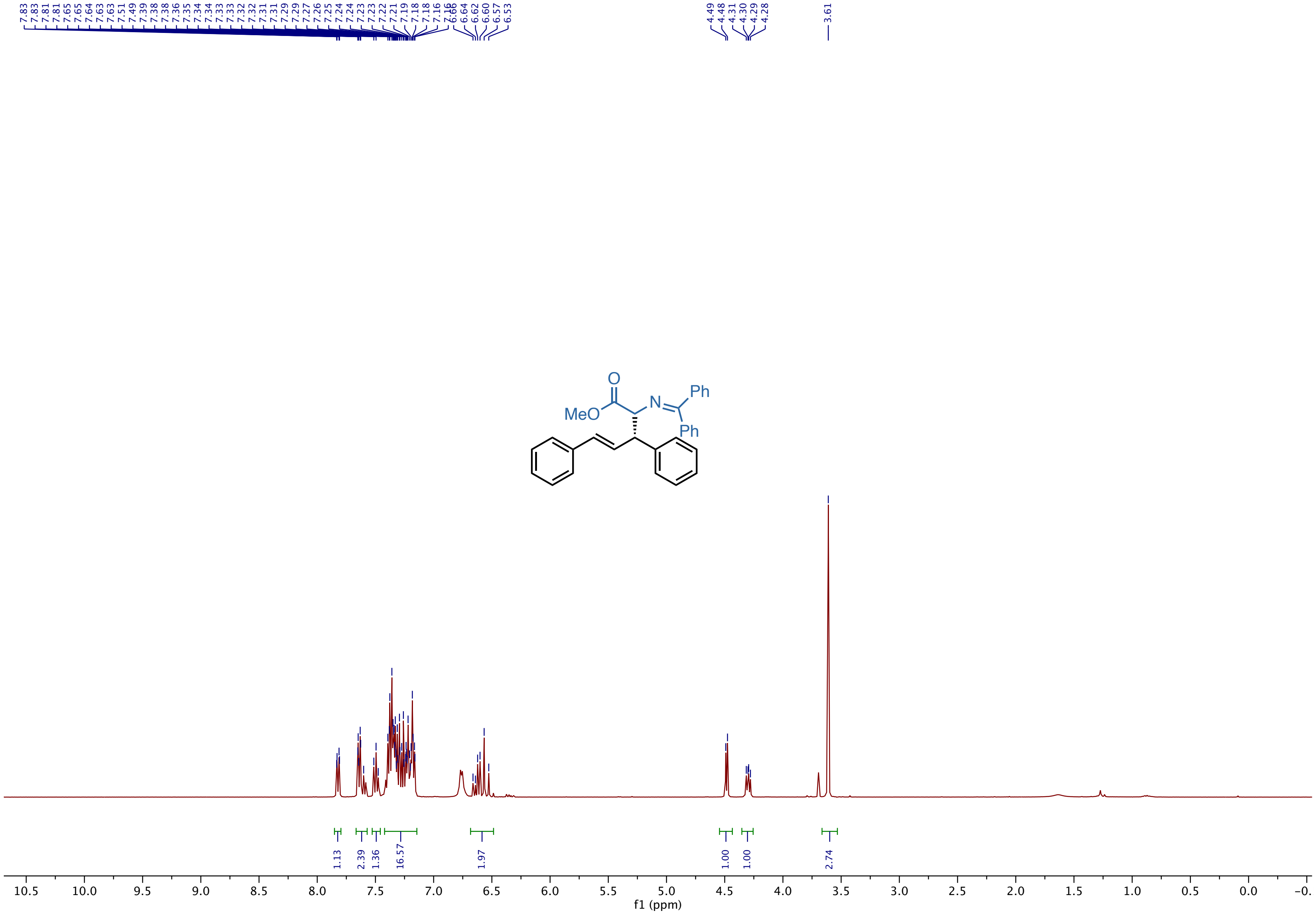

^{13}C NMR (101 MHz, $CDCl_3$): Methyl (*S,E*)-2-((diphenylmethylene)amino)-3,5-diphenylpent-4-enoate (3fa)

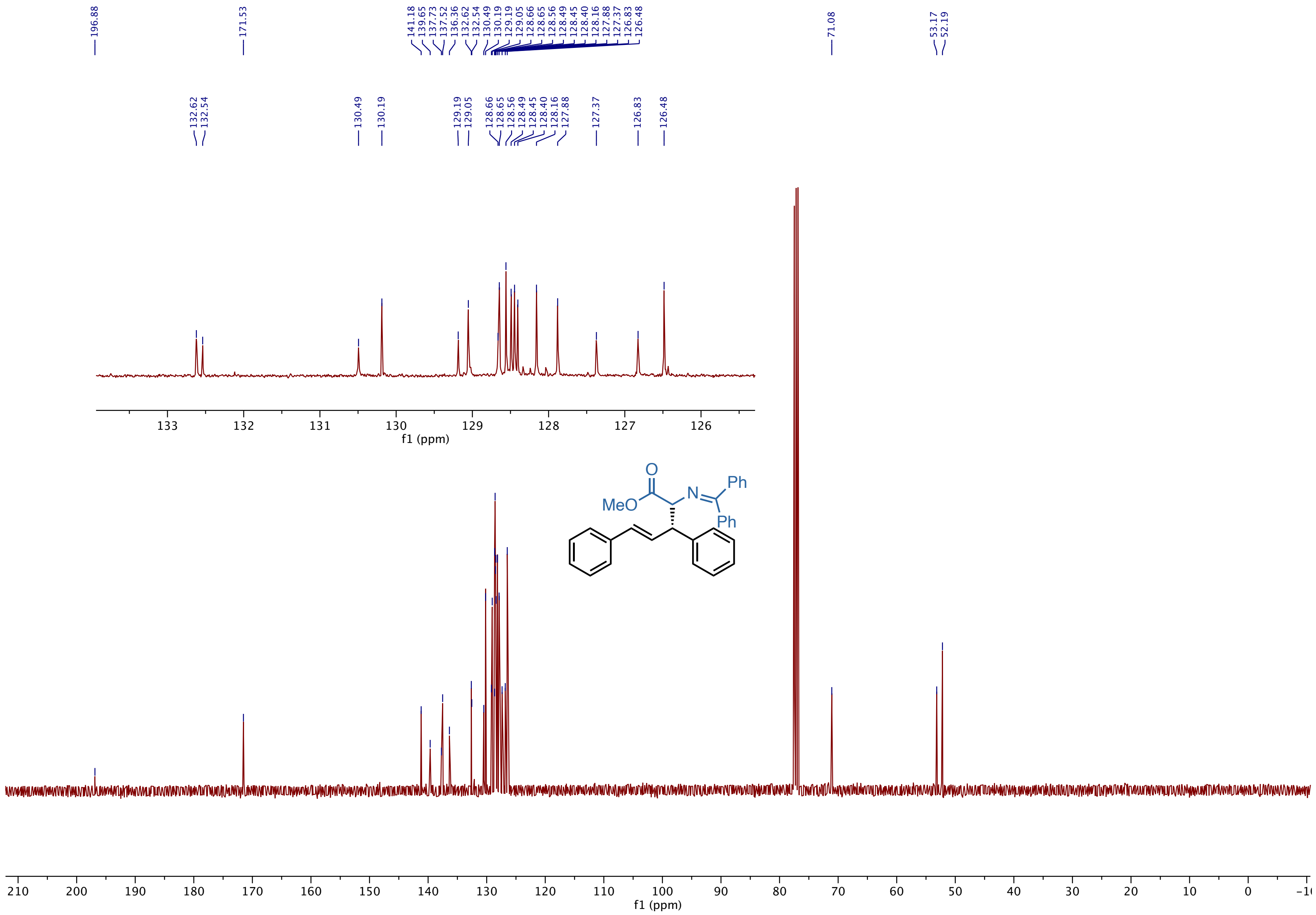

^{1}H NMR (400 MHz, $CDCl_3$): Methyl (*S,E*)-2-((diphenylmethylene)amino)-3,5-diphenylpent-4-enoate (3fb)

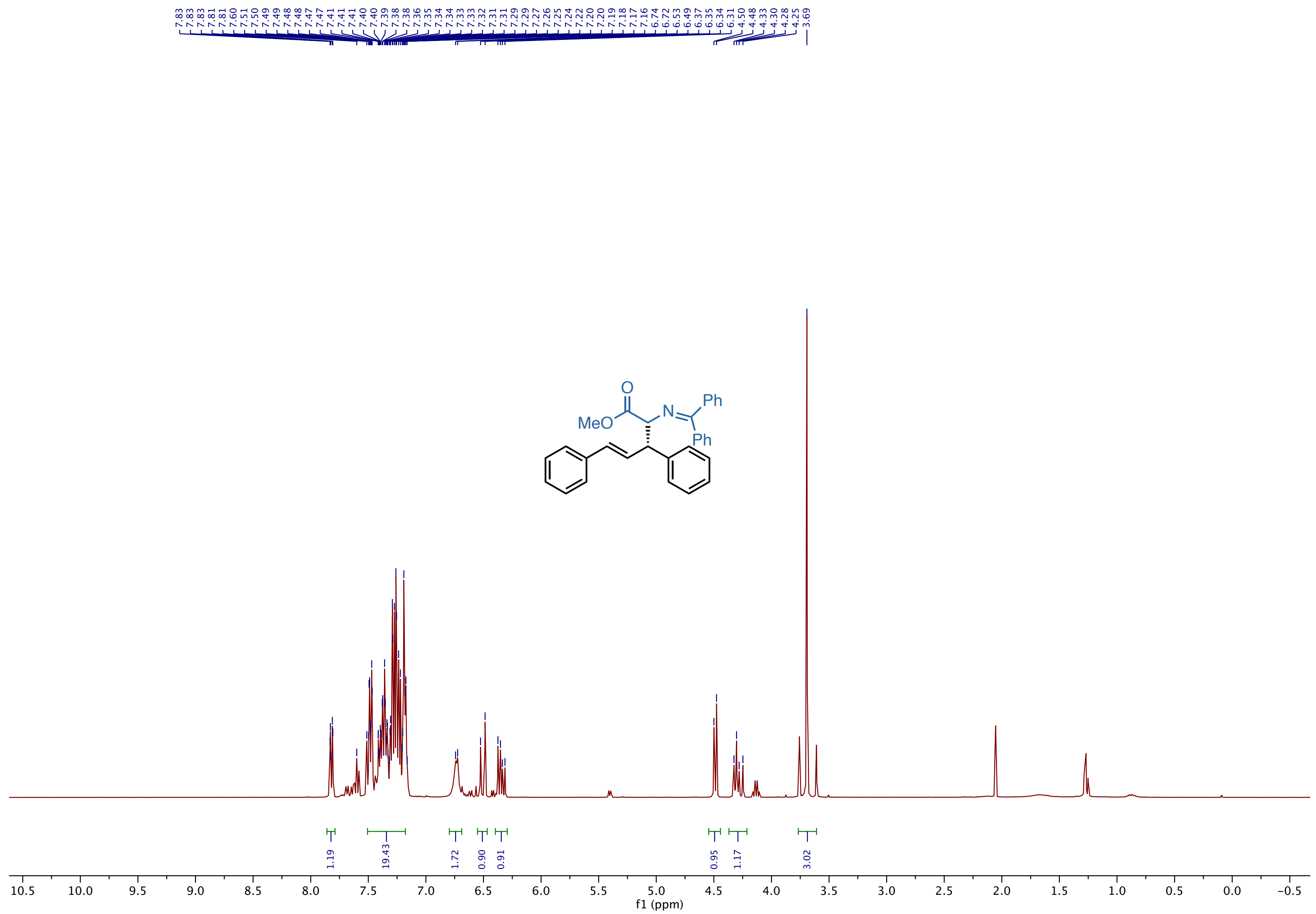

^{13}C NMR (101 MHz, $CDCl_3$): Methyl (*S,E*)-2-((diphenylmethylene)amino)-3,5-diphenylpent-4-enoate (3fb)

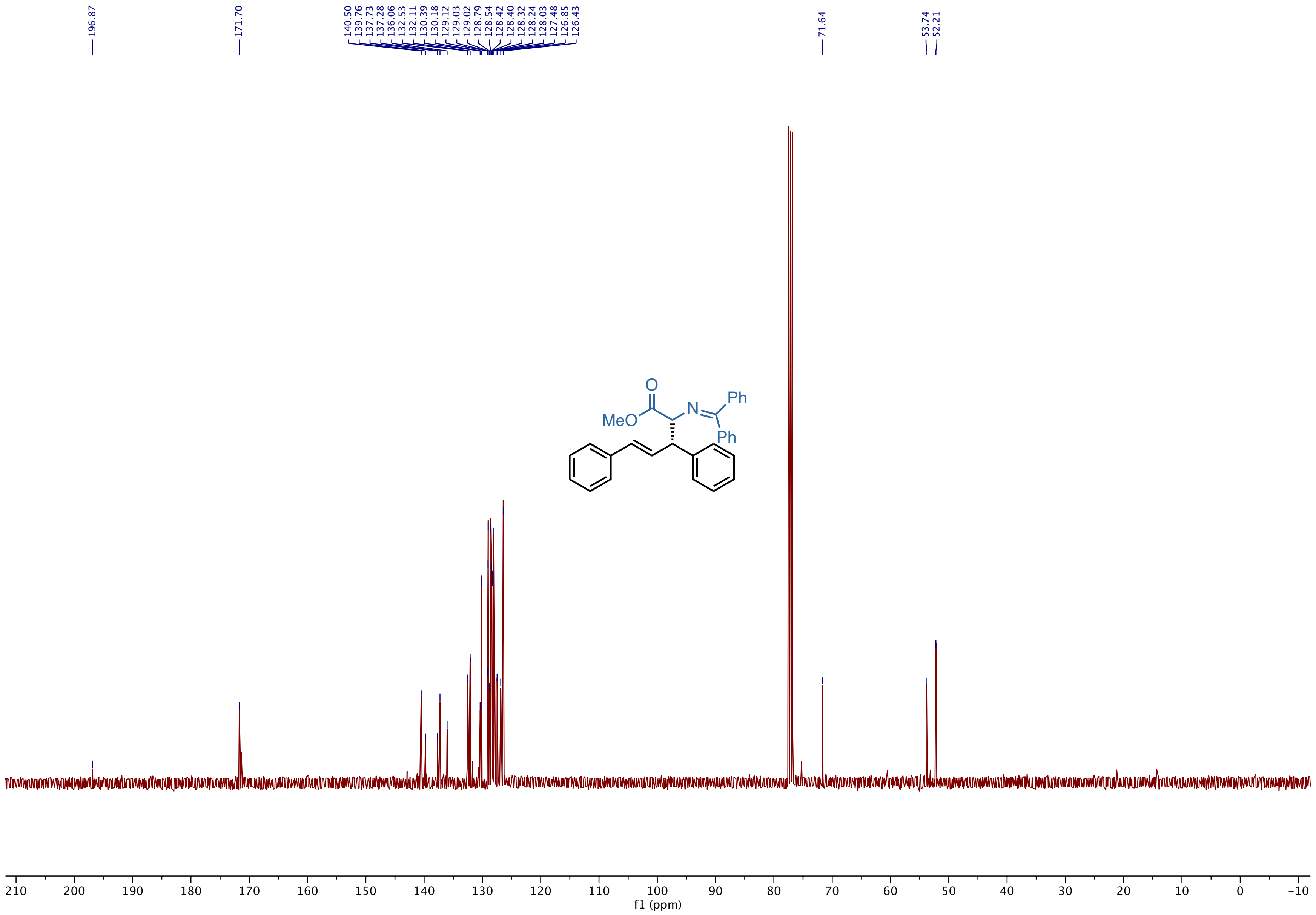

^{1}H NMR (400 MHz, $CDCl_3$): Methyl (*S*,*E*)-1-(1,3-diphenylallyl)-2-oxocyclohexane-1-carboxylate (3ga)

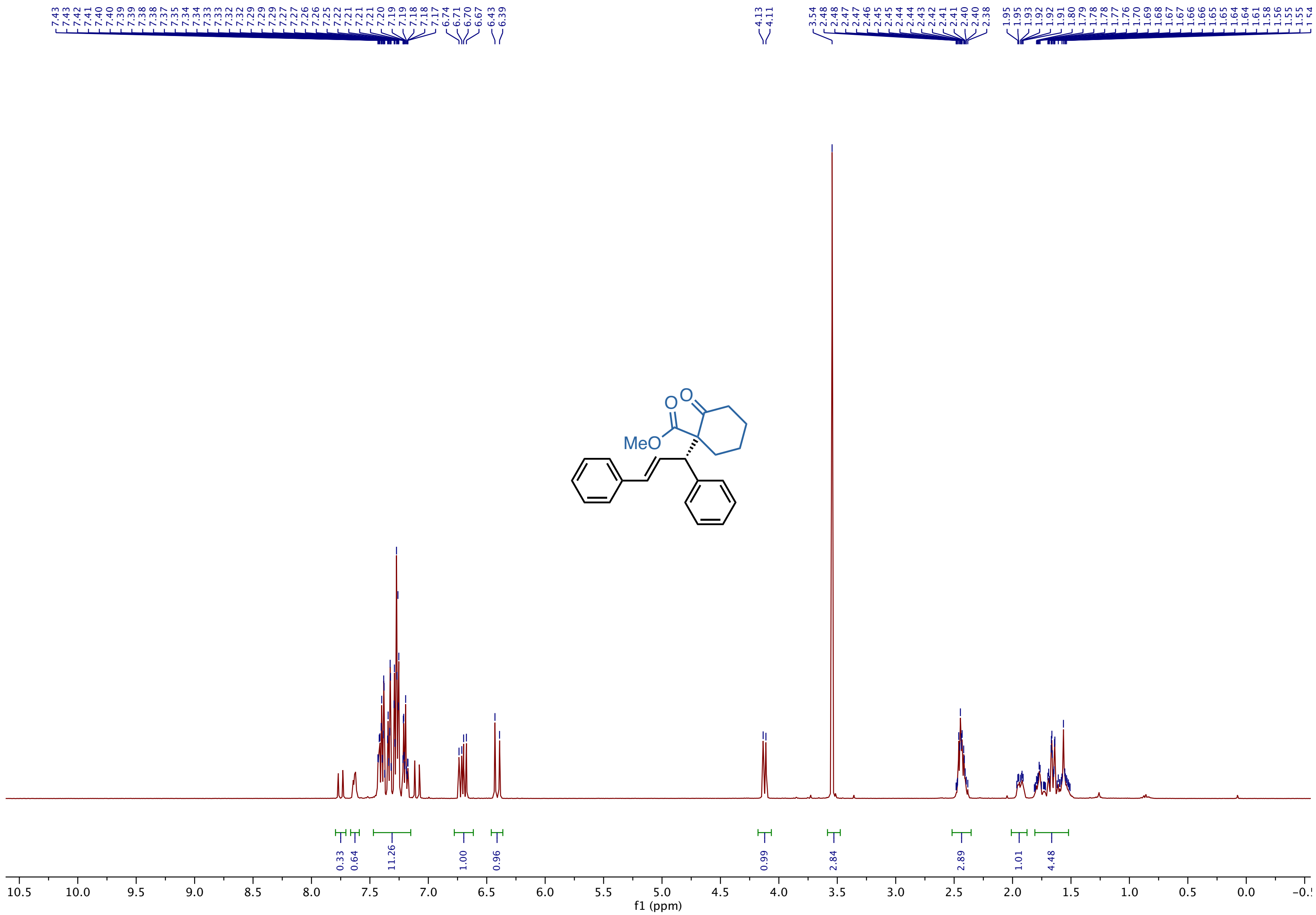

^{13}C NMR (101 MHz, $CDCl_3$): Methyl (*S*,*E*)-1-(1,3-diphenylallyl)-2-oxocyclohexane-1-carboxylate (3ga)

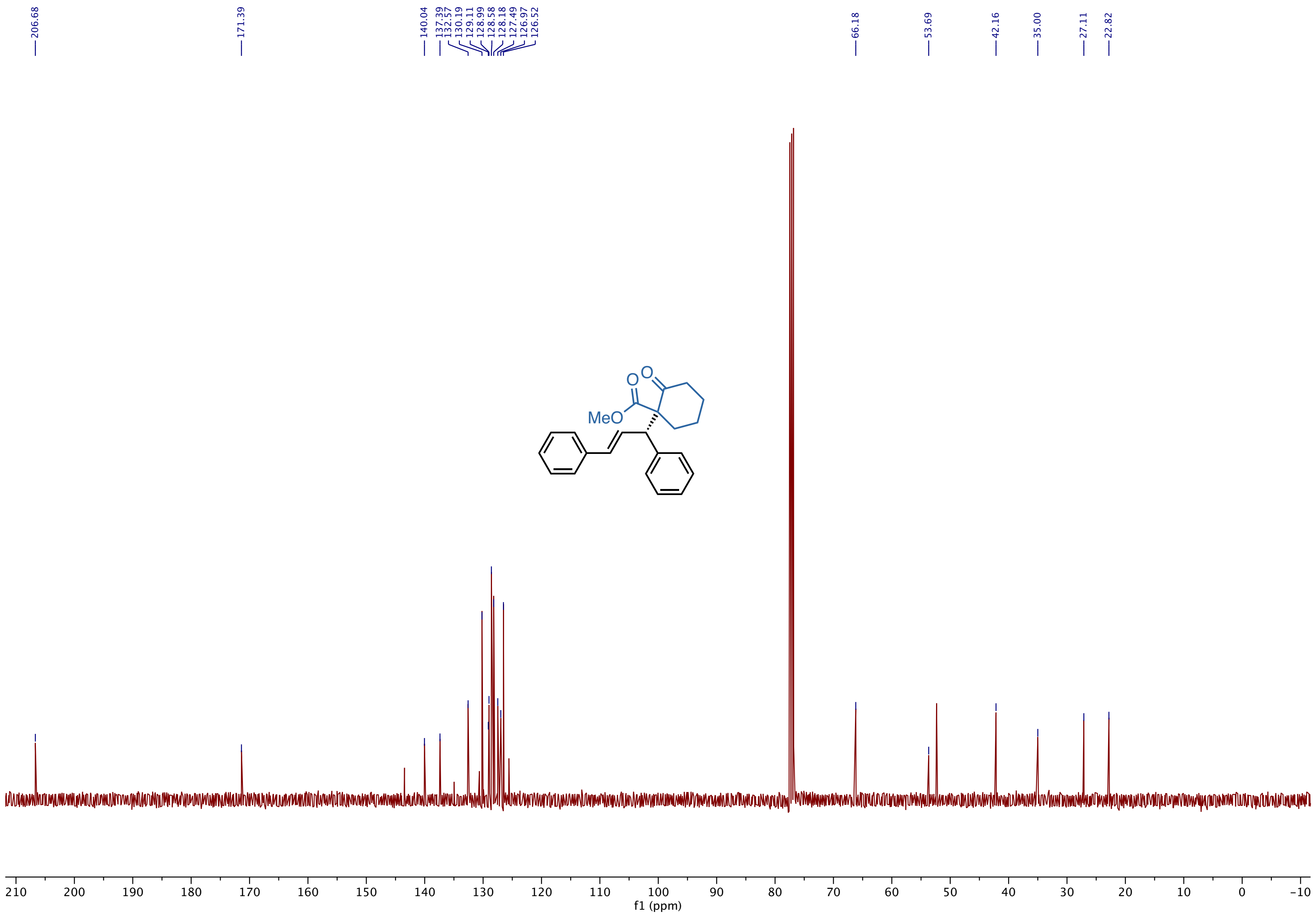

^{1}H NMR (400 MHz, $CDCl_3$): Methyl (*S*,*E*)-1-(1,3-diphenylallyl)-2-oxocyclohexane-1-carboxylate (3gb)

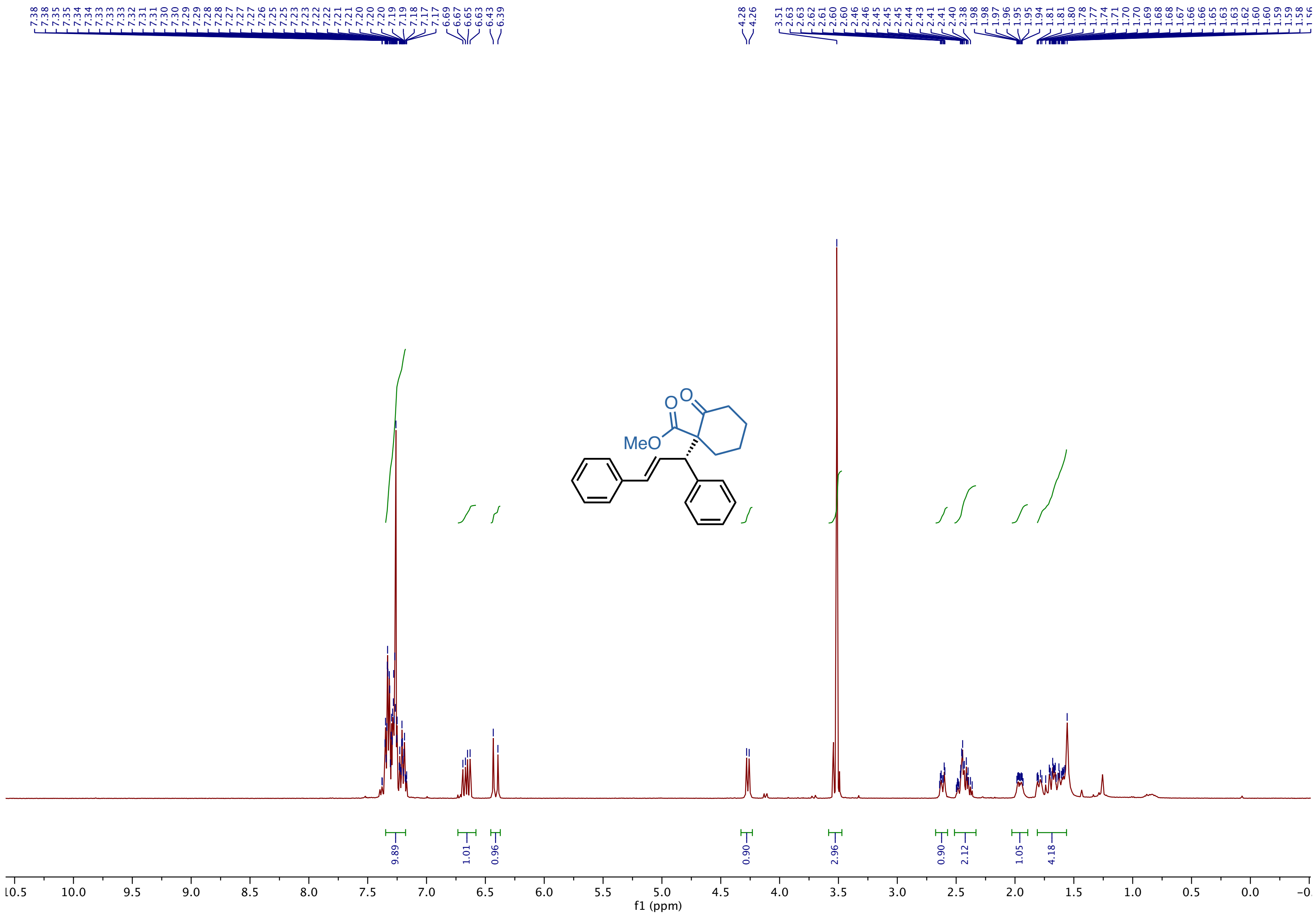

^{13}C NMR (101 MHz, $CDCl_3$): Methyl (*S*,*E*)-1-(1,3-diphenylallyl)-2-oxocyclohexane-1-carboxylate (3gb)

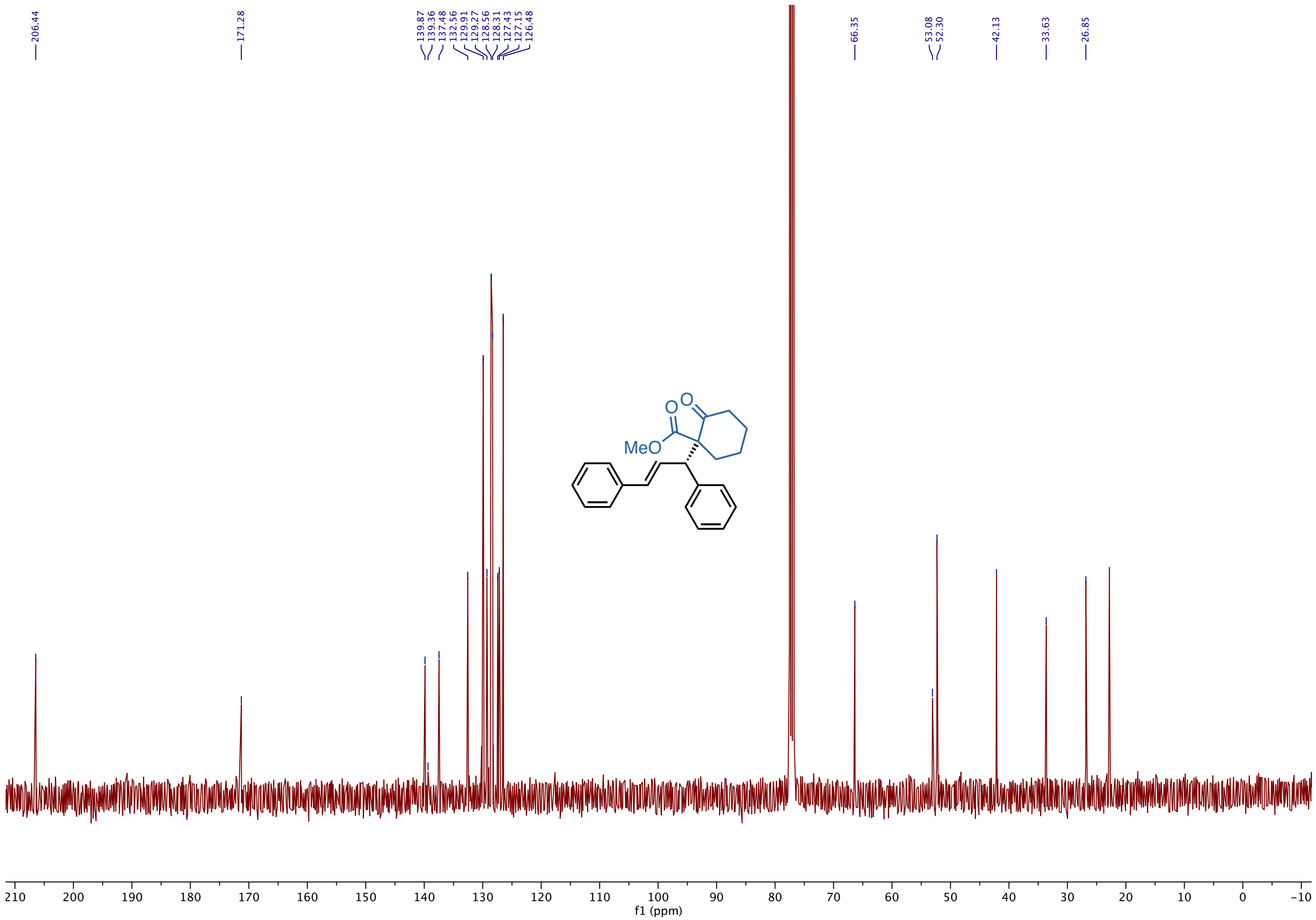

^{1}H NMR (500 MHz, $CDCl_3$): Methyl (*S*,*E*)-2-(1,3-diphenylallyl)-1-oxo-1,2,3,4-tetrahydronaphthalene-2-carboxylate (3ha)

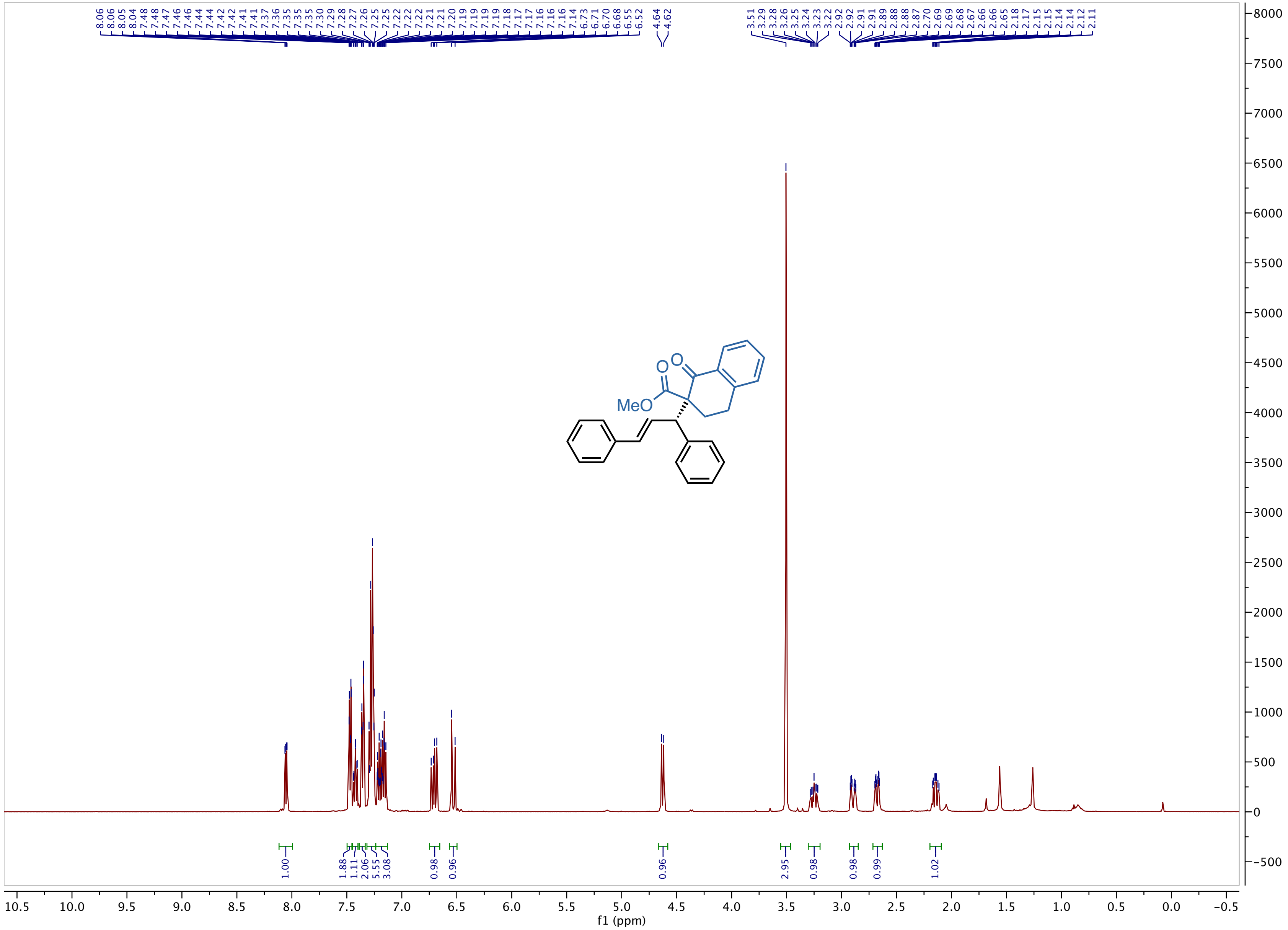

^{13}C NMR (126 MHz, $CDCl_3$): Methyl (*S*,*E*)-2-(1,3-diphenylallyl)-1-oxo-1,2,3,4-tetrahydronaphthalene-2-carboxylate (3ha)

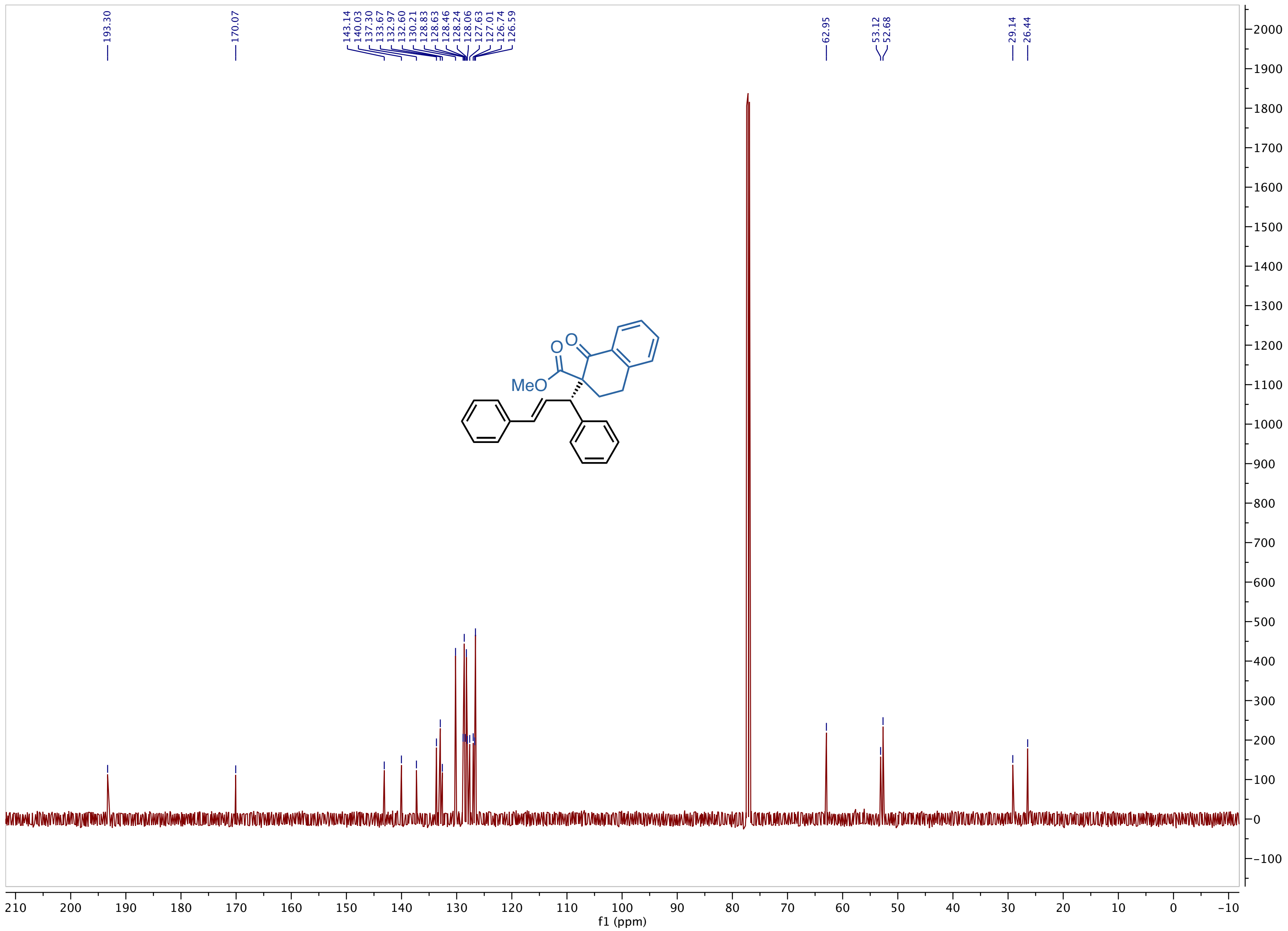

^{1}H NMR (500 MHz, $CDCl_3$): Methyl (*S*,*E*)-2-(1,3-diphenylallyl)-1-oxo-1,2,3,4-tetrahydronaphthalene-2-carboxylate (3hb)

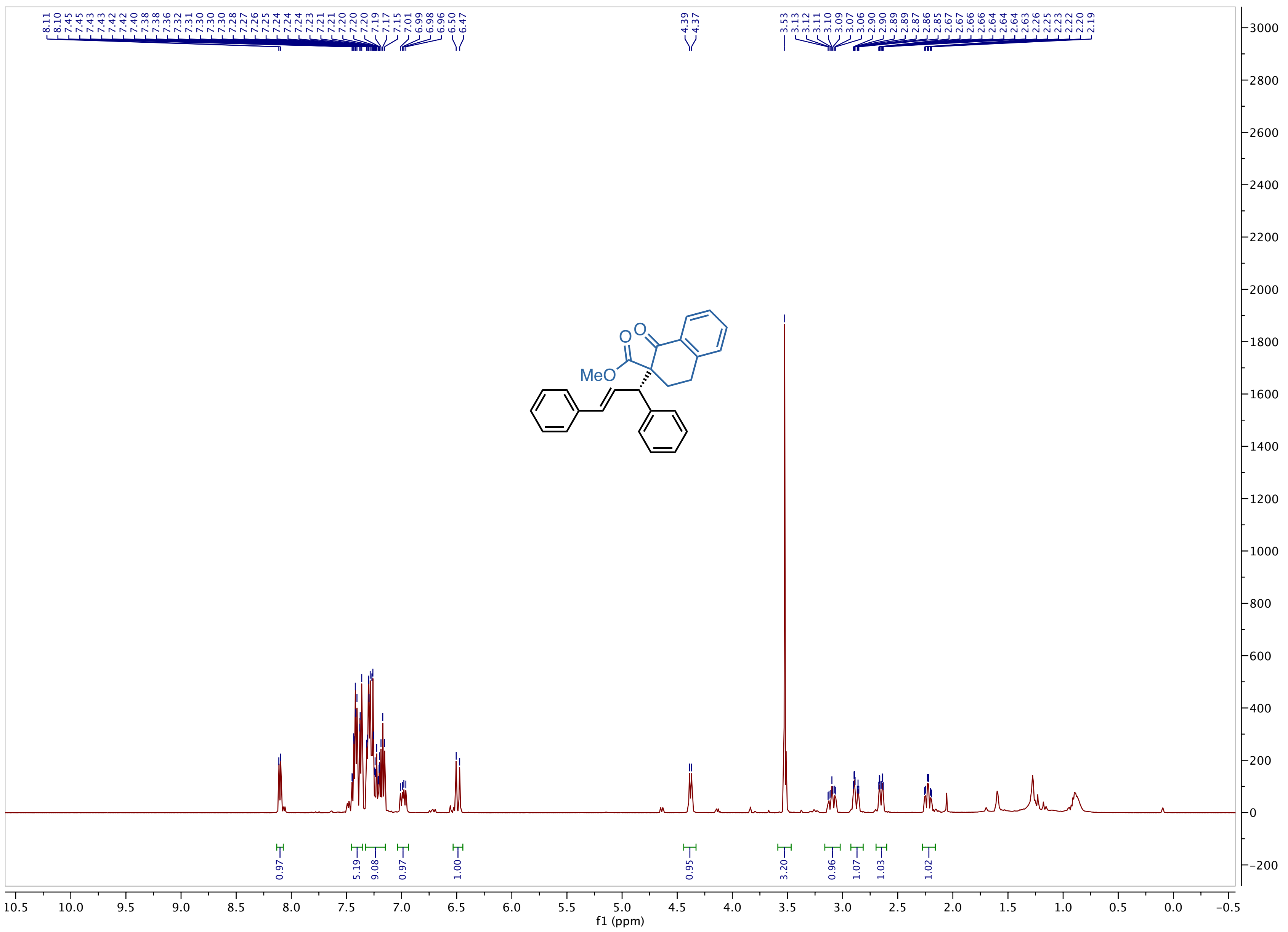

^{13}C NMR (126 MHz, $CDCl_3$): Methyl (*S*,*E*)-2-(1,3-diphenylallyl)-1-oxo-1,2,3,4-tetrahydronaphthalene-2-carboxylate (3hb)

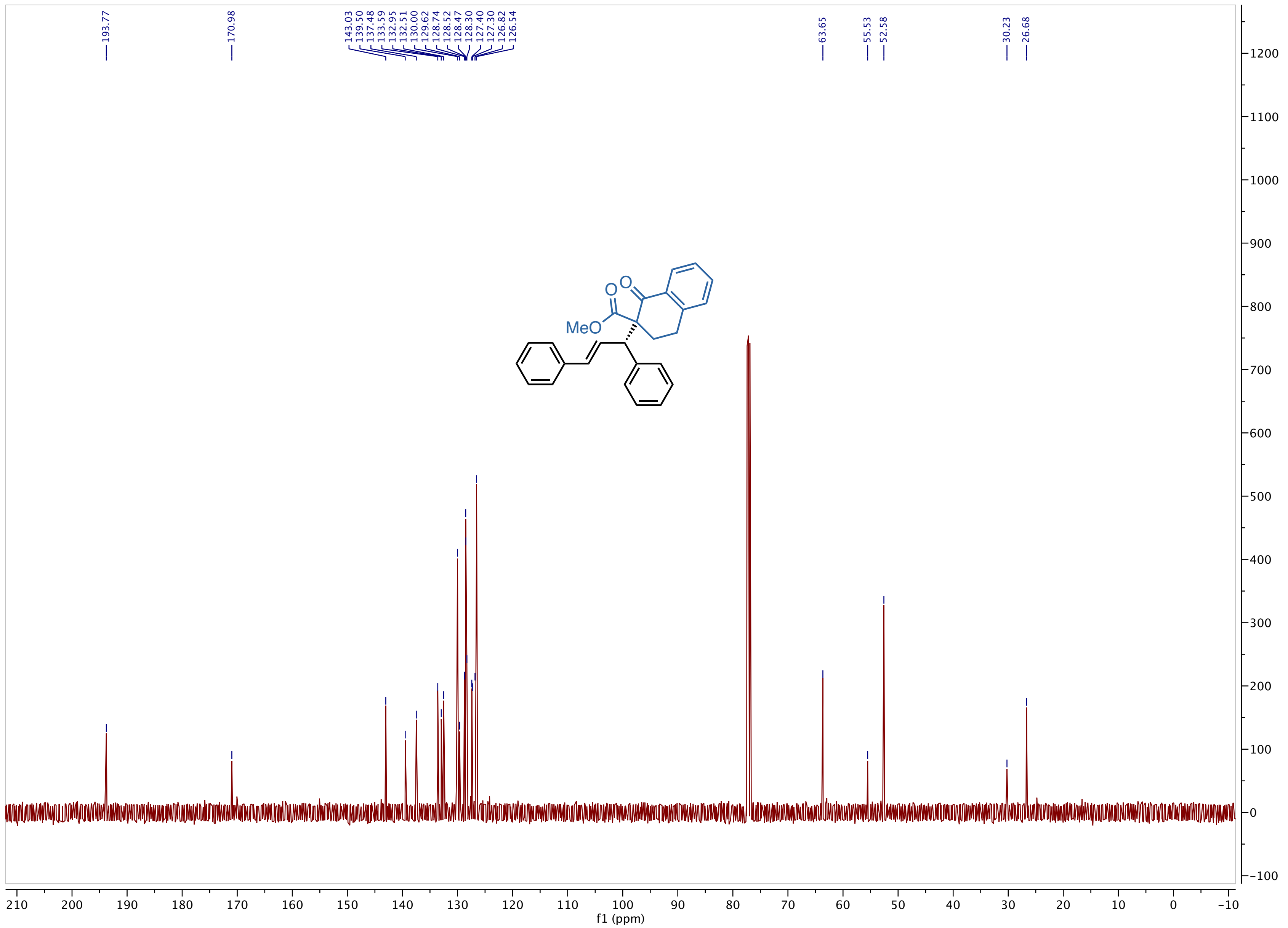

^{1}H NMR (400 MHz, CDCl$_3$): Ethyl (*S*,*E*)-2-ethyl-2-nitro-3,5-diphenylpent-4-enoate (3ia)

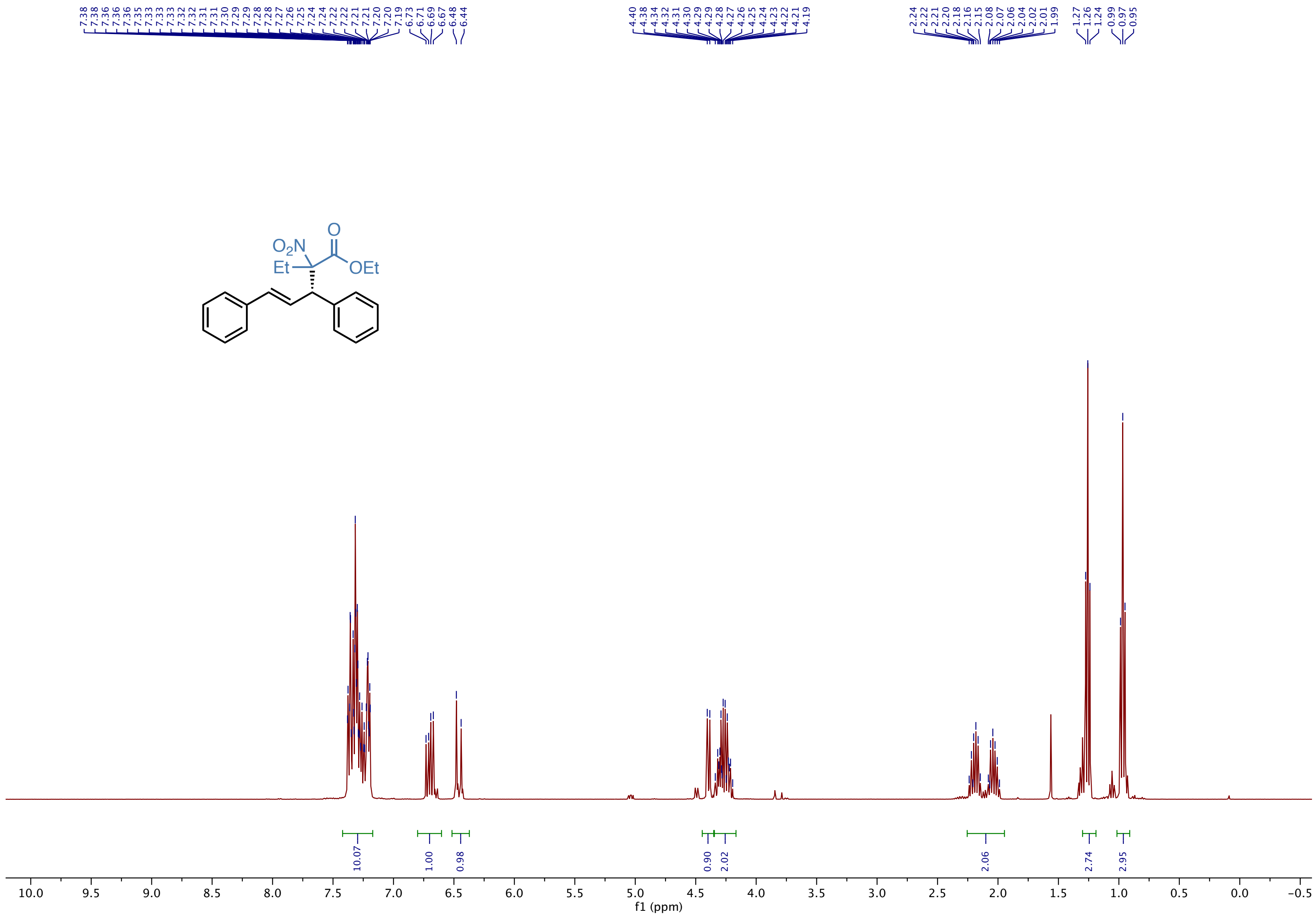

^{13}C NMR (101 MHz, $CDCl_3$): Ethyl (*S*,*E*)-2-ethyl-2-nitro-3,5-diphenylpent-4-enoate (3ia)

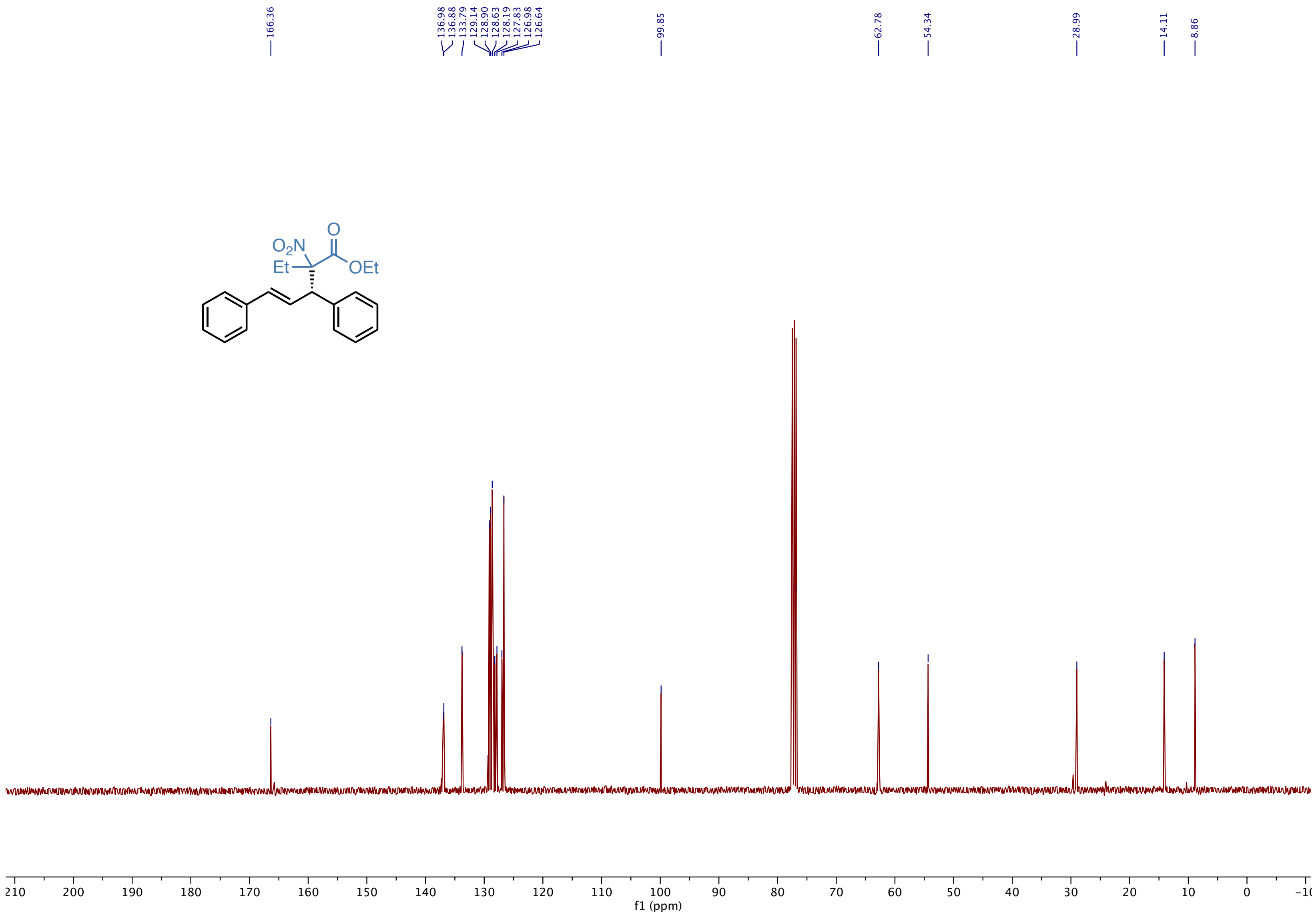

^{1}H NMR (400 MHz, $CDCl_3$): Ethyl (*S*,*E*)-2-ethyl-2-nitro-3,5-diphenylpent-4-enoate (3ib)

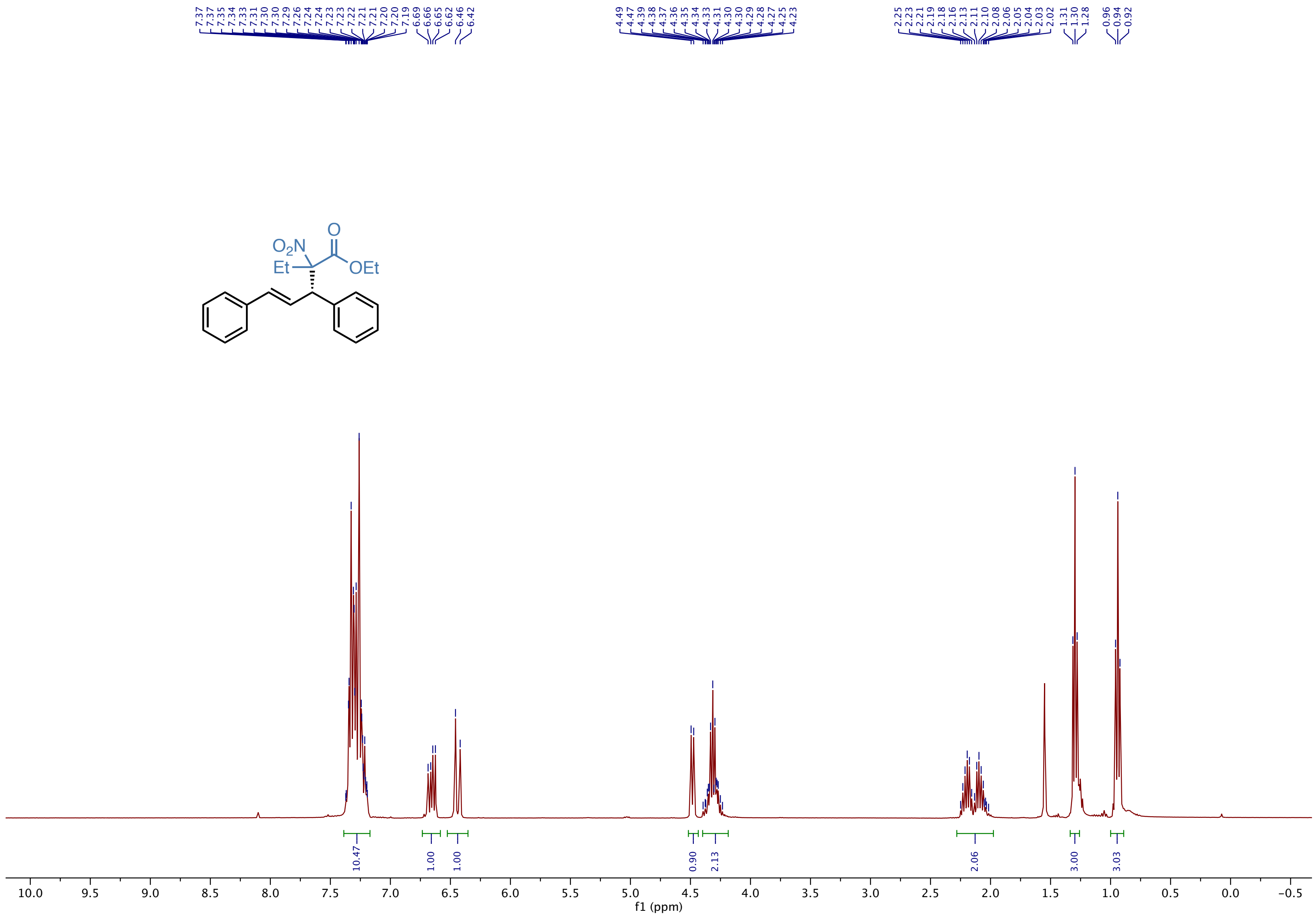

^{13}C NMR (101 MHz, $CDCl_3$): Ethyl (*S*,*E*)-2-ethyl-2-nitro-3,5-diphenylpent-4-enoate (3ib)

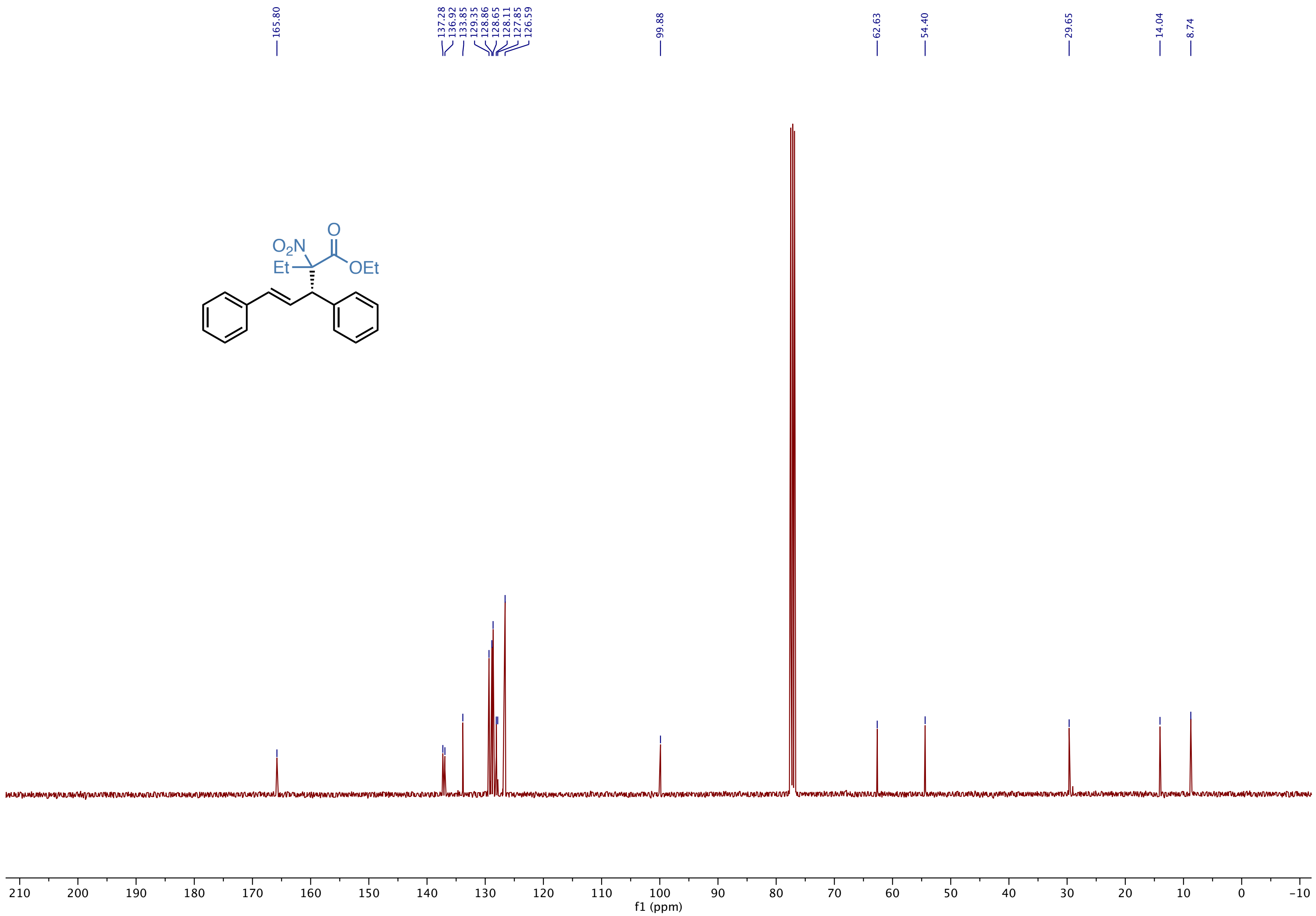

^{1}H NMR (400 MHz, $CDCl_3$): Ethyl (*S*,*E*)-2-nitro-3,5-diphenylpent-4-enoate (3ja + 3jb)

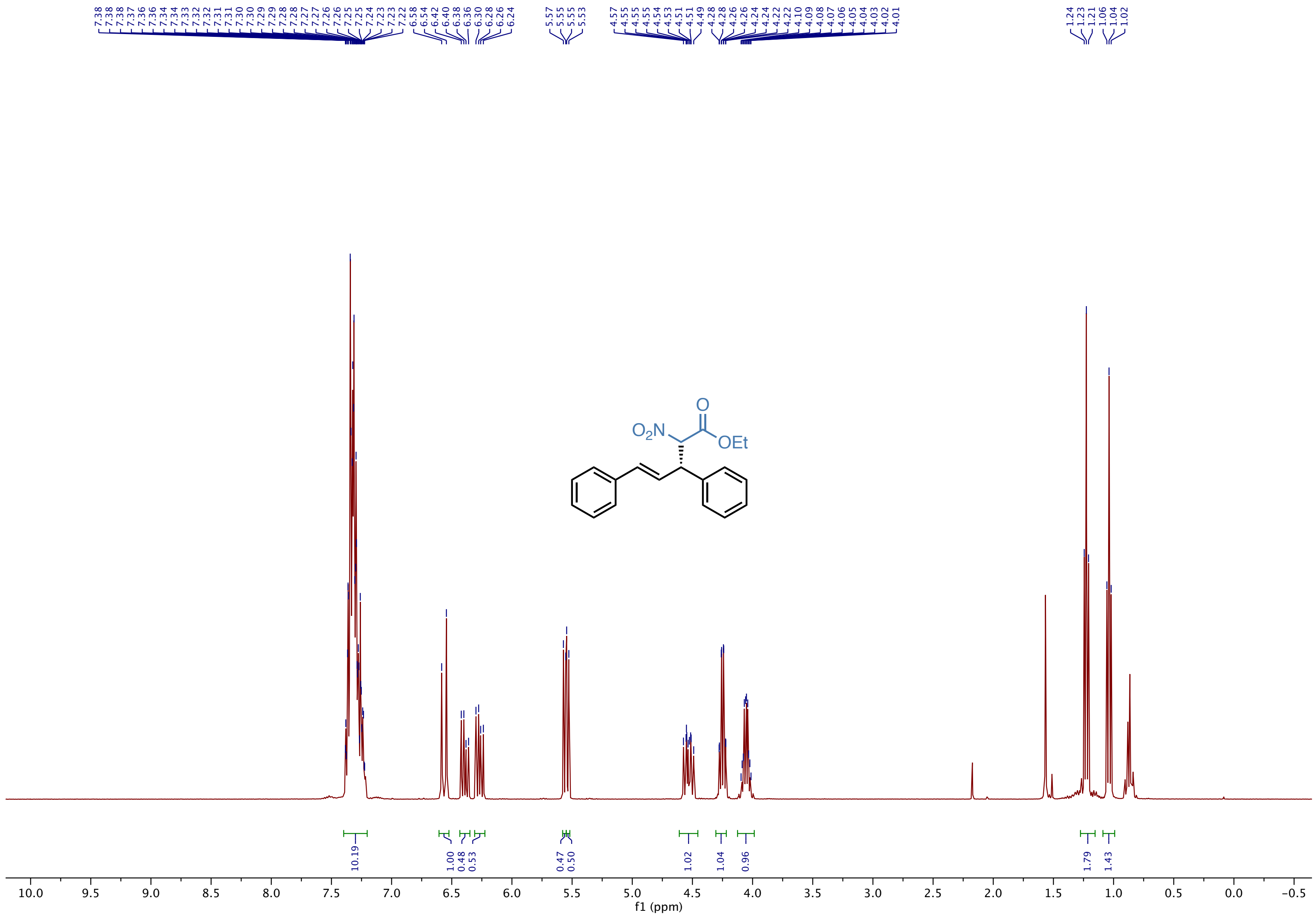

^{13}C NMR (101 MHz, $CDCl_3$): Ethyl (*S*,*E*)-2-nitro-3,5-diphenylpent-4-enoate (3ja + 3jb)

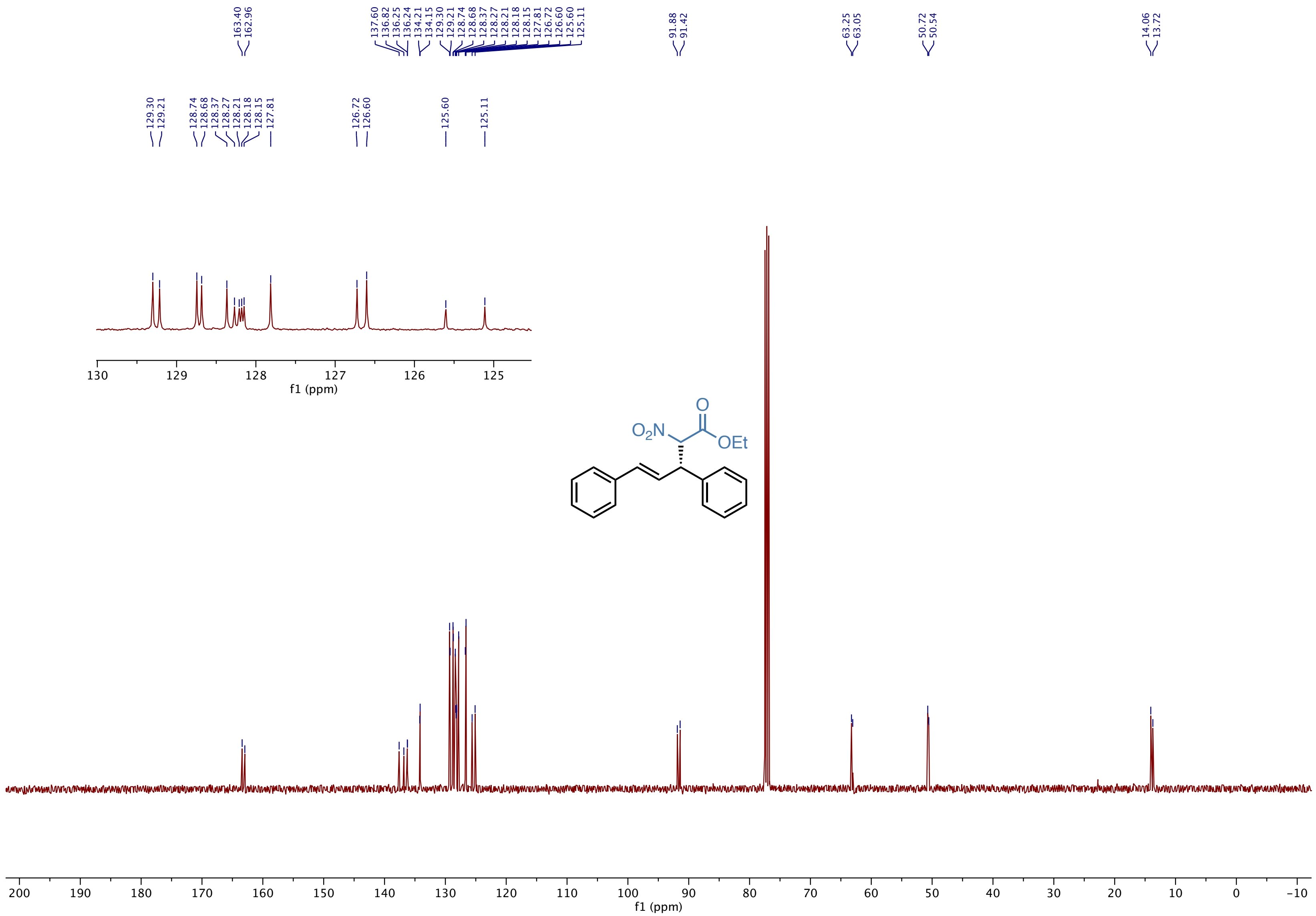

^{1}H NMR (700 MHz, $CDCl_3$): (*E*)-2-(1,3-diphenylallyl)-1,3-Diphenylpropane-1,3-dione (3k)

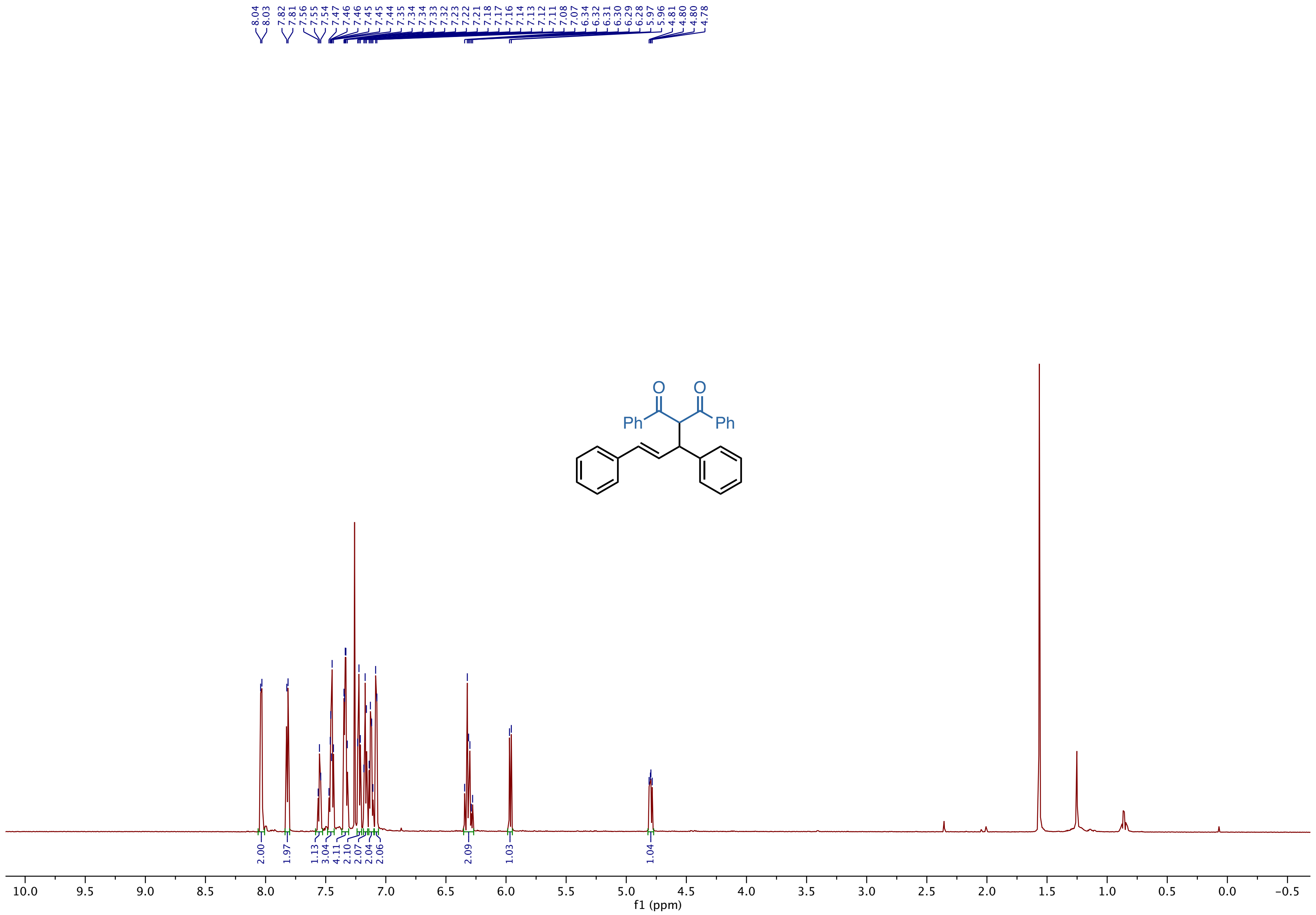

^{13}C NMR (176 MHz, $CDCl_3$): (*E*)-2-(1,3-diphenylallyl)-1,3-Diphenylpropane-1,3-dione (3k)

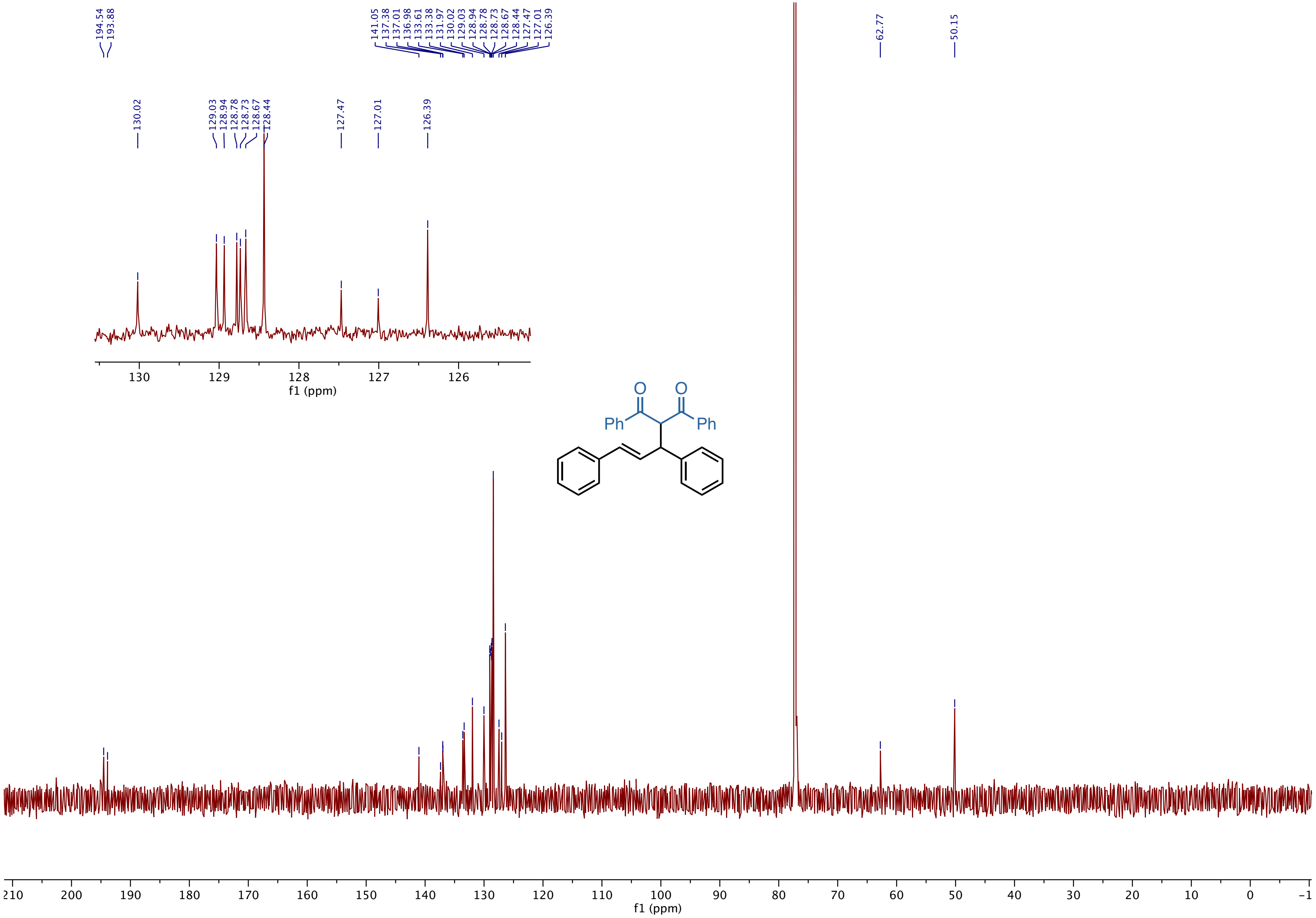

^{1}H NMR (400 MHz, $CDCl_3$): (*E*)-(4,4-bis(phenylsulfonyl)but-1-ene-1,3-diyl)Dibenzene (3l)

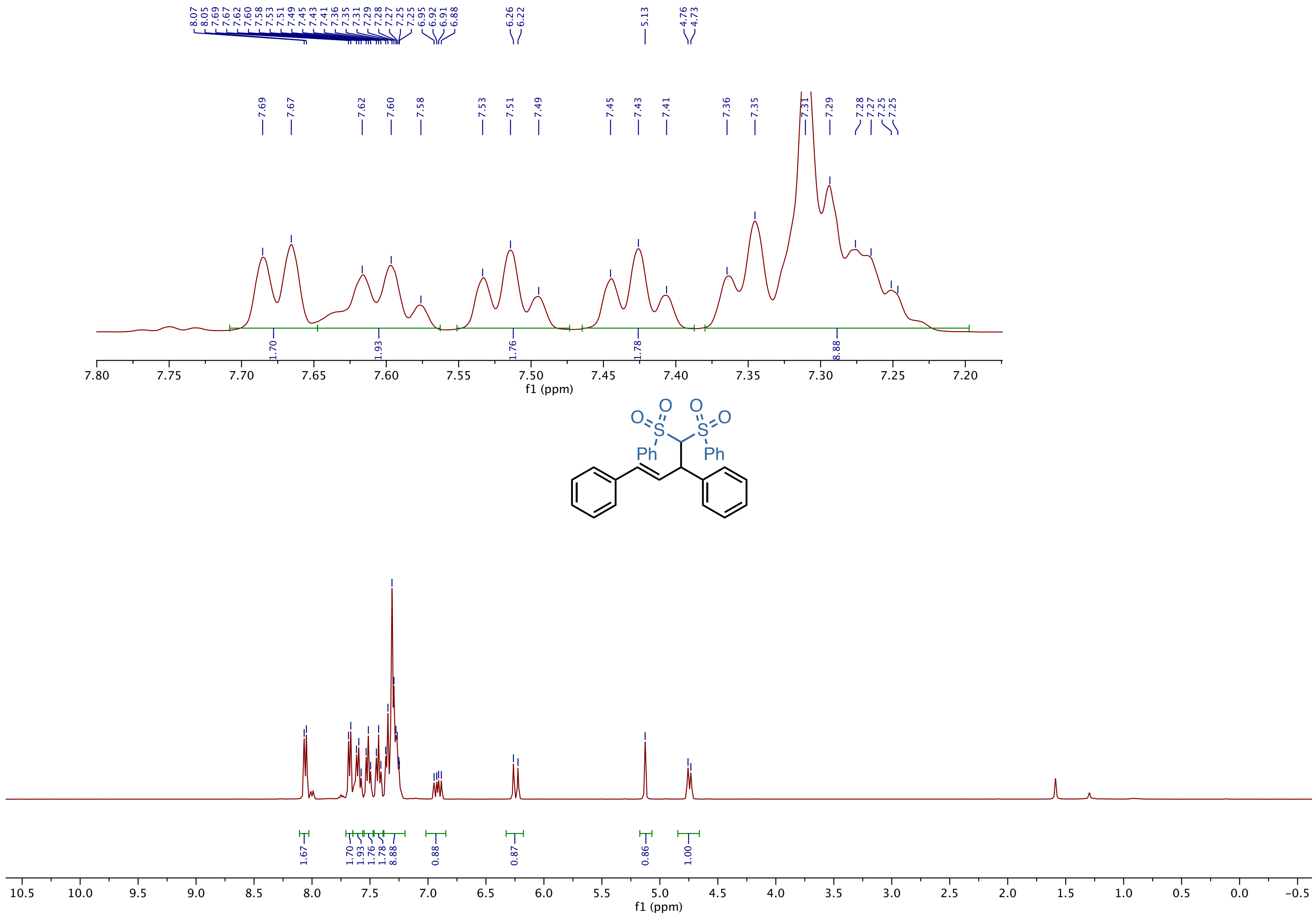

^{13}C NMR (101 MHz, $CDCl_3$): (*E*)-(4,4-bis(phenylsulfonyl)but-1-ene-1,3-diyl)Dibenzene (3l)

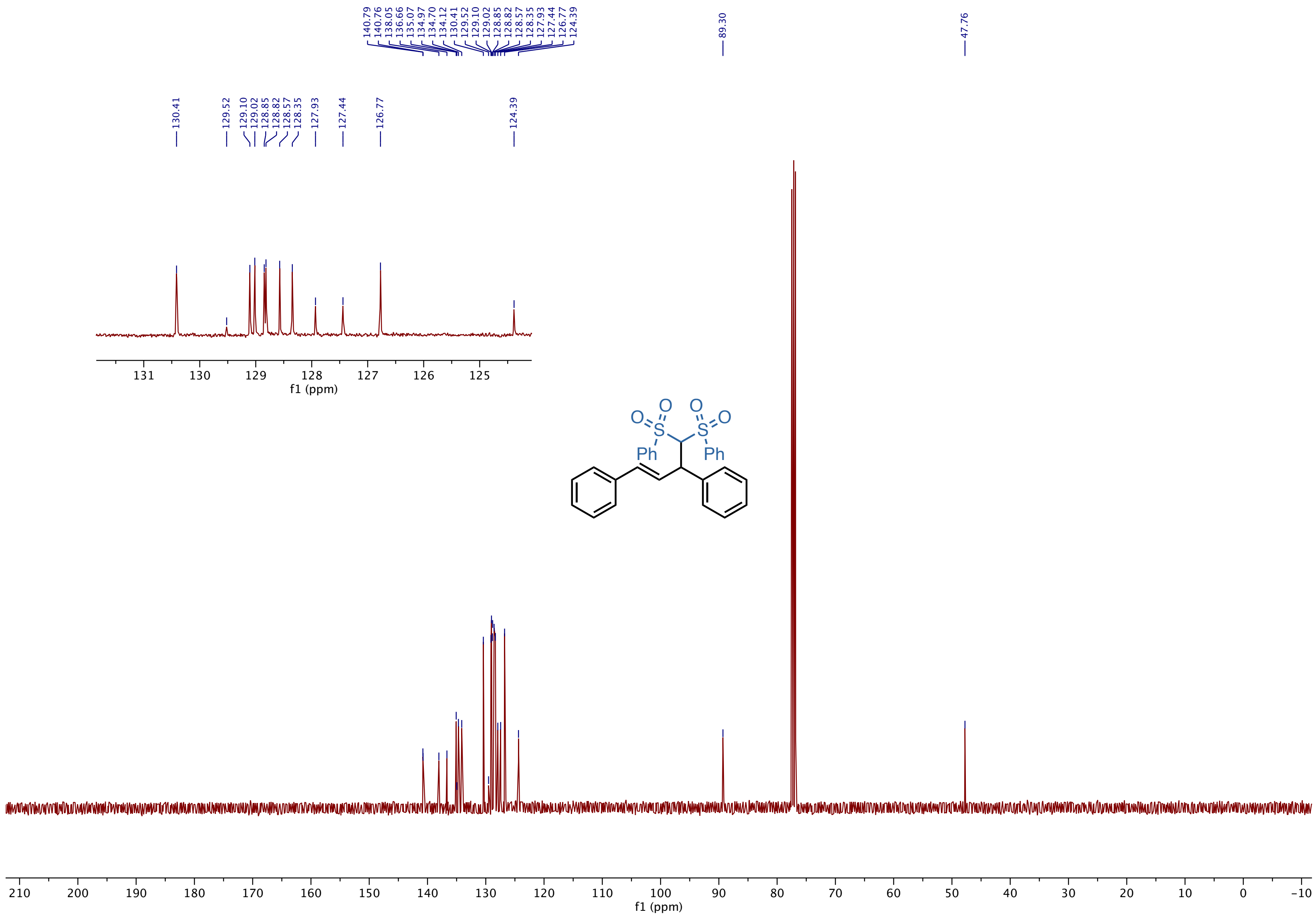

^{1}H NMR (500 MHz, $CDCl_3$): (*E*)-3-(1,3-diphenylallyl)-1*H*-Indole (3m)

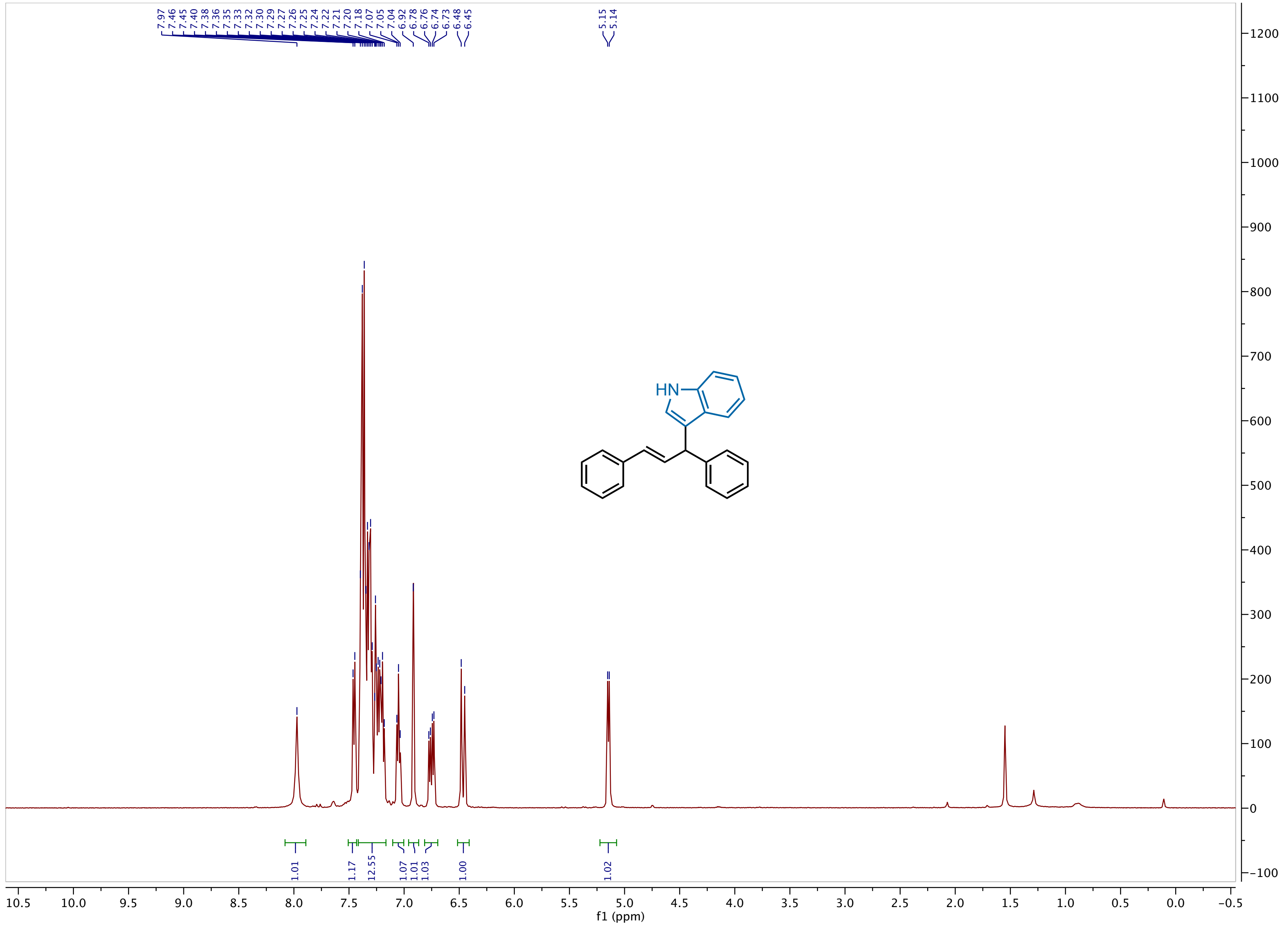

^{13}C NMR (126 MHz, $CDCl_3$): (*E*)-3-(1,3-diphenylallyl)-1*H*-Indole (3m)

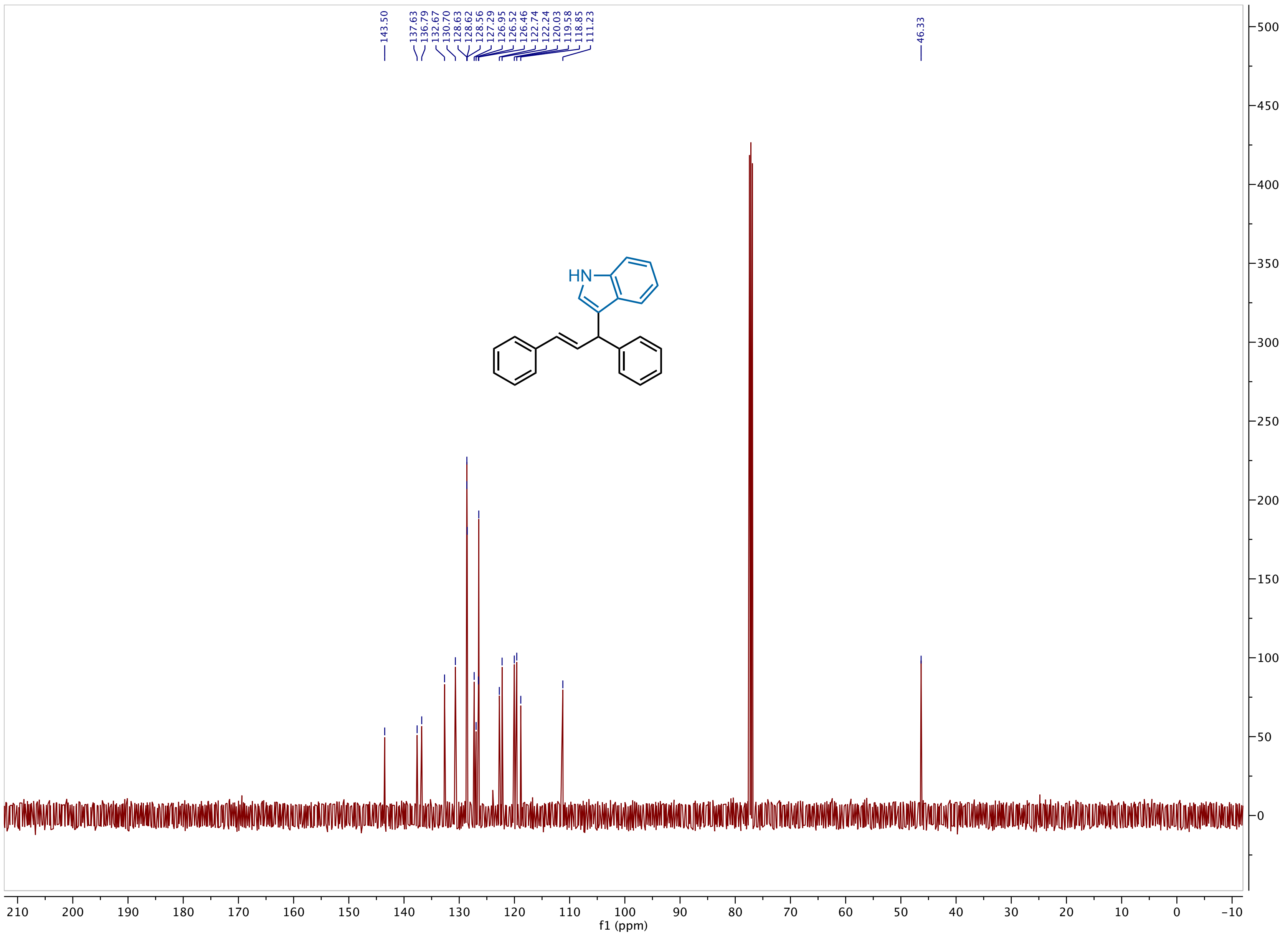

^{1}H NMR (400 MHz, $CDCl_3$): (*E*)-(3-phenoxyprop-1-ene-1,3-diyl)Dibenzene (3o)

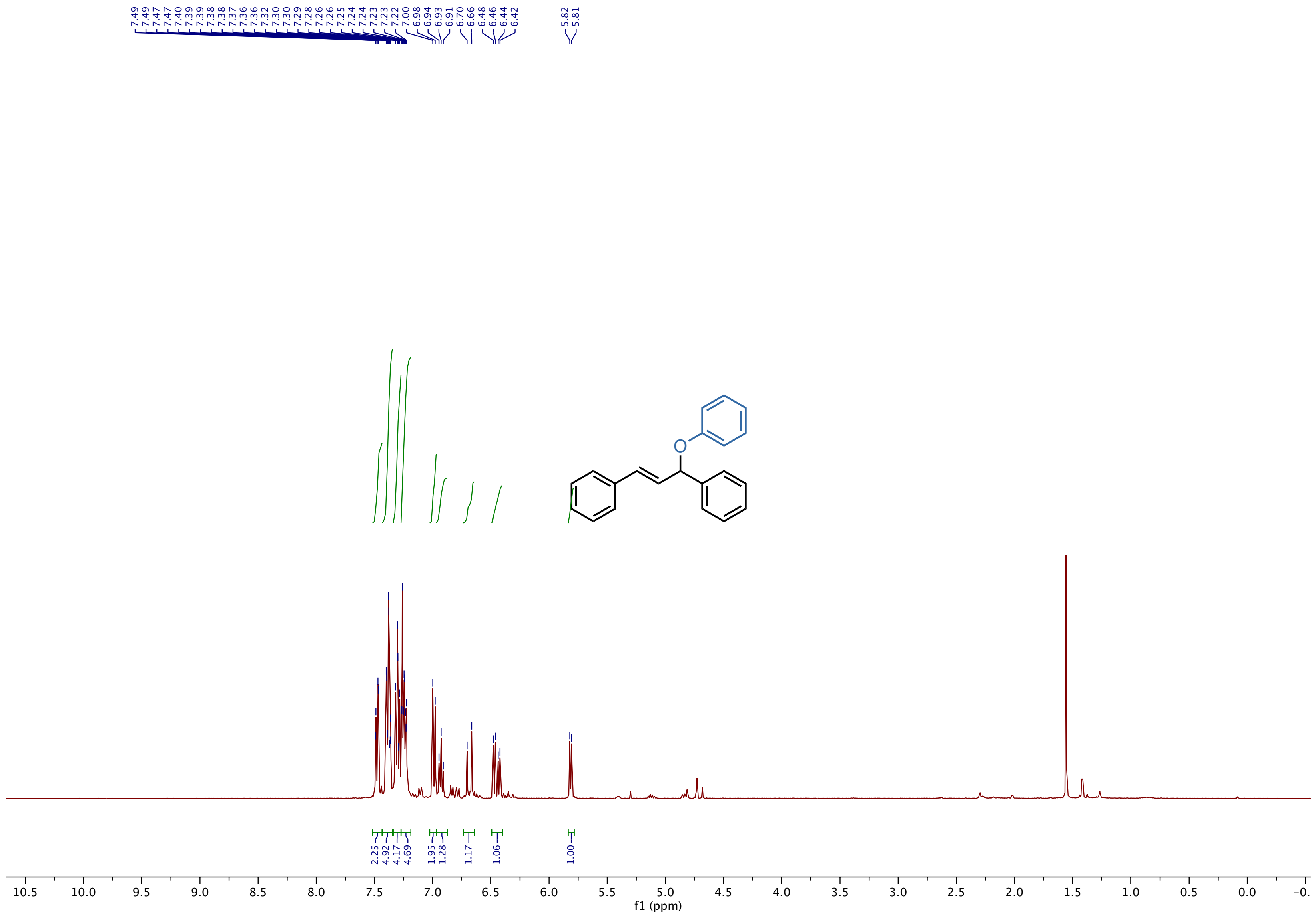

^{13}C NMR (101 MHz, $CDCl_3$): (*E*)-(3-phenoxyprop-1-ene-1,3-diyl)Dibenzene (3o)

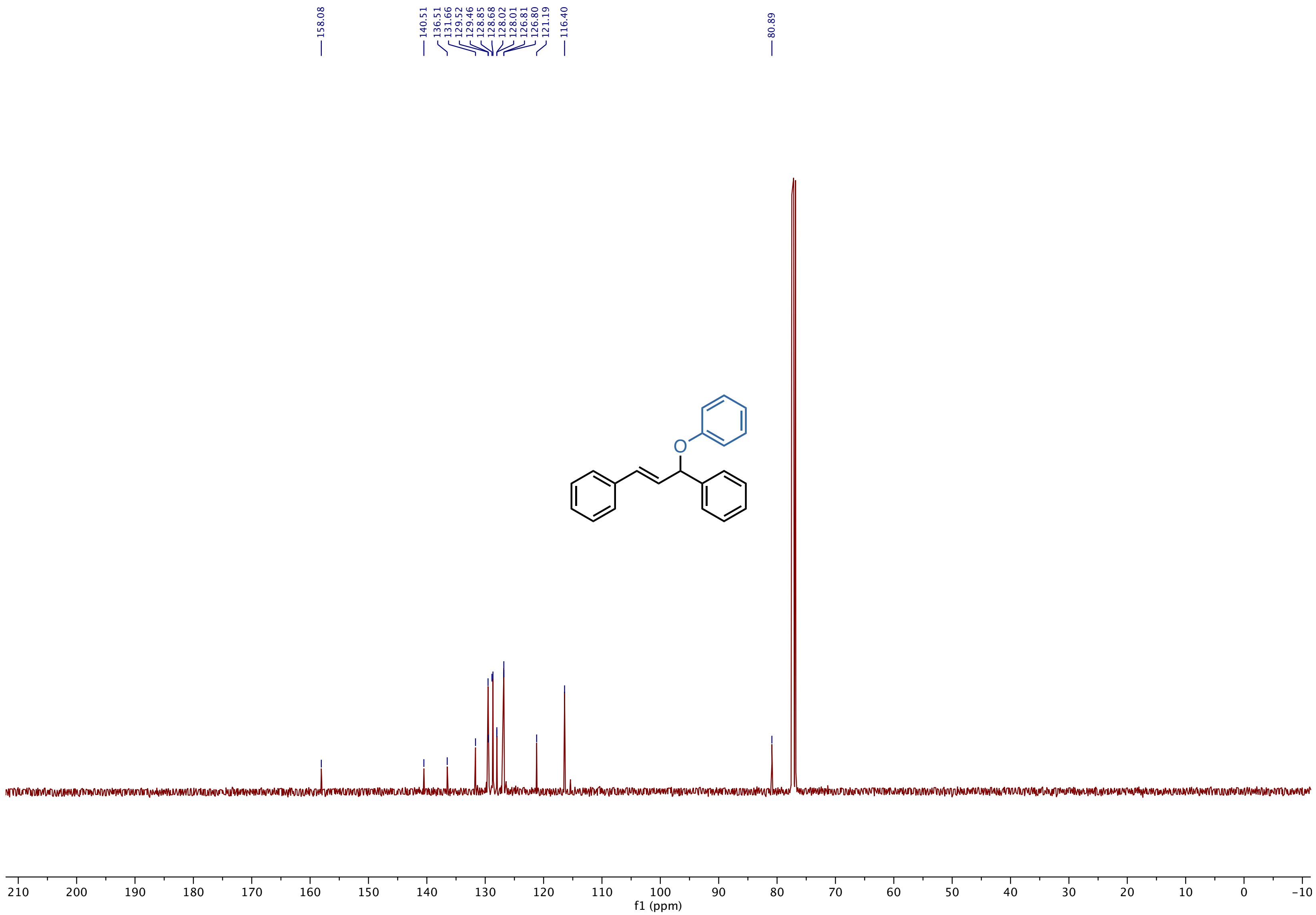

^{1}H NMR (400 MHz, $CDCl_3$): Methyl 1-cinnamyl-2-oxocyclohexane-1-carboxylate (3p)

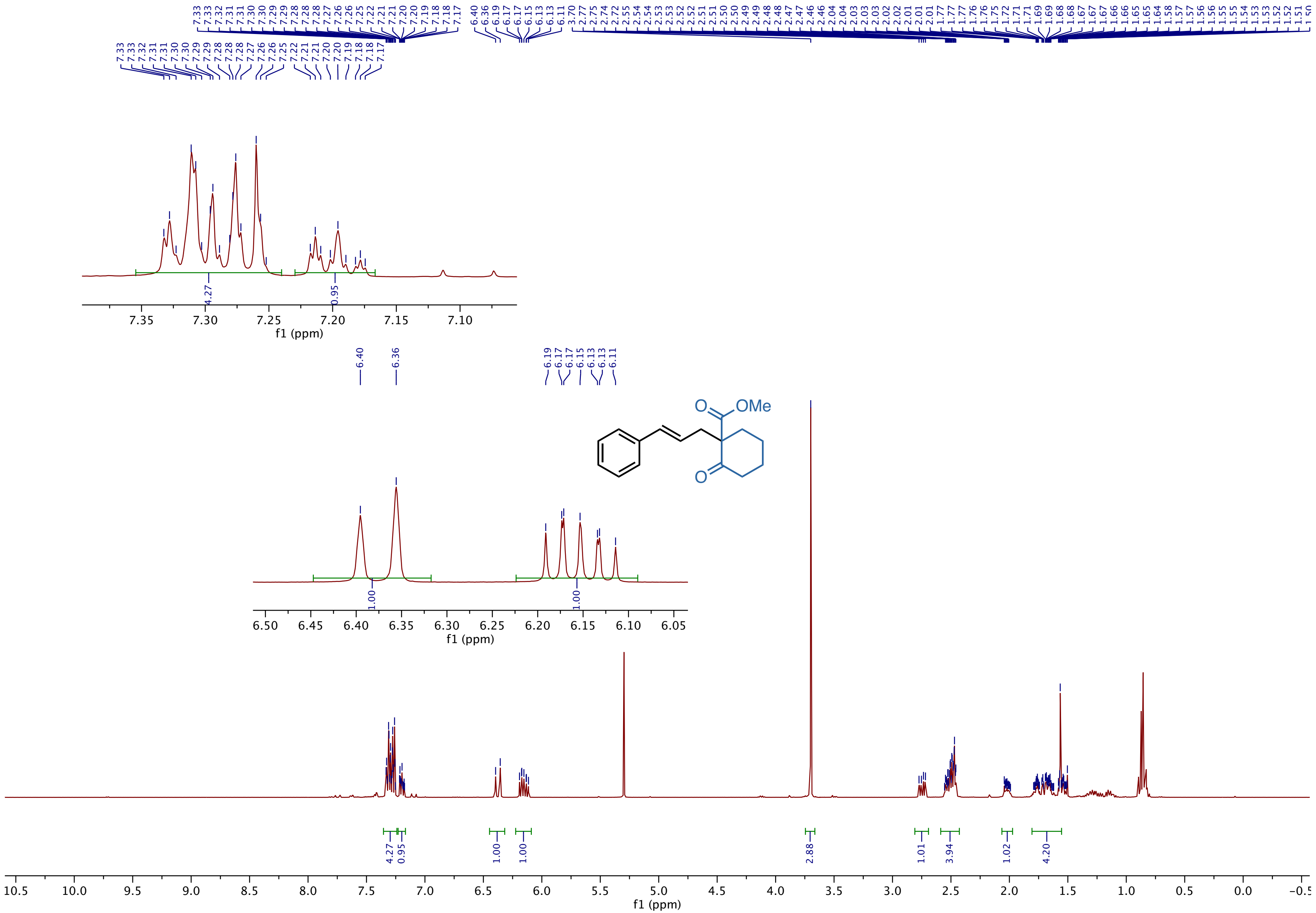

^{13}C NMR (101 MHz, $CDCl_3$): Methyl 1-cinnamyl-2-oxocyclohexane-1-carboxylate (3p)

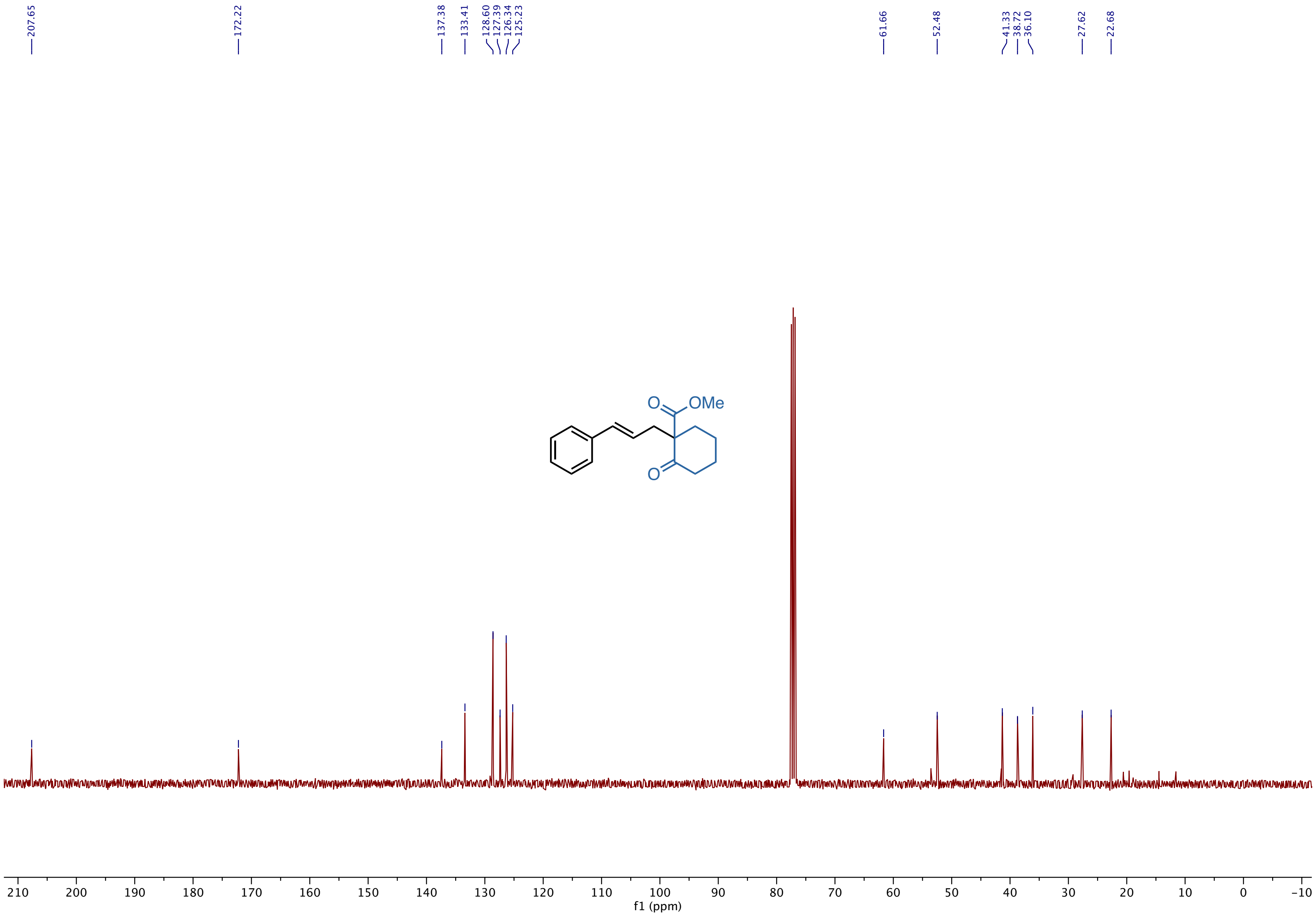

^{1}H NMR (400 MHz, $CDCl_3$): Dimethyl (*E*)-2-(4-phenylbut-3-en-2-yl)malonate (3q)

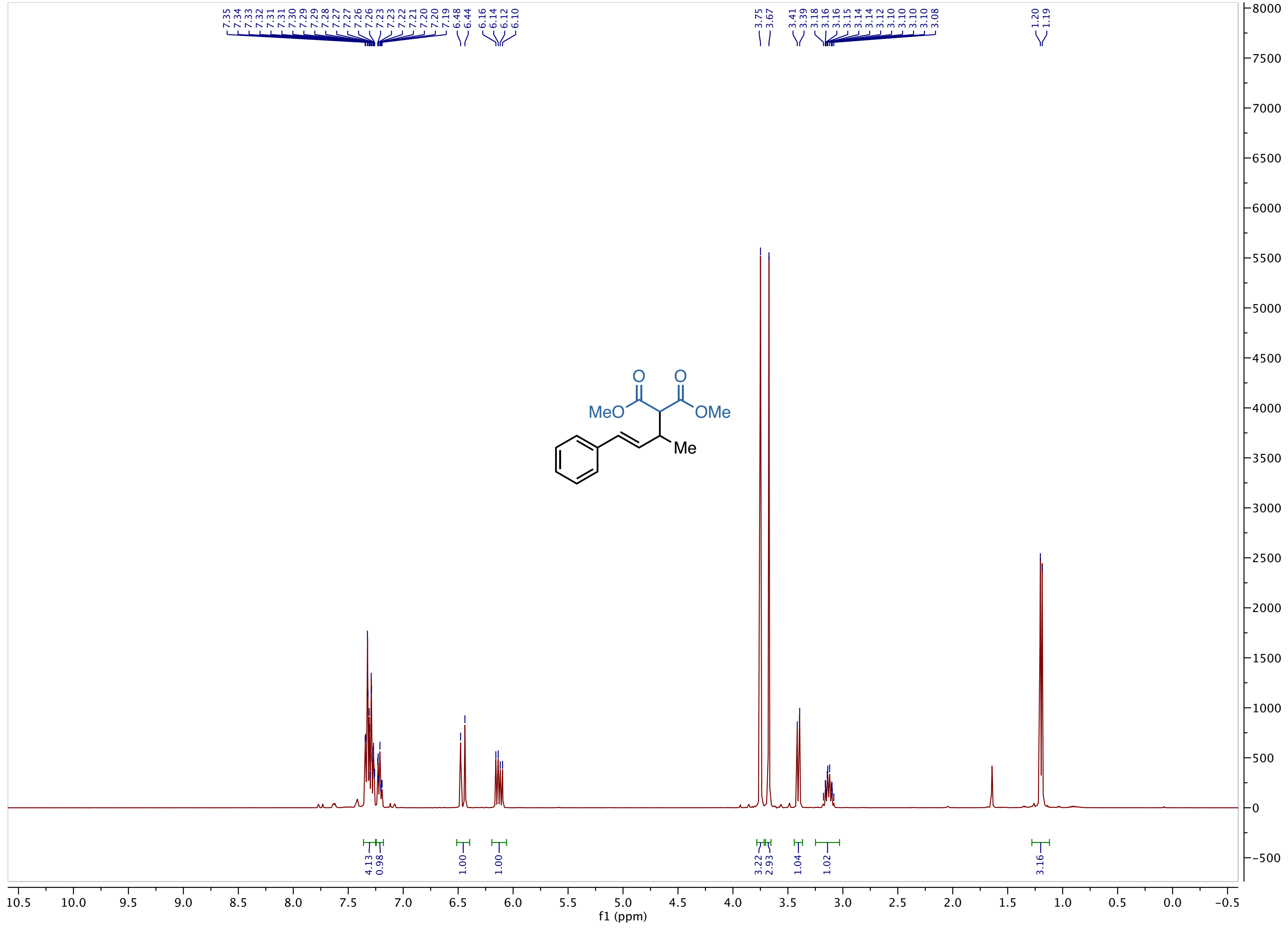

^{13}C NMR (101 MHz, $CDCl_3$): Dimethyl (*E*)-2-(4-phenylbut-3-en-2-yl)malonate (3q)

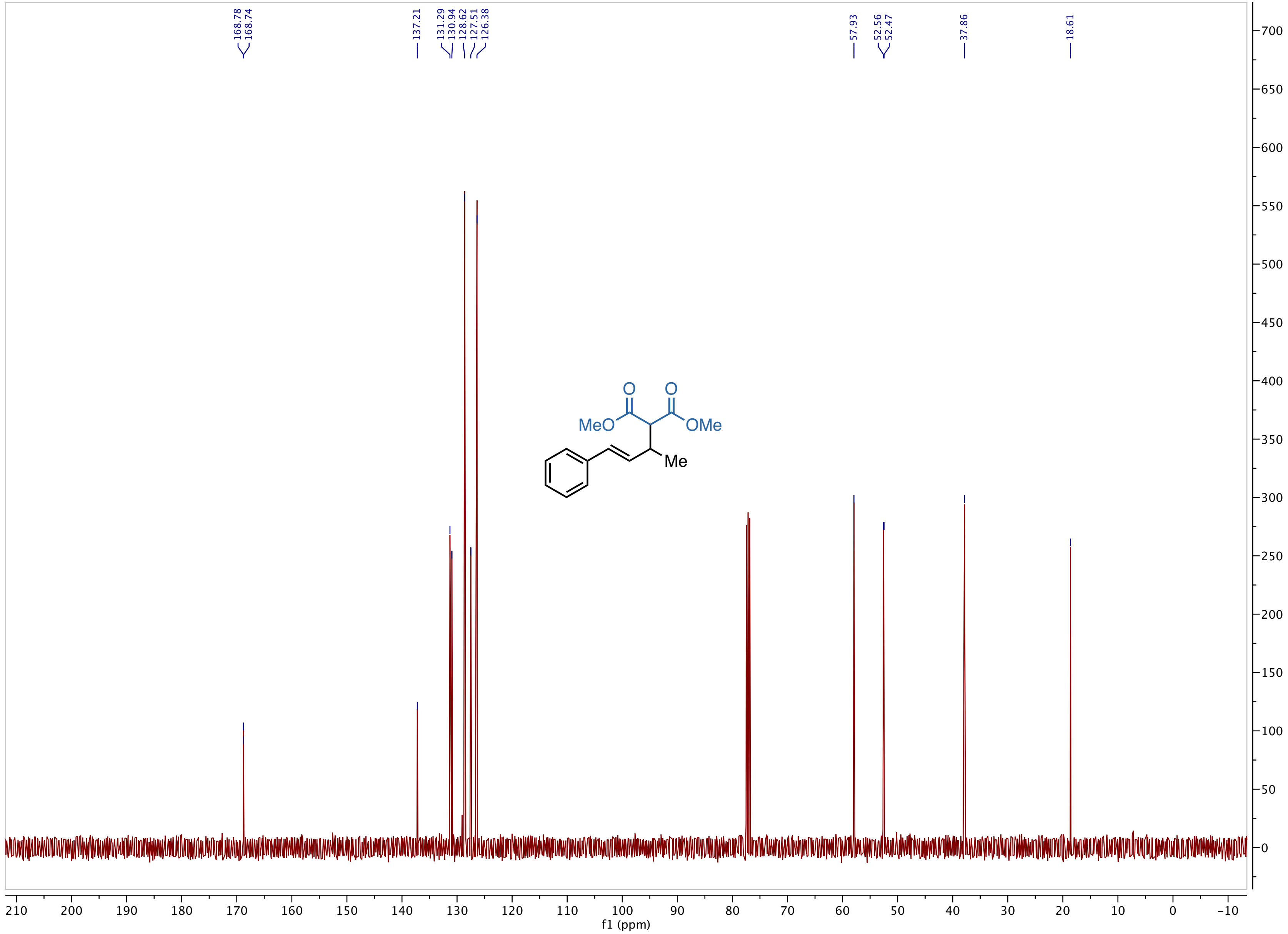

Dimethyl (*R,E*)-2-(1,3-diphenylallyl)malonate (3a) (0.2 mmol scale)

Chiral SFC Analysis CHIRAL ART SJ (CO_2:*i*-PrOH, 99:01, 2.5 mL min^{-1}, 40 °C) t_R = 6.48 (minor), 6.81 (major) minutes.

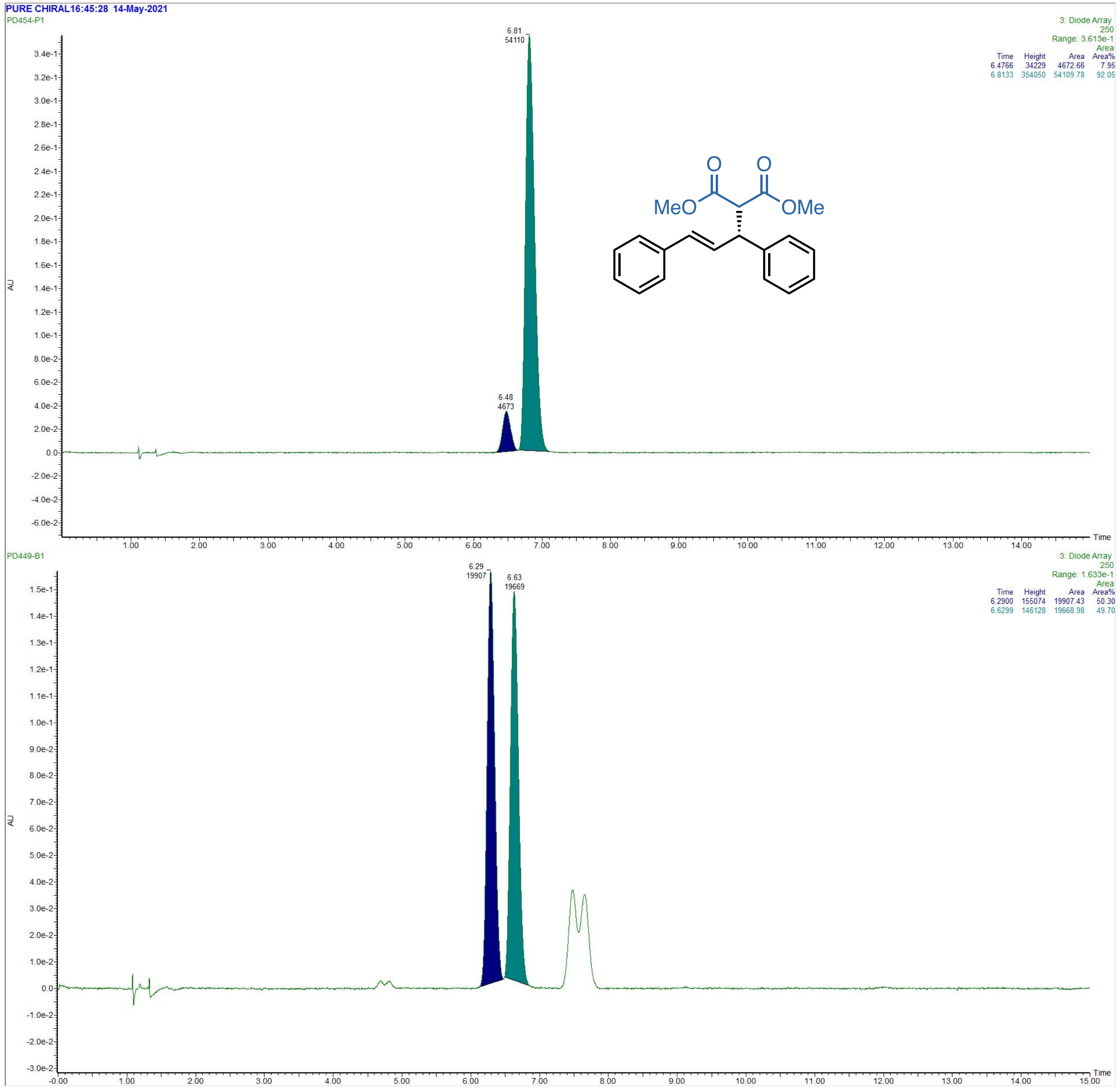

Dimethyl (*R,E*)-2-(1,3-diphenylallyl)malonate (3a) (Control Experiment Scheme 2D, (*R*)-sSPhos-Np as the ligand)

Chiral SFC Analysis CHIRAL PAK IA (CO_2:MeOH, 99:01, 2.5 mL min^{-1}, 40 °C) t_R = 9.23 (major), 10.11 (minor) minutes.

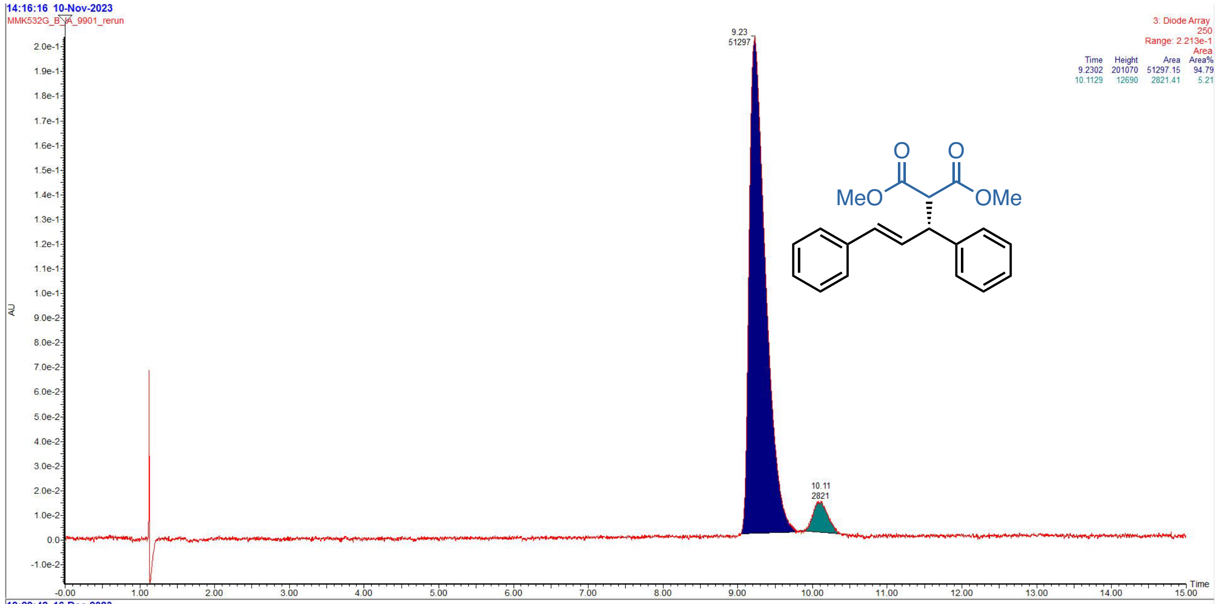

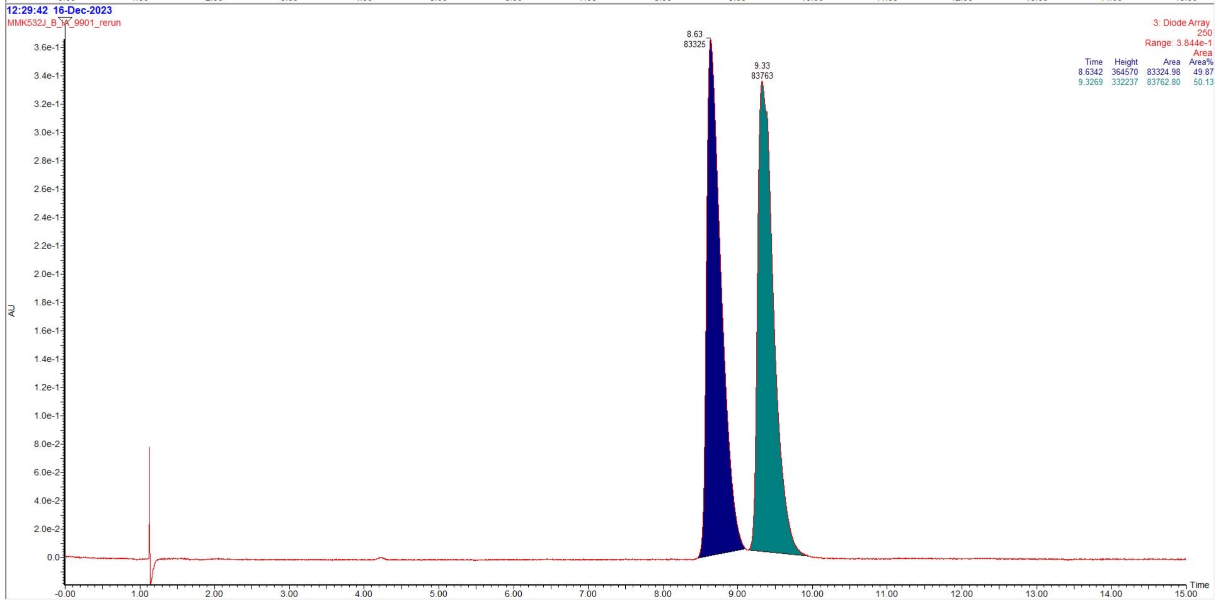

Dimethyl (*R,E*)-2-(1,3-diphenylallyl)malonate (3a) (1 mmol scale)

Chiral SFC Analysis CHIRAL PAK IA (CO_2:MeOH, 99:01, 2.5 mL min^{-1}, 40 °C) t_R = 8.19 (major), 9.08 (minor) minutes.

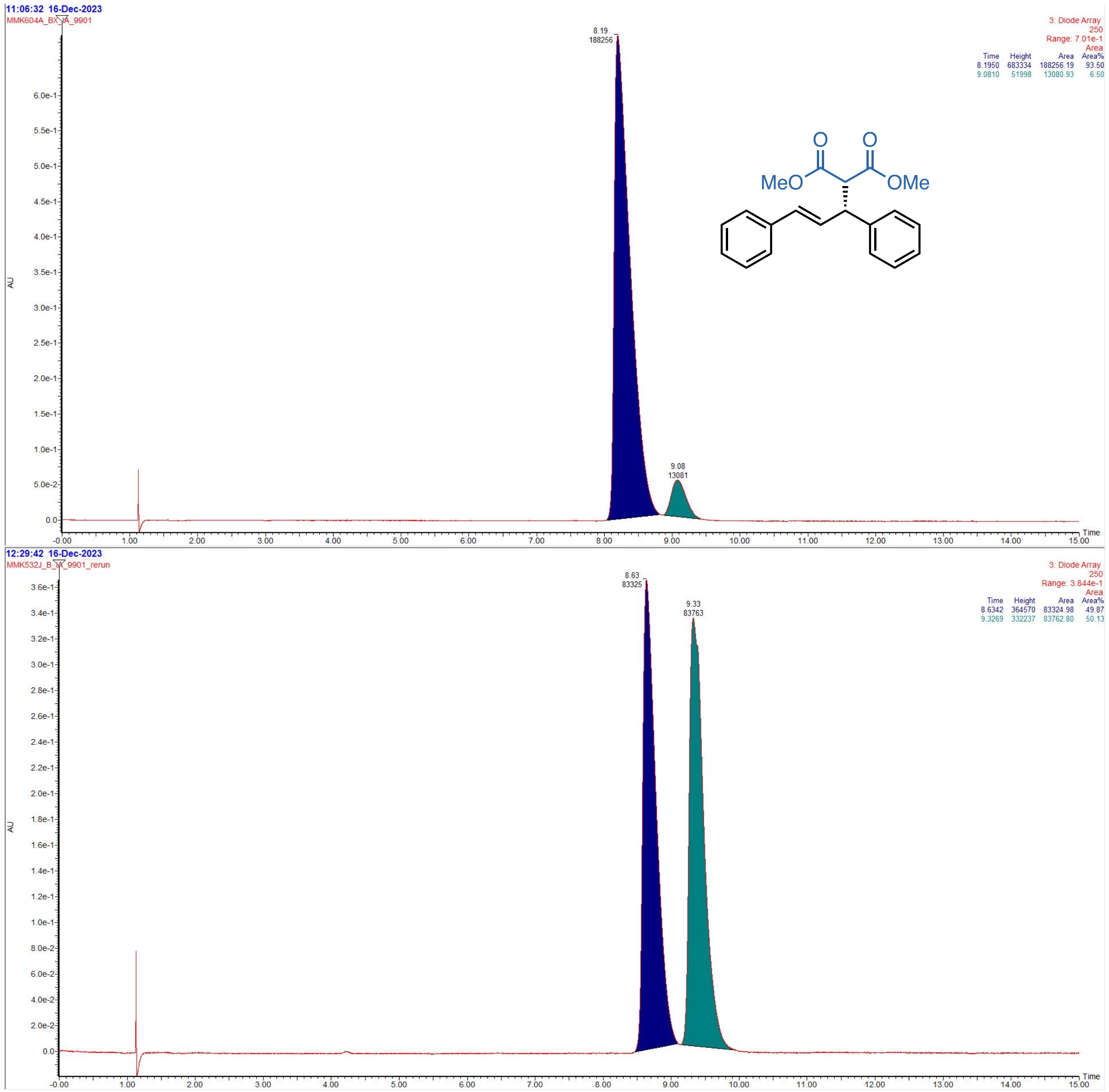

Dimethyl (*S,E*)-2-(1,3-diphenylallyl)-2-methylmalonate (3b)

Chiral SFC Analysis CHIRAL ART SJ (CO_2:*i*-PrOH, 99:01, 2.5 mL min^{-1}, 40 °C) t_R = 6.54 (major), 8.16 (minor) minutes.

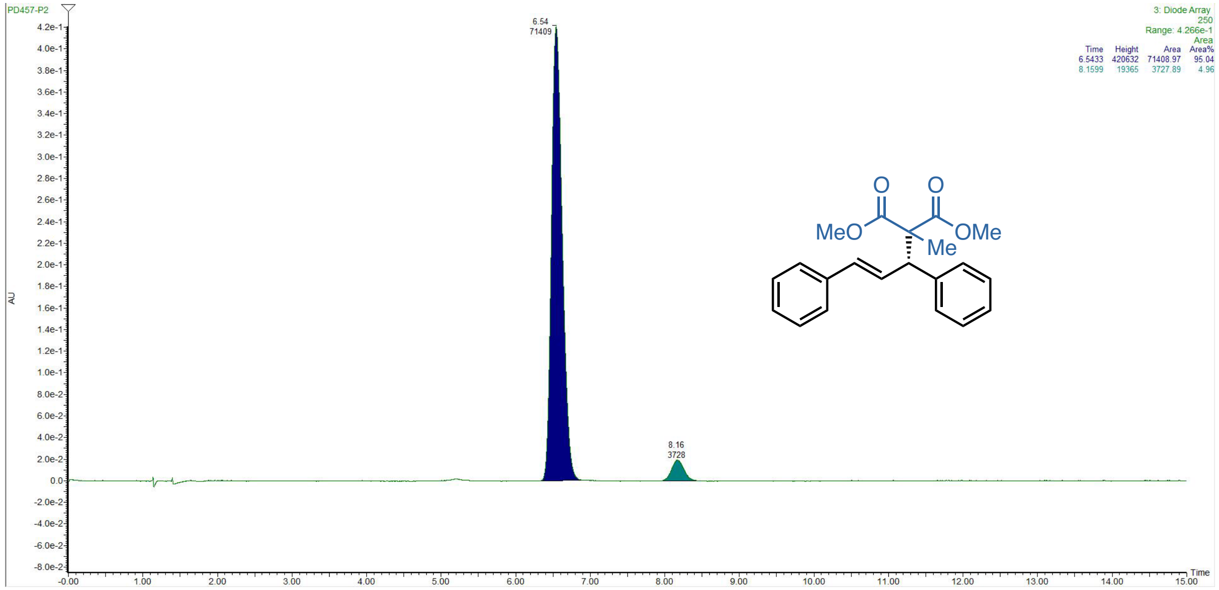

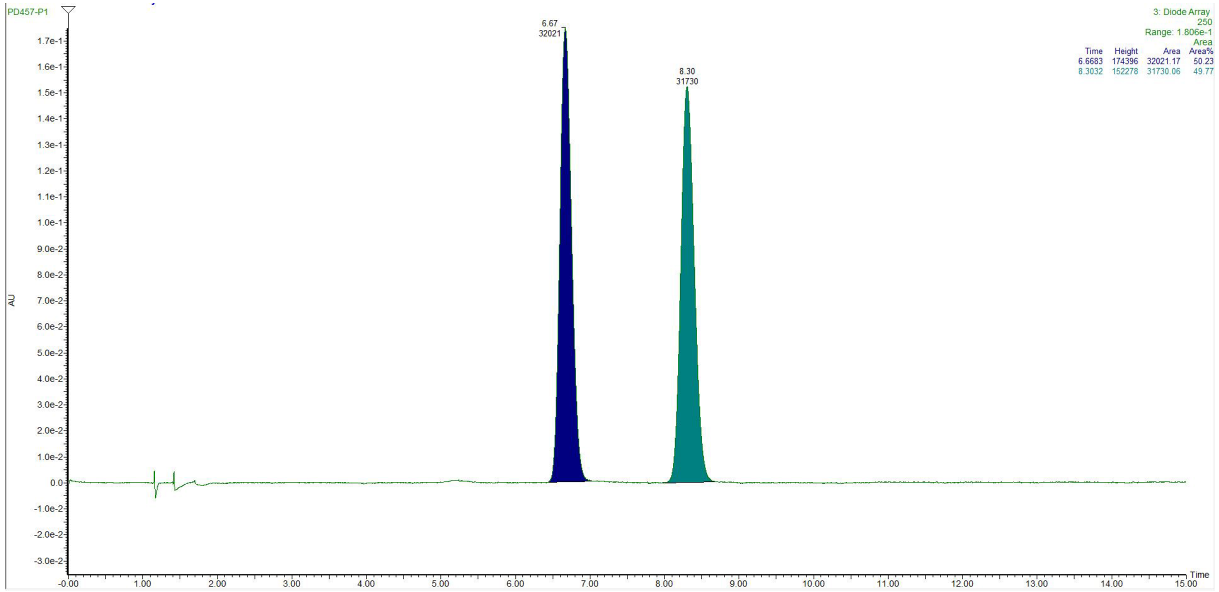

(*R,E*)-3-(1,3-diphenylallyl)Pentane-2,4-dione (3c)

Chiral SFC Analysis CHIRAL ART SJ (CO_2:*i*-PrOH, 99:01, 2.5 mL min^{-1}, 40 °C) t_R = 7.30 (minor), 7.81 (major) minutes.

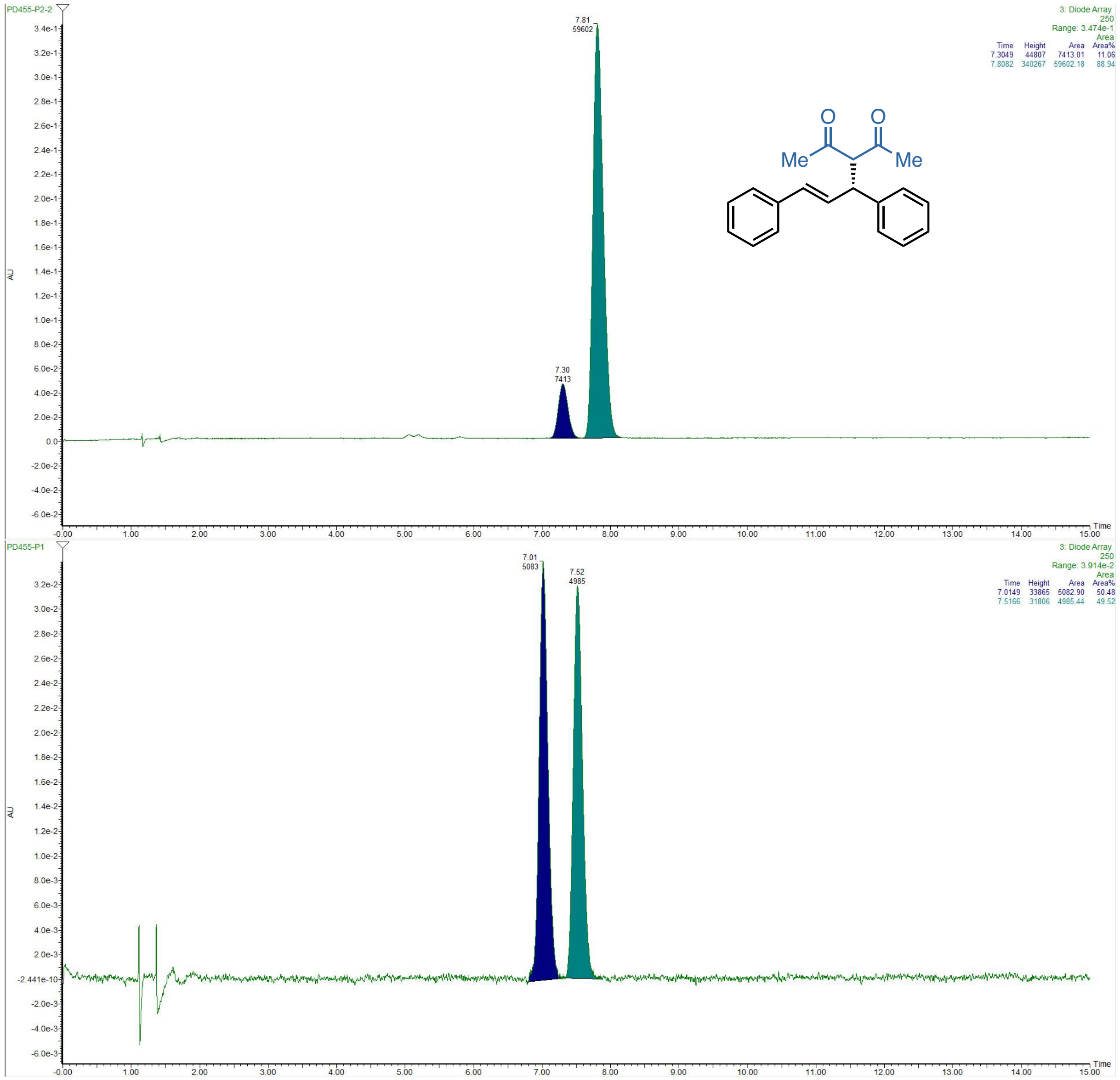

(*S,E*)-(4-methyl-4-nitropent-1-ene-1,3-diyl)Dibenzene (3d)

Chiral SFC Analysis CHIRAL ART SJ (CO_2:*i*-PrOH, 98:02, 2.5 mL min^{-1}, 40 °C) t_R = 9.14 (major), 11.44 (minor) minutes.

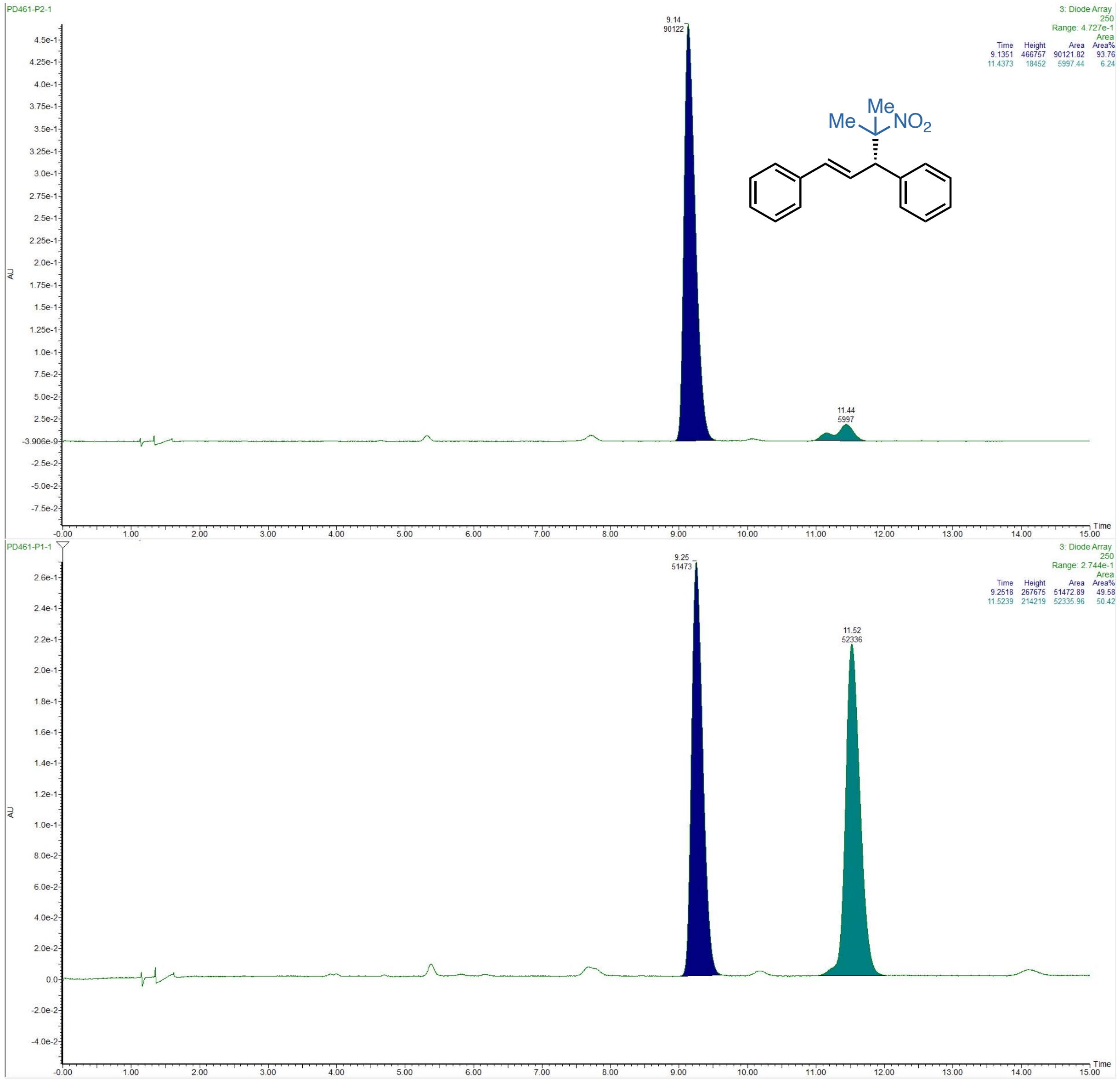

((*S*,*E*)-4-Nitropent-1-ene-1,3-diyl)dibenzene (3ea)

Chiral SFC Analysis CHIRAL ART SJ (CO_2:*i*-PrOH, 99:01, 2.5 mL min^{-1}, 40 °C) t_R = 11.23 (minor), 11.57 (major) minutes.

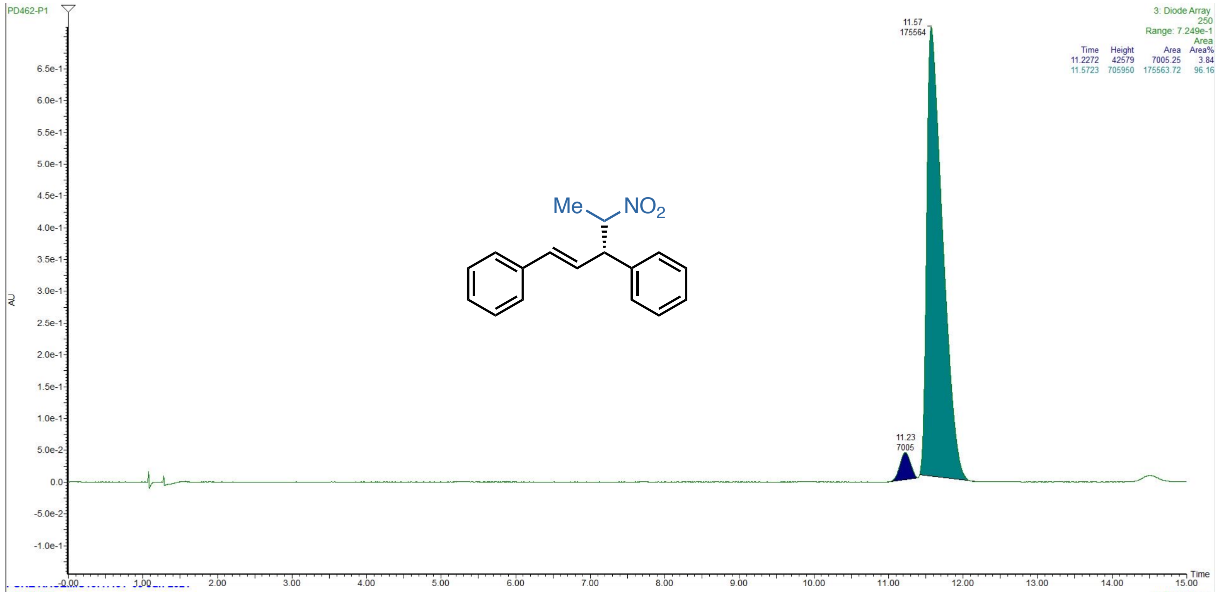

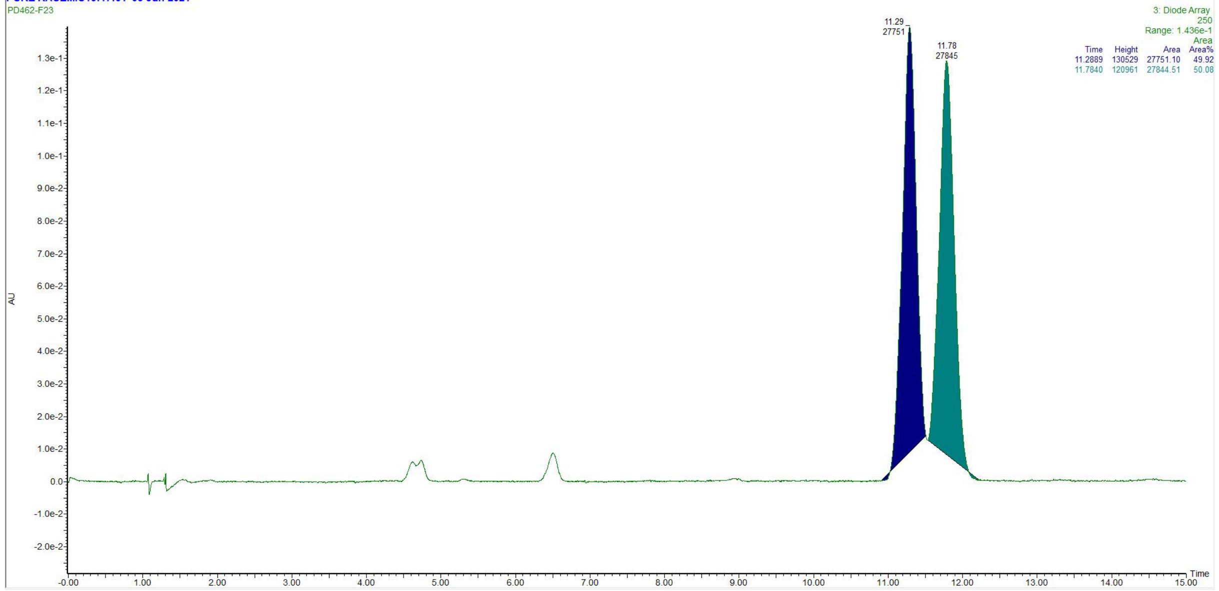

((*S*,*E*)-4-Nitropent-1-ene-1,3-diyl)dibenzene (3eb)

Chiral SFC Analysis CHIRAL ART SJ (CO_2:MeOH, 98:02, 2.5 mL min^{-1}, 40 °C) t_R = 10.31 (major), 11.86 (minor) minutes.

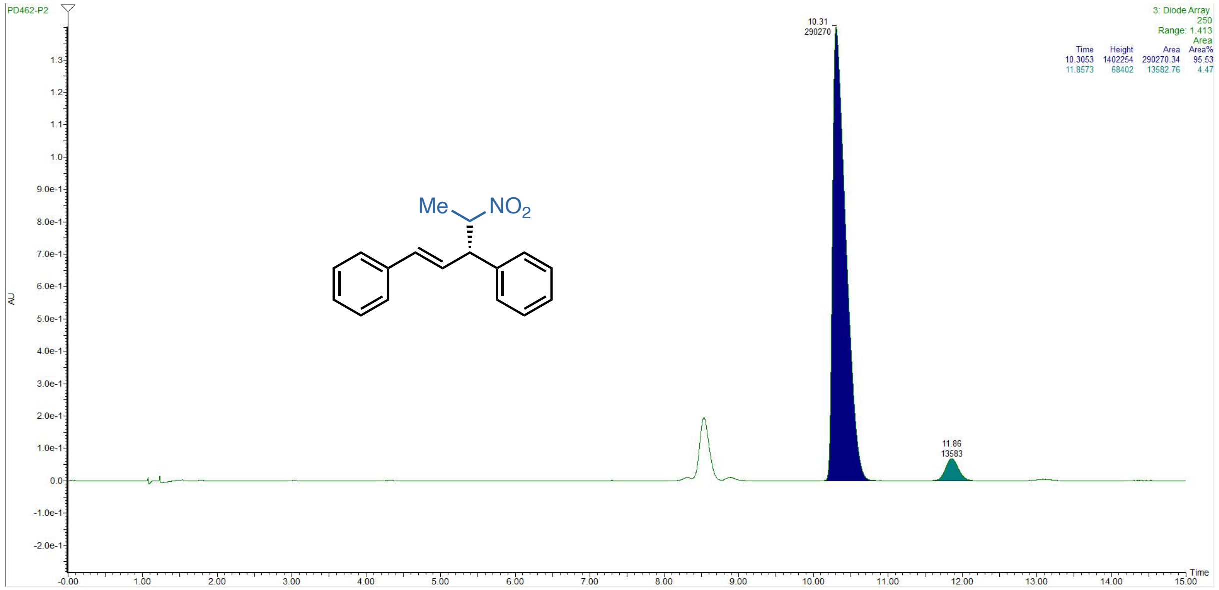

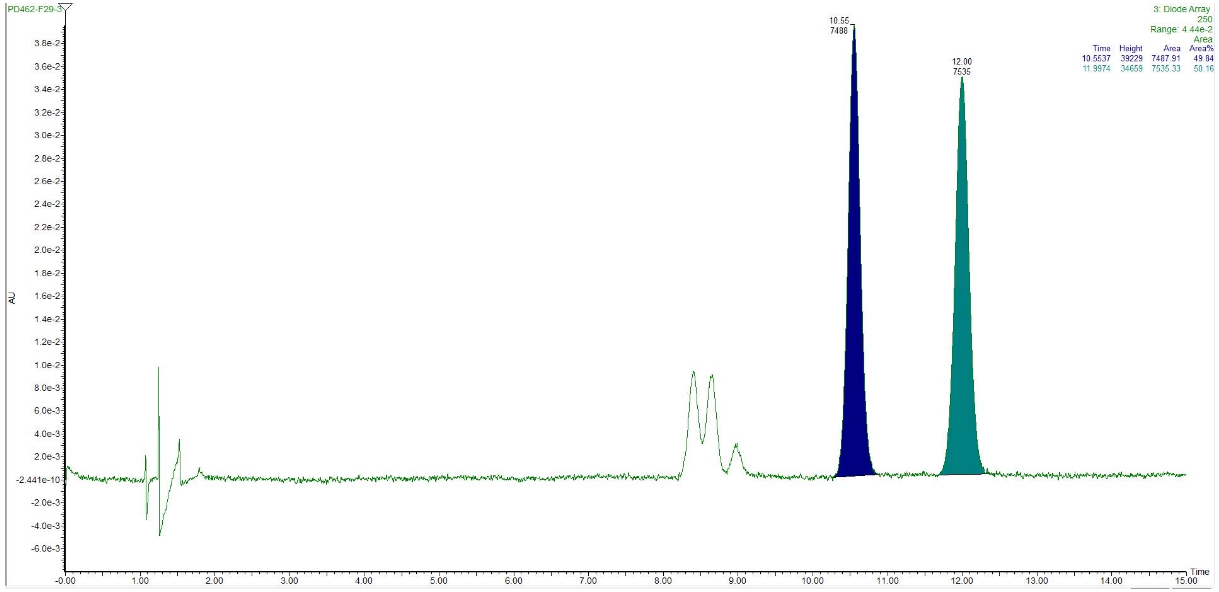

Methyl (*S,E*)-2-((diphenylmethylene)amino)-3,5-diphenylpent-4-enoate (3fa)

Chiral SFC Analysis CHIRAL PAK IG (CO_2:MeOH, 95:05, 2.5 mL min^{-1}, 40 °C) t_R = 8.95 (major), 9.88 (minor) minutes.

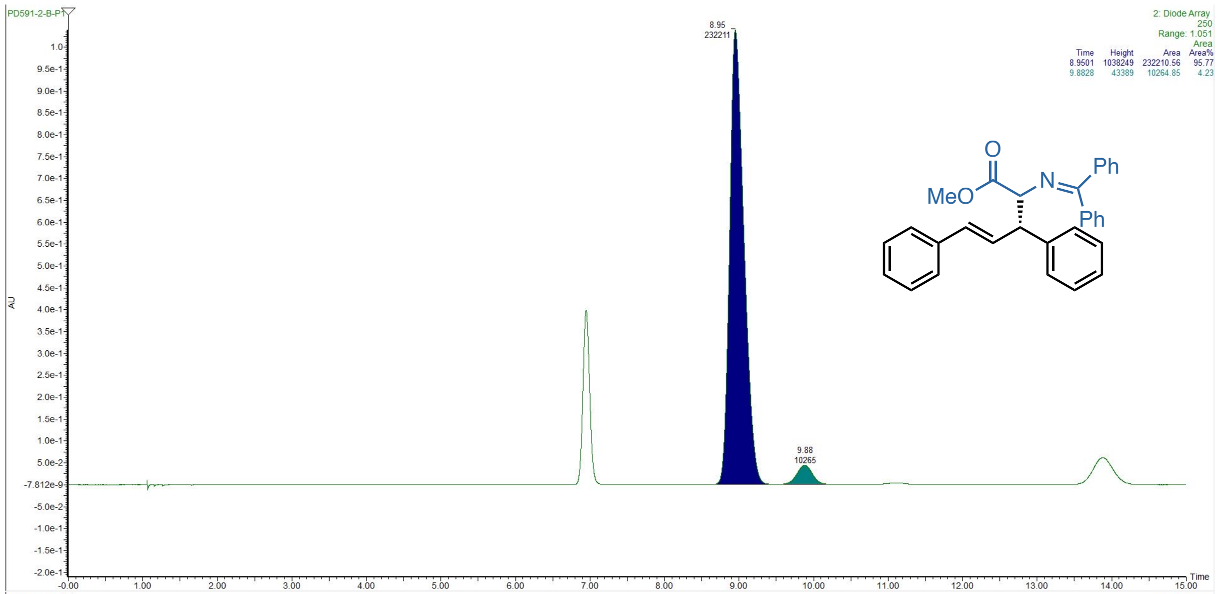

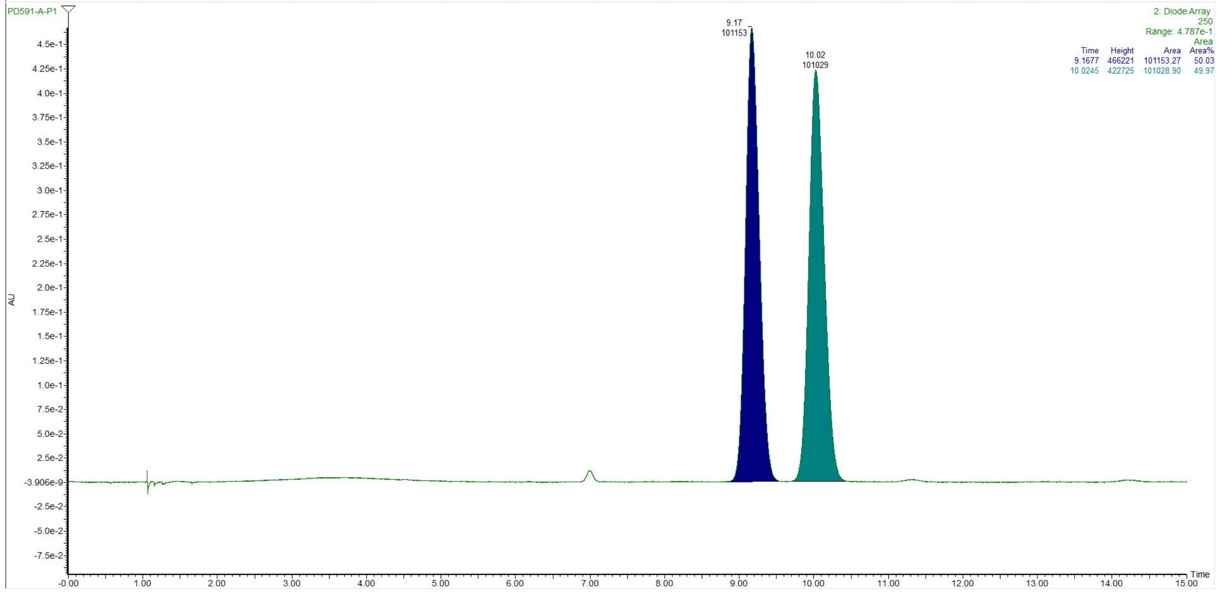

Methyl (*S,E*)-2-((diphenylmethylene)amino)-3,5-diphenylpent-4-enoate (3fb)

Chiral SFC Analysis CHIRAL PAK IG (CO_2:MeOH, 95:05, 2.5 mL min^{-1}, 40 °C) t_R = 11.23 (minor), 13.82 (major) minutes.

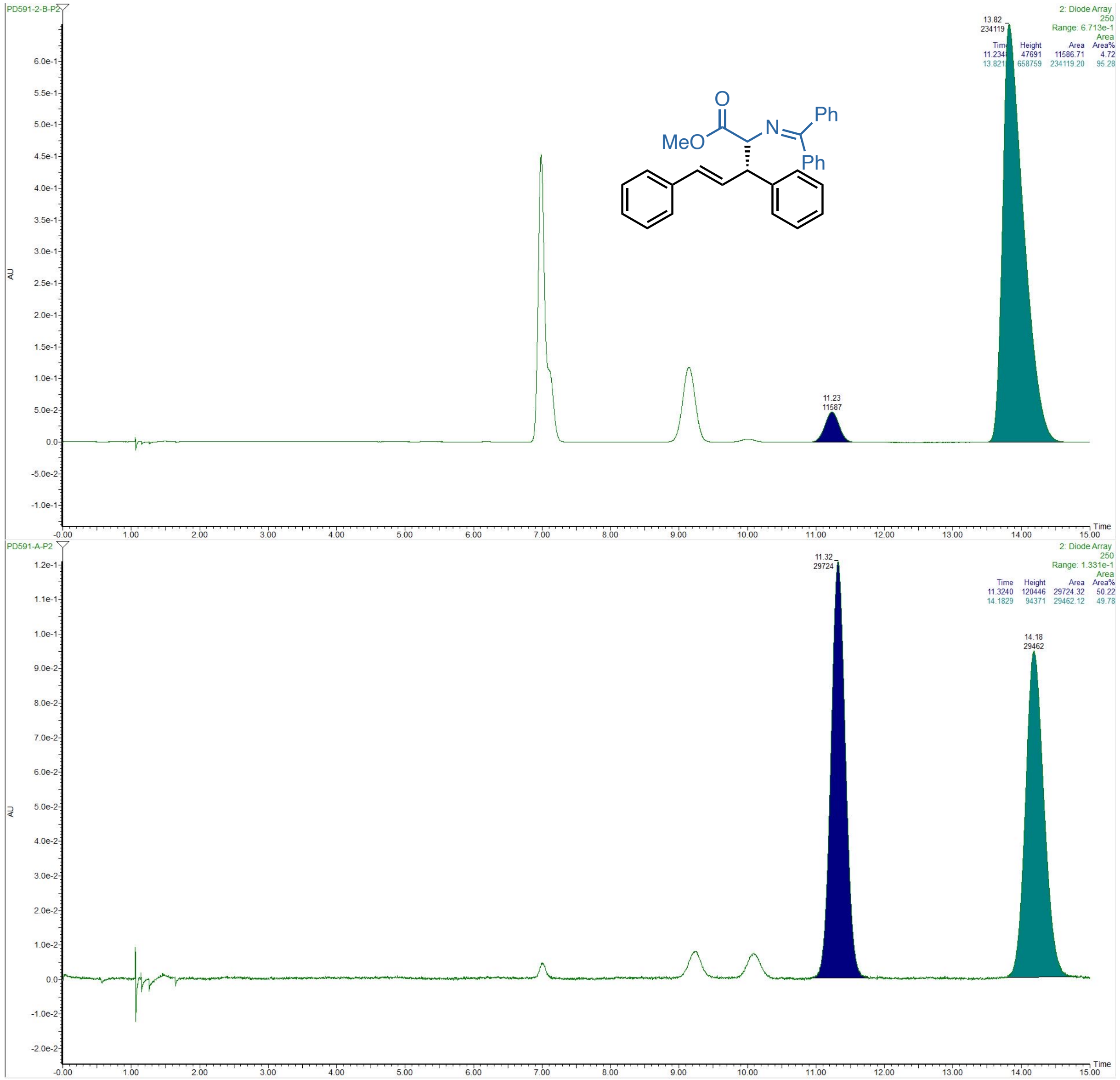

Methyl (*S*,*E*)-1-(1,3-diphenylallyl)-2-oxocyclohexane-1-carboxylate (3ga)

Chiral SFC Analysis CHIRAL PAK IG (CO_2:MeOH, 95:05, 2.5 mL min^{-1}, 40 °C) t_R = 8.26 (minor), 8.59 (major) minutes.

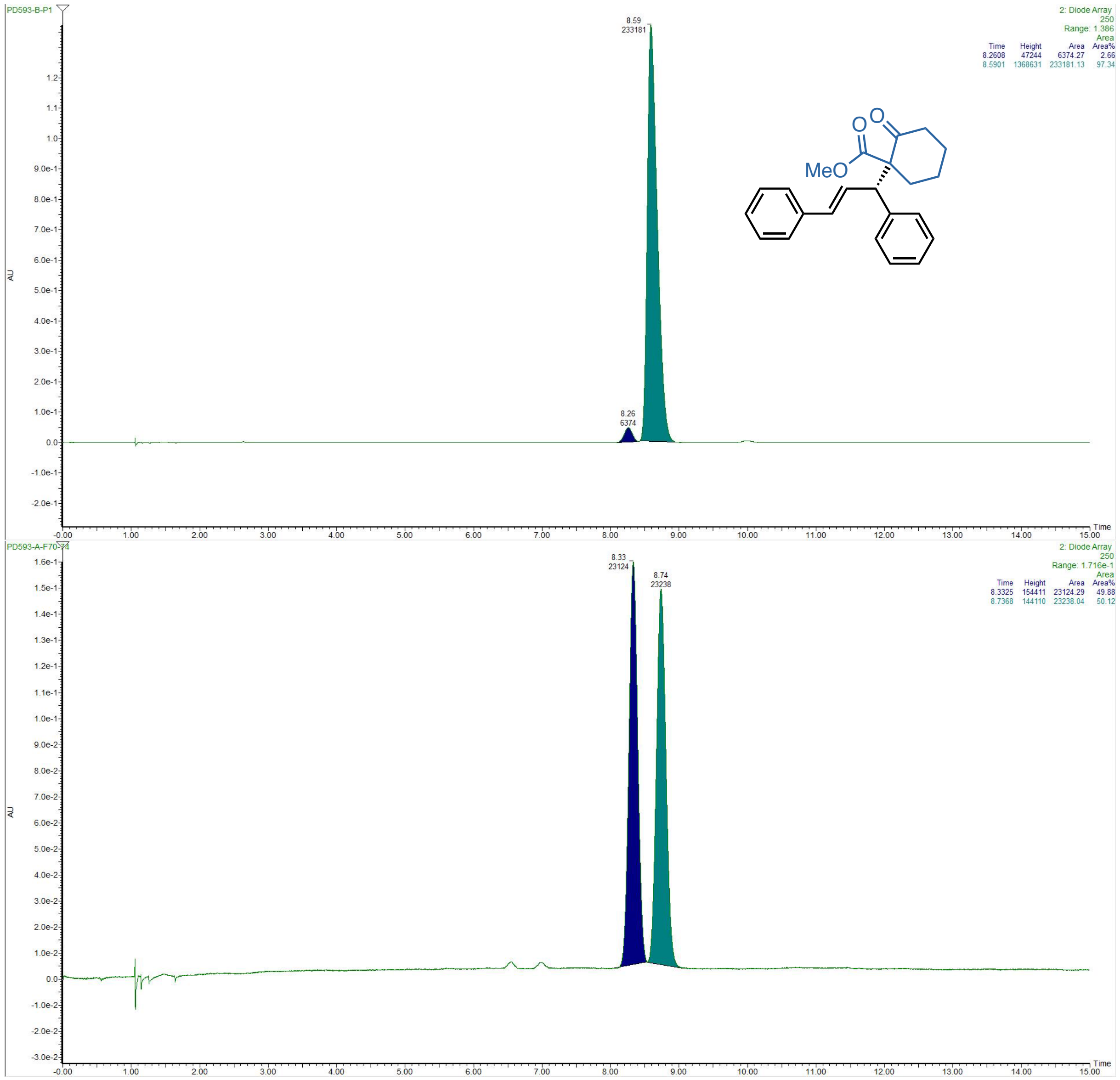

Methyl (*S*,*E*)-1-(1,3-diphenylallyl)-2-oxocyclohexane-1-carboxylate (3gb)

Chiral SFC Analysis CHIRAL PAK IG (CO_2:MeOH, 95:05, 2.5 mL min^{-1}, 40 °C) t_R = 9.92 (major), 12.16 (minor) minutes.

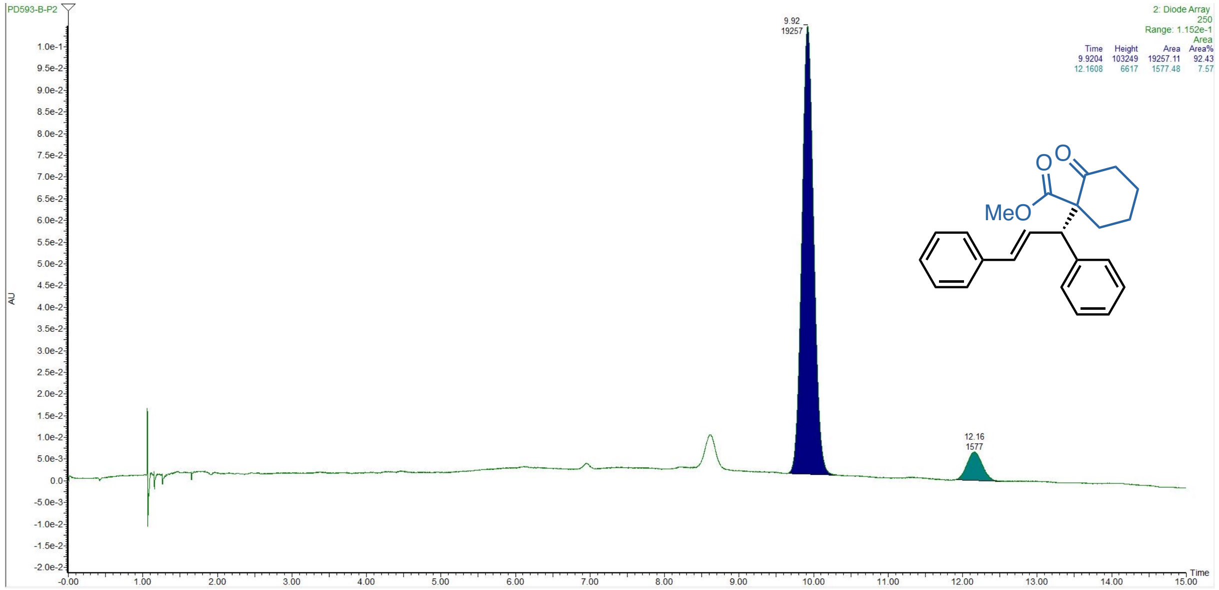

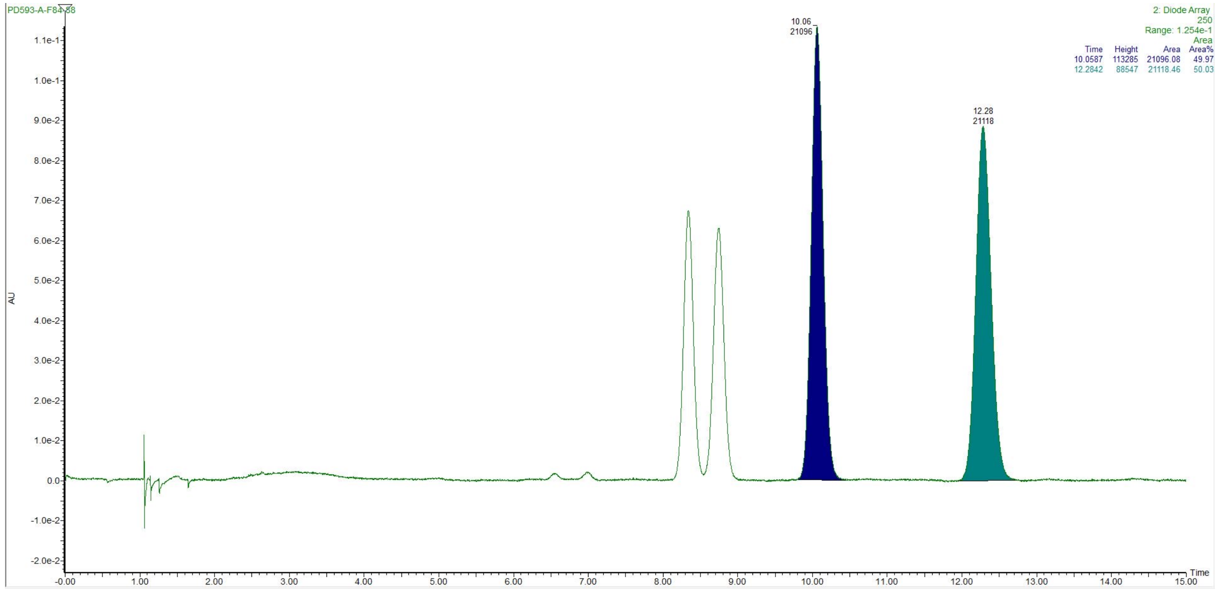

Methyl (*S*,*E*)-2-(1,3-diphenylallyl)-1-oxo-1,2,3,4-tetrahydronaphthalene-2-carboxylate (3ha)

Chiral SFC Analysis CHIRAL PAK IE (CO_2:MeOH, 90:10, 2.5 mL min^{-1}, 40 °C) t_R = 10.29 (minor), 11.72 (major) minutes.

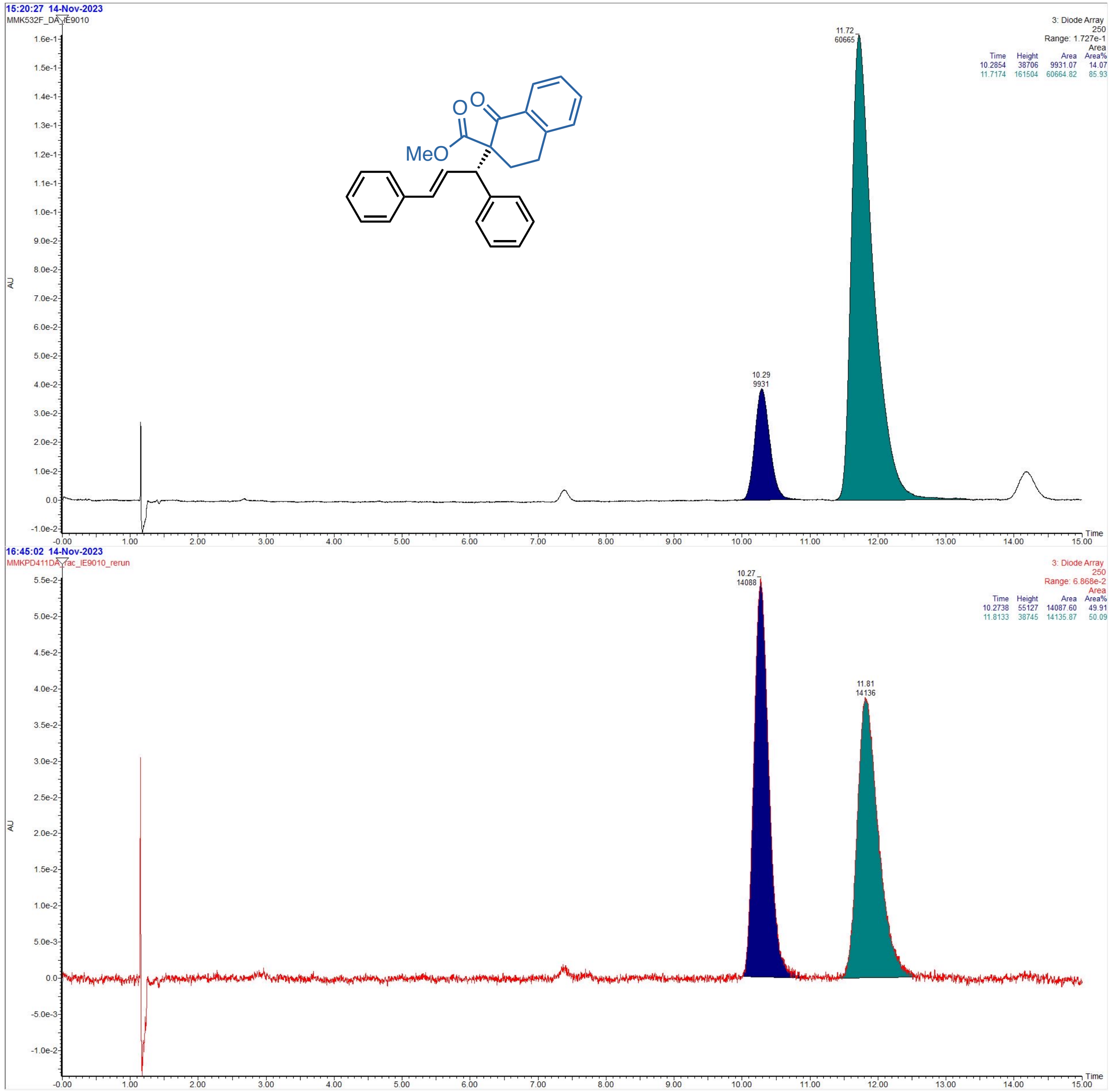

Methyl (*S*,*E*)-2-(1,3-diphenylallyl)-1-oxo-1,2,3,4-tetrahydronaphthalene-2-carboxylate (3hb)

Chiral SFC Analysis CHIRAL PAK IE (CO_2:MeOH, 90:10, 2.5 mL min^{-1}, 40 °C) t_R = 10.23 (minor), 11.67 (major) minutes.

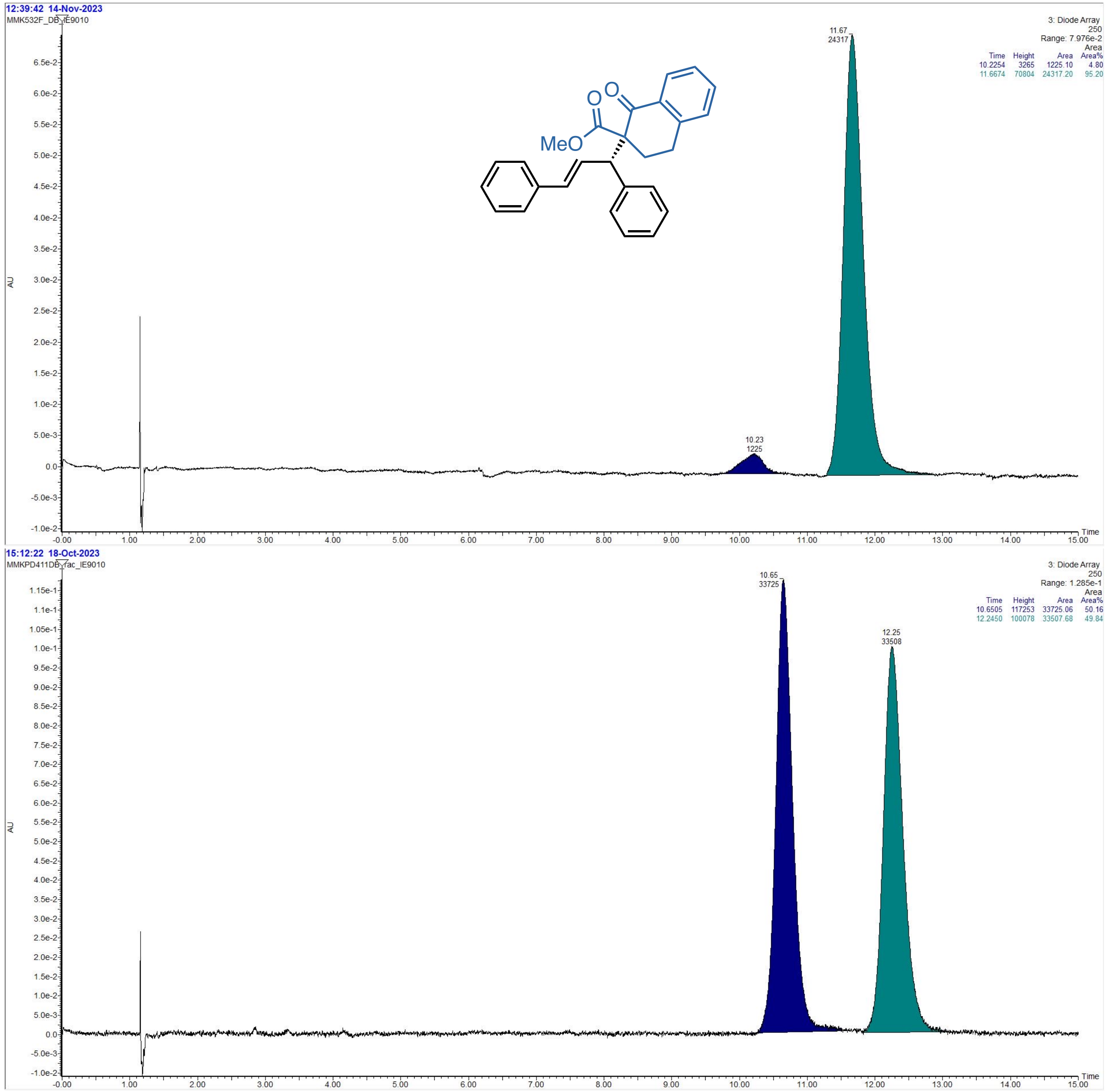

Ethyl (*S*,*E*)-2-ethyl-2-nitro-3,5-diphenylpent-4-enoate (3ia)

Chiral SFC Analysis CHIRAL PAK IG (CO_2:MeOH, 95:05, 2.5 mL min^{-1}, 40 °C) t_R = 4.26 (minor), 4.76 (major) minutes.

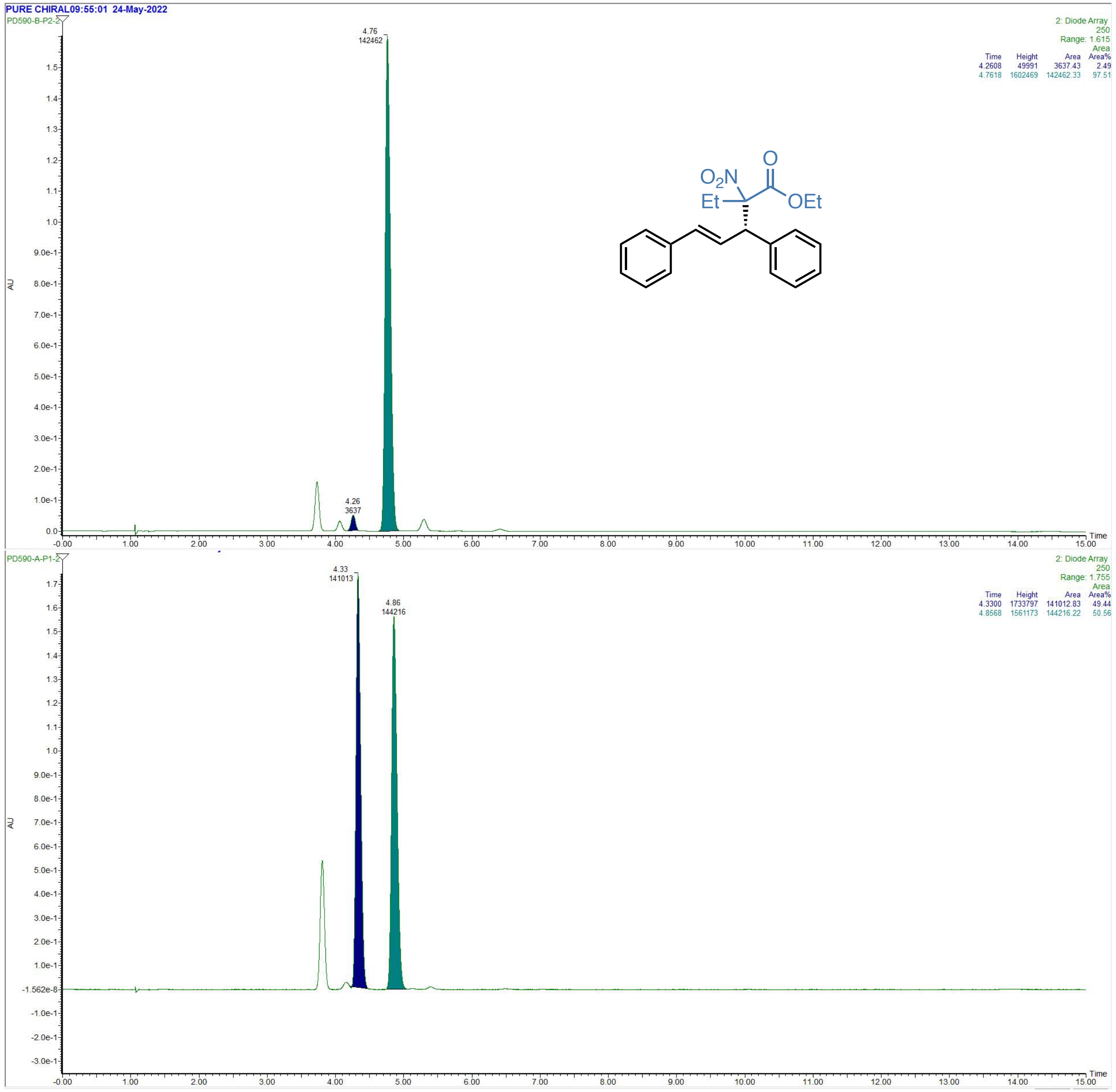

Ethyl (*S*,*E*)-2-ethyl-2-nitro-3,5-diphenylpent-4-enoate (3ib)

Chiral SFC Analysis CHIRAL PAK IJ (CO_2:MeOH, 99:01, 2.5 mL min^{-1}, 40 °C) t_R = 8.89 (major), 9.68 (minor) minutes.

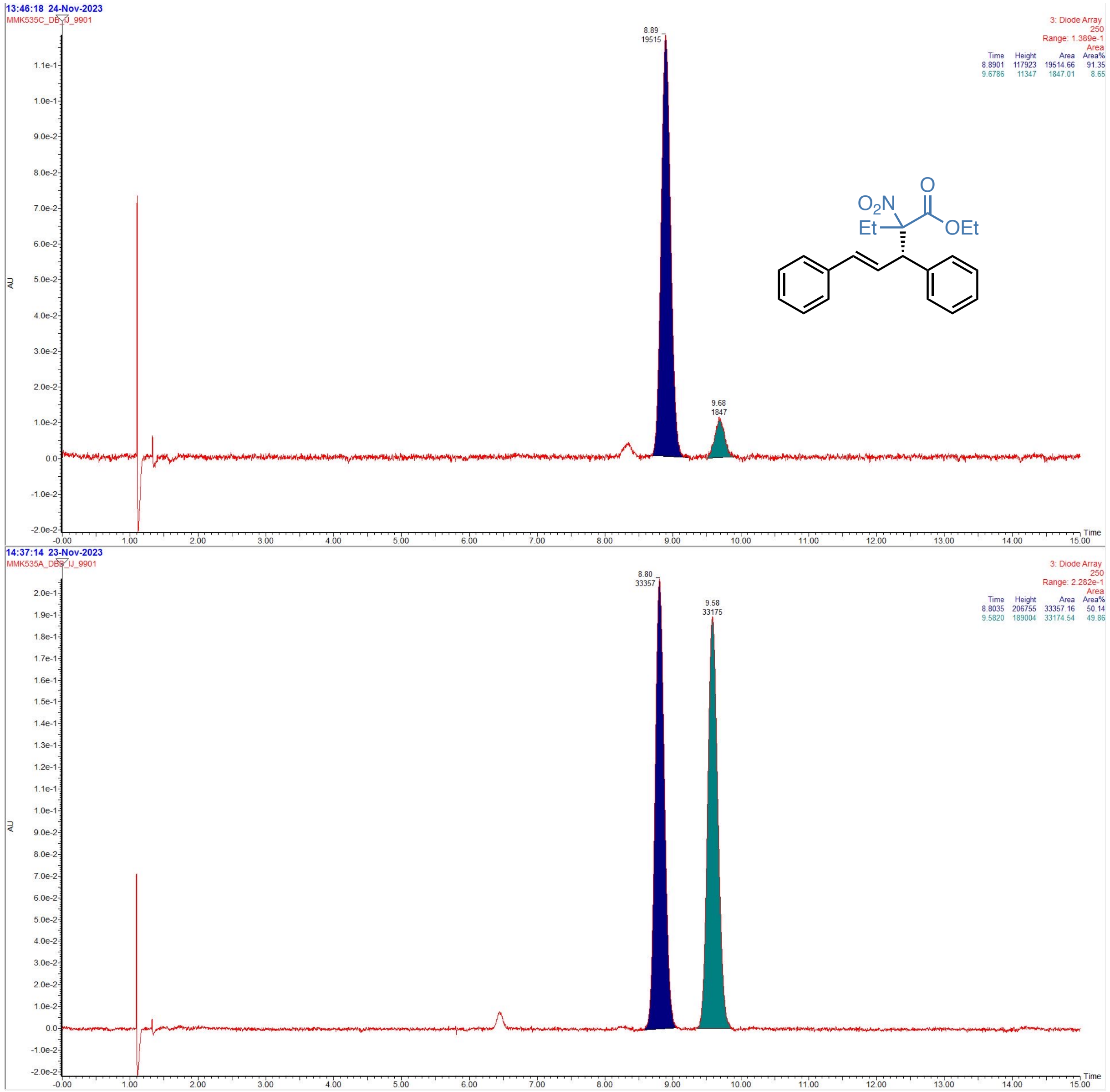

Ethyl (*S*,*E*)-2-nitro-3,5-diphenylpent-4-enoate (3ja + 3jb)

Chiral SFC Analysis CHIRAL PAK IG (CO_2:MeOH, 95:05, 2.5 mL min^{-1}, 40 °C) *Diastereomer A* t_R = 6.06 (minor), 6.39 (major) minutes. *Diastereomer B* t_R = 5.48 (minor), 6.87 (major) minutes.

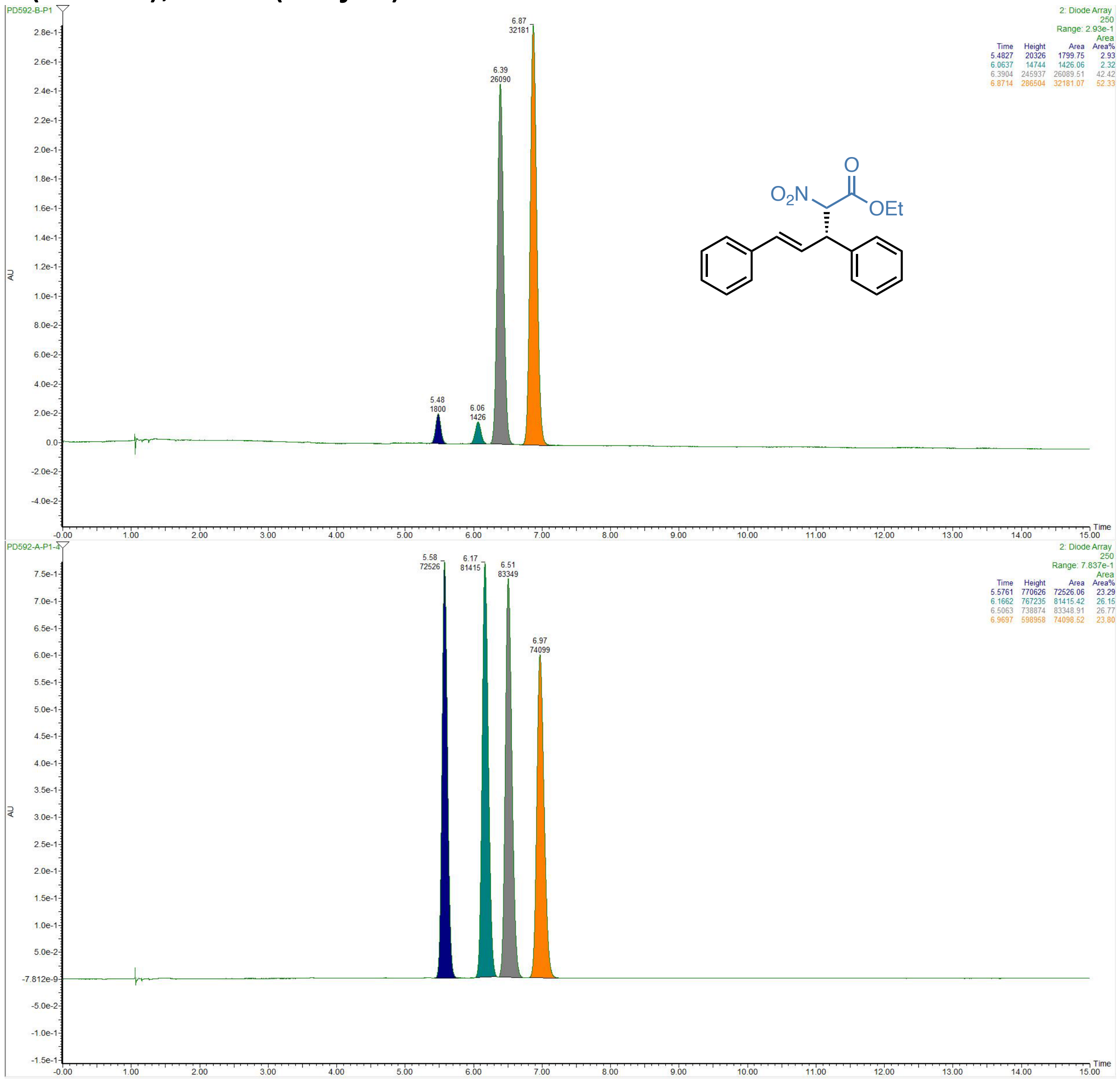

(*E*)-2-(1,3-diphenylallyl)-1,3-Diphenylpropane-1,3-dione (3k)

Chiral SFC Analysis CHIRAL ART SC (CO_2:MeOH, 90:10, 2.5 mL min^{-1}, 40 °C) t_R = 8.71 (major), 11.31 (minor) minutes.

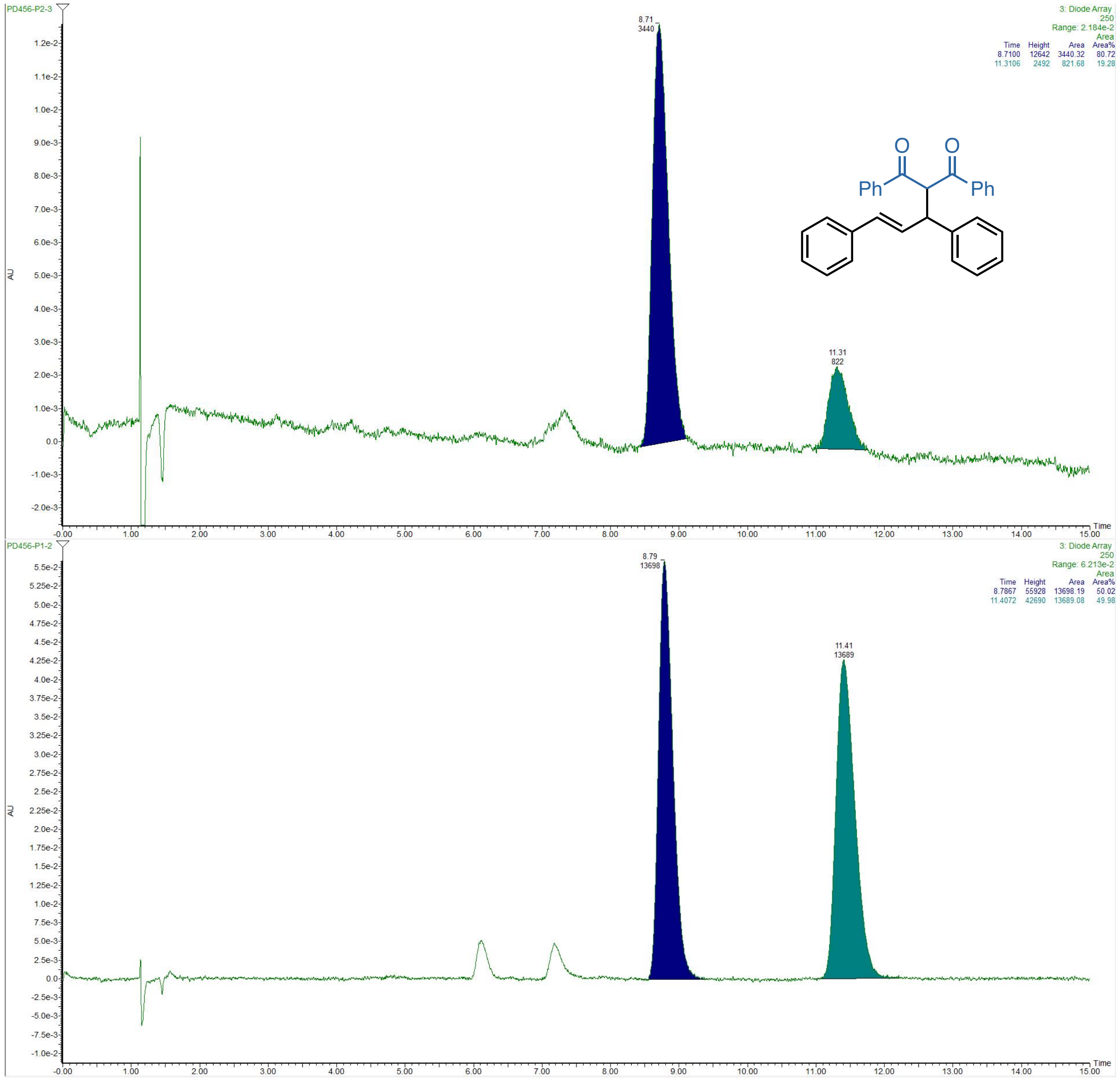

(*E*)-(4,4-bis(phenylsulfonyl)but-1-ene-1,3-diyl)Dibenzene (3l)

Chiral SFC Analysis CHIRAL PAK IG (CO_2:*i*-PrOH, 75:25, 2.5 mL min^{-1}, 40 °C) t_R = 10.06 (major), 12.70 (minor) minutes.

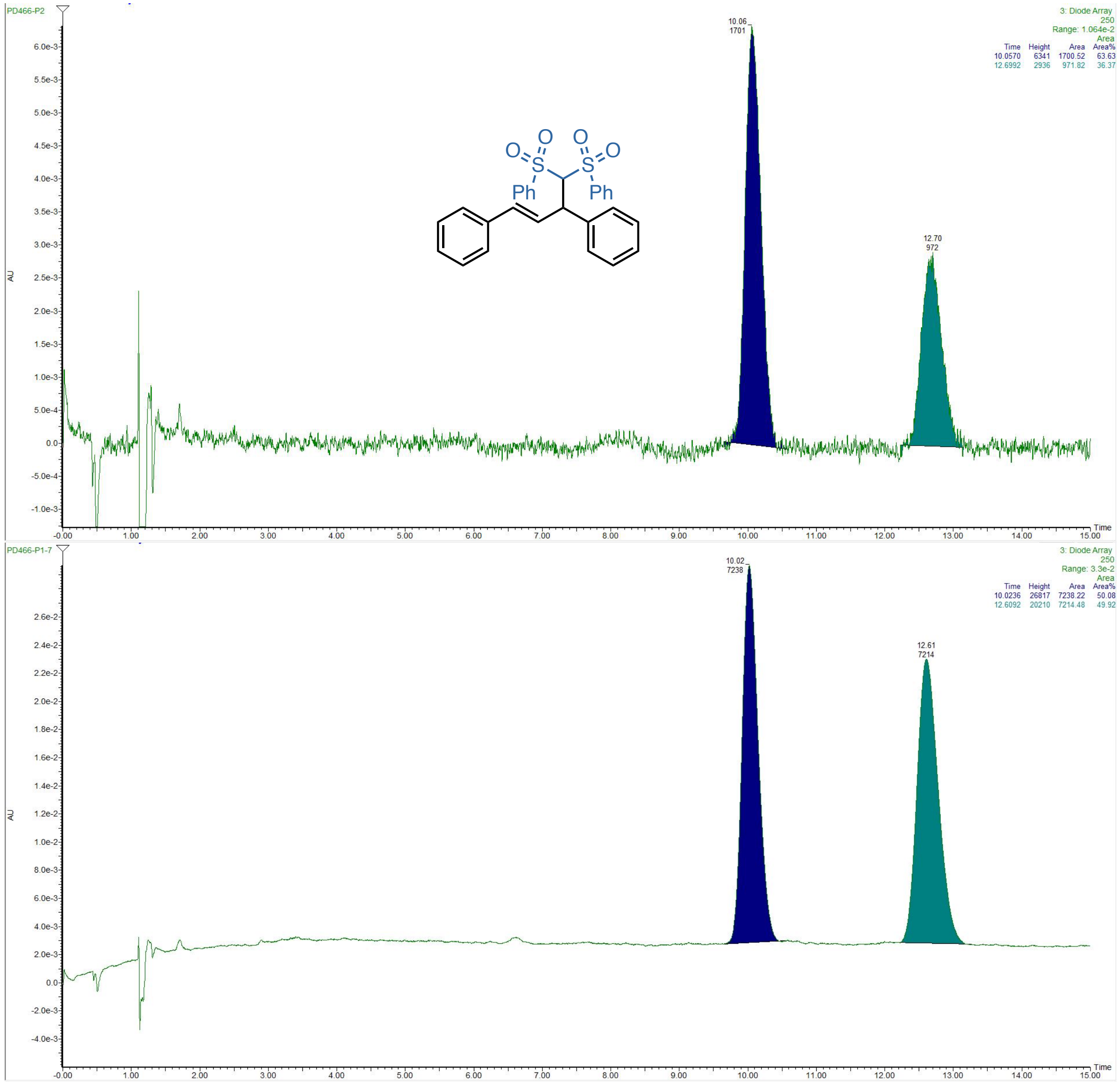

(*E*)-3-(1,3-diphenylallyl)-1*H*-indole (3m)

Chiral SFC Analysis CHIRAL PAK IK (CO_2:MeOH, 85:15, 2.5 mL min^{-1}, 40 °C) t_R = 8.65 (minor), 8.95 (major) minutes.

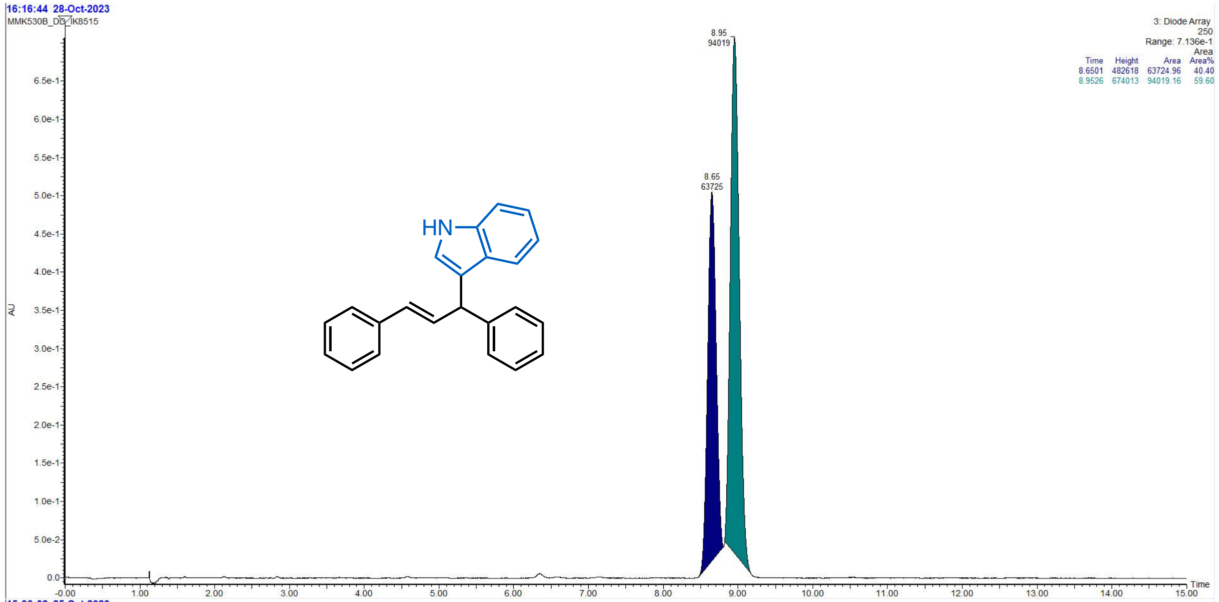

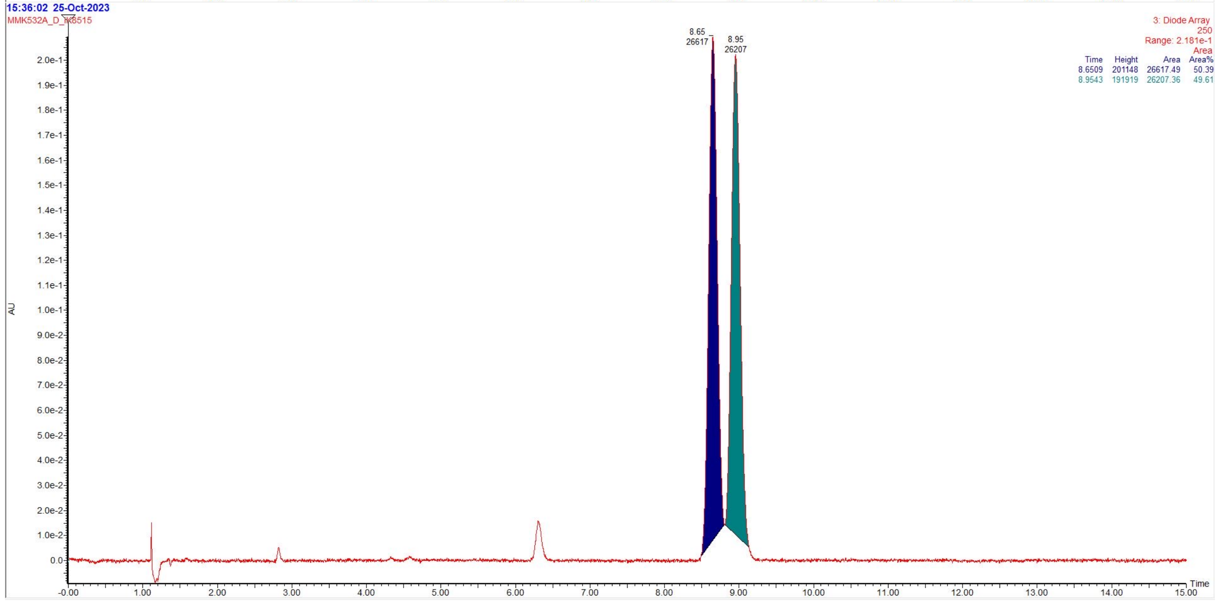

Methyl 1-cinnamyl-2-oxocyclohexane-1-carboxylate (3p)

Chiral SFC Analysis CHIRAL ART SJ (CO_2:*i*-PrOH, 99:01, 2.5 mL min^{-1}, 40 °C) t_R = 6.13 (minor), 7.37 (major) minutes.

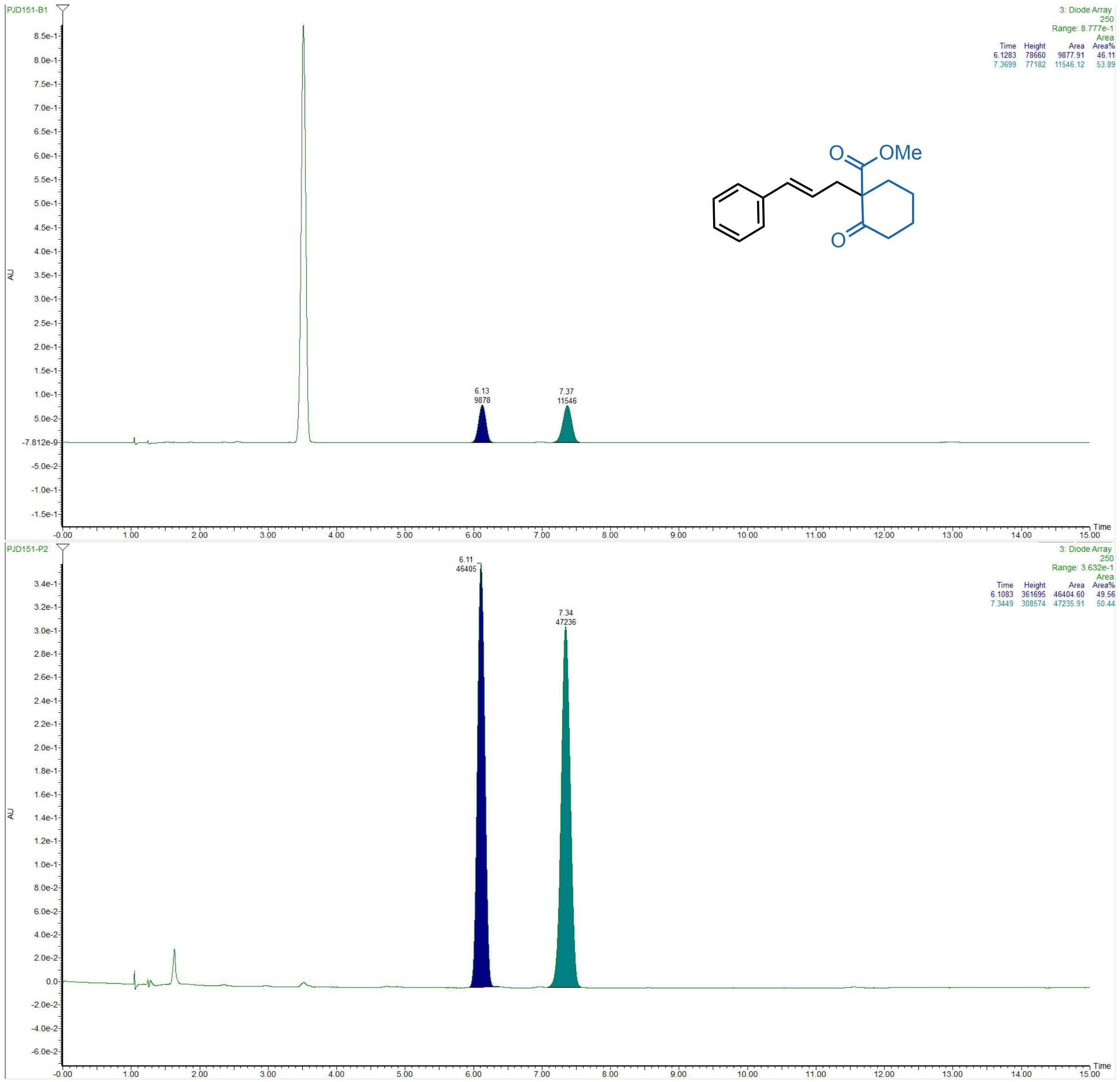

Dimethyl (*E*)-2-(4-phenylbut-3-en-2-yl)malonate (3q)

Chiral SFC Analysis CHIRAL PAK IG (CO_2:MeOH, 95:05, 2.5 mL min^{-1}, 40 °C) t_R = 4.38 (major), 5.35 (minor) minutes.

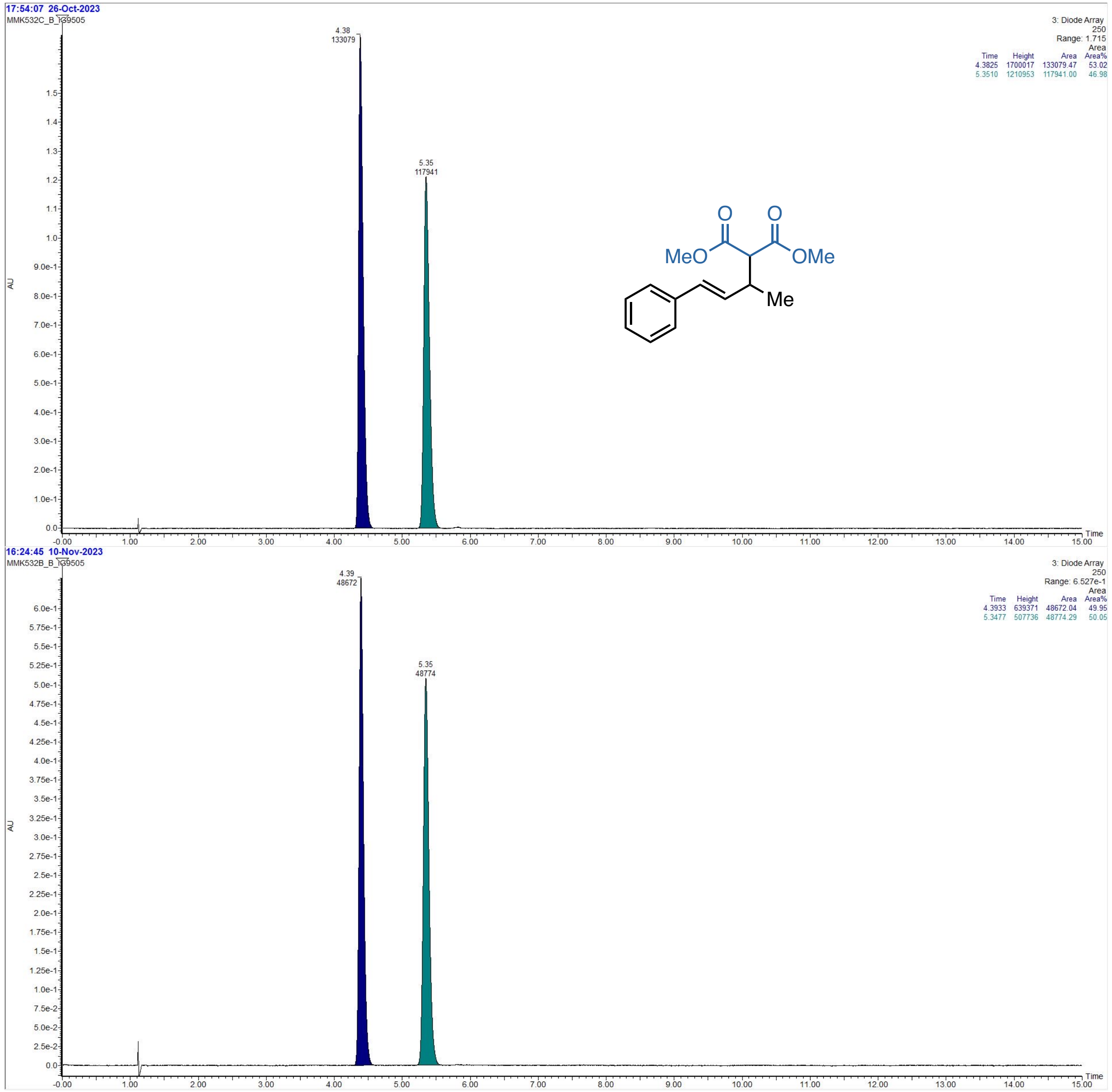

Supplement: Supplementary file 1 — ol3c04025_si_001.pdf [file ol3c04025_si_001.pdf]
